# Reversible Structural Isomerization of Nature’s Water Oxidation Catalyst Prior to O–O Bond Formation

**DOI:** 10.1021/jacs.2c03528

**Published:** 2022-06-24

**Authors:** Yu Guo, Johannes Messinger, Lars Kloo, Licheng Sun

**Affiliations:** † Center of Artificial Photosynthesis for Solar Fuels and Department of Chemistry, School of Science, Westlake University, Hangzhou 310024, China; ‡ Institute of Natural Sciences, Westlake Institute for Advanced Study, Hangzhou 310024, China; § Department of Chemistry, Umeå University, Linnaeus väg 6 (KBC huset), SE-90187 Umeå, Sweden; ∥ Molecular Biomimetics, Department of Chemistry—Ångström Laboratory, Uppsala University, SE-75120 Uppsala, Sweden; ⊥ Department of Chemistry, School of Engineering Sciences in Chemistry, Biotechnology and Health, KTH Royal Institute of Technology, SE-10044 Stockholm, Sweden

## Abstract

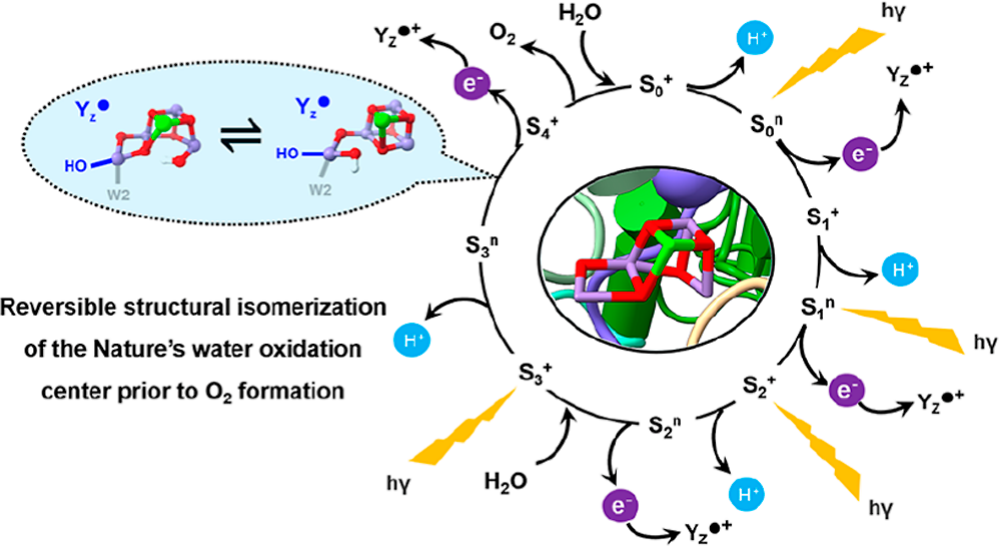

Photosynthetic water oxidation is catalyzed by a manganese–calcium oxide cluster, which experiences five “S-states” during a light-driven reaction cycle. The unique “distorted chair”-like geometry of the Mn_4_CaO_5(6)_ cluster shows structural flexibility that has been frequently proposed to involve “open” and “closed”-cubane forms from the S_1_ to S_3_ states. The isomers are interconvertible in the S_1_ and S_2_ states, while in the S_3_ state, the open-cubane structure is observed to dominate in*Thermosynechococcus elongatus* (cyanobacteria) samples. In this work, using density functional theory calculations, we go beyond the S_3_^+^Y_*z*_ state to the S_3_^*n*^Y_*z*_^•^ → S_4_^+^Y_*z*_ step, and report for the first time that the reversible isomerism, which is suppressed in the S_3_^+^Y_*z*_ state, is fully recovered in the ensuing S_3_^*n*^Y_*z*_^•^ state due to the proton release from a manganese-bound water ligand. The altered coordination strength of the manganese–ligand facilitates formation of the closed-cubane form, in a dynamic equilibrium with the open-cubane form. This tautomerism immediately preceding dioxygen formation may constitute the rate limiting step for O_2_ formation, and exert a significant influence on the water oxidation mechanism in photosystem II.

## Introduction

**Figure 1:**
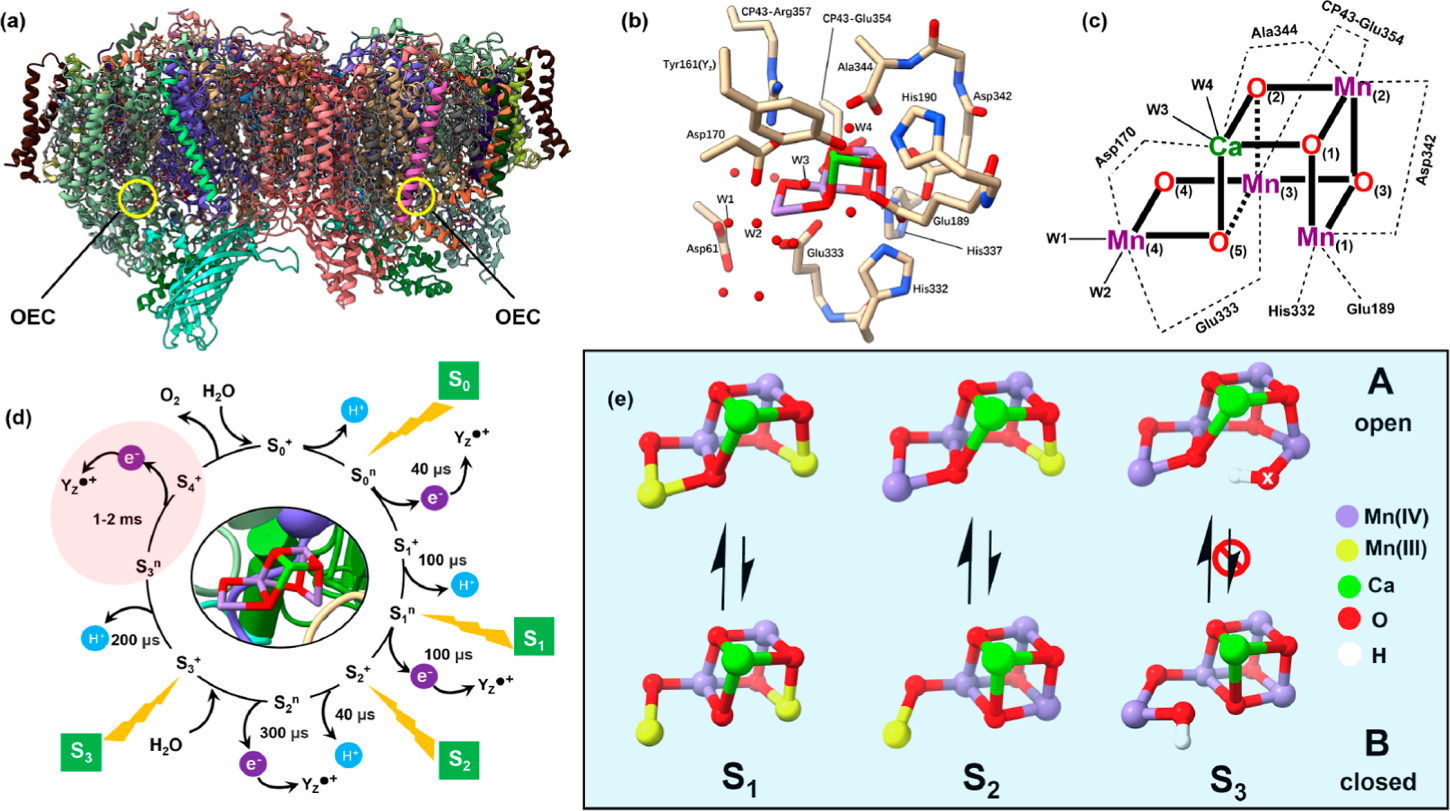
(a) View of PSII dimer and the OEC location from *Thermosynechococcus elongatus* (PDB ID: 6W1O)^38^ (b) Mn_4_CaO_5_ cluster and its local surroundings in its dark-stable S_1_ state. (c) Sketch map of atom labeling and connectivity of the first coordination sphere ligands in the Mn_4_CaO_5_ cluster. (d) Extended S-state cycle including nine intermediates with sequence of proton and electron transfer and kinetics between transitions;^3,13−15,17,21,22,70^ the red phase is the main focus of this study. (e) Structural flexibility of the OEC cluster in the S_1_, S_2_, and S_3_ states, marked with the reversibility between open (A) and closed (B) cubane structures (for references, see the main text).

Photosystem II (PSII) is a metalloenzyme that catalyzes water splitting to molecular oxygen in cyanobacteria, algae, and plants. It evolved about 3 billion years ago at the level of ancient cyanobacteria (Figure 1a). The embedded “oxygen-evolving complex (OEC)”, composed of a Mn_4_CaO_5_ cluster surrounded by water and amino acid ligands (Figure 1b,c), acts as a highly efficient water oxidation catalyst. Due to charge separations in the reaction center of PSII, the OEC is initially stepwise oxidized during the cyclic catalysis, so that it attains four (meta)stable intermediates (S_0_, S_1_, S_2_, and S_3_) and one transient S_4_ state, the latter of which initiates O_2_ formation.^1−10^ Accounting also for proton release and charge of the Mn_4_CaO_5(6)_ complex, the classical five-step “S-state cycle”^11^ can be refined to instead include nine intermediate states that are separated by kinetically distinguishable proton and electron transfer steps (Figure 1d).^3,12−22^

Structural polymorphism of the OEC has been proposed and experimentally observed, mainly by electron paramagnetic resonance (EPR) spectroscopy, for some decades.^1,4,18,23−30^ More recently, the first detailed theoretically models were proposed for interpreting these findings.^31,32^ However, the proposed alternative structures have thus far eluded verification by structural methods such as protein crystallography.^33−40^ The structural flexibility in the S_2_ state is typically attributed to the mobile μ-oxo bridge (O5) between Mn1 and Mn4,^31,41^ producing “open” (A) and “closed” cubane (B) forms of the cluster (Figure 1e, see supplementary references in the Supporting Information). As recently discovered by Pantazis and co-workers, orientational Jahn–Teller isomerism in the resting S_1_ state^41^ generates the precursors for the two interconvertible A and B structures of the S_2_ state,^31^ which give rise to the low-spin (S = 1/2) and high-spin (S = 5/2) EPR signals in plant PSII at *g* = 2 and *g* ≈ 4.1, respectively, and the latter *g* ≈ 4.1 (and similar signals around this value) can only be produced by mutations or chemical treatments in cyanobacteria.^42^ These authors also proposed that the closed-cubane form is the entry to the S_3_ state,^43,44^ in agreement with molecular dynamics studies by Guidoni and co-workers.^32,45^ This closed-cubane interpretation for the S_2_ high-spin (S = 5/2) state is widely accepted in the field and consistent with the calculations in this report and will hence be employed in this study. However, we note that two competing interpretations exist. First, based on broken-symmetry density functional theory (BS-DFT) calculations with focus on spectroscopic parameter analysis, Corry and O’Malley proposed an isomer in the S_2_ state by W1 deprotonation to μ-O4 to rationalize the high-spin (S = 5/2) form^46^ and, on this basis, further identified a high-spin (S = 7/2) deprotonated intermediate with μ-hydroxo O4 during the S_2_ → S_3_ transition, without invoking a closed-cubane structure.^47^ Second, another model for the high-spin (S = 5/2) S_2_ state assumes the early binding of a substrate water to Mn1 as OH^–^ originating from W3, as suggested by Siegbahn^48^ and later by Pushkar et al.^49^ For a detailed discussion of such models, see Text S6 in the Supporting Information.

The open-cubane S_3_ structure contains an extra oxygen ligand to Mn1 due to binding of an additional water molecule. This was proposed first by Siegbahn on the basis of DFT calculations^50−53^ that are found on the results from extended X-ray absorption fine structure (EXAFS) experiments,^54−56^ showing that the S_2_–S_3_ transition involves the conversion of a five-coordinate Mn(III) to a six-coordinate Mn(IV). Cox et al. confirmed by advanced EPR that all Mn ions in the S_3_ state are hexa-coordinate and that the “water-added” open-cubane S_3_ structure, S_3_^A,W^ (“W” denotes the extra water binding), is consistent with their experimental data.^57^ Isobe et al. constructed multiple S_3_ models^58,59^ that vary with regard to total spin and Mn-valence and proposed that the closed-to-open cubane transformation is possible in a stepwise process involving an oxyl–oxo precursor.^60^ By contrast, Capone et al.^61^ and Shoji et al.^62^ showed different feasible pathways for a direct closed-to-open cubane conversion. Regardless of the mechanistic details, a consensus has been reached that the OEC cluster in the S_3_ state (more precisely the S_3_^+^Y_*z*_ state, see below) allows for unidirectional conversion from the water-added closed (S_3_^B,W^) to the open-cubane (S_3_^A,W^) form, but not vice versa (Figure 1e). S_3_^B,W^ (S = 3) is proposed to be the precursor form of the final S_3_^A,W^ (S = 3) under the pivot/carousel mechanism of water binding during the S_2_ → S_3_ transition.^43,44,63^ Importantly, the dominance of the open-cubane Mn core topology is consistent with the S_3_ state structures resolved by serial crystallography using X-ray free electron lasers (XFELs).^35−38^

Nevertheless, alternative S_3_ state models that assume early O–O bonding exist.^1,23,64^ For example, Corry and O’Malley proposed a chemical equilibrium between “oxo-hydroxo” and “peroxo” for O5–Ox in the S_3_ state, based on a comparison of experimental and BS-DFT calculated geometries and magnetic resonance properties.^65−67^ In higher-plant PSII, a recent combined EPR and DFT study by Zahariou et al. provided evidence, in PSII isolated from spinach for S_3_ being a mixed state of S_3_^A,W^ (S = 3) and S_3_^B,unbound^ (S = 6) (“unbound” denotes the unsaturated coordination of Mn4; “S_3_^B,unbound^” is used throughout to refer to the “S_3_^B^” in its original publication, and similarly S_4_^B,unbound^ for S_4_^B^).^68,69^ Here, the dominant state (∼80%) has been identified as the S_3_^B,unbound^ state, that is, a closed-cubane S_3_ state with penta-coordinate Mn4(IV) without additional bound water. In this view, it should be emphasized that the structural isomerism in the S_3_ state introduced here (and discussed later) should apply to that of cyanobacterial PSII, and the less populated S_3_ form in higher plants.

**Scheme 1:**
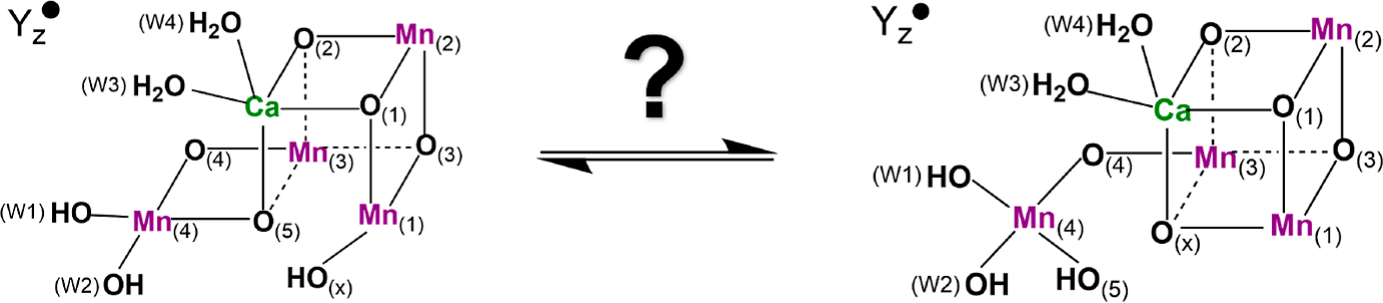
Structural Isomerization between S_3_^A,W^Y_*z*_^•^ (Left) and S_3_^B,W^Y_*z*_^•^ (Right) in the S_3_^*n*^Y_*z*_^•^ (W1 = OH^–^) State Explored in the Present Study Amino acid ligands are omitted for clarity.

Consistent with the abovementioned findings for the S_3_^+^Y_*z*_ state, it is commonly assumed that the O–O bond formation in the S_4_^+^ state also occurs in the open-cubane (S_4_^A,W^) conformation.^4,35,37,51,53,57,71−79^ However, there have been also several proposals based on a closed-cubane structure (S_4_^B,W^ or S_4_^B,unbound^),^4,68,69,73,75,80−87^ which is in sharp contrast in terms of geometric configuration. This motivates us to investigate if structural heterogeneity exists just before the S_4_^+^ state is formed from the S_3_^*n*^ state via electron abstraction by Y_*z*_^•^. In this paper, we mainly focus on the possibility of structural isomerization in the S_3_^*n*^Y_*z*_^•^ states (Scheme 1), employing DFT calculations. The correlation of our results with experimental observations and the implications for the mechanism of O–O bond formation are discussed.

## Results and Discussion

### Unidirectional Structural Isomerization in the S_3_^+^Y_*z*_ State

**Figure 2:**
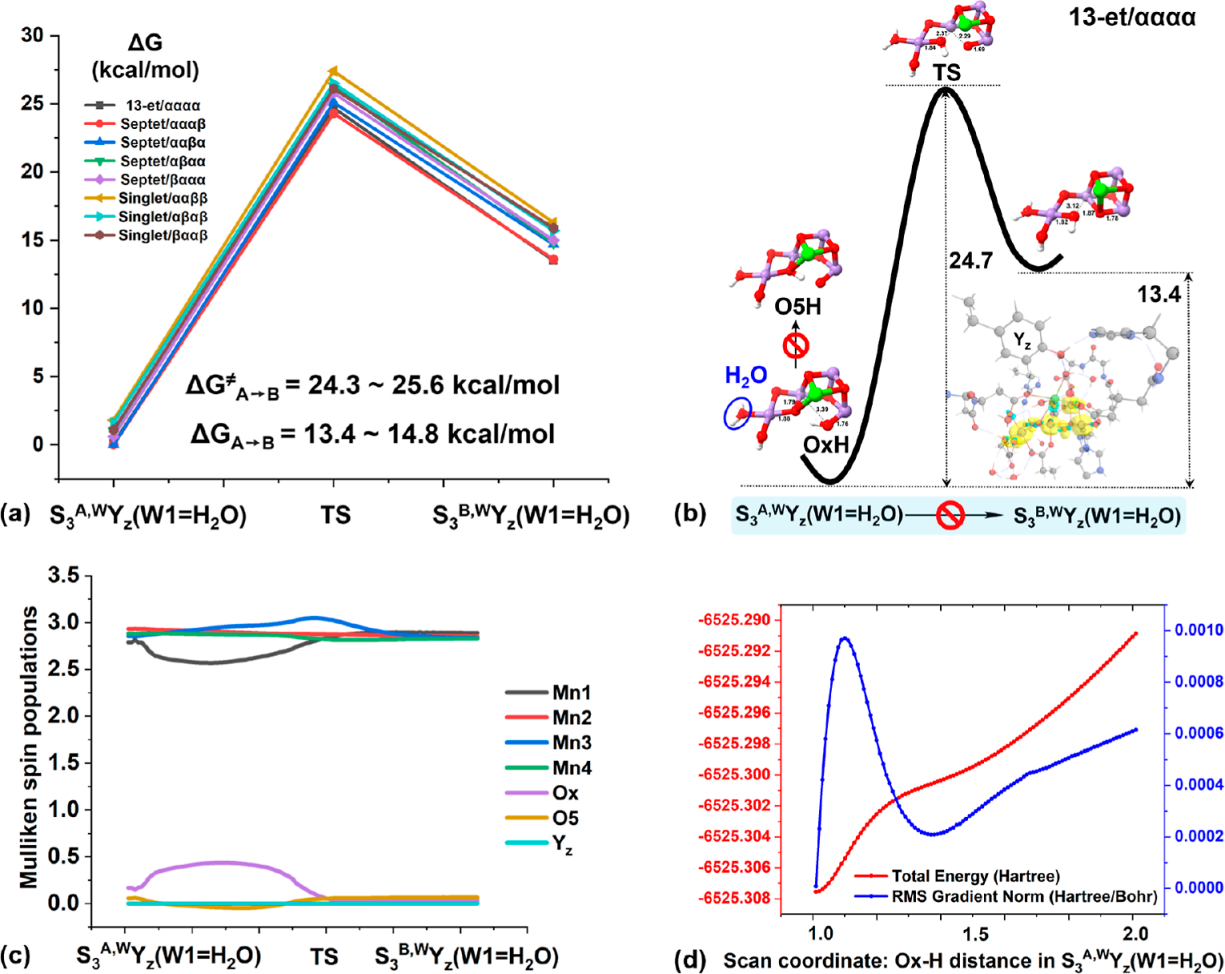
(a) Relative Gibbs free energy profiles for the conversion between S_3_^A,W^Y_*z*_(W1=H_2_O) and S_3_^B,W^Y_*z*_(W1=H_2_O) in all the possible spin states of the S_3_^+^Y_*z*_ state. Because of the close similarity to the other spin states, more information regarding the changes of (b) geometric structures, (c) electronic configurations along the MEP, and (d) relaxed PES scan curve of proton transfer between Ox and O5 are exemplified in the highest 13-et/αααα spin state. Spin populations are displayed in yellow contours and key interatomic distances are given in Å.

Unlike the structural interconversion simply caused by O5 shuttling between Mn1 and Mn4 in the S_2_ state (the most likely mechanism accounting for the EPR isomers, see Text S6 in the Supporting Information), isomerization in the S_3_^+^Y_*z*_ state results from Mn3 ligand exchange between O5 and Ox. We revisited this process by our quantum chemical model (Figure S1 in the Supporting Information) and determined a direct conversion pathway connecting the open and closed-cubane structures. A notable phenomenon is that the incidental proton transfer is directed toward the moiety becoming a terminal ligand; protonation of the μ-oxo ligand is impossible in either isomer (O5H in S_3_^A,W^ or OxH in S_3_^B,W^), which is justified by the relaxed potential energy scan for proton translocation between Ox and O5 (Figure 2d, Text S1 in the Supporting Information).

In contrast to Capone et al.,^61^ where the formal oxidation state of Mn3 is lowered from (IV) to (III), while Mn1 acquires a partial radical character in the proximity of the transition state (TS), our results show that the electronic configuration of the OEC cluster essentially remains constant along the minimum energy paths (MEPs). That means all Mn keep valence (IV) throughout as reflected by the Mulliken spin populations (Figure 2c, Text S2 and Table S2 in the Supporting Information). The reason may be attributed to the exclusion of structural and thermal fluctuations along the MEPs, which are instead present during the molecular dynamics simulations. Anyhow, consistent with Isobe et al.,^60^ our calculated S_3_^A,W^ → S_3_^B,W^ barriers of 24.3–25.6 kcal/mol and stabilization energies of 13.4–14.8 kcal/mol for the open-cubane form (S_3_^A,W^) on all the spin surfaces (Figure 2a,b, Table S1 in the Supporting Information) show that the same conclusion can be drawn, that is, conformational change in the S_3_^+^Y_*z*_ state is essentially confined to the unidirectional closed-to-open cubane conversion and forbidden reversely. Consequently, the bidirectional structural flexibility prevalent in both S_1_ and S_2_ states has disappeared in the following S_3_^+^Y_*z*_ state, with an overwhelming preference for the open-cubane structure.

It is worth mentioning that the abovementioned conclusion is strictly only valid for the S_3_^+^Y_*z*_ state and does not apply for the S_2_^+^Y_*z*_^•^ state, which can be formed from the S_3_^+^Y_*z*_ state by electron back donation from Y_*z*_ to Mn under certain conditions, as shown in some experimental findings.^88−90^ The structural equilibration in the S_2_^*n*^Y_*z*_^•^ state was suggested as a requirement for water exchange in the S_3_^+^Y_*z*_ state,^91^ in which the open-to-closed conversion is involved and readily reversible, but necessitates a Mn(III) center within the cluster.

### Reversible Structural Isomerization in the S_3_^*n*^Y_*z*_^•^ State

**Figure 3:**
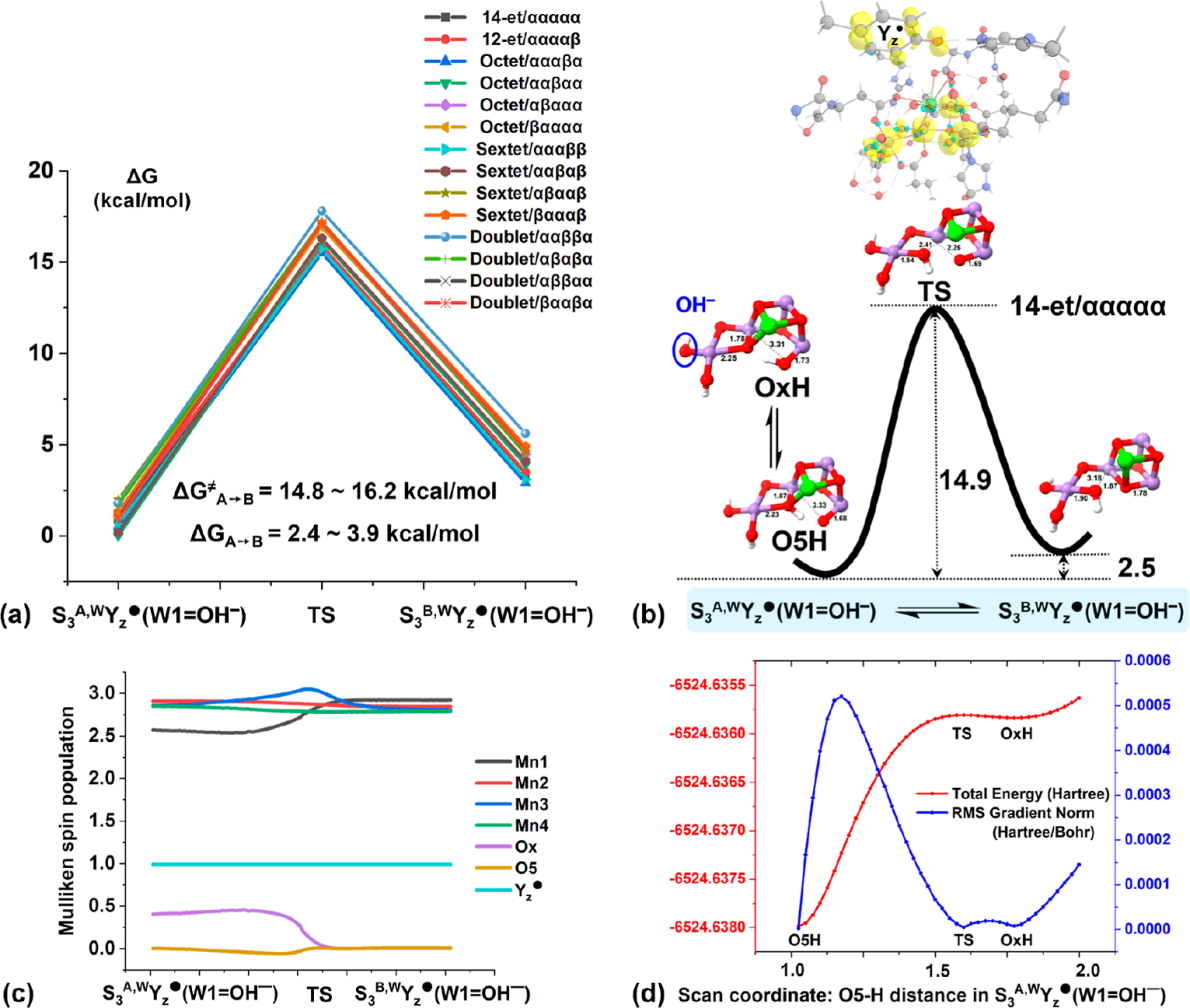
(a) Relative Gibbs free energy profiles for the conversion between S_3_^A,W^Y_*z*_^•^(W1=OH^–^) and S_3_^B,W^Y_*z*_^•^(W1=OH^–^) in all the possible spin states of the S_3_^*n*^Y_*z*_^•^ state. Because of the close similarity to the other spin states, the highest 14-et/ααααα spin state was selected for visualizing more information regarding the changes of (b) geometric structures and (c) electronic configurations along the MEP, and (d) relaxed PES scan curve of proton transfer between Ox and O5.

By various experimental approaches, it has been established that O_2_ formation and release begins after the flash-induced formation of the S_3_^+^Y_*z*_^•^ state only after a lag phase of about 200 μs.^12,70−92^ This lag phase has been assigned to a deprotonation reaction in the S_3_^+^ to S_3_^*n*^ transition, as shown in Figure 1d. Since the initial deprotonation site has been widely acknowledged as W1(H_2_O) (via the egress gate Asp61 to the lumen) during the S_3_ → S_4_ transition,^52,53,78,93−96^ this ligand was formulated as a hydroxide (OH^–^) in our S_3_^*n*^Y_*z*_^•^ model, in agreement with a series of previous computational work.^52,53,94,96^ In analogy to the abovementioned case of S_3_^+^Y_*z*_, redox-related events were not observed at any of the Mn centers along the whole MEP (Figure 3c and Table S4 in the Supporting Information) and various spin couplings do not significantly affect the energetics even after Y_*z*_^•^ addition. The redox-irrelevance and spin-insensitivity for such a ligand exchange are understandable because the octahedral coordination geometry of Mn3(IV) basically maintains during the simultaneous movements of O5 and Ox in opposite directions, and the two oxygens never approach a bonding distance to cause Mn reduction.

Interestingly, the obtained reaction landscapes of the structural isomerization in the S_3_^*n*^Y_*z*_^•^ state is fundamentally changed with regard to both thermodynamics and kinetics (Figure 3a,b, Table S3 in the Supporting Information) as compared to that of the S_3_^+^Y_*z*_ state (Tables S1 in the Supporting Information), allowing for a dynamically reversible isomerization S_3_^A,W^Y_*z*_^•^ ⇌ S_3_^B,W^Y_*z*_^•^ in chemical equilibrium. First, the relative thermodynamic stability of S_3_^B,W^Y_*z*_^•^ is greatly enhanced to only 2.4–3.9 kcal/mol higher in free energy than S_3_^A,W^Y_*z*_^•^ (vs 13.4–14.8 kcal/mol in the S_3_^+^Y_*z*_ state). Superficially, according to the relationship between Δ*G*° and equilibrium constant *K*, this energy difference would still correspond to a major population of S_3_^A,W^Y_*z*_^•^ in the equilibrium at room temperature; however, overemphasis on the precise quantitative population of the isomers in the S_3_^*n*^Y_*z*_^•^ state would be undesirable because of the calculated small energy gap and the well-known intrinsic limitations in the accuracy of DFT methodology,^97−100^ and the ambiguous direction of the equilibrium shifting given the consumption of S_3_^A,W^Y_*z*_^•^ and/or S_3_^B,W^Y_*z*_^•^ when proceeding to the S_4_ state. Thus, the isomerism suggested here in S_3_^*n*^Y_*z*_^•^ resembles the situations in the S_1_ and S_2_ states,^31,32,41^ where the closed-cubane structures are also deemed important for the catalytic progression despite the calculated slight energetic disadvantages compared with the open-cubane forms, that is, +3.2 kcal/mol for S_1_^B^ and +(1–2) kcal/mol for S_2_^B^ (see Text S3 in the Supporting Information for the detailed analysis).^26,31,32,41,48,101−104^ As a consequence of the markedly closer energies of the isomers in the S_3_^*n*^Y_*z*_^•^ state, the predominance of the open-cubane structure is undermined and the significance of S_3_^B,W^Y_*z*_^•^ should be highlighted in addition to S_3_^A,W^Y_*z*_^•^. Strictly speaking, one should not overlook the importance of either isomer in the S_3_^*n*^Y_*z*_^•^ state, considering the aforementioned uncertain factors that would lead to an indefinite identification of a dominant or most reactive component.

Besides the thermodynamics, the free energy barriers from S_3_^A,W^Y_*z*_^•^ to S_3_^B,W^Y_*z*_^•^ for all the possible spin states are dramatically reduced to 14.8–16.2 kcal/mol (vs 24.3–25.6 kcal/mol in the S_3_^+^Y_*z*_ state), which allows for smooth production of S_3_^B,W^Y_*z*_^•^ at a level of milliseconds kinetics (see Text S4 in the Supporting Information for more details). It is noteworthy that the direct reactant for the isomerization turns out to involve the protonated O5H, which is reachable by facile deprotonation from Ox and vice versa (Figure 3d); this remarkably contrasts the situation in the S_3_^+^Y_*z*_ state, where O5H is not achievable for S_3_^A,W^ (Figure 2d). These results show a feasible pathway from S_3_^A,W^Y_*z*_^•^ to S_3_^B,W^Y_*z*_^•^ preceded by Ox deprotonation and demonstrate that the structural heterogeneity lost in the S_3_^+^Y_*z*_ state becomes available again in the S_3_^*n*^Y_*z*_^•^ state. This leads to a more balanced constituent of the isomers, as compared to the dominance of the S_3_^A,W^ conformation and the high energetic barrier for isomerization in the S_3_^+^Y_*z*_ state.

### W1 Deprotonation Facilitates the Open-to-Closed Isomerization

**Figure 4:**
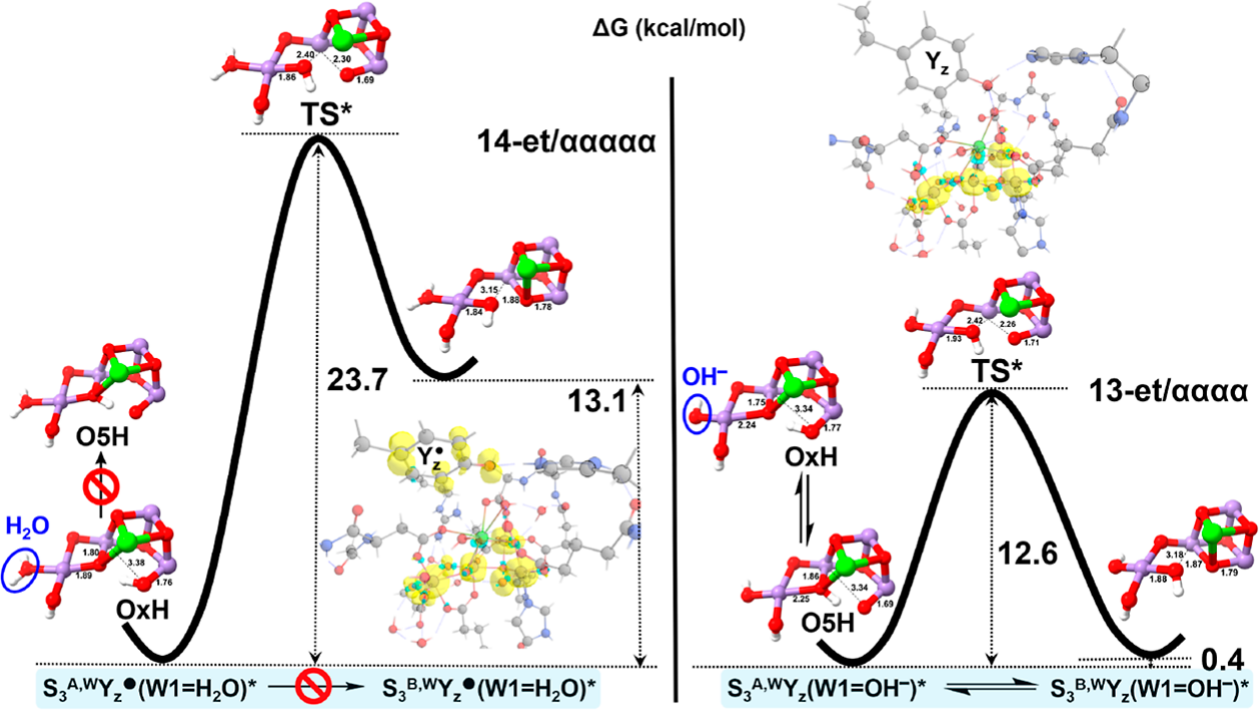
Relative Gibbs free energy profiles for the conversions between the virtual states S_3_^A,W^Y_*z*_^•^(W1=H_2_O)* and S_3_^B,W^Y_*z*_^•^(W1=H_2_O)* (left) and between the virtual states S_3_^A,W^Y_*z*_(W1=OH^–^)* and S_3_^B,W^Y_*z*_(W1=OH^–^)* (right) in their respective highest spin states; “*” denotes a virtual state.

As shown above, a magnitude of ca. 10 kcal/mol decrease in both barrier heights and relative energies from S_3_^+^Y_*z*_ to S_3_^*n*^Y_*z*_^•^ has largely changed the equilibrium distribution of the isomers. This is mainly manifested in the feasibility of S_3_^A,W^Y_*z*_^•^ converting to S_3_^B,W^Y_*z*_^•^, since B to A is attainable in both the S_3_^+^Y_*z*_ and S_3_^*n*^Y_*z*_^•^ states. Quite evidently, the S_3_^*n*^Y_*z*_^•^(W1=OH^–^) state is differentiated from S_3_^+^Y_*z*_(W1=H_2_O) by its oxidized Y_*z*_^•^ unit and deprotonated W1 ligand, that is, the asynchronous departure of an electron and a proton from two separated sites. Thus, two virtual states S_3_^+^Y_*z*_^•^(W1=H_2_O)* and S_3_^*n*^Y_*z*_(W1=OH^–^)* characterizing the single effect were artificially fabricated in order to clarify the ultimate reason for the observed difference. Since the spin state selectivity is expected to bring little impact on the isomerization, only the highest spin states were studied for a comparison, as shown in Figure 4.

The situation for the virtual S_3_^+^Y_*z*_^•^(W1=H_2_O)* and S_3_^*n*^Y_*z*_(W1=OH^–^)* states fairly coincides with that of the S_3_^+^Y_*z*_(W1=H_2_O) and S_3_^*n*^Y_*z*_^•^(W1=OH^–^) states, respectively, in terms of the reaction energetics and geometric parameters (Tables S5–S8 in the Supporting Information), as well as the proton mobility between Ox and O5 (Figures S2 and S3 in the Supporting Information). The comparison clearly reveals that it is the occurrence of W1(H_2_O) deprotonation, rather than appearance of the Y_*z*_^•^ radical, that substantially promotes the A to B isomerization in the S_3_^*n*^Y_*z*_^•^ state. This is reasonable because the covalent bonding interactions within the Mn_4_CaO_6_ cluster should be much more powerful than the electrostatic effect brought by the distal Y_*z*_^•^ group. Specifically, we expect that the strong σ donation from W1=OH^–^ reinforces its coordination to Mn4 but considerably weakens the O5–Mn4 bonding, due to the “structural trans effect” in octahedral transition metal complexes.^105−108^ The diminished overlap between the Mn4 3d and O5 2p orbitals can in turn stabilize the O5 2p–H s covalency, increasing the basicity of O5 and explaining the accessibility of O5 protonation in both S_3_^A,W^Y_*z*_^•^(W1=OH^–^) and S_3_^A,W^Y_*z*_(W1=OH^–^)*. Furthermore, the Mn3–O5 bond is weakened by the protonated O5H, which therefore becomes easier to be substituted by Ox (oxo). The altered bond strengths can be seen from variations of the key bond lengths and Wiberg bond orders (Table S9 in the Supporting Information). To sum up, the feasibility of the open-to-closed isomerization in the S_3_^*n*^Y_*z*_^•^ state is directly attributable to W1(H_2_O) deprotonation, which causes a series of subtle changes in Mn–ligand interactions.

Although Y_*z*_^•^ itself produces little chemical effect on the isomerization, its formation is necessary for the subsequent W1(H_2_O) deprotonation. After the third flash given to dark-adapted PSII, the S_3_^+^Y_*z*_^•^ state forms accompanied by deprotonation of the phenolic oxygen of Y_*z*_ to the ε-nitrogen of His190, and thus an extra positive charge accumulates within the vicinity of the Mn_4_CaO_6_ cluster. Thereafter, the Mn4-bound W1 serves as an ideal deprotonation site for charge compensation because it is in strong hydrogen-bonding interaction with the negatively charged D1-Asp61, which connects further to the proton exit channel and the lumen.^39,52,56,81,95,109−114^ Thereby, the occurrence of Y_*z*_ oxidation is an essential prerequisite for the reversible structural isomerization in the S_3_Y_*z*_^•^ state, from a perspective of the causal relationship.

It is noted that up to date, there is still no unambiguous/conclusive assignment for the protonation states of the titrable groups (especially W2 and Ox) of the OEC cluster in the S_3_^+^Y_*z*_ and S_3_^*n*^Y_*z*_^•^ states; however, our models adopt the protonation states suggested by Cox et al.,^57^ which reproduced the experimental EPR and electron–nuclear double resonance (ENDOR) and electron–electron double resonance-detected nuclear magnetic resonance (EDNMR) of the S_3_ state, and are in good agreement with most computational studies.^51,60,61,91,94,115−118^ Still, we have also performed extensive additional computations and found that our conclusion still holds even if different protonation state distributions were considered (see Text S5 and Tables S10–S14 in the Supporting Information for the details).

### Alternative Computational S_3_ State Models

For the S_3_^+^Y_*z*_ and S_3_^*n*^Y_*z*_^•^ states, Corry and O’Malley proposed “oxo–hydroxo ⇌ oxo–oxo ⇌ peroxo” and “oxo–oxo ⇌ peroxo” equilibria to describe the chemical nature of “O5–Ox” in S_3_^+^Y_*z*_ and S_3_^*n*^Y_*z*_^•^, respectively.^65,67^ Early O–O bond formation in the S_3_ state was also explored by Pushkar et al.^64^ and Isobe et al.^58,59^ We note that the “oxo-hydroxo” model with all octahedral Mn(IV) employed in this study adequately fits the vast majority of results from EXAFS,^5,116,119,120^ EPR/ENDOR/EDNMR, and X-ray absorption and emission spectroscopies in the S_3_ state.^57,118,121^ In contrast, while it still remains unclear if the “oxo–oxo” model is consistent with these spectroscopic data, the “peroxo” model would produce two anisotropic Mn(III) and thus is clearly inconsistent with the experimental observations. It has been also ruled out by all the latest XFEL experiments with updated essential details (e.g., O5–O6/Ox distance),^36−39^ despite support from one initial study.^35^ The calculated S = 4 ground state of the peroxo model^65^ does not agree with the S = 3 signal observed experimentally.^57^ On the basis of substrate-water exchange,^24,122^ although we cannot fully exclude the “peroxo” model given the option of suitable structural/redox equilibria, obviously a stable peroxide can be ruled out. From the aspect of computational modeling, calculations by coupled cluster theory, which is beyond traditional DFT, also strongly disfavors the scenario based on an early-onset O–O bond formation in the S_3_ state.^123^

However, it remains possible that the “peroxo” form could constitute part of the redox equilibria in the S_3_ state and it might be catalytically relevant, but it should not represent the dominant form in the S_3_ state. For the S_3_^*n*^Y_*z*_^•^ state, the “peroxo” model was indeed considered as one possible option because water exchange dramatically slows down as compared to S_3_,^124^ but the model was also ruled out by the authors in that report because of the inconsistency with the results from time-resolved X-ray experiments.^3,70,124^ Still, we have in detail considered the “oxo–oxo” model in both the S_3_^+^Y_*z*_ and S_3_^*n*^Y_*z*_^•^ states (Text S5 and Tables S12–S14 in the Supporting Information), and we can conclude that it does not change the basic conclusion of this study. Finally, we emphasize that the “oxo-hydroxo” model should be adopted (for cyanobacterial PSII) because of its representation of the most stable form of the ground state in the dominant population of the S_3_^+^Y_*z*_ and its derived S_3_^*n*^Y_*z*_^•^ states; for high-plants, the “oxo-hydroxo” model is also valid in the novel closed-cubane S_3_ structure according to Zahariou et al.,^68^ but the circumstances of the structural isomerization, if exist in the water-unbound form, would need further investigations.

### Comparison to Experimental Observations

Since experimental techniques probing into the S_3_ → S_4_ transition remain difficult, so far there is very limited information regarding the morphological changes of the Mn_4_CaO_5(6)_ cluster upon formation of the S_3_^*n*^Y_*z*_^•^ state. Therefore, the structural isomerization found in this study should be seen as a theoretical prediction pending experimental verification. However, some suggestive evidence still exists in support of our proposal.

Nilsson et al. discovered that substrate-water exchange is arrested in the S_3_^*n*^Y_*z*_^•^ state because of the observed dramatically slowed kinetics as compared to earlier S states.^124^ As discussed therein, the possible reasons include the impossibility to generate a Mn(III) center that is required for water exchange,^91^ H^+^ release that leads to much stronger binding of the deprotonated group, and reconstruction of the H-bonding network after proton-coupled electron transfer upon Y_*z*_ oxidation. Our proposed reversible isomerization is compatible with the observation because, in contrast to the S_3_^A,W^Y_*z*_ ⇌ S_2_Y_*z*_^•^ equilibrium that supports water exchange in the S_3_^+^Y_*z*_ state, the S_3_^A,W^Y_*z*_^•^ ⇌ S_3_^B,W^Y_*z*_^•^ equilibrium does not facilitate water exchange due to the lack of Mn(III) formation. In fact, the chemical equilibrium would cause extensive rearrangement of the locations and H-bonding orientations of water molecules and may thus even contribute to slowing down the rate of substrate water exchange in the S_3_^*n*^Y_*z*_^•^ state. Such changes in the H-bonding network have also been suggested to affect the distribution of the conformational microstates of water molecules and to thereby affect the rate of the S_3_^*n*^Y_*z*_^•^ → S_0_Y_*z*_ transition.^125^

Bao and Burnap studied the O_2_ release kinetics by site-directed mutagenesis and found that both the lag and slow phases during the S_3_^+^Y_*z*_ → S_0_^*n*^Y_*z*_ transition are retarded. On that basis, they suggested that “proton tautomerization” and/or “structural isomerization” precede(s) dioxygen formation.^93^ Specifically, they suggested two interpretations. For proton tautomerization, they proposed that it would be followed by O–O bond formation via W3–W2 nucleophilic attack,^72,81,82,85^ while in case of open-closed structural isomerization, O–O bond formation by oxo–oxyl radical coupling between W2 and O5 may occur, in line with previous suggestions.^80,83,84^ We note that O–O bond formation via water nucleophilic attack (WNA) appears less favorable on the basis of recent experiments^36,37,124^ and theoretical calculations.^71,126^ Indeed, our present results provide further support for the variant radical coupling mechanism using a closed-cubane S_4_ structure for O_2_ evolution because our theoretical finding shows that the S_4_^B,W^ structure could be obtainable via the open-to-closed rearrangement in the S_3_^*n*^Y_*z*_^•^ state (rather than in the S_3_^+^Y_*z*_ state as assumed in ref (84)).

Thus, the open-closed isomerization in the S_3_^*n*^Y_*z*_^•^ state may correspond to the proposed “structural isomerization” preceding dioxygen formation^93^ and to thereby constitute the rate limiting 1–2 ms phase (slow phase) that follows a 200 μs lag phase (Figure 1d) and precedes the much more rapid O_2_ formation.^3,12−22,92,127^ According to the Eyring–Polanyi equation of TS theory assuming a standard pre-exponential factor,^128−130^ the 1–2 ms kinetics is calculated to correspond to an activation free energy ∼14 kcal/mol at room temperature. Given the limited errors from DFT methodology and possibly experimental measurement, a safer quantity for the barrier should be around 13–15 kcal/mol for a process that occurs on a timescale of milliseconds.^71^ Siegbahn ascribed the slow phase of O_2_ formation to an intramolecular proton transfer step with 10.2 kcal/mol barrier,^52^ but as he pointed out, considering a typical accuracy within 3–5 kcal/mol, which normally overestimates barriers for a DFT hybrid functional,^51,97,98,131^ it is far below the required limit for a millisecond process.^52^ By comparison, our calculated barriers of 14.8–16.2 kcal/mol for all the possible spin states are in much better agreement with the experimental kinetics (see Text S4 in the Supporting Information for more details). If the open-to-closed transition in the S_3_^*n*^Y_*z*_^•^ state was indeed the main rate limiting step for O_2_ formation, this would mean that O_2_ formation would start exclusively from the S_3_^B,W^Y_*z*_^•^ state, and radical coupling from the S_3_^A,W^Y_*z*_^•^ state would not be possible for reasons still to be determined. Thus, at the current stage of knowledge, we do not emphasize the proposed isomerization as the only possibility taking place in S_3_^*n*^Y_*z*_^•^ → S_4_^+^Y_*z*_ before compelling experimental evidence emerges; however, the reversible open-to-closed structural rearrangement should be regarded as a viable mechanism or at least as part of processes responsible for the slow phase.

### Implications for the Mechanism of O–O Bond Formation

**Scheme 2:**
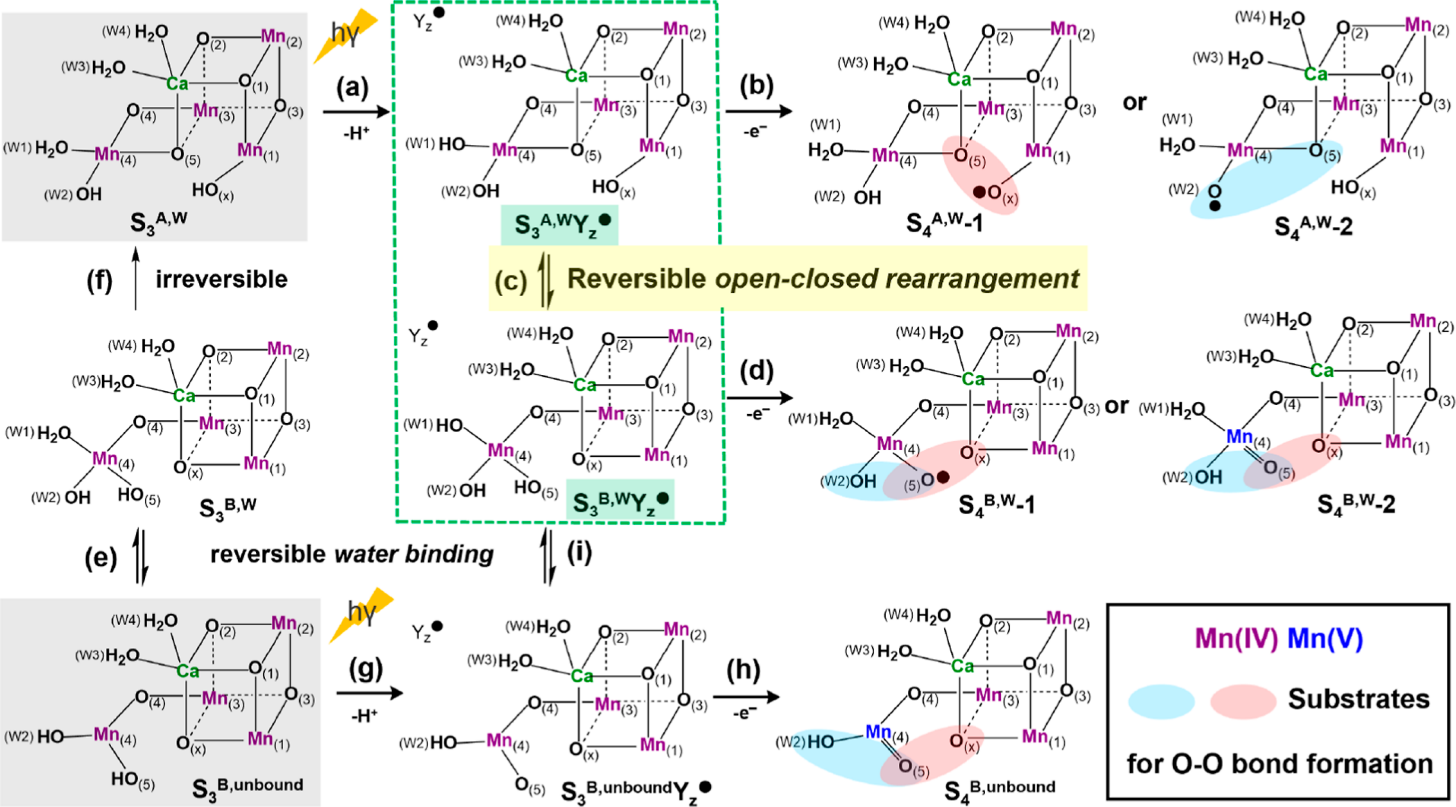
Possible mechanisms of the S_3_ → S_4_ transition and O–O Bond formation in the S_4_ State S_3_^A,W^ and S_3_^B,unbound^ in gray stand for the two potential starting configurations in the S_3_ state, resolved in cyanobacteria and higher-plant PSII by XFEL and EPR experiments, respectively.^35−38,44,68^ The process focused in this work is highlighted in the green dashed box. Candidate substrates are encircled in red (favored) or blue (possible alternatives). Mn formal oxidation states (IV)(V) are displayed in different colors. The superscript “W”/“unbound” means hexa/penta-coordinate Mn4 with a water bound/unbound water trans to O5. The annotations for sequence numbers: (a,g) Y_z_ oxidation followed by proton release; (b,d,h) intramolecular proton transfer followed by Ox/W2/O5/Mn4 oxidation; (c) reversible open-closed rearrangement in the S_3_^*n*^Y_z_^●^ state, as proposed in this study; (e,i) reversible water binding to the five-coordinate Mn4(IV) in the closed-cubane structure; and (f) irreversible closed-to-open conversion in the S_3_ state. Other proposed mechanisms are discussed in the text. It is noted that the oxygen labeling for S_3_^B,W^ and S_3_^B,unbound^ (and their derivatives) is chosen for consistency with that established by serial crystallography for the S_3_^A,W^^36,38,39^ and for convenience to describe all the transitions uniformly. These labels do not reflect the origin of the oxygens with regard to the S_1_ and S_2_ states because several options for water insertion during the S_2_ → S_3_ transition are still discussed;^6,7,32,38,43,48,52,62,82,101,120,132−139^ an alternative nomenclature based on S_3_^B,unbound^ and the pivot/carousel water insertion is shown in Figure S4 in the Supporting Information.

The proposed reversible open-closed interconversion in the S_3_^*n*^Y_*z*_^•^ state has important implications for the mechanism of O–O bond formation in the S_4_ state. This is illustrated in Scheme 2, which starts from the two structural architectures S_3_^A,W^ and S_3_^B,unbound^ observed by XFEL^35−38^ and EPR experiments,^44,68^ respectively. The first route (a) → (b) from S_3_^A,W^ to S_4_^A,W^-1 expresses Siegbahn’s oxo(O5)–oxyl(Ox) coupling mechanism that he found to be energetically most favorable.^51,53,71,76^ Here, the Mn1(IV)-bound Ox radical couples with μ-O5 in an open-cubane structure. Alternatively, the radical could be localized at W2 if it is deprotonated instead of Ox, and its coupling with μ-O5 in S_4_^A,W^-2 might be an option, which, however, has not gained support from recent DFT calculations.^79,84^

For the intermediate S_3_^A,W^Y_*z*_^•^ state prior to S_4_, our present results provide the first theoretical basis for the (reversible) conversion to S_3_^B,W^Y_*z*_^•^ by route (c), thereby diversifying alternative pathways leading to O–O bond formation in the S_4_ state with a closed-cubane type. Specifically, S_3_^B,W^Y_*z*_^•^ with the octahedral Mn4(IV) coordination may proceed to S_4_ by O5 or Mn4 oxidation through route (d), producing the O5 radical in S_4_^B,W^-1, or alternatively, Mn4(V) in S_4_^B,W^-2. Both options, W2–O5 coupling (blue) on Mn4 and O5–Ox coupling involving multiple metal (Mn and Ca) centers (red), are worth considering. It is noted that O5–Ox coupling in the S_4_^B,W^-1 state resembles the variant oxyl–oxo mechanism by Li and Siegbahn,^84^ which was based on previous experimental proposals.^4,80,124^

The S_3_^B,unbound^ state may either evolve to S_3_^A,W^ via S_3_^B,W^ after (reversible) water binding by the “pivot“ or “carousel” mechanism^43,44,120,132−134^ through the route (e) → (f) and then advance to the O_2_ formation routes described above, or, as suggested by Pantazis and collaborators, directly proceed to S_4_^B,unbound^ without water binding by the route (g) → (h).^68,69,87^ The latter pathway involves a penta-coordinate Mn4(IV) center and would lead to Mn4(V) where nucleophilic Ox-O5 coupling^68,69,87^ or hydroxo-oxo coupling between W2 and O5 might be possible. We note that the finding in the present study can provide an additional route (a) → (c) → (i) → (h) from S_3_^A,W^ to S_4_^B,unbound^ (other than from S_3_^B,unbound^).

Alternative mechanisms proposed in the literature include WNA from Ca-bound W3 onto the electron-deficient Mn4(V)=O (W2)^6,72,81,85^ and oxyl-oxo coupling between W1 and μ-oxo O4.^78^ Both appear inconsistent with mass spectrometric and EPR-based substrate water exchange data, which show that both substrates are bound to Mn(IV) in the S_3_^+^Y_*z*_ and S_3_Y_*z*_^•^ states (excluding WNA),^122,124^ and are best consistent with O5 as the slow exchanging substrate water.^4,24,80,83,122,124,140−143^ Nevertheless, these suggestions will also be further scrutinized in future DFT calculations.

Since S_3_^A,W^Y_*z*_^•^ and S_3_^B,W^Y_*z*_^•^ are nearly isoenergetic and for both states, low-energy routes for O–O bond formation via radical coupling have been determined,^51,84^ the intriguing possibility arises from the results of this study that O–O bond formation may occur via two routes, or even more, if also the S_3_^B,unbound^Y_*z*_^•^ → S_4_^B,unbound^Y_*z*_ → S_0_Y_*z*_ path in “water-deficient” catalytic sites is taken into account.^68,69^ While recent water exchange experiments in the S_2_ state have reported first evidence for two possible fast exchanging water substrates,^24,122^ the current water exchange data in the S_3_ state are best consistent with only one set of substrate waters. This would favor that either S_4_^B,W^/S_4_^B,unbound^ (substrates: W2 and O5) or S_4_^A,W^ (substrates: Ox and O5) would be involved. However, the present study suggests that the energetic and kinetic differences between these possible routes are so small that minor differences between species or experimental conditions could favor one or the other pathway.

## Conclusions

In summary, we have identified a reversible open-to-closed isomerization for the S_3_^*n*^Y_*z*_^•^ state, in contrast to the unidirectional conversion in the S_3_^+^Y_*z*_ state. This isomerization immediately before O_2_ formation is activated by deprotonation of a Mn-bound water (W1) after tyrosine Y_*z*_ oxidation. The structural rearrangement may constitute or contribute to the slow kinetic phase that prepares the Mn_4_CaO_6_ cluster for O_2_ formation. Thus, the restored structural heterogeneity prior to the S_4_ state diversifies the viable options for O–O bond formation in PSII. In this way, the availability of both open and closed-cubane structures in the S_4_ state may reflect a “two-pronged” arrangement of the OEC, allowing for efficient and robust water oxidation, and may have contributed to its evolutionary development. The elegant structural reversibility triggered by proton release in the natural enzyme may provide a useful reference for designs of artificial catalysts.

## Supporting Information Available

Model construction, spin state definition, computational details, supplementary texts, tables, figures, and references, and optimized Cartesian coordinates (PDF)
