# Supplementary material for: Reversible Structural Isomerization of Nature’s Water Oxidation Catalyst Prior to O–O Bond Formation
Source: J Am Chem Soc. 2022 Jun 24;144(26):11736–47. doi: 10.1021/jacs.2c03528 (PMC9264352; doi:10.1021/jacs.2c03528)
Supplement: Supplementary file 1 — ja2c03528_si_001.pdf [file ja2c03528_si_001.pdf]

Supporting Information

Reversible Structural Isomerization of Nature's Water Oxidation Catalyst prior to O-O Bond Formation

Yu Guo[1,2], Johannes Messinger[3,4*], Lars Kloo[5*], Licheng Sun [1,2*]

[1]*Center of Artificial Photosynthesis for Solar Fuels and Department of Chemistry, School of Science, Westlake University, Hangzhou 310024, China.*

[2]*Institute of Natural Sciences, Westlake Institute for Advanced Study, Hangzhou 310024, China.*

[3]*Department of Chemistry, Umeå University, Linnaeus väg 6 (KBC huset), SE-90187, Umeå, Sweden.*

[4]*Molecular Biomimetics, Department of Chemistry – Ångström Laboratory, Uppsala, University, SE-75120 Uppsala, Sweden.*

[5]*Department of Chemistry, School of Engineering Sciences in Chemistry, Biotechnology and Health, KTH Royal Institute of Technology, SE-10044 Stockholm, Sweden.*

***Correspondence:**

johannes.messinger@kemi.uu.se (Johannes Messinger)

larsa@kth.se (Lars Kloo)

sunlicheng@westlake.edu.cn (Licheng Sun)

Contents

I. Model construction……………………………………………S2

II. Spin state definition…………… …………………………....S3

III. Computational details……………………………….....................S3

IV. Supplementary texts……………………………….........................S5

V. Supplementary tables………………………………....................S19

VI. Supplementary figures……………………………...................S32

VII. Supplementary references…………………………..….......S34

VIII. Optimized Cartesian coordinates…………………………..S40

I. Model construction

Quantum chemical models for the initial $S_3^+Y_z$ structures were taken from the work by Cox et al. for both open and closed-cubane structures.[1] The OEC model contains the Mn_4CaO_6 core, the first-sphere ligands W1, W2, W3, W4, D1-Asp170, D1-Glu189, D1-His332, D1-Glu333, D1-Asp342, D1-Ala344, CP43-Glu354, the second-sphere ligands D1-Asp61, D1-His337, CP43-Arg357, D1-Tyr161, D1-His190, and some nearby crystal waters in hydrogen bonding interactions. Initial protonation states for all titratable groups follow their original form in supplementary reference 1, i.e., O1=O2=O3=O4=O5=O^{2-}, Ox=OH^-, W1=W3=W4=H_2O, W2=OH^-, neutral Tyr161-His190 pair and doubly protonated His337. Although the exact forms of Ox and W2 are still controversial, i.e., hydroxide (OH^-), oxo (O^{2-}), or oxyl ($O^\bullet$) for Ox[1-5] and H_2O or OH^- for W2,[6-8] the OH^- form for both Ox and W2 was preferentially selected in

this study because of its best consistency with spectroscopic and computational studies,[1, 5, 8-10] thus the 'oxo-hydroxo' model for O5-Ox in the $S_3^{A,W}$ state. However, other choices of protonation states cannot be undoubtedly excluded at this stage of knowledge and thus the effect on the isomerization in question has been also considered in this study (see Text S5 and Tables S10-S14). For the $S_3^nY_z^\bullet$ state, the oxidized Tyr161 deprotonates from its phenolic oxygen to the ε-nitrogen of His190 and one proton of W1 was manually removed from the model system. The model size is shown in Figure S1 by the $S_3^{A,W}$ state.

II. Spin state definition

All the possible spin states were considered given spin-crossover effect[11-12] induced by strong spin–orbit coupling in transition metal chemistry,[13-15] while high spin was preserved on individual Mn ions, that is, 13-et/αααα, septet/(αααβ, ααβα, αβαα, βααα) and singlet/[ααββ(=ββαα), αβαβ(=βαβα), βααβ(=αββα)] for the $S_3^+Y_z$ state; 14-et/ααααα, 12-et/ααααβ, octet/(αααβα, ααβαα, αβααα, βαααα), sextet/(αααββ, ααβαβ, αβααβ, βαααβ) and doublet/(ααββα, αβαβα, αββαα, βααβα, βαβαα, ββααα) for the $S_3^nY_z^\bullet$ state.

III. Computational details

An initial guess for each spin configuration was generated from the 'fragments' module followed by wavefunction optimization using 'stable=opt' for a stable electronic state. By importing the respective spin state, geometry optimizations with backbone constraints[16] were carried out using the unrestricted hybrid functional B3LYP* with 15% Hartree-Fock exact exchange, which shows the most reliable energetics compared with

experimental results for the OEC system (see Text S4 and Table S14 below for more details).[17] The effective core potential basis set LanL2DZ was employed for Mn and Ca, and the full electron basis set 6-31G* for the rest C, N, O, H atoms. Analytic frequency calculations on the entire models were performed at the same level as geometry optimizations to verify all the local minima. Zero-point energies and thermal effects (298.15K, 1 atm) were obtained in the form of Gibbs free energy corrections. Transition states (TS) were judged by eigenvectors required to have negative eigenvalues and the imaginary frequency vibration, followed by intrinsic reaction coordinate (IRC) analysis to confirm the connectivity of TS with reactants (Rea) and products (Pro) on the minimum energy path (MEP). On the basis of the optimized structures, more accurate single point energies were computed with a larger basis set: SDD for Mn, Ca, and cc-pvtz (*-f*) for the rest H, C, N, O atoms, added by SMD continuum solvation model[18] with the dielectric constant set to 6.0 representing the surrounding protein medium (solvent-accessible surface). Grimme's empirical formula DFT-D3 (BJ-damping) was used for dispersion correction in both geometry optimizations and single point energies. Besides, relaxed potential energy surface (PES) scans for proton transfer were performed on the level of geometry optimization, using the step size of 0.01 Å. All the DFT calculations were executed by Gaussian 16 (version C. 01).[19] Multiwfn (version 3.8)[20] was used to calculate Wiberg bond orders, and verify the formal oxidation states of Mn by localized orbital bonding analysis (LOBA),[21] in addition to Mulliken spin populations shown by Gaussian 16.

For an unimolecular reaction, as the isomerization studied here, the rate constant

according to the Eyring-Polanyi equation of transition state theory (TST)[22-24] can be shown as (ignoring the tunneling effect and assuming the impact of rotational symmetry number σ has been reflected in $\Delta G^{\neq}$): $k = 2.1*10^{10} T\, e^{-1000\Delta G^{\neq}/(1.9859*T)}$ where temperature T is in K, free barrier $\Delta G^{\neq}$ is in kcal/mol. Normally the '97% completion' can be recognized as the time for the reaction duration, which is calculated by $t = -(ln0.03)/k$. Besides, the equilibrium constant for a reversible chemical reaction is expressed as $K_{eq} = \exp(-\Delta G^{o}/RT)$.

IV. Supplementary texts

Text S1. Analysis for the scan curve for the conversion between $S_3^{A,W}Y_z$(W1=H_2O) and $S_3^{B,W}Y_z$(W1=H_2O)

The scan curve is analyzed as follows: the Ox-protonated $S_3^{A,W}$-OxH state is clearly a stationary point characterized by frequency analysis and the RMS gradient norm shown here. However, when proton approaches O5 gradually by the scan process, the energy is always rising up and no stationary point can be found for the O5-protonated $S_3^{A,W}$-O5H state because the RMS gradient norm is never close enough to zero. Any tentative optimization starting from the $S_3^{A,W}$-O5H state would automatically decaying back to the $S_3^{A,W}$-OxH state. These strongly indicate protonation on O5 is impossible and the similar interpretation will be used hereafter.

Text S2. Analysis for the spin configurations for the conversion between $S_3^{A,W}Y_z$(W1=H_2O) and $S_3^{B,W}Y_z$(W1=H_2O)

The minor spin changes on Mn1 and Ox before TS results from the deprotonation process from Ox to O5, which improves its covalency to Mn1. The slight spins (< 0.4)

appearing on Ox comes from partial electron donation from Mn1, which leads to a minimum spin ca. 2.6 on the MEP. However, a magnitude less than 0.4 spins should assign Ox as oxo rather than oxyl,[25] and Mn1 is still in formal oxidation state IV. After TS, Ox becomes a μ-oxo between Mn1 and Mn3 while O5 is protonated as a terminal ligand of Mn4, so that spins on Ox and O5 are nearly zero.

Text S3. Reflections on the component proportion in the $S_3^nY_z^\bullet$ state

According to the basic formula $\Delta G° = -RT \ln K_{eq}$ (K=[B]/[A]) in chemical equilibrium A⇌B, one can calculate the ratio of $[S_3^{B,W}Y_z^\bullet]/[S_3^{A,W}Y_z^\bullet]$ or the percentage of $S_3^{A,W}Y_z^\bullet$ and $S_3^{B,W}Y_z^\bullet$ in an equilibrium state. According to the results above, $S_3^{A,W}Y_z^\bullet$ would be present to more than 98%, 11%-90%, or more than 97% when 2.4～3.9 kcal/mol (B3LYP*-D3BJ, W2=OH^-), -1.1～1.3 kcal/mol (B3LYP*-D3BJ, W2=H_2O) or 2.1～3.5 kcal/mol (B3LYP-D3BJ, W2=OH^-) is respectively used for $\Delta G°$ at room temperature. However, it should be emphasized that an accurate prediction of the population is impossible based on the current DFT results and the obtained percentage is meaningless. Consequently, one should not ignore the following points that would fundamentally change the predominant form of the $S_3^nY_z^\bullet$ state and cannot overlook the importance of either component.

The formula $\Delta G° = -RT \ln K_{eq}$ (K=[B]/[A]) is usually based on accurate measurements of constituent concentrations in experiments, such as those by Messinger and coworkers on the driving force for the $S_3 \rightarrow S_0$ transition.[26] However, when one aims to use a computational DFT approach to instead calculate the constituent concentrations (the percentage/population of $S_3^{A,W}Y_z^\bullet$ or $S_3^{B,W}Y_z^\bullet$, as discussed here), one must bear

in mind that the ratio [$S_3^{B,W}Y_z$•]/[$S_3^{A,W}Y_z$•] is extremely sensitive to the value of ΔG°, and only a slight variation of ΔG° in kcal/mol will lead to a large (in fact, exponential) change of the relative percentage of A or B. For example, a slight decrease of ΔG° (of a reaction in chemical equilibrium) from 0.0 to -0.1, -0.5, -1.0, -2.0 would lead to the ratio of [$S_3^{B,W}Y_z$•]/[$S_3^{A,W}Y_z$•] to change from 1.0 to 1.2, 2.3, 5.4, 29.3, respectively; a slight increase from 0.0 to 0.1, 0.5, 1.0, 2.0 would lead to a ratio of [$S_3^{B,W}Y_z$•]/[$S_3^{A,W}Y_z$•] from 1.0 to 0.84, 0.43, 0.18, 0.03, respectively. Furthermore, as mentioned above, a few kcal/mol error is expected and unavoidable and acceptable for DFT results, and therefore the ratio of [$S_3^{B,W}Y_z$•]/[$S_3^{A,W}Y_z$•] or percentage/population of $S_3^{A,W}Y_z$• or $S_3^{B,W}Y_z$• estimated from ΔG from DFT results cannot be invoked as a strong argument to identify the predominant substance in a chemical equilibrium. In addition to the intrinsic limitations in the accuracy of DFT methodology, different choices of models and computational details, as well as the kinds of approximations in treating quantum chemical models may also bring a small variance in calculated energetics but would give rise to a large uncertainty in any estimate of the [$S_3^{B,W}Y_z$•]/[$S_3^{A,W}Y_z$•] ratio.

The above demonstration is reflected in the literatures with respect to the well-known O5-shifting isomerization in both the S_2 and the S_2Y_z• states. As computed by Pantazis et al., the relative energy between the S_2^A and S_2^B models is on the order of 1 kcal/mol, but comes out exactly as 1.92 kcal/mol by B3LYP and 0.42 kcal/mol by TPSSH-D3 functionals;[27] this corresponds to 0.04 and 0.71, respectively, for the ratio [S_2-B]/[S_2-A]; Bovi et al.[28] obtained ΔG=1.1 kcal/mol by ab initio molecular dynamics simulations corresponding to [S_2^B]/[S_2^A]=0.16. Besides, Siegbahn,[29] Ugur et al.,[30] Boussac et al.,[31]

Vinyard et al.,[32] Isobe et al.,[33] Saitow et al.[34] reported 1.1, 1.1, 1.1, 0.8, 1.3, 2.0 kcal/mol for ΔG. These results correspond to the smallest $[S_2^B]/[S_2^A]$=0.03. For the $S_2Y_z^\bullet$ state, the relative stability is even reversed as calculated by Pantazis and coworkers and Guidoni and coworkers: the former got 1.3 kcal/mol higher in energy of $S_2^BY_z^\bullet$ than $S_2^AY_z^\bullet$[35] while the latter instead reported 2.6 kcal/mol lower energy of $S_2^BY_z^\bullet$ than $S_2^AY_z^\bullet$,[36] which indicates as much as 3.9 kcal/mol difference and ranging the ratio of $[S_2^BY_z^\bullet]/[S_2^AY_z^\bullet]$ from 0.11 to 80.7. Clearly, despite the minor thermodynamical difference in a few kcal/mol, the dominance of $S_2^BY_z^\bullet$ or $S_2^AY_z^\bullet$ in the $S_2Y_z^\bullet$ state is uncertain, but both open and closed forms are deemed important in the water oxidation mechanism.

For the recently proposed isomerism in the S_1 state by Pantazis and coworkers,[37] the authors pointed out that "using the BP86 optimized geometries in the respective spin configurations, the most stable of the three isomers discussed in the present work is S_1^A, followed by S_1^B (3.2 kcal/mol) and S_1^C (5.3 kcal/mol). Similar energy differences are obtained using different functionals with the same BP86 geometries. It is noted that with some functionals the relative stability of S_1^A and S_1^B may be reversed, but S_1^C remains higher than both, in accordance with the description of the Jahn–Teller potential energy surface discussed in this work. For example, using the popular B3LYP* functional (15% exact exchange) S_1^A is 1.7 kcal/mol and S_1^C is 3.1 kcal/mol higher than S_1^B. Using a larger, more polarized basis set (the def2 versions of the ZORA all-electron basis sets) the differences are negligible (with B3LYP*, S_1^A is 2.0 kcal/mol and S_1^C is 3.4 kcal/mol higher than S_1^B). Such small energy differences are well within

the intrinsic uncertainty of the method and the uncertainty related to the structural definition of the models." Consequently, one should not overlook the consequences of small errors in the Gibbs free energy differences and overstated the relative energy while making an unnecessary connection to the substance population/percentage.

Another important nature for the isomerization in chemical equilibrium in the $S_3{}^nY_z{}^\bullet$ state is the dynamic factor, i.e., shift of an equilibrium that affects the component proportion. The ultimate reaction direction will shift depending on the consumption of $S_3{}^{A,W}Y_z{}^\bullet$ and $S_3{}^{B,W}Y_z{}^\bullet$ to $S_4{}^{A,W}$ and $S_4{}^{B,W}$, which will considerably affect the relative concentration/population of $S_3{}^{A,W}Y_z{}^\bullet$ and $S_3{}^{B,W}Y_z{}^\bullet$. The concept of dynamic equilibrium is well known in chemistry and for instance used in various process, such as chiral separation. The water oxidation is a successive and cyclic reaction, in which the $S_3{}^nY_z{}^\bullet$ state in question is not the end and it is just a transient state prior to the S_4 state where O-O bond formation takes place. Once the $S_3{}^nY_z{}^\bullet$ state is reached and the chemical equilibrium forms in terms of isomerization (reversible $S_3{}^{A,W}Y_z{}^\bullet$ to $S_3{}^{B,W}Y_z{}^\bullet$, as proposed in this study), it will rapidly enter the S_4 state (triggered by $Y_z{}^\bullet$ oxidizing the $Mn_4CaO_{5(6)}$ cluster). According to the present study, both $S_3{}^{A,W}Y_z{}^\bullet$ and $S_3{}^{B,W}Y_z{}^\bullet$ could evolve to the S_4 state as $S_4{}^{A,W}$ and $S_4{}^{B,W}$, and also both the open and closed forms of S_4 have been proposed in the literatures.[38-43] It is unknown which component is consumed faster because the relative thermodynamic stability between $S_4{}^{A,W}$ and $S_4{}^{B,W}$ and the kinetics from $S_3{}^{A,W}Y_z{}^\bullet$ to $S_4{}^{A,W}$ and $S_3{}^{B,W}Y_z{}^\bullet$ to $S_4{}^{B,W}$ are still inconclusive. Regarding this, Siegbahn has found O-O bond formation are feasible for both $S_4{}^{A,W}$ and $S_4{}^{B,W}$, with rather similar barriers counting from the optimal S_3 state.[41, 44] Therefore, at

present, it is still impossible to predict the orientation change of the dynamic equilibrium and the dominant substance in the previous $S_3^nY_z^\bullet$ state as either $S_3^{A,W}Y_z^\bullet$ or $S_3^{B,W}Y_z^\bullet$.

It is known that the barriers $\Delta G^{\neq}$ (difference between the TS and reactants) derived from DFT computations may have an error within 3-5 kcal/mol normally overestimating barriers,[45-48] it is not unlikely that similar situation applies to ΔG (standard Gibbs free energy difference between products and reactants), although not well established in literatures. If this were the case, $S_3^{A,W}Y_z^\bullet$ and $S_3^{B,W}Y_z^\bullet$ might be exactly isoenergetic. To say the least, think of S_1^A/S_1^B with 3.2 kcal/mol difference (S_1^A more stable) and S_2^A/S_2^B with 1-2 kcal/mol difference (S_2^A more stable), and the importance of S_1^B and S_2^B for their relevance with EPR signals and the proposed mechanistic roles in the S-state cycle.[27-28, 36, 39, 49-50] Similarly, one should not look down upon the importance of $S_3^{B,W}Y_z^\bullet$ in the $S_3^{A,W}Y_z^\bullet \rightleftharpoons S_3^{B,W}Y_z^\bullet$ equilibrium with 2.4-3.9 kcal/mol energy difference even if the energy gap were regarded accurate.

Text S4. Error analysis for the calculated barriers related to experimental kinetics

Unlike O-O bond formation in the S_4 state, the isomerization between $S_3^{A,W}Y_z^\bullet$ and $S_3^{B,W}Y_z^\bullet$ is spin-insensitive because it does not involve any ligand-metal single electron transfer (SET) that can change the formal oxidation states of the metal centers, meaning that ferromagnetic or antiferromagnetic coupling between metals does not affect the energetics, as shown in above calculations for all the possible spin states. The relative free energy ranges within '2.0 (0.0～2.0) kcal/mol' in $S_3^{A,W}Y_z^\bullet$, '2.3 (15.5～17.8) kcal/mol' in TS, and '2.7 (2.9～5.6) kcal/mol' in $S_3^{B,W}Y_z^\bullet$ are sufficiently small to

account for the aforementioned spin-insensitivity, and it is not clear why it is necessary and how to simply select a 'most stable' reactant or a 'most favorable' spin channel while discriminating other alternatives, given the close energies and unavoidable errors in the DFT methodology.[45, 51-52] Consequently, each individual spin state should represent a possible channel of isomerization, which is the reason why we addressed $\Delta G^{\neq}(A \rightarrow B)$ and $\Delta G(A \rightarrow B)$ from their respective spin states, instead of from the octet/ααβαα spin state with '0.0 kcal/mol' $S_3^{A,W}Y_z^{\bullet}$ and in this process obtained '14.8～16.2 kcal/mol' barriers. According to the Eyring-Polanyi equation for the transition state theory (TST),[22-24] the experimental timescale of '1～2 ms' corresponds to a barrier ～14 kcal/mol. Considering the '3～5 kcal/mol' error bar of a hybrid DFT functional normally overestimating barriers,[45-47, 53] the obtained 14.8～16.2 kcal/mol are fully compatible with the required ～14 kcal/mol even if one would use the calculated upper limit 16.2 kcal/mol. In fact, one should consider the range of the difference 1.8～3.6 kcal/mol, noting that such an energy difference is even smaller than the strength of a weak O-H hydrogen bond (4～8 kcal/mol).[54] The main reason we pointed out that spin-crossing is quite feasible due to large spin-orbit coupling is to indicate that each spin state is easily reachable and could be potentially involved in the isomerization process. If the calculated energy differences were sufficiently accurate to discriminate between the different spin states, we would have been able to show that $S_3^{A,W}Y_z^{\bullet}$ in the octet/ααβαα spin state would be mostly populated. However, one must bear in mind that the start-of-art DFT models do not allow such an arbitrary judgment based on merely a few kcal/mol difference, since there are many fundamental reasons originating

from the basic approximations employed in DFT.[45, 51-53, 55] Furthermore, different protonation state distributions and choices of methods can also affect the identification of the 'most stable' spin state (see below). Therefore, identifying a 'most stable' spin state with the lowest energy with only minor difference is not convincing at the current stage, and instead, identification of an energy range after considering all the possible spin states represents a more prudent for a quantitative estimate of the energies involved.

In another perspective, if one counts the barrier from the 'superficially' most stable spin state, in the meanwhile, one needs to regard the calculated relative energies to be sufficiently accurate and also distinguishable to allow a selection of the most feasible spin channel through which the reaction occurs, and only the lowest barrier should be adequate for comparison with the experimental results. Taking such a strategy, the route from $S_3^{A,W}Y_z^{\bullet}$ in the octet/ααβαα spin state (0.0 kcal/mol) onto TS in octet/αααβα (15.5 kcal/mol) and then to $S_3^{B,W}Y_z^{\bullet}$ in octet/αααβα (2.9 kcal/mol) can be identified as the lowest energy channel, where the barrier '15.5 kcal/mol' also corresponds well to the experimental results given the DFT error bar within 3～5 kcal/mol.[45-47, 53] However, we do not suggest selecting a precise value for the barrier among the 16 possible spin states because of the close energies and inherent (and unavoidable) errors associated by the current DFT methodology. Regardless of employing a barrier as '14.8～16.2' or '15.5' kcal/mol, both alternatives after error corrections are consistent with '1 ～ 2' ms timescale. It is noted that although we made a plausible connection for the isomerization to the $S_3^{n}Y_z^{\bullet} \rightarrow S_4^{+}Y_z$ transition, for strictness, we emphasize it is a 'possible' mechanism and maybe part of the reactions during this period.

Text S5. Isomerization with alternative protonation states

Up to now, there are no experimental techniques that can directly and unambiguously determine the protonation states of the OEC cluster (nor many other metalloenzyme systems) in any of the (meta)stable states, even when considering techniques such as XFEL, EPR, NMR, XAS, XANES, etc. If there were, many related mechanistic issues during the catalytic cycle would have been already settled. However, before the (possible) development of such a powerful experimental technique in the future (e.g. neutron diffraction at high-enough resolution), researchers in this and related fields are restrained to theoretical modeling based on existing experimental knowledges. The $S_3^nY_z^\bullet \rightarrow S_4^+Y_z$ period in the OEC catalytic cycle is even more difficult to characterize by experiments, and computational chemists are confined to select a realistic protonation state that is the most reliable when constructing a model according to current literature results involving suggested protonation states. In fact, all the computational works on the OEC (except those specially aiming for the protonation states) can be regarded as of this type. In our study, we have chosen to use the protonation states of the $S_3^+Y_z$ state suggested by Cox et al.,[1] which reproduced the experimental EPR and ^{55}Mn-EDNMR spectra of the $S_3^+Y_z$ state, and in good agreement with most computational studies,[10, 46, 48, 56-59] and in line with an illustration on water oxidation mechanism in a very recent experimental work on substrate water exchange.[60] For the $S_3^nY_z^\bullet$ state, W1 deprotonation has been widely acknowledged,[38-39, 57, 61-66] so that W1 was described as a hydroxide ligand (after H_2O deprotonation) in the $S_3^nY_z^\bullet$ state of our model. We are of course aware that future studies specially aiming at the

protonation states might lead to new results, but the above noted literatures have clearly shown that the protonation states in our study can be regarded as the current state of knowledge and thus can be regarded as reliable models for the study of OEC structural isomerization.

We can notice that besides the consensus on the protonation states for the titrable groups in the $S_3^+Y_z$ model (already adopted in our models), i.e., W1, W3, W4=H_2O, O1, O2, O3, O4, O5=oxo, all unprotonated carboxyl ligands, protonated Arg357 and doubly protonated His337, the remaining controversies about the protonation states in the $S_3^+Y_z$ state come from W2=OH^- or H_2O,[6-8, 10, 67-68] and Ox=OH^- or oxo or oxyl,[1-5] although the OH^- forms for both appear to be more favored as we initially adopted. The protonation state itself is not the theme of this work, but we have performed extensive additional computations and found that our conclusion still holds even if different protonation states of W2 and Ox were considered, as discussed below. Five cases (a-e) of the potential protonation states regarding W2 and Ox can be identified for S_3Y_z and $S_3^nY_z^\bullet$ (the consequences of the above situations are summarized in Table S14):

a. (i) $S_3^+Y_z$ (W1=H_2O, W2=OH^-, Ox=OH^-) (Table S1), (ii) $S_3^nY_z^\bullet$ (W1=OH^-, W2=OH^-, Ox=OH^-) (Table S2), preferred protonation states, shown in the main text;
b. (i) $S_3^+Y_z$ (W1=H_2O, W2=H_2O, Ox=OH^-) (Table S10), (ii) $S_3^nY_z^\bullet$ (W1=OH^-, W2=H_2O, Ox=OH^-) (Table S11);
c. (i) $S_3^+Y_z$ (W1=H_2O, W2=OH^-, Ox=oxo) (Table S12), (ii) $S_3^nY_z^\bullet$ (W1=OH^-,

W2=OH^{-}, Ox=oxo);

d. (i) $S_3^{+}Y_z$ (W1=H_2O, W2=H_2O, Ox=oxo) (Table S13), (ii) $S_3^{n}Y_z^{\bullet}$ (W1=OH^{-}, W2=H_2O, Ox=oxo);

e. (i) $S_3^{+}Y_z$ (W1=H_2O, W2=OH^{-}/H_2O, Ox=oxyl), (ii) $S_3^{n}Y_z^{\bullet}$ (W1= OH^{-}, W2=OH^{-}/H_2O, Ox=oxyl).

Comparing the free energy landscapes of a and b, W2=OH^{-} or H_2O does not differ much regarding the isomerization energetics for both $S_3^{+}Y_z$ [(a, i) versus (b, i)] and $S_3^{n}Y_z^{\bullet}$ [(a, ii) versus (b, ii)] when Ox=OH^{-}. For the $S_3^{n}Y_z^{\bullet}$ isomerization with W2=H_2O (b, ii), the free energy of $S_3^{B,W}Y_z^{\bullet}$ could be even slightly lower than that of $S_3^{A,W}Y_z^{\bullet}$ by 0.2-1.1 kcal/mol for a few spin states, however, following the discussion above, these energy differences are too small to be considered of significance.

When Ox=oxo is involved in $S_3^{+}Y_z$, the isomerization energetics of (c, i) resembles the situations in (a, i) and (b, i). The proton states of (d, i) can be energetically compared with (a, i), only differing in one proton located at W2 or Ox. It is seen that $S_3^{A,W}Y_z$ (W1=H_2O, W2=H_2O, Ox=oxo) of (d, i) is about 10 kcal/mol higher in free energy in each attainable spin state than that of (a, i). This indicates that (d, i) is less likely to be a model representing a stable protonation form. Consequently, the isomerization pathway in the (d, i) state and the subsequent tyrosine-oxidized state (d, ii) are therefore not investigated further.

It is found that the protonation state (c, ii) violates the electrostatic rule that leads to the deprotonated W1 from its preceding state (c, i).[69-72] The driving force to expel a proton from W1 to the lumen (via Asp61) can be explained as the charge-compensating

effect of the Mn_4CaO_6 cluster from an electrostatically induced pK_a shift of W1 (H_2O). After Y_z is oxidized to $Y_z^•$ going from $S_3^+Y_z$ to $S_3^nY_z^•$, the phenolic proton of $Y_z^•$ transfers to His190 as a result of proton-coupled electron transfer (PCET). As His190 links to the Mn_4CaO_6 cluster via Glu189, the net charge of the Mn_4CaO_6 cluster increases from zero [when (W2=OH^- and Ox=OH^-) or (W2=H_2O and Ox=oxo)] to +1 because of the extra proton, causing one H^+ dissociates from W1 (onto the Asp61 and then the lumen) due to its lower pK_a and the negatively charged electronic attraction from Asp61. However, when W2=OH^- and Ox=oxo (with one positive charge less) in the $S_3^nY_z^•$ state, the electrostatic driving force to expel H^+ from W1 is substantially weakened, and the state after H^+-release to the lumen would give rise to both negatively charged Mn_4CaO_6 cluster and Asp61 causing mutual electrostatic repulsion. These consequences demonstrate that the proton distribution of W2=OH^- and Ox=oxo in the $S_3^nY_z^•$ state is less likely and an investigation into the details of an isomerization pathway for the scenario is unnecessary. Besides, as shown by our attempts, any tentative optimization of the (c, ii) state would lead to reduction of $Y_z^•$ and oxidation of Ox, i.e. forming S_4 instead of $S_3^nY_z^•$, which also implies an inherent instability of the $S_3^nY_z^•$ state with such a proton distribution.

The oxyl form of Ox [(e, i) and (e, ii)] would cause Mn4 reduction to valence (III) in the $S_3^+Y_z$ state which is inconsistent with spectroscopic observations[1] and would destabilize W1=OH^- coordination to Mn4 in the $S_3^nY_z^•$ state, thus it was not considered for the isomerization.

Text S6. Analysis on the current models accounting for the high-spin S_2 state

The research community has almost reached a consensus on the low-spin (S=1/2) configuration of the S_2 state, in which the Mn formal oxidation states are Mn1(III)Mn2(IV)Mn3(IV)Mn4(IV).[10, 27, 73-77] However, to date there are three theoretical models accounting for the high-spin (S=5/2) S_2 state: (1) closed-cubane structure by μ-O5 shift;[27-28] (2) OH^- binding to Mn1 (open-cubane);[29, 78] (3) μ-O4 protonation (open-cubane).[79] All these models can be suggested based on plausible arguments and therefore qualify as likely candidates.

As pointed by Drosou et al. (in the SI),[37] these different models may be rationalized under different conditions/treatments using different scenarios, so there is no compelling reason to immediately exclude any of the possible models before solid experimental evidence emerge. However, it should also be acceptable that a specific model is selected for model studies. Below some points are outlined for the reason why model (1) should be considered more plausible. Firstly, the closed-cubane structure is in good accordance with spectroscopic results, such as intermetallic distances obtained from EXAFS, as well as exchange coupling constants J_{ij} and isotropic ^{55}Mn hyperfine constants (HFCs).[27] Secondly, EPR studies by Mino and coworkers have shown that Mn(III) of the high-spin S_2 state is localized at the dangler position of the cluster,[80-81] which imposes the valence distribution Mn1(IV)Mn2(IV)Mn3(IV)Mn4(III) on the high-spin S_2 configuration. Based on this, models (1) and (2) are consistent because valence rearrangement is accompanied, while model (3) does not. Thirdly, the low and high-spin forms should be almost iso-energetic because they can coexist at very low temperatures,[31, 82-84] and an experimental estimation of the energy difference only

amounts to (0.7 ± 0.1) kcal/mol.[32] Only model (1) provides a computed energy difference of similar magnitude, 1-2 kcal/mol,[27-28, 30-31, 33-34, 36, 85] while model (2) renders 7.1 kcal/mol[79] and model (3) ca. 10 kcal/mol.[29] Furthermore, model (2) enquires multistep process to reach the required valence distribution and no isomers are included, although in part favored by water exchange in the S_2 state.[86] In summary, only model (1) is eligible by agreeable Mn(III) location in coherence with EPR results and favorable computed energetics for the high-spin S_2 state, and consequently it should be considered as the most appropriate model to be used, based on the current experimental foundation. The open-closed S_2 isomers have been widely used for further studies, for example, in a very recent study by Kaila and coworkers, who concluded that both the structural isomers could play a role in the catalytic cycle.[87]

A comparative study on the structural isomerization of the cluster between the $S_3^+Y_z$ and $S_3^nY_z^\bullet$ states is actually independent of the chemical nature of the high-spin form in the S_2 state, which is still under debate as mentioned earlier. However, the validity of the open-closed S_2 isomers would be relevant to the mechanistic progression of the $S_2 \rightarrow S_3$ transition. Although not affecting the obtained results and beyond the scope of this study, an objective analysis of current plausible models is presented above, and the rational analysis provides strong support for the structural isomerization shown in Figure 1e, which represents the background of this article.

Text S7. Effect of dispersion parameters

Since there are no DFT-D3(BJ) dispersion parameters specific for the hybrid functional B3LYP*, the dispersion parameters developed for B3LYP were used instead. However,

this 'mismatch' is not a mistake; instead as the reasons below this is an established approach. Firstly, As Siegbahn pointed out, the usage of B3LYP* instead of B3LYP shows the best agreement with experiments,[17] and it was also highlighted that significant van der Waals effects (dispersion effects) are essential for transition metal complexes such as the OEC and that Grimme's empirical formula is recommended.[88] Therefore, they are both important and B3LYP*-D (D3-BJ latest and available in Gaussian 16) is such a combination. Secondly, B3LYP* includes only '5%' HF exact exchange less than B3LYP, and such a minor difference is expected to lead to little effect on the results; Thirdly, the combination of B3LYP* and Grimme's dispersion corrections has been widely used in literatures. For instance, Siegbahn has consistently used B3LYP*-D2 on the OEC systems although there are also no D2 dispersion parameters specifically developed for B3LYP*.[9, 16, 29, 41, 44, 46-48, 55, 65, 89-97] A very recent study by Kaila and coworkers performed B3LYP*-D3.[87] The results in these studies indicate that the use of the B3LYP*-D combination represents a robust approach. Still, we performed a test for the $S_3{}^nY_z{}^\bullet$ state using B3LYP-D3(BJ) and found the free energy landscape is quite similar with the situation obtained by using B3LYP*-D3(BJ), as shown in Table S15.

V. Supplementary tables

Table S1. Gibbs free energies of $S_3{}^{A,W}Y_z(W1{=}H_2O)$, TS and $S_3{}^{B,W}Y_z(W1{=}H_2O)$ for all the possible spin states for A-B conversion when W2=OH⁻, Ox=OH⁻ in the $S_3{}^+Y_z$ state.

A-B / Spin states		Gibbs free energy in a.u. (Relative free energy in kcal/mol)		
		**$S_3{}^{A,W}Y_z(W1{=}H_2O)$**	**TS**	**$S_3{}^{B,W}Y_z(W1{=}H_2O)$**
**13-et**	**αααα**	-6528.0664 (0.1)	-6528.0272 (24.7)	-6528.0451 (13.5)

**Septet**	**αααβ**	-6528.0666 (0.0)	-6528.0278 (24.3)	-6528.0449 (13.6)
	**ααβα**	-6528.0666 (0.0)	-6528.0266 (25.1)	-6528.0431 (14.7)
	**αβαα**	-6528.0650 (1.0)	-6528.0247 (26.3)	-6528.0430 (14.8)
	**βααα**	-6528.0657 (0.6)	-6528.0255 (25.8)	-6528.0427 (15.0)
**Singlet**	**ααββ**	-6528.0637 (1.8)	-6528.0230 (27.4)	-6528.0406 (16.3)
	**αβαβ**	-6528.0640 (1.6)	-6528.0244 (26.5)	-6528.0416 (15.7)
	**βααβ**	-6528.0649 (1.1)	-6528.0250 (26.1)	-6528.0413 (15.9)

Table S2. Mulliken spin populations of the key atoms in $S_3^{A,W}Y_z(W1=H_2O)$, TS and $S_3^{B,W}Y_z(W1=H_2O)$ for all the possible spin states for A-B conversion when W2=OH⁻, Ox=OH⁻ in the $S_3^{+}Y_z$ state.

**A-B** / **Spin states**			**Mulliken spin population**		
			**$S_3^{A,W}Y_z(W1=H_2O)$**	**TS**	**$S_3^{B,W}Y_z(W1=H_2O)$**
**13-et**	**α**	**Mn1**	2.95	2.85	2.98
	**α**	**Mn2**	3.01	2.98	2.95
	**α**	**Mn3**	2.86	3.04	2.87
	**α**	**Mn4**	2.98	2.96	2.93
		**O5**	0.05	0.00	0.04
		**Ox**	0.07	0.16	-0.01
**Septet**	**α**	**Mn1**	2.95	2.84	2.97
	**α**	**Mn2**	3.01	2.98	2.94
	**α**	**Mn3**	2.87	3.01	2.85
	**β**	**Mn4**	-2.90	-2.90	-2.86
		**O5**	0.00	-0.01	-0.06
		**Ox**	0.07	0.17	-0.01
	**α**	**Mn1**	2.95	2.83	2.94
	**α**	**Mn2**	2.98	2.97	2.97
	**β**	**Mn3**	-2.89	-2.92	-2.79

	α	Mn4	2.91	2.90	2.87
		O5	0.02	0.01	0.05
		Ox	0.06	0.15	0.04
	α	Mn1	2.93	2.86	3.00
	β	Mn2	-2.96	-2.95	-2.96
	α	Mn3	2.85	3.02	2.85
	α	Mn4	2.98	2.95	2.93
		O5	0.04	0.00	0.04
		Ox	0.06	0.15	-0.03
	β	Mn1	-2.93	-2.83	-2.94
	α	Mn2	2.99	2.97	2.95
	α	Mn3	2.88	2.98	2.85
	α	Mn4	2.97	2.95	2.93
		O5	0.03	0.01	0.06
		Ox	-0.06	-0.16	-0.03
Singlet	α	Mn1	2.94	2.82	2.93
	α	Mn2	2.98	2.97	2.97
	β	Mn3	-2.88	-2.95	-2.81
	β	Mn4	-2.97	-2.95	-2.93
		O5	-0.03	-0.01	-0.06
		Ox	0.07	0.16	0.04
	α	Mn1	2.94	2.85	2.99
	β	Mn2	-2.96	-2.95	-2.96
	α	Mn3	2.86	2.98	2.83
	β	Mn4	-2.90	-2.90	-2.87

		**O5**	-0.01	-0.02	-0.06
		**Ox**	0.06	0.16	-0.03
	**β**	**Mn1**	-2.93	-2.84	-2.94
	**α**	**Mn2**	2.99	2.96	2.94
	**α**	**Mn3**	2.89	2.96	2.83
	**β**	**Mn4**	-2.91	-2.90	-2.87
		**O5**	-0.01	-0.01	-0.05
		**Ox**	-0.06	-0.15	-0.03

Table S3. Gibbs free energies of $S_3^{A,W}Y_z^{\bullet}$(W1=OH⁻), TS and $S_3^{B,W}Y_z^{\bullet}$(W1=OH⁻) for all the possible spin states for A-B conversion when W2=OH⁻, Ox=OH⁻in the $S_3^{n}Y_z^{\bullet}$ state.

**A-B / Spin states**		**Gibbs free energy in a.u. (Relative free energy in kcal/mol)**		
		**$S_3^{A,W}Y_z^{\bullet}$(W1=OH⁻)**	**TS**	**$S_3^{B,W}Y_z^{\bullet}$(W1=OH⁻)**
**14-et**	**ααααα**	-6527.3993 (0.9)	-6527.3757 (15.8)	-6527.3954 (3.4)
**12-et**	**ααααβ**	-6527.3991 (1.1)	-6527.3754 (15.9)	-6527.3952 (3.5)
**Octet**	**αααβα**	-6527.4000 (0.5)	-6527.3761 (15.5)	-6527.3961 (2.9)
	**ααβαα**	-6527.4008 (0.0)	-6527.3750 (16.2)	-6527.3946 (3.9)
	**αβααα**	-6527.3979 (1.8)	-6527.3737 (17.0)	-6527.3937 (4.5)
	**βαααα**	-6527.3991 (1.1)	-6527.3739 (16.9)	-6527.3932 (4.8)
**Sextet**	**αααββ**	-6527.3998 (0.6)	-6527.3758 (15.7)	-6527.3958 (3.1)
	**ααβαβ**	-6527.4005 (0.2)	-6527.3748 (16.3)	-6527.3943 (4.1)
	**αβααβ**	-6527.3976 (2.0)	-6527.3734 (17.2)	-6527.3935 (4.6)
	**βαααβ**	-6527.3988 (1.3)	-6527.3736 (17.1)	-6527.3930 (4.9)
**Doublet**	**ααββα**	-6527.3979 (1.8)	-6527.3724 (17.8)	-6527.3918 (5.6)
	**αβαβα**	-6527.3981 (1.7)	-6527.3736 (17.1)	-6527.3937 (4.5)
	**αββαα**	-6527.3992 (1.0)	-6527.3736 (17.1)	-6527.3933 (4.7)
	**βααβα**	-6527.3993 (0.9)	-6527.3736 (17.1)	-6527.3933 (4.7)
	**βαβαα**	-6527.3981 (1.7)	-6527.3736 (17.1)	-6527.3938 (4.4)

	ββααα	-6527.3979 (1.8)	-6527.3724 (17.8)	-6527.3918 (5.6)

Table S4. Mulliken spin populations of the key atoms in $S_3^{A,W}Y_z$•(W1=OH⁻), TS and $S_3^{B,W}Y_z$•(W1=OH⁻) for all the possible spin states for A-B conversion when W2=OH⁻, Ox=OH⁻ in the $S_3^nY_z$• state.

A-B / Spin states			Mulliken spin population		
			**$S_3^{A,W}Y_z$•(W1=OH⁻)**	**TS**	**$S_3^{B,W}Y_z$•(W1=OH⁻)**
**14-et**	**α**	**Mn1**	2.69	2.86	3.00
	**α**	**Mn2**	2.99	2.98	2.94
	**α**	**Mn3**	2.89	3.06	2.86
	**α**	**Mn4**	2.94	2.89	2.90
	**α**	**Y_z•**	1.01	0.97	0.98
		**O5**	-0.01	-0.03	0.00
		**Ox**	0.33	0.14	-0.03
**12-et**	**α**	**Mn1**	2.69	2.85	3.00
	**α**	**Mn2**	2.99	2.98	2.94
	**α**	**Mn3**	2.89	3.06	2.86
	**α**	**Mn4**	2.94	2.89	2.90
	**β**	**Y_z•**	-1.02	-0.99	-0.99
		**O5**	-0.01	-0.03	0.00
		**Ox**	0.33	0.14	-0.03
**Octet**	**α**	**Mn1**	2.69	2.85	3.00
	**α**	**Mn2**	2.99	2.97	2.94
	**α**	**Mn3**	2.87	3.02	2.84
	**β**	**Mn4**	-2.87	-2.82	-2.83
	**α**	**Y_z•**	1.01	0.99	0.99
		**O5**	-0.01	-0.02	-0.03

		Ox	0.34	0.15	-0.03
	α	Mn1	2.69	2.83	2.95
	α	Mn2	2.98	2.98	2.97
	β	Mn3	-2.88	-2.93	-2.79
	α	Mn4	2.87	2.82	2.84
	α	$Y_z^•$	1.02	1.01	1.01
		O5	0.32	0.02	0.01
		Ox	0.04	0.16	0.05
	α	Mn1	2.68	2.87	3.02
	β	Mn2	-2.97	-2.96	-2.97
	α	Mn3	2.88	3.05	2.86
	α	Mn4	2.94	2.89	2.90
	α	$Y_z^•$	1.00	0.98	0.98
		O5	-0.02	-0.03	0.00
		Ox	0.32	0.13	-0.06
	β	Mn1	-2.69	-2.85	-2.95
	α	Mn2	2.98	2.97	2.94
	α	Mn3	2.89	2.99	2.82
	α	Mn4	2.94	2.89	2.90
	α	$Y_z^•$	1.01	1.00	1.00
		O5	-0.03	-0.02	0.02
		Ox	-0.32	-0.16	-0.04
Sextet	α	Mn1	2.69	2.85	3.00
	α	Mn2	2.99	2.97	2.94

α	Mn3	2.87	3.02	2.84
β	Mn4	-2.87	-2.82	-2.83
β	$Y_z^•$	-1.01	-0.99	-0.99
	O5	-0.01	-0.02	-0.03
	Ox	0.34	0.15	-0.03
α	Mn1	2.69	2.83	2.95
α	Mn2	2.98	2.98	2.97
β	Mn3	-2.88	-2.93	-2.79
α	Mn4	2.87	2.82	2.84
β	$Y_z^•$	-1.01	-1.00	-1.00
	O5	0.04	0.02	0.01
	Ox	0.32	0.16	0.05
α	Mn1	2.68	2.87	3.02
β	Mn2	-2.97	-2.96	-2.97
α	Mn3	2.88	3.05	2.86
α	Mn4	2.94	2.89	2.90
β	$Y_z^•$	-0.99	-0.98	-0.98
	O5	-0.02	-0.03	-0.01
	Ox	0.32	0.13	-0.06
β	Mn1	-2.69	-2.85	-2.95
α	Mn2	2.98	2.97	2.94
α	Mn3	2.89	2.99	2.82
α	Mn4	2.94	2.89	2.90
β	$Y_z^•$	-1.02	-1.01	-1.01

		**O5**	-0.03	-0.02	0.02
		**Ox**	-0.32	-0.16	-0.04
**Doublet**	α	**Mn1**	2.69	2.84	2.95
	α	**Mn2**	2.98	2.97	2.97
	β	**Mn3**	-2.89	-2.96	-2.81
	β	**Mn4**	-2.94	-2.89	-2.90
	α	**$Y_z^\bullet$**	1.01	1.00	1.00
		**O5**	0.04	0.02	-0.02
		**Ox**	0.33	0.16	0.05
	α	**Mn1**	2.68	-2.87	3.02
	β	**Mn2**	-2.97	-2.96	-2.98
	α	**Mn3**	2.87	3.01	2.84
	β	**Mn4**	-2.87	-2.82	-2.84
	α	**$Y_z^\bullet$**	1.01	1.00	0.99
		**O5**	-0.01	-0.03	-0.04
		**Ox**	0.33	0.14	-0.06
	α	**Mn1**	2.69	2.85	2.96
	β	**Mn2**	-2.98	-2.96	-2.94
	β	**Mn3**	-2.88	-2.96	-2.80
	α	**Mn4**	2.87	2.82	2.84
	α	**$Y_z^\bullet$**	1.02	1.00	0.99
		**O5**	0.03	0.01	0.01
		**Ox**	0.31	0.15	0.04
	β	**Mn1**	-2.69	-2.85	-2.96

	α	**Mn2**	2.98	2.96	2.94
	α	**Mn3**	2.88	2.96	2.80
	β	**Mn4**	-2.87	-2.82	-2.84
	α	**Y_z•**	1.01	0.99	0.98
		**O5**	-0.03	-0.01	-0.01
		**Ox**	-0.31	-0.15	-0.04
	β	**Mn1**	-2.68	-2.87	-3.02
	α	**Mn2**	2.97	2.96	2.98
	β	**Mn3**	-2.87	-3.01	-2.84
	α	**Mn4**	2.87	2.82	2.84
	α	**Y_z•**	1.01	0.98	0.99
		**O5**	0.01	0.03	0.04
		**Ox**	-0.33	-0.14	0.06
	β	**Mn1**	-2.69	-2.84	-2.95
	β	**Mn2**	-2.98	-2.97	-2.97
	α	**Mn3**	2.89	2.96	2.81
	α	**Mn4**	2.94	2.89	2.90
	α	**Y_z•**	1.02	0.99	0.99
		**O5**	-0.04	-0.02	-0.05
		**Ox**	-0.33	-0.16	0.02

Table S5. Gibbs free energies of $S_3^{A,W}Y_z$•(W1=H_2O)*, TS* and $S_3^{B,W}Y_z$•(W1=H_2O)* for 14-et spin state for A-B conversion when W2=OH^-, Ox=OH^- in the virtual $S_3^nY_z$•(W1=H_2O)* state.

A-B / Spin state	Gibbs free energy in a.u. (Relative free energy in kcal/mol)		
	**$S_3^{A,W}Y_z$•(W1=H_2O)***	**TS***	**$S_3^{B,W}Y_z$•(W1=H_2O)***

14-et	ααααα	-6527.8565 (0.0)	-6527.8187 (23.7)	-6527.8356 (13.1)

Table S6. Mulliken spin populations of the key atoms in $S_3^{A,W}Y_z$•($W1=H_2O$)*, TS* and $S_3^{B,W}Y_z$•($W1=H_2O$)* for all the possible spin states for A-B conversion when W2=OH¯, Ox=OH¯ in the virtual $S_3^nY_z$•($W1=H_2O$)* state.

A-B / Spin states			Mulliken spin population		
			**$S_3^{A,W}Y_z$•(W1=H_2O)***	**TS***	**$S_3^{B,W}Y_z$•(W1=H_2O)***
**14-et**	**α**	**Mn1**	2.94	2.84	2.99
	**α**	**Mn2**	3.01	2.99	2.96
	**α**	**Mn3**	2.86	3.01	2.86
	**α**	**Mn4**	2.98	2.97	2.94
	**α**	**Y_z•**	0.97	0.96	0.96
		**O5**	0.04	-0.01	0.04
		**Ox**	0.09	0.18	0.00

Table S7. Gibbs free energies of $S_3^{A,W}Y_z$(W1=OH¯)*, TS* and $S_3^{B,W}Y_z$(W1=OH¯)* for 13-et spin state for A-B conversion when W2=OH¯, Ox=OH¯ in the virtual $S_3^+Y_z$(W1=OH¯)* state.

A-B / Spin state		Gibbs free energy in a.u. (Relative free energy in kcal/mol)		
		**$S_3^{A,W}Y_z$(W1=OH¯)***	**TS***	**$S_3^{B,W}Y_z$(W1=OH¯)***
**13-et**	**αααα**	-6527.5942 (0.0)	-6527.5742 (12.6)	-6527.5936 (0.4)

Table S8. Mulliken spin populations of the key atoms in $S_3^{A,W}Y_z$(W1=OH¯)*, TS* and $S_3^{B,W}Y_z$(W1=OH¯)* for 13-et spin state spin state for A-B conversion when W2=OH¯, Ox=OH¯ in the virtual $S_3^+Y_z$(W1=OH¯)* state.

A-B / Spin states			Mulliken spin population		
			**$S_3^{A,W}Y_z$(W1=OH¯)***	**TS***	**$S_3^{B,W}Y_z$(W1=OH¯)***
**13-et**	**α**	**Mn1**	2.75	2.88	2.98
	**α**	**Mn2**	2.98	2.96	2.92
	**α**	**Mn3**	2.90	3.04	2.85
	**α**	**Mn4**	2.96	2.90	2.91
		**O5**	0.00	-0.02	0.01

	Ox	0.28	0.12	-0.02

Table S9. Calculated Wiberg bond orders of the key bonds in $S_3^{A,W}Y_z(W1=H_2O)$ and $S_3^{A,W}Y_z$•(W1=OH⁻).

**Key bonds**	**Wiberg bond orders**		
	**$S_3^{A,W}Y_z$ (W1=H_2O)**	**$S_3^{A,W}Y_z$•(W1=OH⁻) (OxH)**	**$S_3^{A,W}Y_z$•(W1=OH⁻) (O5H)**
**Mn4-W1**	0.42	0.64	0.65
**Mn4-O5**	0.44	0.24	0.22
**Mn3-O5**	0.53	0.57	0.44

Table S10. Gibbs free energies of $S_3^{A,W}Y_z(W1=H_2O)$, TS and $S_3^{B,W}Y_z(W1=H_2O)$ for all the possible spin states for A-B conversion when W2=H_2O, Ox=OH⁻ in the $S_3^{+}Y_z$ state.

**A-B / Spin states**		**Gibbs free energy in a.u. (Relative free energy in kcal/mol)**		
		**$S_3^{A,W}Y_z(W1=H_2O)$**	**TS**	**$S_3^{B,W}Y_z(W1=H_2O)$**
**13-et**	**αααα**	-6528.5013 (0.0)	-6528.4579 (27.2)	-6528.4776 (14.9)
**Septet**	**αααβ**	-6528.5002 (0.7)	-6528.4574 (27.5)	-6528.4765 (15.6)
	**ααβα**	-6528.5013 (0.0)	-6528.4575 (27.5)	-6528.4771 (15.2)
	**αβαα**	-6528.4994 (2.2)	-6528.4577 (27.4)	-6528.4750 (15.2)
	**βααα**	-6528.5003 (0.6)	-6528.4563 (28.2)	-6528.4756 (16.1)
**Singlet**	**ααββ**	-6528.4983 (1.9)	-6528.4566 (28.0)	-6528.4735 (17.4)
	**αβαβ**	-6528.4988 (1.6)	-6528.4535 (30.0)	-6528.4727 (17.9)
	**βααβ**	-6528.4999 (0.9)	-6528.4548 (29.2)	-6528.4733 (17.6)

Table S11. Gibbs free energies of $S_3^{A,W}Y_z$•(W1=OH⁻), TS and $S_3^{B,W}Y_z$•(W1=OH⁻) for all the possible spin states for A-B conversion when W2=H_2O, Ox=OH⁻ in the $S_3^{n}Y_z$• state.

**A-B / Spin states**		**Gibbs free energy in a.u. (Relative free energy in kcal/mol)**		
		**$S_3^{A,W}Y_z$•(W1=OH⁻)**	**TS**	**$S_3^{B,W}Y_z$•(W1=OH⁻)**
**14-et**	**ααααα**	-6527.8377 (0.6)	-6527.8129 (16.1)	-6527.8380 (0.4)
**12-et**	**ααααβ**	-6527.8375 (0.7)	-6527.8128 (16.2)	-6527.8380 (0.4)
**Octet**	**αααβα**	-6527.8361 (1.6)	-6527.8139 (15.5)	-6527.8378 (0.5)

	**αaβαα**	-6527.8386 (0.0)	-6527.8120 (16.7)	-6527.8367 (1.2)
	**αβααα**	-6527.8361 (1.6)	-6527.8110 (17.3)	-6527.8359 (1.7)
	**βαααα**	-6527.8359 (1.7)	-6527.8107 (17.5)	-6527.8362 (1.5)
**Sextet**	**αααββ**	-6527.8372 (0.9)	-6527.8126 (16.3)	-6527.8376 (0.6)
	**ααβαβ**	-6527.8383 (0.2)	-6527.8117 (16.9)	-6527.8364 (1.4)
	**αβααβ**	-6527.8359 (1.7)	-6527.8107 (17.5)	-6527.8356 (1.9)
	**βαααβ**	-6527.8380 (0.4)	-6527.8113 (17.1)	-6527.8359 (1.7)
**Doublet**	**ααββα**	-6527.8367 (1.2)	-6527.8100 (17.9)	-6527.8347 (2.4)
	**αβαβα**	-6527.8372 (0.9)	-6527.8106 (17.6)	-6527.8353 (2.1)
	**αββαα**	-6527.8377 (0.6)	-6527.8115 (17.0)	-6527.8366 (1.3)
	**βααβα**	-6527.8372 (0.9)	-6527.8106 (17.6)	-6527.8352 (2.1)
	**βαβαα**	-6527.8354 (2.0)	-6527.8103 (17.8)	-6527.8353 (2.1)
	**ββααα**	-6527.8367 (1.2)	-6527.8100 (17.9)	-6527.8347 (2.4)

Table S12. Gibbs free energies of $S_3^{A,W}Y_z(W1=H_2O)$, TS and $S_3^{B,W}Y_z(W1=H_2O)$ for all the possible spin states for A-B conversion when W2=OH$^-$, Ox=oxo in the $S_3^+Y_z$ state.

**A-B** / **Spin states**		**Gibbs free energy in a.u. (Relative free energy in kcal/mol)**		
		**$S_3^{A,W}Y_z(W1=H_2O)$**	**TS**	**$S_3^{B,W}Y_z(W1=H_2O)$**
**13-et**	**αααα**	-6527.5950 (0.0)	-6529.5562 (24.3)	-6529.5740 (13.2)
**Septet**	**αααβ**	-6529.5944 (0.4)	-6529.5585 (22.9)	-6529.5715 (14.7)
	**ααβα**	-6529.5948 (0.1)	-6529.5578 (23.3)	-6529.5699 (15.8)
	**αβαα**	-6529.5933 (1.1)	-6529.5535 (26.0)	-6529.5761 (11.9)
	**βααα**	-6527.5933 (1.1)	-6529.5537 (25.9)	-6529.5690 (16.3)
**Singlet**	**ααββ**	-6529.5914 (2.3)	-6529.5515 (27.3)	-6529.5669 (17.6)
	**αβαβ**	-6529.5918 (2.0)	-6529.5579 (23.3)	-6529.5685 (16.6)
	**βααβ**	-6529.5931 (1.2)	-6529.5548 (25.2)	-6529.5685 (16.6)

Table S13. Gibbs free energies of $S_3^AY_z(W1=H_2O)$ for all the possible spin states when W2=H_2O, Ox=oxo in the $S_3^+Y_z$ state.

**A-B**	**Gibbs free energy (a.u.)**

Spin states		$S_3^{A,W}Y_z(W1=H_2O)$
**13-et**	**αααα**	-6528.0520
**Septet**	**αααβ**	-6528.0520
	**ααβα**	-6528.0523
	**αβαα**	-6528.0504
	**βααα**	-6528.0507
**Singlet**	**ααββ**	-6528.0487
	**αβαβ**	-6528.0491
	**βααβ**	-6528.0505

Table S14. A summary of the effect of different protonation states on the structural isomerization in the $S_3^+Y_z$ and $S_3^nY_z^\bullet$ states.

Protonation states	(i) $S_3^+Y_z$ (W1=H_2O)	(ii) $S_3^nY_z^\bullet$ (W1=OH^-)
a. W2=OH^-, Ox=OH^-	Unidirectional structural isomerization $S_3^{B,W}Y_z \rightarrow S_3^{A,W}Y_z$: $\Delta G^{\neq}(S_3^{A,W}Y_z \rightarrow S_3^{B,W}Y_z)$= 24.3~25.6 kcal/mol; $\Delta G(S_3^{A,W}Y_z \rightarrow S_3^{B,W}Y_z)$= 13.4~14.8 kcal/mol.	Reversible structural isomerization $S_3^{A,W}Y_z^\bullet \rightleftharpoons S_3^{B,W}Y_z^\bullet$: $\Delta G^{\neq}(S_3^{A,W}Y_z^\bullet \rightarrow S_3^{B,W}Y_z^\bullet)$= 14.8~16.2 kcal/mol; $\Delta G(S_3^{A,W}Y_z^\bullet \rightarrow S_3^{B,W}Y_z^\bullet)$= 2.4~3.9 kcal/mol.
b. W2=H_2O, Ox=OH^-	Unidirectional structural isomerization $S_3^{B,W}Y_z \rightarrow S_3^{A,W}Y_z$: $\Delta G^{\neq}(S_3^{A,W}Y_z \rightarrow S_3^{B,W}Y_z)$= 25.2~28.4 kcal/mol; $\Delta G(S_3^{A,W}Y_z \rightarrow S_3^{B,W}Y_z)$= 14.9~16.7 kcal/mol.	Reversible structural isomerization $S_3^{A,W}Y_z^\bullet \rightleftharpoons S_3^{B,W}Y_z^\bullet$: $\Delta G^{\neq}(S_3^{A,W}Y_z^\bullet \rightarrow S_3^{B,W}Y_z^\bullet)$= 13.5~16.7 kcal/mol; $\Delta G(S_3^{A,W}Y_z^\bullet \rightarrow S_3^{B,W}Y_z^\bullet)$= -1.1~1.3 kcal/mol.
c. W2=OH^-, Ox=oxo	Unidirectional structural isomerization $S_3^{B,W}Y_z \rightarrow S_3^{A,W}Y_z$: $\Delta G^{\neq}(S_3^{A,W}Y_z \rightarrow S_3^{B,W}Y_z)$= 22.5~25.0 kcal/mol;	This protonation state violates the electrostatic rule that leads to the deprotonated W1 from its preceding state (c, i); and any

	$\Delta G(S_3{}^{A,W}Y_z \rightarrow S_3{}^{B,W}Y_z)$= 13.2~15.7 kcal/mol.	tentative optimization of the (c, ii) state would lead to reduction of $Y_z^{\bullet}$ and oxidation of Ox, forming S_4 instead of $S_3{}^{n}Y_z^{\bullet}$.
d. W2=H_2O, Ox=oxo	This proton distribution is calculated to be ca. 10 kcal/mol higher than the (a, i) state. in terms of $S_3{}^{A,W}Y_z(W1=H_2O)$, therefore, it is not a reasonable model, and the isomerization was not investigated further.	Given the exclusion of the model in situation (d, i), its derived tyrosine-oxidized state (d, ii) is also impossible and was not investigated further.
e. W2=OH^{-}/H_2O, Ox=oxyl	The oxyl form of Ox would cause Mn4 reduction to valence (III) in the $S_3{}^{+}Y_z$ state which is inconsistent with spectroscopic observations and would destabilize W1=OH^{-} coordination to Mn4 in the $S_3{}^{n}Y_z^{\bullet}$ state, thus it was not considered for the isomerization.	

Table S15. Gibbs free energies of $S_3{}^{A,W}Y_z^{\bullet}(W1=OH^{-})$, TS and $S_3{}^{B,W}Y_z^{\bullet}(W1=OH^{-})$ for all the possible spin states for A-B conversion when W2=H_2O, Ox=oxo in the $S_3{}^{n}Y_z^{\bullet}$ state calculated by B3LYP-D3(BJ).

A-B / Spin states		Gibbs free energy in a.u. (Relative free energy in kcal/mol)		
		**$S_3{}^{A,W}Y_z^{\bullet}(W1=OH^{-})$**	**TS**	**$S_3{}^{B,W}Y_z^{\bullet}(W1=OH^{-})$**
**14-et**	**ααααα**	-6531.0087 (0.6)	-6530.9843 (15.9)	-6531.0053 (2.8)
**12-et**	**ααααβ**	-6531.0086 (0.7)	-6530.9847 (15.7)	-6531.0052 (2.8)
**Octet**	**αααβα**	-6531.0092 (0.3)	-6530.9857 (15.1)	-6531.0058 (2.4)
	**ααβαα**	-6531.0097 (0.0)	-6530.9839 (16.2)	-6531.0041 (3.5)
	**αβααα**	-6531.0075 (1.4)	-6530.9830 (16.8)	-6531.0037 (3.8)
	**βαααα**	-6531.0085 (0.8)	-6530.9831 (16.7)	-6531.0031 (4.1)
**Sextet**	**αααββ**	-6531.0089 (0.5)	-6530.9849 (15.6)	-6531.0054 (2.7)
	**ααβαβ**	-6531.0094 (0.2)	-6530.9836 (16.4)	-6531.0038 (3.7)

	**αβααβ**	-6531.0072 (1.6)	-6530.9827 (16.9)	-6531.0035 (4.3)
	**βαααβ**	-6531.0083 (0.9)	-6530.9828 (16.9)	-6531.0028 (4.3)
**Doublet**	**ααββα**	-6531.0072 (1.6)	-6530.9816 (17.6)	-6531.0017 (5.0)
	**αβαβα**	-6531.0073 (1.5)	-6530.9826 (17.0)	-6531.0035 (3.9)
	**αββαα**	-6531.0083 (0.9)	-6530.9826 (17.0)	-6531.0027 (4.4)
	**βααβα**	-6531.0083 (0.9)	-6530. 9826 (17.0)	-6531.0027 (4.4)
	**βαβαα**	-6531.0072 (1.6)	-6530.9826 (17.0)	-6531.0036 (3.8)
	**ββααα**	-6531.0072 (1.6)	-6530.9815 (17.7)	-6531.0016 (5.1)

VI. Supplementary figures

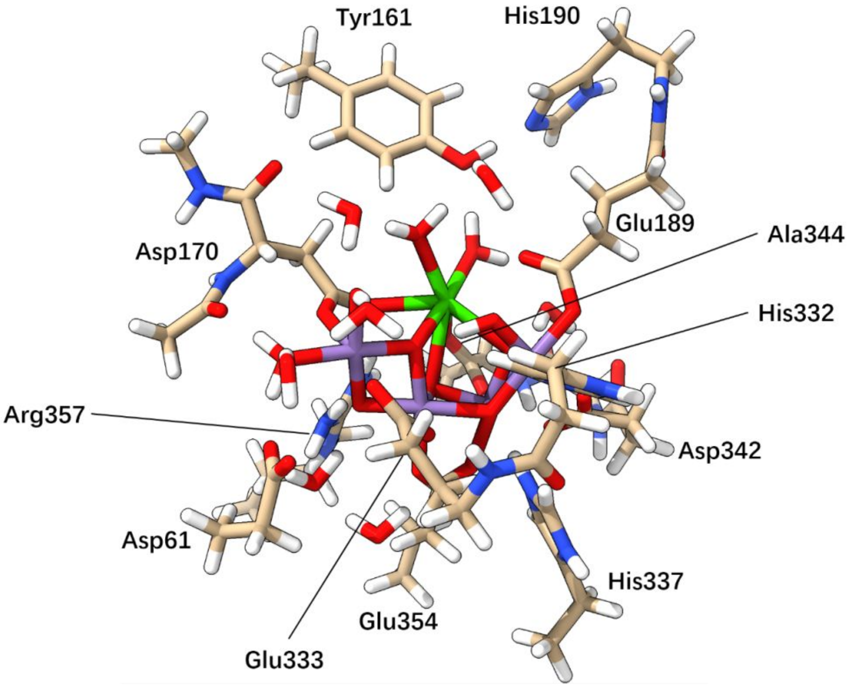

Figure S1. The model size exemplified by the $S_3^{A,W}Y_z$ (W1=H_2O) state

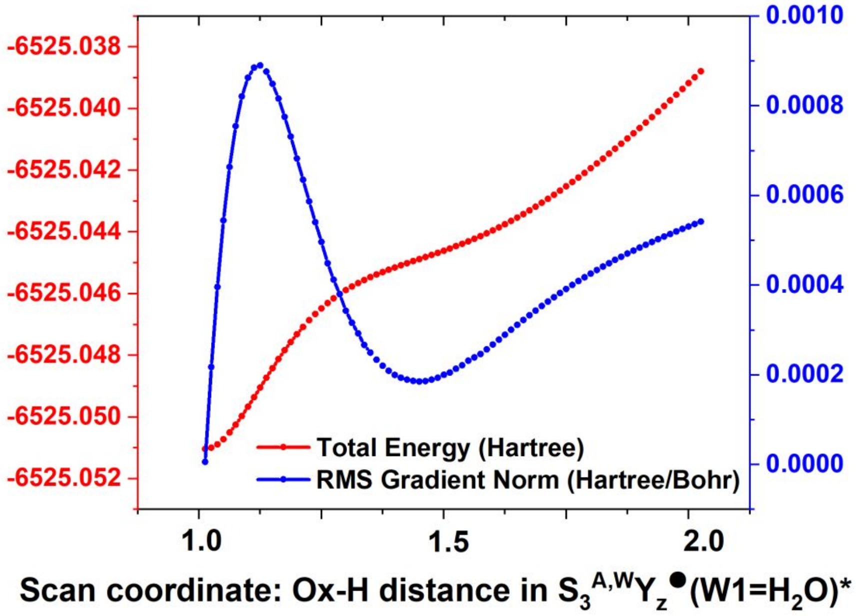

Figure S2. Relaxed PES scan curve of proton transfer between Ox and O5 in $S_3^{A,W}Y_z^{\bullet}(W1{=}H_2O)^*$ of 14-et spin state.

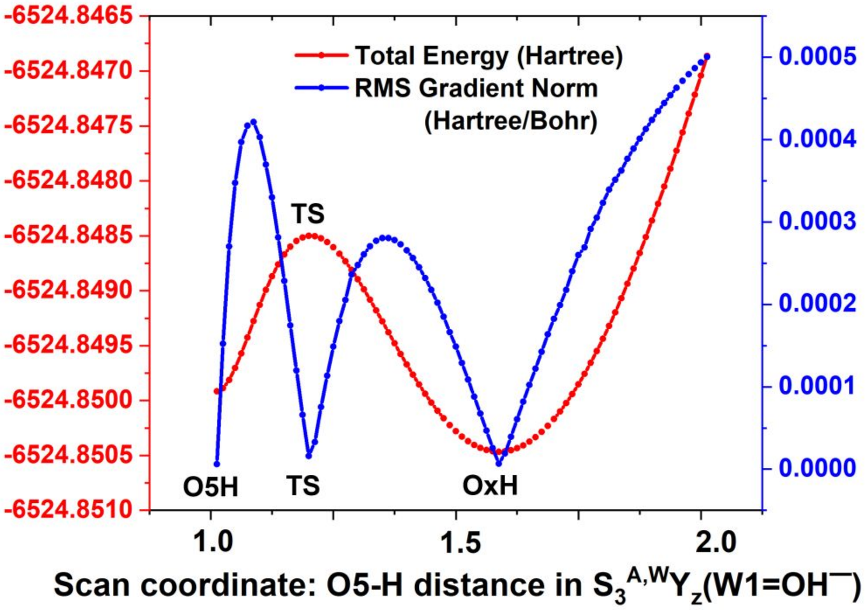

Figure S3. Relaxed PES scan curve of proton transfer between Ox and O5 in $S_3^{A,W}Y_z(W1{=}OH^{-})^*$ of 14-et spin state.

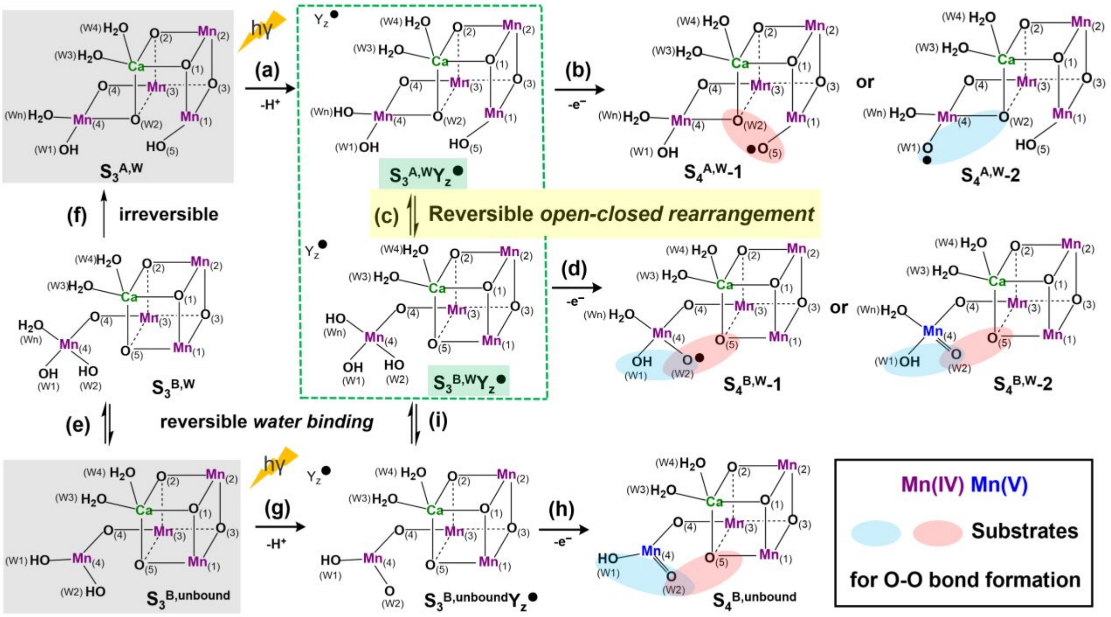

Figure S4. Possible mechanisms of the $S_3 \rightarrow S_4$ transition and O-O bond formation in the S_4 state. The ligand labelling is based on $S_3^{B,unbound}$ and the pivot/carousel water insertion during the $S_2 \rightarrow S_3$ transition, in contrast to that in the main text.

VII. Supplementary references

(1) Cox, N.; Retegan, M.; Neese, F.; Pantazis, D. A.; Boussac, A.; Lubitz, W., Electronic structure of the oxygen-evolving complex in photosystem II prior to O-O bond formation. *Science* **2014,** *345*, 804-808.

(2) Kern, J.; Chatterjee, R.; Young, I. D.; Fuller, F. D.; Lassalle, L.; Ibrahim, M.; Gul, S.; Fransson, T.; Brewster, A. S.; Alonso-Mori, R.; Hussein, R.; Zhang, M.; Douthit, L.; de Lichtenberg, C.; Cheah, M. H.; Shevela, D.; Wersig, J.; Seuffert, I.; Sokaras, D.; Pastor, E.; Weninger, C.; Kroll, T.; Sierra, R. G.; Aller, P.; Butryn, A.; Orville, A. M.; Liang, M.; Batyuk, A.; Koglin, J. E.; Carbajo, S.; Boutet, S.; Moriarty, N. W.; Holton, J. M.; Dobbek, H.; Adams, P. D.; Bergmann, U.; Sauter, N. K.; Zouni, A.; Messinger, J.; Yano, J.; Yachandra, V. K., Structures of the intermediates of Kok's photosynthetic water oxidation clock. *Nature* **2018,** *563*, 421-425.

(3) Suga, M.; Akita, F.; Yamashita, K.; Nakajima, Y.; Ueno, G.; Li, H.; Yamane, T.; Hirata, K.; Umena, Y.; Yonekura, S.; Yu, L.-J.; Murakami, H.; Nomura, T.; Kimura, T.; Kubo, M.; Baba, S.; Kumasaka, T.; Tono, K.; Yabashi, M.; Isobe, H.; Yamaguchi, K.; Yamamoto, M.; Ago, H.; Shen, J.-R., An oxyl/oxo mechanism for oxygen-oxygen coupling in PSII revealed by an x-ray free-electron laser. *Science* **2019,** *366*, 334-338.

(4) Mandal, M.; Saito, K.; Ishikita, H., The Nature of the short oxygen–oxygen distance in the Mn_4CaO_6 complex of photosystem II crystals. *J. Phys. Chem. Lett.* **2020,** *11*, 10262-10268.

(5) Pantazis, D. A., The S_3 state of the oxygen-evolving complex: overview of spectroscopy and XFEL crystallography with a critical evaluation of early-onset models for O-O bond formation. *Inorganics* **2019,** *7*, 55.

(6) Wang, J.; Askerka, M.; Brudvig, G. W.; Batista, V. S., Crystallographic data support the carousel mechanism of water supply to the oxygen-evolving complex of photosystem II. *ACS Energy Lett.* **2017,** *2*, 2299–2306.

(7) Nakamura, S.; Noguchi, T., Quantum mechanics/molecular mechanics simulation of the ligand vibrations of the water-oxidizing Mn_4CaO_5 cluster in photosystem II. *Proc. Natl. Acad. Sci. U.S.A.* **2016,** *113*, 12727-12732.

(8) Ames, W.; Pantazis, D. A.; Krewald, V.; Cox, N.; Messinger, J.; Lubitz, W.; Neese, F., Theoretical evaluation of structural models of the S_2 state in the oxygen evolving complex of photosystem II: protonation states and magnetic interactions. *J. Am. Chem. Soc.* **2011,** *133*, 19743-19757.

(9) Siegbahn, P. E. M., Computational investigations of S_3 structures related to a recent X-ray free electron laser study. *Chem. Phys. Lett.* **2017,** *690*, 172-176.

(10) Krewald, V.; Retegan, M.; Cox, N.; Messinger, J.; Lubitz, W.; DeBeer, S.; Neese, F.; Pantazis, D. A., Metal oxidation states in biological water splitting. *Chem. Sci.* **2015,** *6*, 1676-1695.

(11) Gaspar, A. B.; Weber, B., *Spin crossover phenomenon in coordination compounds*. Molecular Magnetic Materials: 2016; Vol. pp. 231-252.

(12) Spiering, H.; Kohlhaas, T.; Romstedt, H.; Hauser, A.; Bruns-Yilmaz, C.; Kusz, J.; Gütlich, P., Correlations of the distribution of spin states in spin crossover compounds. *Coord. Chem. Rev.* **1999,** *190-192*, 629-647.

(13) Poli, R.; Harvey, J. N., Spin Forbidden Chemical Reactions of Transition Metal Compounds. New Ideas and New Computational Challenges. *Chem. Soc. Rev.* **2003,** *32*, 1-8.

(14) Harvey, J. N.; Poli, R.; Smith, K. M., Understanding the reactivity of transition metal complexes involving multiple spin states. *Coord. Chem. Rev.* **2003,** *238-239*, 347-361.

(15) Harvey, J. N., Spin-forbidden reactions: computational insight into mechanisms and kinetics. *WIREs Comput. Mol. Sci.* **2014,** *4*, 1-14.

(16) Siegbahn, P. E. M., The effect of backbone constraints: the case of water oxidation by the oxygen-evolving complex in PSII. *ChemPhysChem* **2011,** *12*, 3274-3280.

(17) Siegbahn, P. E. M.; Blomberg, M. R. A., Energy diagrams for water oxidation in photosystem II using different density functionals. *J. Chem. Theory Comput.* **2014,** *10*, 268-272.

(18) Marenich, A. V.; Cramer, C. J.; Truhlar, D. G., Universal solvation model based on solute electron density and on a continuum model of the solvent defined by the bulk dielectric constant and atomic surface tensions. *J. Phys. Chem. B* **2009,** *113*, 6378-6396.

(19) Frisch, M. J.; Trucks, G. W.; Schlegel, H. B.; Scuseria, G. E.; Robb, M. A.; Cheeseman, J. R.; Scalmani, G.; Barone, V.; Petersson, G. A.; Nakatsuji, H.; Li, X.; Caricato, M.; Marenich, A. V.; Bloino, J.; Janesko, B. G.; Gomperts, R.; Mennucci, B.; Hratchian, H. P.; Ortiz, J. V.; Izmaylov, A. F.; Sonnenberg, J. L.; Williams; Ding, F.; Lipparini, F.; Egidi, F.; Goings, J.; Peng, B.; Petrone, A.; Henderson, T.; Ranasinghe, D.; Zakrzewski, V. G.; Gao, J.; Rega, N.; Zheng, G.; Liang, W.; Hada, M.; Ehara, M.; Toyota, K.; Fukuda, R.; Hasegawa, J.; Ishida, M.; Nakajima, T.; Honda, Y.; Kitao, O.; Nakai, H.; Vreven, T.; Throssell, K.; Montgomery Jr., J. A.; Peralta, J. E.; Ogliaro, F.; Bearpark, M. J.; Heyd, J. J.; Brothers, E. N.; Kudin, K. N.; Staroverov, V. N.; Keith, T. A.; Kobayashi, R.; Normand, J.; Raghavachari, K.; Rendell, A. P.; Burant, J. C.; Iyengar, S. S.; Tomasi, J.; Cossi, M.; Millam, J. M.; Klene, M.; Adamo, C.; Cammi, R.; Ochterski, J. W.; Martin, R. L.; Morokuma, K.; Farkas, O.; Foresman, J. B.; Fox, D. J. *Gaussian 16 Rev. C.01*, Wallingford, CT, 2019.

(20) Lu, T.; Chen, F., Multiwfn: A multifunctional wavefunction analyzer. *J. Comput. Chem.* **2012,** *33*, 580-592.

(21) Thom, A. J. W.; Sundstrom, E. J.; Head-Gordon, M., LOBA: a localized orbital bonding analysis to calculate oxidation states, with application to a model water oxidation catalyst. *Phys.*

Chem. Chem. Phys. **2009,** *11*, 11297-11304.
(22) Eyring, H., The activated complex in chemical reactions. *J. Chem. Phys.* **1935,** *3*, 107-115.
(23) Laidler, K. J.; King, M. C., Development of transition-state theory. *J. Phys. Chem.* **1983,** *87*, 2657-2664.
(24) Truhlar, D. G.; Garrett, B. C.; Klippenstein, S. J., Current Status of Transition-State Theory. *J. Phys. Chem.* **1996,** *100*, 12771-12800.
(25) Gupta, R.; Taguchi, T.; Lassalle-Kaiser, B.; Bominaar, E. L.; Yano, J.; Hendrich, M. P.; Borovik, A. S., High-spin Mn-oxo complexes and their relevance to the oxygen-evolving complex within photosystem II. *Proc. Natl. Acad. Sci. U.S.A.* **2015,** *112*, 5319-5324.
(26) Nilsson, H.; Cournac, L.; Rappaport, F.; Messinger, J.; Lavergne, J., Estimation of the driving force for dioxygen formation in photosynthesis. *Biochim. Biophys. Acta, Bioenerg.* **2016,** *1857*, 23-33.
(27) Pantazis, D. A.; Ames, W.; Cox, N.; Lubitz, W.; Neese, F., Two interconvertible structures that explain the spectroscopic properties of the oxygen-evolving complex of photosystem II in the S_2 state. *Angew. Chem. Int. Ed.* **2012,** *51*, 9935-9940.
(28) Bovi, D.; Narzi, D.; Guidoni, L., The S_2 state of the oxygen-evolving complex of photosystem II explored by QM/MM dynamics: spin surfaces and metastable states suggest a reaction path towards the S_3 state. *Angew. Chem. Int. Ed.* **2013,** *52*, 1-6.
(29) Siegbahn, P. E. M., The S_2 to S_3 transition for water oxidation in PSII (photosystem II), revisited. *Phys. Chem. Chem. Phys.* **2018,** *20*, 22926-22931.
(30) Ugur, I.; Rutherford, A. W.; Kaila, V. R. I., Redox-coupled substrate water reorganization in the active site of photosystem II-the role of calcium in substrate water delivery. *Biochim. Biophys. Acta, Bioenerg.* **2016,** *1857*, 740-748.
(31) Boussac, A.; Ugur, I.; Marion, A.; Sugiura, M.; Kaila, V. R. I.; Rutherford, A. W., The low spin-high spin equilibrium in the S_2-state of the water oxidizing enzyme. *Biochim. Biophys. Acta Bioenerg.* **2018,** *1859*, 342-356.
(32) Vinyard, D. J.; Khan, S.; Askerka, M.; Batista, V. S.; Brudvig, G. W., Energetics of the S_2 state spin isomers of the oxygen-evolving complex of photosystem II. *J. Phys. Chem. B* **2017,** *121*, 1020-1025.
(33) Isobe, H.; Shoji, M.; Shen, J.-R.; Yamaguchi, K., Strong coupling between the hydrogen bonding environment and redox chemistry during the S_2 to S_3 transition in the oxygen-evolving complex of photosystem II. *J. Phys. Chem. B* **2015,** *119*, 13922-13933.
(34) Saitow, M.; Becker, U.; Riplinger, C.; Valeev, E. F.; Neese, F., A new near-linear scaling, efficient and accurate, open-shell domain-based local pair natural orbital coupled cluster singles and doubles theory. *J. Chem. Phys.* **2017,** *146*, 164105
(35) Lohmiller, T.; Krewald, V.; Navarro, M. P.; Retegan, M.; Rapatskiy, L.; Nowaczyk, M. M.; Boussac, A.; Neese, F.; Lubitz, W.; Pantazis, D. A.; Cox, N., Structure, ligands and substrate coordination of the oxygen-evolving complex of photosystem II in the S_2 state: a combined EPR and DFT study. *Phys. Chem. Chem. Phys.* **2014,** *16*, 11877-11892.
(36) Narzi, D.; Bovi, D.; Guidoni, L., Pathway for Mn-cluster oxidation by tyrosine-Z in the S_2 state of photosystem II. *Proc. Natl. Acad. Sci. U.S.A.* **2014,** *111*, 8723-8728.
(37) Drosou, M.; Zahariou, G.; Pantazis, D. A., Orientational Jahn–Teller isomerism in the dark-stable state of nature's water oxidase. *Angew. Chem. Int. Ed.* **2021,** *60*, 13493-13499.
(38) Krewald, V.; Neese, F.; Pantazis, D. A., Implications of structural heterogeneity for the

electronic structure of the final oxygen-evolving intermediate in photosystem II. *J. Inorg. Biochem.* **2019,** *199*, 110797.
(39) Zahariou, G.; Ioannidis, N.; Sanakis, Y.; Pantazis, D. A., Arrested substrate binding resolves catalytic intermediates in higher-plant water oxidation. *Angew. Chem. Int. Ed.* **2021,** *60*, 3156-3162.
(40) Cox, N.; Messinger, J., Reflections on substrate water and dioxygen formation. *Biochim. Biophys. Acta, Bioenerg.* **2013,** *1827*, 1020-1030.
(41) Li, X.; Siegbahn, P. E. M., Alternative mechanisms for O_2 release and O-O bond formation in the oxygen evolving complex of photosystem II. *Phys. Chem. Chem. Phys.* **2015,** *17*, 12168-12174.
(42) Zhang, B.; Sun, L., Why nature chose the Mn_4CaO_5 cluster as water-splitting catalyst in photosystem II: a new hypothesis for the mechanism of O-O bond formation. *Dalton Trans.* **2018,** *47*, 14381-14387.
(43) Orio, M.; Pantazis, D. A., Successes, challenges, and opportunities for quantum chemistry in understanding metalloenzymes for solar fuels research. *Chem. Commun.* **2021,** *57*, 3952-3974.
(44) Siegbahn, P. E. M., Water oxidation mechanism in photosystem II, including oxidations, proton release pathways, O-O bond formation and O_2 release. *Biochim. Biophys. Acta, Bioenerg.* **2013,** *1827*, 1003-1019.
(45) Siegbahn, P. E. M., The performance of hybrid DFT for mechanisms involving transition metal complexes in enzymes. *J. Biol. Inorg. Chem.* **2006,** *11*, 695–701.
(46) Siegbahn, P. E. M., Structures and energetics for O_2 formation in photosystem II. *Acc. Chem. Res.* **2009,** *42*, 1871-1880.
(47) Blomberg, M. R. A.; Borowski, T.; Himo, F.; Liao, R.-Z.; Siegbahn, P. E. M., Quantum chemical studies of mechanisms for metalloenzymes. *Chem. Rev.* **2014,** *114*, 3601-3658.
(48) Siegbahn, P. E. M., Substrate water exchange for the oxygen evolving complex in PSII in the S_1, S_2, and S_3 states. *J. Am. Chem. Soc.* **2013,** *135*, 9442-9449.
(49) Retegan, M.; Krewald, V.; Mamedov, F.; Neese, F.; Lubitz, W.; Cox, N.; Pantazis, D. A., A five-coordinate Mn(IV) intermediate in biological water oxidation: spectroscopic signature and a pivot mechanism for water binding. *Chem. Sci.* **2016,** *7*, 72-84.
(50) Chrysina, M.; Heyno, E.; Kutin, Y.; Reus, M.; Nilsson, H.; Nowaczyk, M. M.; DeBeer, S.; Neese, F.; Messinger, J.; Lubitz, W.; Cox, N., Five-coordinate Mn^{IV} intermediate in the activation of nature's water splitting cofactor. *Proc. Natl. Acad. Sci. U.S.A.* **2019,** *116*, 16841-16846.
(51) Cramer, C. J.; Truhlar, D. G., Density functional theory for transition metals and transition metal chemistry. *Phys. Chem. Chem. Phys.* **2009,** *11*, 10757-10816.
(52) Cohen, A. J.; Mori-Sánchez, P.; Yang, W., Challenges for Density Functional Theory. *Chem. Rev.* **2012,** *112*, 289-320.
(53) Siegbahn, P. E. M.; Blomberg, M. R. A., Density functional theory of biologically relevant metal centers. *Annu. Rev. Phys. Chem.* **1999,** *50*, 221-249.
(54) Crabtree, R. H., Hypervalency, secondary bonding and hydrogen bonding: siblings under the skin. *Chem. Soc. Rev.* **2017,** *46*, 1720-1729.
(55) Siegbahn, P. E. M.; Himo, F., The quantum chemical cluster approach for modeling enzyme reactions. *WIREs Comput. Mol. Sci.* **2011,** *1*, 323-336.
(56) Li, X.; Siegbahn, P. E. M.; Ryde, U., Simulation of the isotropic EXAFS spectra for the S_2 and S_3 structures of the oxygen evolving complex in photosystem II. *Proc. Natl. Acad. Sci. U.S.A.* **2015,** *112*, 3979-3984.
(57) Narzi, D.; Capone, M.; Bovi, D.; Guidoni, L., Evolution from S_3 to S_4 states of the oxygen-

evolving complex in photosystem II monitored by quantum mechanics/molecular mechanics (QM/MM) dynamics. *Chem. Eur. J.* **2018,** *24*, 10820-10828.
(58) Capone, M.; Bovi, D.; Narzi, D.; Guidoni, L., Reorganization of substrate waters between the closed and open cubane conformers during the S_2 to S_3 transition in the oxygen evolving complex. *Biochemistry* **2015,** *54*, 6439-6442.
(59) Isobe, H.; Shoji, M.; Suzuki, T.; Shen, J.-R.; Yamaguchi, K., Exploring reaction pathways for the structural rearrangements of the Mn cluster induced by water binding in the S3 state of the oxygen evolving complex of photosystem II. *J. Photochem. Photobiol., A* **2021,** *405*, 112905.
(60) de Lichtenberg, C.; Kim, C. J.; Chernev, P.; Debus, R. J.; Messinger, J., The exchange of the fast substrate water in the S_2 state of photosystem II is limited by diffusion of bulk water through channels – implications for the water oxidation mechanism. *Chem. Sci.* **2021,** *12*, 12763-12775.
(61) Klauss, A.; Haumann, M.; Dau, H., Alternating electron and proton transfer steps in photosynthetic water oxidation. *Proc. Natl. Acad. Sci. U.S.A.* **2012,** *109*, 16035-16040.
(62) Kawashima, K.; Takaoka, T.; Kimura, H.; Saito, K.; Ishikita, H., O_2 evolution and recovery of the water-oxidizing enzyme. *Nat. Commun.* **2018,** *9*, 1247.
(63) Meyer, T. J.; Huynh, M. H. V.; Thorp, H. H., The possible role of proton-coupled electron transfer (PCET) in water oxidation by photosystem II. *Angew. Chem. Int. Ed.* **2007,** *46*, 5284-5304.
(64) Dilbeck, P. L.; Hwang, H. J.; Zaharieva, I.; Gerencser, L.; Dau, H.; Burnap, R. L., The D1-D61N mutation in synechocystis sp. PCC 6803 allows the observation of pH-sensitive intermediates in the formation and release of O_2 from photosystem II. *Biochemistry* **2012,** *51*, 1079-1091.
(65) Siegbahn, P. E. M., Mechanisms for proton release during water oxidation in the S_2 to S_3 and S_3 to S_4 transitions in photosystem II. *Phys. Chem. Chem. Phys.* **2012,** *14*, 4849-4856.
(66) Bao, H.; Burnap, R. L., Structural rearrangements preceding dioxygen formation by the water oxidation complex of photosystem II. *Proc. Natl. Acad. Sci. U.S.A.* **2015,** *112*, 6139-6147.
(67) Narzi, D.; Mattioli, G.; Bovi, D.; Guidoni, L., A Spotlight on the Compatibility between XFEL and Ab Initio Structures of the Oxygen Evolving Complex in Photosystem II. *Chem. Eur. J.* **2017,** *23*, 6969 - 6973.
(68) Miyagawa, K.; Isobe, H.; Kawakami, T.; Shoji, M.; Yamanaka, S.; Okumura, M.; Nakajima, T.; Yamaguchi, K., Domain-based local pair natural orbital CCSD(T) calculations of fourteen different S_2 intermediates for water oxidation in the Kok cycle of OEC of PSII. Re-visit to one LS-two HS model for the S_2 state. *Chem. Phys. Lett.* **2019,** *734*, 136731.
(69) Barber, J.; Anderson, J. M.; Junge, W.; Haumann, M.; Ahlbrink, R.; Mulkidjanian, A.; Clausen, J., Electrostatics and proton transfer in photosynthetic water oxidation. *Philos. Trans. R. Soc., B* **2002,** *357*, 1407-1418.
(70) Boussac, A.; Setif, P.; Rutherford, A. W., Inhibition of tyrosine Z photooxidation after formation of the S_3-state in calcium-depleted and chloride-depleted photosystem-II. *Biochemistry* **1992,** *31*, 1224-1234.
(71) Lavergne, J.; Junge, W., Proton release during the redox cycle of the water oxidase. *Photosynth. Res.* **1993,** *38*, 279-296.
(72) McEvoy, J. P.; Brudvig, G. W., Water-splitting chemistry of photosystem II. *Chem. Rev.* **2006,** *106*, 4455-4483.
(73) Pantazis, D. A.; Orio, M.; Petrenko, T.; Zein, S.; Bill, E.; Lubitz, W.; Messinger, J.; Neese, F., A new quantum chemical approach to the magnetic properties of oligonuclear transition-metal complexes: application to a model for the tetranuclear manganese cluster of photosystem II. *Chem.*

Eur. J. **2009,** *15*, 5108-5123.
(74) Peloquin, J. M.; Campbell, K. A.; Randall, D. W.; Evanchik, M. A.; Pecoraro, V. L.; Armstrong, W. H.; Britt, R. D., ^{55}Mn ENDOR of the S_2-state photosystem II: implications on the structure of the tetranuclear Mn cluster. *J. Am. Chem. Soc.* **2000,** *122*, 10926-10942.
(75) Cox, N.; Rapatskiy, L.; Su, J.-H.; Pantazis, D. A.; Sugiura, M.; Kulik, L.; Dorlet, P.; Rutherford, A. W.; Neese, F.; Boussac, A.; Lubitz, W.; Messinger, J., Effect of Ca^{2+}/Sr^{2+} substitution on the electronic structure of the oxygen-evolving complex of photosystem II: a combined multifrequency EPR, ^{55}Mn-ENDOR, and DFT study of the S_2 state. *J. Am. Chem. Soc.* **2011,** *133*, 3635-3648.
(76) Kulik, L. V.; Epel, B.; Lubitz, W.; Messinger, J., ^{55}Mn Pulse ENDOR at 34 GHz of the S_0 and S_2 States of the oxygen-evolving complex in photosystem II. *J. Am. Chem. Soc.* **2005,** *127*, 2392-2393.
(77) Cheah, M. H.; Zhang, M.; Shevela, D.; Mamedov, F.; Zouni, A.; Messinger, J., Assessment of the manganese cluster's oxidation state via photoactivation of photosystem II microcrystals. *Proc. Natl. Acad. Sci. U.S.A.* **2020,** *117*, 141-145.
(78) Pushkar, Y.; Ravari, A. K.; Jensen, S. C.; Palenik, M., Early binding of substrate oxygen is responsible for a spectroscopically distinct S_2 state in photosystem II. *J. Phys. Chem. Lett.* **2019,** *10*, 5284-5291.
(79) Corry, T. A.; O'Malley, P. J., Proton isomers rationalize the high- and low-spin forms of the S_2 state intermediate in the water-oxidizing reaction of photosystem II. *J. Phys. Chem. Lett.* **2019,** *10*, 5226-5230.
(80) Taguchi, S.; Noguchi, T.; Mino, H., Molecular structure of the S_2 state with a g = 5 signal in the oxygen evolving complex of photosystem II. *J. Phys. Chem. B* **2020,** *124*, 5531-5537.
(81) Mino, H.; Nagashima, H., Orientation of ligand field for dangling manganese in photosynthetic oxygen-evolving complex of photosystem II. *J. Phys. Chem. B* **2020,** *124*, 128-133.
(82) Boussac, A.; Girerd, J.-J.; Rutherford, A. W., Conversion of the spin state of the manganese complex in photosystem II induced by near-infrared light. *Biochemistry* **1996,** *35*, 6984-6989.
(83) Boussac, A.; Kuhl, H.; Un, S.; Rögner, M.; Rutherford, A. W., Effect of near-infrared light on the S_2-state of the manganese complex of photosystem II from Synechococcus elongatus. *Biochemistry* **1998,** *37*, 8995-9000.
(84) Boussac, A.; Un, S.; Horner, O.; Rutherford, A. W., High-spin states ($S \geq 5/2$) of the photosystem II manganese complex. *Biochemistry* **1998,** *37*, 4001-4007.
(85) Guo, Y.; Li, H.; He, L.-L.; Zhao, D.-X.; Gong, L.-D.; Yang, Z.-Z., Theoretical reflections on the structural polymorphism of the oxygen-evolving complex in the S_2 state and the correlations to substrate water exchange and water oxidation mechanism in photosynthesis. *Biochim. Biophys. Acta, Bioenerg.* **2017,** *1858*, 833-846.
(86) de Lichtenberg, C.; Messinger, J., Substrate water exchange in the S_2 state of photosystem II is dependent on the conformation of the Mn_4Ca cluster. *Phys. Chem. Chem. Phys.* **2020,** *22*, 12894-12908.
(87) Allgöwer, F.; Gamiz-Hernandez, A. P.; Rutherford, A. W.; Kaila, V. R. I., Molecular principles of redox-coupled protonation dynamics in photosystem II. *J. Am. Chem. Soc.* **2022,** *144*, 7171–7180.
(88) Siegbahn, P. E. M.; Blomberg, M. R. A.; Chen, S.-L., Significant van der waals effects in transition metal complexes. *J. Chem. Theory Comput.* **2010,** *6*, 2040-2044.
(89) Siegbahn, P. E. M., A quantum chemical approach for the mechanisms of redox-active

metalloenzymes. *RSC Adv.* **2021,** *11*, 3495-3508.
(90) Siegbahn, P. E. M., Nucleophilic water attack is not a possible mechanism for O-O bond formation in photosystem II. *Proc. Natl. Acad. Sci. U.S.A.* **2017,** *114*, 4966-4968.
(91) Li, X.; Siegbahn, P. E. M., Water oxidation for simplified models of the oxygen-evolving complex in photosystem II. *Chem. Eur. J.* **2015,** *21*, 1-8.
(92) Siegbahn, P. E. M., Water oxidation energy diagrams for photosystem II for different protonation states, and the effect of removing calcium. *Phys. Chem. Chem. Phys.* **2014,** *16*, 11893-11900.
(93) Siegbahn, P. E. M.; Blomberg, M. R. A., Quantum chemical studies of proton-coupled electron transfer in metalloenzymes. *Chem. Rev.* **2010,** *110*, 7040-7061.
(94) Blomberg, M. R. A.; Siegbahn, P. E. M., Quantum chemistry as a tool in bioenergetics. *Biochim. Biophys. Acta, Bioenerg.* **2010,** *1797*, 129-142.
(95) Siegbahn, P. E. M., A structure-consistent mechanism for dioxygen formation in photosystem II. *Chem. Eur. J.* **2008,** *14*, 8290-8302.
(96) Siegbahn, P. E. M., Theoretical studies of O-O bond formation in photosystem II. *Inorg. Chem.* **2008,** *47*, 1779-1786.
(97) Siegbahn, P. E. M., O-O bond formation in the S_4 state of the oxygen-evolving complex in photosystem II. *Chem. Eur. J.* **2006,** *12*, 9217-9227.

VIII. Optimized Cartesian coordinates

Because of the high similarity, only the coordinates for the highest spin states are given here; those for other spin states can be given upon request.

$S_3^{A,W}Y_z$ (W1=H_2O, W2=OH^-, Ox=OH^-)

Mn	-0.01916200	-2.51865400	-1.05213400
Mn	0.72391200	-1.77569100	1.52249700
Mn	2.09972300	0.12329900	-0.08314700
Mn	1.72024300	2.45710900	-1.57487700
Ca	-1.55508400	0.36887200	0.11113000
O	-0.76417400	-1.95550500	0.53307500
O	0.87492500	0.00028800	1.28786600
O	1.58600500	-1.87902500	-0.19459300
O	2.68295000	1.78318600	0.01384300
O	-0.44072600	-1.07912700	-1.97852100
O	2.87327800	3.92109400	-1.84977300
O	0.67105000	2.80317700	-2.97975600
O	-2.71810600	1.63346900	-1.50923800
O	-3.67529300	0.01723100	1.20554500
H	7.62935600	5.30327200	-0.45282500
C	7.78459500	4.28688600	-0.07228600

H	8.82357400	4.20625000	0.27028300
C	7.50526300	3.23913000	-1.15734500
C	6.13325100	3.34132000	-1.79337100
O	5.21943700	3.85619300	-0.97340800
O	5.90047800	2.96405400	-2.93891100
C	-7.58415700	7.22422200	1.38498200
C	-7.23495500	6.65811200	-0.00093400
C	-6.90071700	5.18307100	0.04154500
C	-7.86143600	4.25322600	0.46341500
C	-5.63160900	4.70490000	-0.31504500
C	-7.57429700	2.89135800	0.53820900
C	-5.32413300	3.34408800	-0.25373600
C	-6.30041100	2.44158500	0.17476900
O	-5.98721600	1.09758100	0.20483700
H	2.06369000	8.19514200	1.43322100
C	2.49187200	7.86933900	0.47790300
C	1.55602100	7.01073400	-0.35260500
O	1.91421700	6.52741700	-1.42673500
H	3.41294900	7.30616200	0.66561300
N	0.27869200	6.85841100	0.12712900
C	-0.74203400	6.11942900	-0.60906700
C	-1.83357300	7.00853500	-1.21572400
O	-2.77490700	6.48746900	-1.82464300
C	-1.29097100	4.90984300	0.16789900
C	-0.44499200	3.62233500	0.10768100
O	-0.85880800	2.61437800	0.69773500
O	0.65263800	3.70974700	-0.57275600
N	-1.62722000	8.34007500	-1.10166600
C	-2.40184500	9.34923700	-1.80327500
H	-1.80143000	9.82938700	-2.58671100
H	-5.93397500	-3.78923800	-3.98605200
C	-6.03749300	-4.13233000	-2.94609800
C	-7.49442800	-4.16819800	-2.54100800
O	-7.87556900	-4.77426000	-1.53004600
C	-5.17909300	-3.26117300	-2.03271200
C	-3.74926200	-3.32166800	-2.56600000
C	-2.73452900	-2.63486000	-1.69033400
O	-2.97547100	-1.56354400	-1.12142000
O	-1.60660100	-3.29548100	-1.63051700
N	-8.34380200	-3.47677300	-3.34969900
C	-9.76392500	-3.36167600	-3.07409300
H	-10.32743700	-3.50655600	-4.00396600
C	-10.17061200	-2.01667000	-2.43424700
C	-9.31657000	-1.61081200	-1.27424000

C	-8.82486300	-0.38378900	-0.89487400
N	-8.83600000	-2.49954600	-0.32324000
C	-8.06217900	-1.80250600	0.54839800
N	-8.05257500	-0.51314600	0.24420700
H	3.53550400	-4.57633700	-6.85322000
C	3.66147000	-3.50483100	-6.66032000
C	4.69972500	-3.35506900	-5.55676100
O	4.78343200	-4.17703000	-4.62773600
C	2.31293800	-2.87067000	-6.20895000
C	1.88098600	-3.18629100	-4.80427500
C	1.21694500	-2.42016700	-3.87652300
N	2.03703000	-4.42990600	-4.19986600
C	1.50999900	-4.37857900	-2.95254800
N	0.99608100	-3.17458500	-2.73632800
N	5.45215100	-2.23263000	-5.60574200
C	6.31743500	-1.83212600	-4.50115400
H	6.88050500	-2.71252500	-4.17516200
C	5.51517500	-1.26410200	-3.29654700
C	4.67740400	-0.01294000	-3.59684300
C	3.65428500	0.38566000	-2.52722900
O	3.45390600	-0.36357300	-1.52195500
O	3.03741600	1.47572200	-2.74740400
H	7.65222100	-5.86392100	0.95120700
C	7.08191700	-5.80084600	1.88723600
H	7.50168900	-6.53546100	2.58292700
C	5.58236500	-6.08671500	1.66324400
C	4.87895600	-5.13217900	0.74569300
C	3.84263400	-4.25895000	0.96376900
N	5.17737600	-5.00048800	-0.60997300
C	4.36205600	-4.09057300	-1.18180000
N	3.54945000	-3.63079800	-0.23383600
H	-0.57245000	-7.54884000	2.76359100
C	-0.26968100	-6.49361200	2.71521100
C	-1.36962500	-5.66541700	3.38878000
O	-2.33757900	-5.23662100	2.75927600
C	-0.08752800	-6.09925600	1.24600300
C	0.35081100	-4.66624300	0.93717200
O	0.64527500	-3.86069600	1.86842800
O	0.38541600	-4.38049700	-0.30427700
N	-1.23570100	-5.47404800	4.73455600
C	-2.35219800	-5.02470900	5.56522600
C	-2.76859700	-3.54804000	5.42899800
O	-3.87590300	-3.19503300	5.82184400
N	-1.83976200	-2.70500700	4.91437700

C	-2.19079100	-1.33818600	4.53827700
C	-1.25514300	-0.88341400	3.40296300
O	-1.45405300	0.18854800	2.82845500
O	-0.25651100	-1.69719600	3.14615900
O	-1.89214000	3.78509400	-2.81405400
O	3.81004400	4.26147100	-4.41839700
O	2.11084800	2.35378100	-5.20874300
O	5.02859400	2.26405100	1.29694800
O	-4.87946200	0.14249900	-2.18374100
O	-3.17783000	-2.67732200	1.73922700
H	5.68637700	-2.21655200	5.56413100
C	5.52194900	-1.13404700	5.47418400
H	5.30287300	-0.74571500	6.47723000
C	4.38102900	-0.83266800	4.50190000
C	4.68836300	-1.34922100	3.08485400
C	3.48474800	-1.21808800	2.18047500
O	2.44834900	-1.89375000	2.50147200
O	3.57441500	-0.46059200	1.17353400
H	4.94479800	3.81103900	8.70119900
C	3.93667600	4.22681200	8.83092400
C	3.06935900	3.97393400	7.59130400
C	2.90275900	2.47896300	7.27721600
C	1.94167400	2.17888900	6.12322300
N	2.46669400	2.68383800	4.85556800
C	1.94678300	2.39604500	3.64171500
N	0.81527900	1.70510500	3.53668500
N	2.57748200	2.83723100	2.54387100
O	6.17477600	0.02533000	-0.06598600
H	-8.45246300	6.71046700	1.81619300
H	-6.74509800	7.09332600	2.08080600
H	-7.82138900	8.29495900	1.32878000
H	-6.38453400	7.21083800	-0.42340700
H	-8.08314900	6.83059400	-0.68133400
H	-8.85738500	4.59765100	0.74026100
H	-8.32564000	2.18042400	0.87537000
H	-4.34966800	2.96529700	-0.55621000
H	-4.87342100	5.40342100	-0.66999600
H	-6.81873700	0.53103600	0.38501700
H	-8.97131200	0.58037900	-1.36585800
H	-7.52411300	-2.26586800	1.36539900
H	-1.01904500	-3.08888400	4.45594200
H	-0.43884100	-5.89470400	5.19592700
H	5.31455700	-1.58809600	-6.37417100
H	-7.95017100	-2.93222500	-4.10717400

H	-10.01105300	-4.19405500	-2.40749000
H	-11.22982900	-2.09597600	-2.14292200
H	-10.11829400	-1.21593400	-3.18375500
H	-3.69726800	-2.82799800	-3.54985500
H	-5.54253200	-2.22709500	-2.03418800
H	-5.67405800	-5.16793800	-2.90987600
H	-3.43228500	-4.36043400	-2.71507700
H	-5.22575400	-3.63441900	-1.00192900
H	7.23185700	-4.79865700	2.30557600
H	3.27785100	-4.02730500	1.85596000
H	4.37551500	-3.82617400	-2.23212000
H	0.84548700	-1.40820900	-3.94794500
H	1.49057400	-5.19350000	-2.24421700
H	3.97821800	-3.03519900	-7.59895100
H	7.02908500	-1.09247500	-4.88356600
H	-0.20397200	5.72334500	-1.48016700
H	0.07340800	7.16067800	1.07282100
H	2.76517300	8.75289600	-0.11100500
H	-0.76259300	8.61803000	-0.65416000
H	7.13593700	4.15125500	0.79971700
H	1.53626200	-3.18502700	-6.92093300
H	2.37587800	-1.77846000	-6.29075000
H	0.67318500	-6.39786200	3.27033600
H	-3.20230000	-1.33436900	4.11540100
C	-2.13241600	-0.36117300	5.71787700
H	-2.40734800	0.64472300	5.37976900
H	-2.84158600	-0.68512300	6.48607100
H	-1.12478300	-0.34064300	6.15711200
H	-2.08793400	-5.20785200	6.61221900
H	-3.25196600	-5.60726500	5.33687200
H	0.62900400	-6.77318100	0.75654400
H	-1.03980500	-6.22532500	0.71677700
H	-2.26577000	4.63203200	-0.24765700
H	-1.46487200	5.15104500	1.22564200
H	6.46299700	-0.67989400	5.13660600
H	3.45085900	-1.30007800	4.85201300
H	4.94799300	-2.41701000	3.14206900
H	4.19735600	0.25134500	4.45540000
H	5.52740200	-0.81515500	2.62725200
H	8.23884500	3.29604300	-1.96963800
H	7.57426200	2.21978100	-0.74660000
H	5.05454500	-6.05478900	2.62444600
H	5.45721000	-7.11098100	1.28077300
H	2.77363200	-2.88841000	-0.32878000

H	4.03847200	5.30015300	9.02964800
H	3.51571000	4.50027200	6.73180500
H	2.51168400	1.96841600	8.16808500
H	1.79902600	1.09434200	6.02219400
H	3.49420800	3.75964500	9.72053500
H	2.07427100	4.42066600	7.73739100
H	3.88061900	2.02287400	7.06017300
H	3.16121500	3.41964700	4.88685500
H	0.95863900	2.62943300	6.33947000
H	3.60466000	2.85580900	2.51739200
H	0.18724200	1.57030900	4.31621000
H	2.21646200	2.54451800	1.62328200
H	0.56202700	1.23735300	2.65689300
H	4.84107700	-2.04954100	-2.94505100
H	5.30804400	0.87349300	-3.74535200
H	6.20968100	-1.04349400	-2.47526700
H	4.09834400	-0.14847900	-4.52408200
H	2.70277700	-5.12542400	-4.52100100
H	1.13309900	2.69189700	-3.87204700
H	4.24804100	3.86434000	-1.38124500
H	5.25701400	3.00695100	0.69436200
H	4.23868700	1.89343100	0.82178000
H	5.27631300	-0.31137600	-0.22959000
H	5.98969000	0.78824600	0.52684700
H	-2.76269100	10.11440300	-1.10556100
H	-3.25766500	8.85021200	-2.26557500
H	-5.39420500	0.48875900	-1.41931700
H	-4.31124400	-0.55184300	-1.77913800
H	-3.80569200	-0.91969800	1.46942300
H	-2.45031000	2.44833900	-2.01976700
H	-2.11643900	4.71350000	-2.60514000
H	-0.91779200	3.67055500	-2.92393000
H	-4.53514100	0.38575500	0.90184700
H	-3.50324400	1.23022600	-1.96423000
H	2.57055700	1.72538600	-4.62221200
H	2.72538200	3.14404500	-5.12748400
H	3.32662900	4.30736200	-3.55938100
H	4.61403600	3.77656300	-4.13493400
H	-3.09413300	-3.50034900	2.25964400
H	-2.33617200	-2.61313600	1.23965900
H	-8.79187900	-3.51533700	-0.45514600
H	5.90800000	-5.49818300	-1.10597900
O	0.87416800	0.82065400	-1.19040400
H	0.23444900	-0.32410200	-1.89221800

H	2.40934900	4.76119200	-1.62649700

$S_3^{TS}Y_z$ (W1=H_2O, W2=OH^-, Ox=OH^-)

Mn	0.20674200	-2.54358800	-1.05666500
Mn	0.71343200	-1.91233500	1.55331200
Mn	1.90461300	-0.05605000	-0.20007700
Mn	1.58835600	2.63761600	-1.56816600
Ca	-1.51528100	0.32433800	0.10275400
O	-0.70587400	-2.12287200	0.50919500
O	0.75049700	-0.11752500	1.17975700
O	1.74614500	-2.09193600	0.00722700
O	2.40454900	1.65935800	-0.11445000
O	0.31675200	-0.94596700	-1.59399000
O	2.89002500	3.98992200	-1.72702400
O	0.68006800	3.17389800	-3.02854800
O	-2.70028200	1.63675200	-1.48566000
O	-3.66474700	-0.12861500	1.09112600
H	7.52208200	5.47534500	-0.42529900
C	7.69364700	4.49083800	0.02559500
H	8.71740400	4.47085600	0.41963300
C	7.49817800	3.37053500	-1.00349700
C	6.13811800	3.40145300	-1.66791500
O	5.19740700	3.89327700	-0.86559000
O	5.93581900	3.01024700	-2.81442200
C	-7.75326400	7.06238700	1.34072200
C	-7.35932100	6.50152500	-0.03518700
C	-7.00338300	5.03238500	0.02124500
C	-7.96251700	4.08888700	0.41534300
C	-5.71544300	4.57376900	-0.28960600
C	-7.65571300	2.73267300	0.50769700
C	-5.38785600	3.21862100	-0.20984300
C	-6.36283800	2.30186400	0.19052900
O	-6.03079900	0.96353800	0.24005000
H	1.85995400	8.26868200	1.47066500
C	2.31245300	7.94663400	0.52530900
C	1.41450000	7.05637000	-0.31382000
O	1.80578400	6.56718100	-1.37361500
H	3.24557700	7.41114600	0.73352800
N	0.13231400	6.88675500	0.14443000
C	-0.86887900	6.11991300	-0.59010800
C	-1.97764700	6.98604000	-1.19927400
O	-2.91949200	6.44825900	-1.79365900
C	-1.39732700	4.91448000	0.20340300
C	-0.47239600	3.68462800	0.21495700

O	-0.80424000	2.67794700	0.85099400
O	0.62136600	3.84462900	-0.47907400
N	-1.79153000	8.32164600	-1.10058400
C	-2.59400600	9.30883400	-1.80133200
H	-2.01545200	9.78794100	-2.60171100
H	-5.81329000	-3.98113600	-4.03117300
C	-5.89746600	-4.25576400	-2.96931500
C	-7.34943300	-4.29663600	-2.55039800
O	-7.72017300	-4.88936900	-1.52822900
C	-5.01649500	-3.30437000	-2.12939700
C	-3.58145200	-3.34865100	-2.67974100
C	-2.52385200	-2.56802500	-1.90667400
O	-2.76255400	-1.47440200	-1.36793200
O	-1.36224900	-3.15350500	-1.91749900
N	-8.21129700	-3.64487400	-3.37980200
C	-9.63979500	-3.57403100	-3.13178800
H	-10.17919900	-3.74458100	-4.07147000
C	-10.10149400	-2.23713600	-2.51138400
C	-9.28760500	-1.79958300	-1.33390200
C	-8.83213800	-0.55885800	-0.95341600
N	-8.81450000	-2.66934700	-0.36177600
C	-8.07782200	-1.94849400	0.52212800
N	-8.08709800	-0.66195500	0.20664800
H	3.71788200	-4.47605400	-6.77632300
C	3.81737500	-3.39917700	-6.59520200
C	4.81124600	-3.21674400	-5.45419400
O	4.83409600	-3.99074100	-4.48512500
C	2.44322400	-2.78752100	-6.20217100
C	1.97800200	-3.14616500	-4.82505700
C	1.57057500	-2.36178200	-3.77492100
N	1.87833000	-4.45302500	-4.35738000
C	1.44481700	-4.42963100	-3.07486700
N	1.24515300	-3.17219700	-2.70290100
N	5.59844300	-2.11857000	-5.53587400
C	6.41298600	-1.66295800	-4.41284300
H	6.99309100	-2.51301000	-4.03877200
C	5.54360700	-1.09421900	-3.24874000
C	4.61258800	0.08132200	-3.60631400
C	3.50820700	0.40969500	-2.58559700
O	3.29733000	-0.39695400	-1.62201400
O	2.83330700	1.46514500	-2.80225500
H	7.86892200	-5.45331300	1.11868900
C	7.21315300	-5.60998600	1.98458800
H	7.69895100	-6.33237800	2.65017200

C	5.82685900	-6.12831800	1.55745500
C	5.07753800	-5.19510800	0.65879300
C	4.05889200	-4.31168900	0.91086500
N	5.33571300	-5.06283300	-0.70489100
C	4.50799200	-4.14786000	-1.25371100
N	3.73440300	-3.68606200	-0.27618100
H	-0.37983600	-7.53962900	2.87544900
C	-0.12712700	-6.47709700	2.74581300
C	-1.25002200	-5.66322000	3.40213000
O	-2.22470200	-5.26135200	2.76649200
C	0.01692900	-6.17237100	1.26266400
C	0.44108800	-4.75237100	0.90998900
O	0.73889400	-3.94284000	1.85302600
O	0.45446400	-4.48955800	-0.32667900
N	-1.13094800	-5.46252600	4.75069600
C	-2.26995400	-5.05706700	5.57582700
C	-2.74125000	-3.59591700	5.42737800
O	-3.88150500	-3.28694800	5.75545300
N	-1.81163200	-2.71397600	4.97788000
C	-2.18687900	-1.36813000	4.54821100
C	-1.28488300	-0.96139700	3.37757000
O	-1.49498000	0.09080000	2.76644500
O	-0.29800100	-1.78555900	3.14370200
O	-1.97240900	3.82026900	-2.79951900
O	4.00880200	4.56545400	-4.21442700
O	2.24221800	2.79943500	-5.17097800
O	4.75541500	2.21532200	1.24577100
O	-4.85377600	0.13508700	-2.17243700
O	-3.15034300	-2.79353300	1.61144600
H	6.01385400	-1.94488500	5.40599500
C	5.50996300	-0.98024700	5.55435200
H	5.20986600	-0.91457300	6.60735300
C	4.27839300	-0.84935100	4.63180300
C	4.60650600	-0.90730500	3.12435400
C	3.36918400	-0.99567000	2.22894100
O	2.42035200	-1.74574800	2.61381300
O	3.39877700	-0.33078000	1.13881600
H	4.78817800	3.94389400	8.82941300
C	3.76702500	4.34262600	8.89331300
C	2.97909900	4.06670000	7.60008900
C	2.85664900	2.56673200	7.27901900
C	1.88044200	2.21776000	6.14809200
N	2.36808300	2.64364900	4.83380500
C	1.78514400	2.29433700	3.66178400

N	0.66546800	1.57300700	3.66175800
N	2.33769300	2.67892600	2.50638800
O	5.98726600	0.05380800	-0.16577600
H	-8.62397200	6.53462300	1.74955200
H	-6.93042600	6.94496500	2.05799900
H	-8.00614800	8.12923100	1.27816200
H	-6.50685500	7.06690000	-0.43626400
H	-8.19223400	6.65931900	-0.73761200
H	-8.97273600	4.41829300	0.65655000
H	-8.40619300	2.01096300	0.82283400
H	-4.39724300	2.85728800	-0.47884300
H	-4.95803900	5.28331100	-0.62368300
H	-6.86286100	0.38764000	0.38572600
H	-8.98664000	0.39791000	-1.43667900
H	-7.55034900	-2.39363200	1.35600300
H	-0.96690000	-3.08096600	4.54943600
H	-0.33223000	-5.87369500	5.21779900
H	5.49809000	-1.50938200	-6.33803000
H	-7.82626300	-3.11329300	-4.15066800
H	-9.87369500	-4.40838800	-2.46260800
H	-11.16467600	-2.34544900	-2.24482300
H	-10.05403900	-1.44047000	-3.26548800
H	-3.57253200	-2.94181300	-3.70413500
H	-5.40785200	-2.28115600	-2.18184900
H	-5.51397900	-5.27978000	-2.87006000
H	-3.23378200	-4.38592100	-2.75510000
H	-5.03208500	-3.61361800	-1.07642000
H	7.12429200	-4.65416600	2.51437200
H	3.52162000	-4.07605000	1.81960700
H	4.48987100	-3.88182400	-2.30686500
H	1.44299800	-1.29189800	-3.70224900
H	1.24951700	-5.29441400	-2.45732100
H	4.16292500	-2.93963600	-7.52910400
H	7.11124400	-0.90934600	-4.79175400
H	-0.32248300	5.72241000	-1.45483000
H	-0.09487600	7.20722800	1.07908200
H	2.57191500	8.83277800	-0.06610500
H	-0.92570900	8.61903000	-0.66828300
H	7.00422900	4.38396100	0.86955600
H	1.69999000	-3.08935100	-6.95391800
H	2.50108400	-1.69322300	-6.25005300
H	0.81879400	-6.30303700	3.27600300
H	-3.21103200	-1.39792500	4.15592200
C	-2.11414300	-0.33689900	5.68025900

H	-2.40740600	0.65030500	5.30369600
H	-2.80171700	-0.63005900	6.48007200
H	-1.09737500	-0.28878900	6.09634900
H	-2.00115900	-5.22489000	6.62436600
H	-3.14617900	-5.67388100	5.34607300
H	0.73297900	-6.86197800	0.79527000
H	-0.94352700	-6.32813000	0.75689200
H	-2.33188600	4.56939200	-0.25273100
H	-1.63761900	5.17967600	1.24202400
H	6.24378900	-0.18714900	5.35878200
H	3.55708800	-1.64370300	4.86183000
H	5.20154600	-1.80996200	2.90845700
H	3.76728400	0.10338700	4.83817300
H	5.20260100	-0.04914000	2.79846700
H	8.25120500	3.41057600	-1.79853800
H	7.59319200	2.38033500	-0.53127200
H	5.20762700	-6.29741600	2.44715500
H	5.92841400	-7.10634500	1.06487100
H	2.93562100	-2.96646500	-0.31040100
H	3.83692400	5.41912800	9.08793100
H	3.46024100	4.59871200	6.76344800
H	2.49465300	2.04419600	8.17566100
H	1.73404500	1.12973200	6.10992200
H	3.27668200	3.87317300	9.75607600
H	1.96856900	4.49315500	7.69179600
H	3.84460600	2.13896400	7.05103800
H	3.05573100	3.38572500	4.79465000
H	0.90194700	2.68036000	6.36070300
H	3.36145800	2.73457300	2.40723800
H	0.06544600	1.51111200	4.47212800
H	1.91222000	2.35756500	1.62598000
H	0.30578400	1.15786400	2.79806900
H	4.92677300	-1.91250200	-2.86755200
H	5.17431500	1.01332300	-3.74721700
H	6.20682500	-0.78539900	-2.43113700
H	4.08316600	-0.11782600	-4.55196400
H	2.22322800	-5.27254400	-4.84037700
H	1.21946000	3.15089300	-3.88332100
H	4.22058200	3.90445700	-1.28701700
H	5.03416200	2.96632300	0.67160800
H	3.99467100	1.84060600	0.72869000
H	5.10654000	-0.29019700	-0.39682200
H	5.75867300	0.77923500	0.45579600
H	-2.95424400	10.07760500	-1.10726800

H	-3.44980900	8.79045000	-2.24172300
H	-5.37501200	0.39895400	-1.38078500
H	-4.23431600	-0.55089000	-1.83288300
H	-3.77295700	-1.06389300	1.37413600
H	-2.46077500	2.47057000	-1.98049200
H	-2.25919900	4.73171900	-2.58930700
H	-0.99486200	3.78784600	-2.94446200
H	-4.54269900	0.24583300	0.85788100
H	-3.51049000	1.25236600	-1.91324900
H	2.61831400	2.10457000	-4.59660000
H	2.90814600	3.53544000	-5.03965200
H	3.47422700	4.53368000	-3.38805100
H	4.77100200	4.00586700	-3.95342300
H	-3.06119000	-3.58266900	2.18087600
H	-2.28524700	-2.72898700	1.14976700
H	-8.73210000	-3.68318900	-0.49063200
H	6.04179200	-5.57164500	-1.22439100
O	0.31901500	1.32986400	-1.29592000
H	0.02592300	1.04588700	-2.18320100
H	2.46082800	4.85673300	-1.53206400

$S_3^{B,W}Y_Z$ (W1=H_2O, W2=OH^-, Ox=OH^-)

Mn	0.39581600	-2.46653600	-0.99791600
Mn	0.82996700	-1.88215300	1.62062100
Mn	1.95834100	-0.15872400	-0.21230600
Mn	1.30364600	2.56758000	-1.80034700
Ca	-1.44989900	0.34842100	-0.01224700
O	-0.57316400	-2.11961100	0.49132100
O	0.81700600	-0.15258400	1.21858000
O	1.90930200	-2.13508100	0.08057200
O	1.99860100	1.58740400	-0.30412000
O	0.61349500	-0.74016500	-1.37585000
O	2.72795500	3.86509100	-1.78290000
O	0.58374700	3.32082300	-3.28815800
O	-2.95453300	1.71749100	-1.31888100
O	-3.57585200	-0.27413500	0.97390200
H	7.40711800	5.80637500	-0.47906800
C	7.51187100	4.88130400	0.10088500
H	8.50108200	4.88761500	0.57484900
C	7.35993200	3.64429500	-0.79488800
C	6.03751600	3.55817900	-1.52833900
O	5.01174600	4.03867800	-0.82853600
O	5.93204100	3.08318400	-2.65737500
C	-8.05310600	6.72917100	1.22609800

C	-7.84844800	6.08089400	-0.15215700
C	-7.39572800	4.64595200	-0.02312200
C	-8.30718500	3.64604000	0.34165900
C	-6.05182600	4.28568900	-0.19256500
C	-7.90159700	2.32593900	0.53644700
C	-5.62441200	2.97141800	-0.00337700
C	-6.55434300	1.99317900	0.35791300
O	-6.11509100	0.69359300	0.50749900
H	1.49863800	8.36331700	1.47497400
C	1.96902800	8.08230100	0.52515400
C	1.15154600	7.09849200	-0.29714900
O	1.60772000	6.56108200	-1.30252300
H	2.95512200	7.64821800	0.72065900
N	-0.14944400	6.91684700	0.11810700
C	-1.10972300	6.10488200	-0.62073100
C	-2.25885300	6.91639300	-1.23089600
O	-3.18646300	6.33544600	-1.80778900
C	-1.57810100	4.86382100	0.15803700
C	-0.61051900	3.67354700	0.09605200
O	-0.81533600	2.66891300	0.80585800
O	0.36276000	3.83762600	-0.73655200
N	-2.12158600	8.25983700	-1.15648900
C	-2.96800900	9.20559600	-1.86357500
H	-2.41700700	9.69173700	-2.67906000
H	-5.53243000	-4.25691300	-4.08982300
C	-5.61768600	-4.50260600	-3.02099100
C	-7.07132800	-4.61764800	-2.62247400
O	-7.42587600	-5.24028800	-1.61276500
C	-4.79696700	-3.47138600	-2.19823700
C	-3.33486100	-3.47359300	-2.68704100
C	-2.33721000	-2.55518300	-1.97429100
O	-2.65426600	-1.46873400	-1.46669900
O	-1.11645100	-3.01183600	-2.01140600
N	-7.95228000	-4.00485900	-3.46123700
C	-9.38568800	-3.99772900	-3.22963200
H	-9.90650200	-4.17199000	-4.17887400
C	-9.90423900	-2.68866800	-2.59238200
C	-9.13196300	-2.24149600	-1.38915000
C	-8.74055500	-0.99049700	-0.97020300
N	-8.65308500	-3.11037000	-0.41901000
C	-7.97565200	-2.37776200	0.50201200
N	-8.02888100	-1.08672500	0.21146800
H	4.01150000	-4.28251600	-6.70571900
C	4.08852700	-3.20214800	-6.53619500

C	5.07159900	-2.98223500	-5.39173900
O	5.12500600	-3.76018000	-4.42603900
C	2.70027500	-2.60950900	-6.15314100
C	2.23332300	-2.96733100	-4.77454200
C	1.76560100	-2.19572100	-3.73760200
N	2.20442200	-4.27238300	-4.29328200
C	1.75653500	-4.26187400	-3.01630500
N	1.48133400	-3.01465400	-2.65859400
N	5.80508500	-1.84781500	-5.46382800
C	6.57384700	-1.33934800	-4.32995100
H	7.20496200	-2.14895200	-3.94827400
C	5.65688400	-0.82095600	-3.18461300
C	4.62614200	0.24808700	-3.58800100
C	3.46382600	0.45419500	-2.60675900
O	3.41252900	-0.29626400	-1.57509000
O	2.60961300	1.33997700	-2.92176900
H	8.15489300	-4.94314700	1.27058100
C	7.48240500	-5.22485200	2.09062400
H	8.02721700	-5.90184700	2.75830800
C	6.20616100	-5.90742300	1.56402400
C	5.39694300	-5.03633500	0.65620600
C	4.31144200	-4.23263100	0.89502300
N	5.67829800	-4.86452600	-0.69832900
C	4.80213000	-4.00295000	-1.25732600
N	3.97367200	-3.61491200	-0.29199900
H	0.00873600	-7.50601200	2.93881300
C	0.18237500	-6.43314800	2.76916300
C	-0.98732600	-5.67634500	3.41167300
O	-1.97761800	-5.33101800	2.76659800
C	0.28245400	-6.16810300	1.27128500
C	0.60375800	-4.72388500	0.90968300
O	0.87310400	-3.92451500	1.86351800
O	0.56968800	-4.44079900	-0.32896300
N	-0.89018100	-5.45848400	4.75929800
C	-2.05808000	-5.10659800	5.56877100
C	-2.59986900	-3.67156500	5.40246600
O	-3.76270600	-3.41917200	5.69680500
N	-1.70271900	-2.74339700	4.97761000
C	-2.13582500	-1.42096600	4.52889700
C	-1.24535300	-0.97654700	3.36751700
O	-1.49787000	0.06129800	2.74731900
O	-0.22829500	-1.76789900	3.15418400
O	-2.13688200	3.78172500	-2.86678200
O	3.94697600	4.38792200	-4.21590200

O	2.22754500	2.67367300	-5.33762000
O	4.06298000	2.55804500	1.25964600
O	-4.95072300	0.00292500	-1.98458600
O	-2.97182900	-2.89399000	1.60645900
H	5.85660600	-1.71442800	5.66566800
C	5.52184400	-0.66922300	5.62347100
H	5.22925700	-0.36808500	6.63717200
C	4.34255800	-0.50782600	4.64407200
C	4.68387500	-0.88437300	3.18682500
C	3.46020000	-0.93952100	2.27572600
O	2.50604200	-1.70461800	2.66500500
O	3.47874800	-0.26748600	1.20486700
H	4.57158000	4.49770900	8.73769600
C	3.49173400	4.57614700	8.92247400
C	2.67994400	4.19123900	7.67177500
C	2.94474700	2.75062900	7.19978100
C	1.93939700	2.19790500	6.18075000
N	2.09622400	2.77322800	4.83782000
C	1.27006700	2.46771400	3.80224600
N	0.05866300	1.95698200	4.05467900
N	1.62389100	2.73584300	2.54905900
O	5.92683500	0.67902200	0.12837900
H	-8.80736300	6.18195000	1.80606000
H	-7.11903000	6.71353300	1.80293500
H	-8.38441900	7.77249300	1.13501400
H	-7.10552700	6.65359400	-0.72432600
H	-8.78960300	6.13138000	-0.71925200
H	-9.35715200	3.90425900	0.47952300
H	-8.61738200	1.55907400	0.82456700
H	-4.58853400	2.68649600	-0.17232800
H	-5.32715700	5.04063800	-0.49999000
H	-6.90440300	0.04694600	0.55899600
H	-8.92118400	-0.03070700	-1.43802500
H	-7.45707100	-2.81776200	1.34412200
H	-0.83252900	-3.07175600	4.56996000
H	-0.07753900	-5.82767300	5.23757100
H	5.67483000	-1.24089900	-6.26367300
H	-7.58221700	-3.44248700	-4.21755900
H	-9.59390100	-4.85213900	-2.57757700
H	-10.96807500	-2.83799400	-2.34902700
H	-9.87022500	-1.87765700	-3.33182700
H	-3.30495400	-3.17718700	-3.74853600
H	-5.23125200	-2.46944600	-2.30525000
H	-5.17749700	-5.49867800	-2.88260300

H	-2.92216000	-4.48909800	-2.64431000
H	-4.83789700	-3.73411100	-1.13316100
H	7.23337500	-4.31380100	2.64763000
H	3.73990900	-4.04153900	1.79353800
H	4.79149300	-3.72161500	-2.30761400
H	1.57708200	-1.13253200	-3.68239200
H	1.60209400	-5.13123300	-2.39332200
H	4.42750300	-2.74494300	-7.47345500
H	7.22635300	-0.54386300	-4.70455400
H	-0.54095800	5.73839300	-1.48555400
H	-0.41800500	7.27071900	1.02945300
H	2.12421600	8.98857500	-0.07336700
H	-1.26108900	8.59452400	-0.74048800
H	6.75287900	4.89422400	0.89079100
H	1.96611200	-2.93180800	-6.90527100
H	2.73846300	-1.51496200	-6.21180000
H	1.11965000	-6.17088200	3.27653700
H	-3.15180800	-1.50813000	4.12320400
C	-2.14159900	-0.38328600	5.65802000
H	-2.48481000	0.58520300	5.27483800
H	-2.82738100	-0.71354700	6.44460600
H	-1.13761200	-0.27942200	6.09417300
H	-1.79303800	-5.25287300	6.62151300
H	-2.89938400	-5.76813800	5.33347400
H	1.04419100	-6.81459000	0.81410600
H	-0.66929300	-6.40612100	0.78218300
H	-2.51216600	4.49522500	-0.28319700
H	-1.79328900	5.09071000	1.21032600
H	6.37988600	-0.05461600	5.32093600
H	3.49939700	-1.12932900	4.97396200
H	5.12755100	-1.89312600	3.17357100
H	3.98880600	0.53447300	4.65868400
H	5.41120900	-0.19929100	2.73941000
H	8.14944800	3.59766200	-1.55333000
H	7.43426900	2.71878900	-0.20427400
H	5.56720200	-6.19556700	2.40793400
H	6.46524900	-6.84012900	1.04273500
H	3.14269900	-2.94651900	-0.32457600
H	3.27706700	5.60751500	9.22573100
H	2.88976600	4.89798100	6.85572000
H	2.89745900	2.07926400	8.06925400
H	2.03132400	1.10229700	6.12178000
H	3.24714200	3.91694600	9.76582100
H	1.60701600	4.29465900	7.89502500

H	3.96846900	2.65216200	6.80416700
H	3.01395300	3.12244200	4.58555100
H	0.92917800	2.43065500	6.54613700
H	2.61391600	2.82671200	2.26364700
H	-0.23490900	1.75921800	4.99951300
H	0.98382500	2.46506400	1.79528100
H	-0.48202900	1.50092300	3.31342500
H	5.11891500	-1.67834900	-2.77152800
H	5.09540900	1.23296600	-3.70509200
H	6.29026300	-0.42331000	-2.38093900
H	4.15982100	-0.00038300	-4.55321200
H	2.61432100	-5.07169300	-4.75911700
H	1.14495400	3.19780000	-4.11474200
H	4.06790000	3.92260000	-1.31032800
H	4.44844300	3.28492700	0.71796600
H	3.41372700	2.13765800	0.63218400
H	5.21809300	0.10705700	-0.21431700
H	5.40529400	1.29785800	0.68611300
H	-3.34737300	9.97257600	-1.17776800
H	-3.80957800	8.64903200	-2.28432900
H	-5.45105100	0.16596700	-1.15443000
H	-4.25233300	-0.63857200	-1.72313000
H	-3.63566900	-1.19071500	1.32225700
H	-2.69946600	2.47752000	-1.90672900
H	-2.48788100	4.67221800	-2.65866100
H	-1.17712200	3.85004300	-3.08892800
H	-4.47936400	0.10597700	0.92155900
H	-3.74629900	1.26920500	-1.71888000
H	2.47384100	1.96910200	-4.70733800
H	2.92531300	3.36274900	-5.13798000
H	3.37021600	4.33432300	-3.41629900
H	4.74458400	3.91670700	-3.89559600
H	-2.86037400	-3.68946700	2.16332000
H	-2.11090600	-2.79614400	1.14337600
H	-8.52209400	-4.11606000	-0.56676200
H	6.43002900	-5.31684900	-1.20621200
O	-0.16602500	1.48726900	-1.79868300
H	-0.63203000	1.72831600	-2.61984400
H	2.28905500	4.72951400	-1.61489700

$S_3^{A,W}Y_z^{\bullet}$ (W1=OH⁻, W2=OH⁻, Ox=OH⁻)

Mn	-0.1184010	-2.58953200	-1.12510700
Mn	0.55531400	-1.83175600	1.47571300
Mn	2.04959900	-0.03225600	-0.11557300
Mn	1.93861700	2.57320400	-1.60700300

Ca	-1.47889100	0.41364000	0.02392000
O	-0.90404800	-1.93311800	0.45270900
O	0.80586700	-0.06314300	1.24061500
O	1.48091100	-2.01998900	-0.15741800
O	2.65244100	1.59113800	-0.01996800
O	-0.32868100	-1.24512000	-2.10434900
O	3.27077500	3.70703600	-1.57985200
O	1.04162200	3.05359100	-3.07540000
O	-2.89390900	1.87388200	-1.26417100
O	-3.83015900	-0.18191200	0.79804700
H	7.34111800	5.02699100	-0.67848000
C	7.82185100	4.13186700	-0.26215700
H	8.85084400	4.40177000	0.00968100
C	7.81863200	2.98312700	-1.28135900
C	6.41906300	2.71169500	-1.82632400
O	5.49071200	2.56429500	-0.92795700
O	6.23201900	2.64150000	-3.05758100
C	-7.48221800	7.48262500	0.96683700
C	-6.60678200	6.86790800	-0.15063300
C	-6.40616600	5.39600800	0.06611700
C	-7.54235000	4.53663100	0.08956700
C	-5.11848100	4.84937500	0.30371100
C	-7.41611600	3.20812000	0.39587300
C	-4.96471100	3.51701500	0.62286200
C	-6.11979500	2.64956200	0.71474500
O	-6.01433000	1.42826900	1.06974600
H	2.37299200	7.83750400	1.16508600
C	2.61069700	7.85556000	0.09482200
C	1.42212800	7.49401000	-0.77808000
O	1.34457100	7.86433500	-1.95478300
H	3.41807500	7.13553400	-0.09585400
N	0.44004900	6.75766600	-0.18130500
C	-0.65740900	6.13378300	-0.93343100
C	-1.79188200	7.16667000	-1.19433700
O	-2.92243600	7.04200600	-0.70205200
C	-1.21181200	4.93660800	-0.15504800
C	-0.32718300	3.68723300	-0.05938200
O	-0.77431900	2.69054400	0.53262500
O	0.82828300	3.79982300	-0.62575900
N	-1.41175600	8.18014100	-2.00217300
C	-2.23543100	9.34360100	-2.28044700
H	-2.33229700	9.49895800	-3.36230300
H	-6.08223400	-3.82565900	-3.90863100
C	-6.17629800	-4.08861300	-2.84452100

C	-7.62942300	-4.07241500	-2.44154900
O	-8.02345600	-4.55064400	-1.36413100
C	-5.25318900	-3.19076100	-2.02333500
C	-3.87746100	-3.26955000	-2.69259200
C	-2.79099200	-2.56980400	-1.91015700
O	-2.94687900	-1.39517000	-1.53978200
O	-1.75908900	-3.32434900	-1.69744700
N	-8.47889300	-3.47281000	-3.32283200
C	-9.88330400	-3.23926600	-3.03003000
H	-10.47884100	-3.36731700	-3.93962000
C	-10.13118000	-1.81740900	-2.44997900
C	-9.17048100	-1.39471700	-1.37162600
C	-8.55430000	-0.18999800	-1.13160400
N	-8.73485400	-2.24179900	-0.35801800
C	-7.87011600	-1.59160400	0.43316800
N	-7.76854100	-0.33744100	-0.00458900
H	3.47971600	-4.93297400	-6.68029400
C	3.55379800	-3.84982900	-6.52239700
C	4.53472100	-3.63530600	-5.37872200
O	4.55004600	-4.38279600	-4.38839200
C	2.16110900	-3.26519200	-6.18028800
C	1.69100600	-3.55405900	-4.79135800
C	1.22836600	-2.70021000	-3.82642600
N	1.62539600	-4.82044200	-4.21694000
C	1.15854800	-4.69903000	-2.94876900
N	0.89843300	-3.42398800	-2.69657800
N	5.33982400	-2.55530000	-5.49607100
C	6.23652400	-2.14350000	-4.42327300
H	6.79015300	-3.02685500	-4.08557700
C	5.47636500	-1.52381200	-3.22171200
C	4.70014400	-0.24787700	-3.55882900
C	3.64758700	0.19938800	-2.54474300
O	3.35173200	-0.54508400	-1.55498000
O	3.10774000	1.31369200	-2.81078100
H	7.50585000	-5.84558300	1.24151700
C	6.87554000	-5.83945600	2.14011600
H	7.29228700	-6.56400100	2.84834900
C	5.41182000	-6.20719000	1.81239700
C	4.70306400	-5.28276100	0.86643300
C	3.66492900	-4.40600800	1.06238200
N	4.98890400	-5.19466000	-0.49609900
C	4.16318500	-4.30680200	-1.09144900
N	3.35865900	-3.81963900	-0.15192400
H	-0.77994200	-7.37301500	3.12961100

C	-0.49426000	-6.32598500	2.95216700
C	-1.58121600	-5.44585300	3.58166800
O	-2.55396400	-5.04139600	2.94253200
C	-0.36172500	-6.08779100	1.44946900
C	0.10432700	-4.69923400	1.01330300
O	0.40137700	-3.84020400	1.90782600
O	0.15547900	-4.50273000	-0.23129200
N	-1.44274900	-5.18569700	4.91722600
C	-2.55718500	-4.68422500	5.72102100
C	-2.96543300	-3.21790800	5.47883800
O	-4.09885000	-2.84461800	5.76786600
N	-1.99466400	-2.39919300	5.00028500
C	-2.30492200	-1.05154800	4.53155800
C	-1.44349700	-0.75031800	3.28806900
O	-1.70075200	0.24550100	2.59632800
O	-0.47337400	-1.59170300	3.09396300
O	-1.58592200	3.38108100	-3.04965500
O	4.31639800	4.42141600	-4.05587700
O	2.61533200	2.70057300	-5.15335200
O	5.63961000	2.52273100	1.89815000
O	-5.10500100	0.24365700	-1.61869900
O	-3.31152600	-2.73110500	1.42330900
H	5.49190700	-2.04852200	5.75767600
C	5.40546200	-0.98264500	5.50600700
H	5.22820700	-0.43143700	6.43860000
C	4.27192900	-0.74212200	4.50214100
C	4.50837600	-1.48591500	3.17286900
C	3.32063800	-1.35730500	2.23807500
O	2.25059400	-1.95937400	2.56814500
O	3.47011600	-0.66305800	1.18458800
H	4.95777800	4.18963300	8.51768500
C	3.92711500	4.55763700	8.61051900
C	3.08593800	4.15716200	7.39502500
C	3.00129200	2.63511700	7.21021600
C	2.10941100	2.19822000	6.04652200
N	2.66789500	2.64052600	4.77350900
C	2.23523800	2.22731200	3.55733600
N	1.06281100	1.58237500	3.46141500
N	2.97410000	2.46045800	2.48467100
O	6.00464300	0.18999000	0.41278600
H	-8.47637300	7.02120700	1.00245600
H	-7.01065100	7.34905400	1.94780700
H	-7.61432900	8.55715500	0.79368200
H	-5.63358100	7.36949800	-0.20720500

H	-7.10946300	7.02553800	-1.11833200
H	-8.52416900	4.95694400	-0.12076300
H	-8.28496600	2.55545500	0.45681200
H	-3.98118500	3.08069500	0.77543600
H	-4.25158500	5.50429200	0.21175800
H	-7.13195300	0.38811400	0.42284300
H	-8.59369500	0.74383000	-1.67258100
H	-7.32274100	-2.02145000	1.26014800
H	-1.15707100	-2.81042100	4.59675300
H	-0.65104300	-5.59219400	5.39993100
H	5.25794200	-1.96984600	-6.31731400
H	-8.08292500	-3.05363600	-4.15542400
H	-10.18857800	-4.00924000	-2.31466900
H	-11.16532700	-1.77301500	-2.07683400
H	-10.06165900	-1.07520700	-3.25523700
H	-3.92359500	-2.79757600	-3.68641300
H	-5.61293300	-2.15465600	-2.00111200
H	-5.83503600	-5.12827400	-2.74799000
H	-3.58747400	-4.31577300	-2.84067900
H	-5.19343000	-3.54280800	-0.98555200
H	6.93684300	-4.83983100	2.58621300
H	3.10466100	-4.14849300	1.95044800
H	4.16488700	-4.07846800	-2.15228500
H	1.05816800	-1.63469700	-3.85716100
H	0.98849200	-5.51164500	-2.25763300
H	3.91001400	-3.40962700	-7.46192700
H	6.95396800	-1.43005800	-4.84298200
H	-0.24728900	5.79258100	-1.89356200
H	0.64815200	6.35427500	0.72406600
H	2.96607200	8.84995400	-0.18859500
H	-0.42332000	8.18674400	-2.27955900
H	7.29579900	3.84899200	0.65386300
H	1.43827800	-3.63202200	-6.92338900
H	2.18476200	-2.17366700	-6.28617200
H	0.46365000	-6.15881200	3.46250700
H	-3.35464300	-1.03391700	4.21407000
C	-2.09012000	0.01621300	5.61129200
H	-2.33132000	1.00837800	5.21118800
H	-2.74221200	-0.19182100	6.46616700
H	-1.04732200	0.00779000	5.95756800
H	-2.28640200	-4.79384100	6.77684000
H	-3.45944100	-5.27782000	5.53583800
H	0.32300900	-6.82284700	1.00543300
H	-1.33512000	-6.23712400	0.96659100

H	-2.13122700	4.59437400	-0.64128200
H	-1.51158100	5.23964600	0.85734700
H	6.37055300	-0.65291900	5.09960900
H	3.31226300	-1.07025000	4.92465600
H	4.65772600	-2.55519800	3.38077500
H	4.17994500	0.33234900	4.29348800
H	5.40233200	-1.10051800	2.67078600
H	8.47808700	3.19785500	-2.12927400
H	8.18508500	2.06430700	-0.79738700
H	4.82566400	-6.22513100	2.73977100
H	5.37306900	-7.23048700	1.40947400
H	2.58924400	-3.06046300	-0.25728100
H	3.96956000	5.64769600	8.72165900
H	3.50765600	4.62901400	6.49418600
H	2.59961100	2.18182600	8.12712500
H	2.00956300	1.10308800	6.02912100
H	3.50648200	4.14111600	9.53562000
H	2.06673300	4.55980000	7.49717800
H	4.00849000	2.21214400	7.07039900
H	3.49411100	3.22473600	4.78276100
H	1.10114000	2.62180300	6.18828600
H	4.01254800	2.56499200	2.49813200
H	0.35725200	1.65783800	4.17952600
H	2.63091800	2.18637300	1.54458300
H	0.81895400	1.08543400	2.59100000
H	4.77469000	-2.27056500	-2.84257000
H	5.36262800	0.62164000	-3.68215800
H	6.18953300	-1.31859800	-2.41205400
H	4.15752000	-0.37058900	-4.50965300
H	2.04135600	-5.65673700	-4.60588000
H	1.56850800	2.95435400	-3.94461500
H	4.12500600	3.18747200	-1.31541000
H	5.52725200	2.85588300	0.97478300
H	5.88369500	1.58777200	1.68702300
H	5.10602200	-0.17222700	0.55325200
H	5.83208700	0.95159500	-0.19418600
H	-1.79999500	10.24783000	-1.83389000
H	-3.22580400	9.17221800	-1.85024400
H	-4.88884700	0.12823800	-0.66140900
H	-4.42935800	-0.38496800	-1.96739500
H	-3.78981600	-1.09861400	1.18819900
H	-2.49239200	2.46174600	-1.96037200
H	-1.70207700	4.32964800	-3.21286700
H	-0.59613500	3.22193700	-3.09284400

H	-4.32562800	0.39826600	1.40540400
H	-3.73344200	1.48929400	-1.61007800
H	3.00151200	1.97383400	-4.62594800
H	3.25095100	3.44949900	-4.92224400
H	3.81208600	4.44433700	-3.21127600
H	5.04333200	3.79339000	-3.82677600
H	-3.20765400	-3.41930600	2.11126300
H	-2.40748000	-2.62871900	1.03359700
H	-8.76814900	-3.27579500	-0.43858500
H	5.71278700	-5.70907900	-0.98425300
O	0.75653400	0.72015600	-1.23245600
H	0.47042300	-0.02179800	-1.87298800

$S_3^{TS}Y_Z^{\bullet}$ (W1=OH^{-}, W2=OH^{-}, Ox=OH^{-})

Mn	-0.00637100	-2.58636400	-1.04039100
Mn	0.52818500	-1.89724100	1.54290600
Mn	1.84139500	-0.19044200	-0.29967300
Mn	1.75806800	2.66279700	-1.46905600
Ca	-1.49654700	0.38781500	-0.02886100
O	-0.91584900	-2.05875100	0.49058000
O	0.67844600	-0.14392500	1.11281300
O	1.52996000	-2.23580500	0.01181900
O	2.46631100	1.43821400	-0.15578000
O	0.19601500	-1.00592600	-1.61748500
O	3.27338000	3.60356200	-1.44102000
O	1.01166400	3.45848800	-2.90508900
O	-2.95755100	1.93019800	-1.18413000
O	-3.84216700	-0.34458800	0.68043000
H	6.88532100	4.69549200	0.33007500
C	7.75826500	4.24520200	-0.15946600
H	8.58230900	4.96895000	-0.10865300
C	7.44295500	3.88158100	-1.60770900
C	6.35557600	2.82187000	-1.80662700
O	5.67188000	2.44693300	-0.77859200
O	6.16623600	2.40680500	-2.97408700
C	-7.59791500	7.36534200	1.02557700
C	-6.68860300	6.77019500	-0.07523500
C	-6.45850000	5.30190300	0.13894100
C	-7.57366600	4.41482500	0.13394000
C	-5.16299800	4.78366600	0.39426800
C	-7.41833500	3.08409200	0.41639600
C	-4.98037800	3.45017100	0.69427700
C	-6.11247000	2.54958200	0.73833100
O	-5.97968600	1.31829100	1.04974200
H	2.24907600	7.86316400	1.27059700

C	2.49227200	7.89047800	0.20167600
C	1.30650100	7.53683500	-0.67890700
O	1.22376400	7.93618100	-1.84525200
H	3.30041000	7.17191300	0.00823200
N	0.33626100	6.77456600	-0.09400400
C	-0.74601400	6.12942200	-0.85397400
C	-1.89402400	7.14598100	-1.12085100
O	-3.02316100	7.00668600	-0.62830800
C	-1.29787400	4.93934800	-0.05961800
C	-0.39281800	3.71579200	0.13869900
O	-0.85301400	2.72848700	0.72891000
O	0.81556400	3.84551700	-0.33559000
N	-1.52910500	8.16597600	-1.92688000
C	-2.36442900	9.32608200	-2.18423800
H	-2.47109400	9.49474400	-3.26320500
H	-6.02760200	-3.95538700	-3.94401300
C	-6.10799600	-4.15703100	-2.86548200
C	-7.55833100	-4.14937000	-2.45405800
O	-7.94419700	-4.62115700	-1.37181500
C	-5.17802800	-3.17954500	-2.10587000
C	-3.79997400	-3.20271000	-2.80080300
C	-2.67945900	-2.42733600	-2.10452700
O	-2.81005400	-1.22533200	-1.80893500
O	-1.61778900	-3.13948100	-1.90744400
N	-8.41686100	-3.57820600	-3.34665100
C	-9.82604800	-3.35993800	-3.06134500
H	-10.41515300	-3.49845400	-3.97340400
C	-10.08802600	-1.93643700	-2.48907100
C	-9.13271300	-1.50060300	-1.41061100
C	-8.50451200	-0.29862600	-1.18753300
N	-8.71658800	-2.33314200	-0.37650900
C	-7.85119000	-1.67818000	0.40999600
N	-7.73034600	-0.43393500	-0.05035500
H	3.48383800	-4.82738700	-6.64027600
C	3.63365900	-3.75033800	-6.49780400
C	4.64813300	-3.57283500	-5.37359700
O	4.66034700	-4.33536400	-4.39393700
C	2.29608300	-3.06151200	-6.12250500
C	1.81050300	-3.36942700	-4.74096800
C	1.36685600	-2.54483900	-3.73896600
N	1.71000600	-4.65726400	-4.22189300
C	1.24031800	-4.58264400	-2.95336100
N	1.01528800	-3.31290500	-2.64479100
N	5.46015700	-2.49741100	-5.48716300

C	6.28220100	-2.02115100	-4.37403900
H	6.87513600	-2.86431900	-4.00292300
C	5.40794200	-1.45183300	-3.22515500
C	4.58156100	-0.21619500	-3.60359100
C	3.42205200	0.13766600	-2.67650300
O	3.15394700	-0.64245000	-1.69059800
O	2.76847100	1.18032800	-2.95299600
H	7.60438200	-5.67986800	1.28994400
C	6.94585400	-5.75643900	2.16459600
H	7.40458800	-6.45718400	2.87125600
C	5.53748100	-6.24108100	1.77266600
C	4.82134500	-5.33340900	0.82256900
C	3.78908700	-4.45040500	1.01155200
N	5.13104700	-5.23597000	-0.53342500
C	4.32247400	-4.33887100	-1.13709600
N	3.50806100	-3.85771300	-0.20352800
H	-0.66624500	-7.40685900	3.14928100
C	-0.41963300	-6.35539300	2.93992300
C	-1.52556900	-5.49754200	3.56834400
O	-2.50267500	-5.10591200	2.92819300
C	-0.31458000	-6.14877800	1.43576100
C	0.09978400	-4.74707600	1.00451000
O	0.42324400	-3.91253500	1.91537200
O	0.07924700	-4.53100900	-0.24097400
N	-1.39451200	-5.24331800	4.90616400
C	-2.51820000	-4.76428500	5.71156500
C	-2.95854400	-3.30959800	5.45599500
O	-4.11040700	-2.96623500	5.70538700
N	-1.99071500	-2.46506700	5.01581100
C	-2.31333300	-1.11959500	4.55021600
C	-1.49208800	-0.81206200	3.28748200
O	-1.79271400	0.14416800	2.56350500
O	-0.48808900	-1.62368700	3.11893800
O	-1.62520900	3.41271200	-2.97753600
O	4.52243800	4.53963100	-3.75366500
O	2.61947700	3.09473700	-4.96002700
O	6.03755800	2.01411300	1.89968700
O	-5.11277300	0.23573200	-1.70451700
O	-3.34622900	-2.90635100	1.28108200
H	5.94686300	-1.89179800	5.49502800
C	5.39090300	-0.94664600	5.55973000
H	5.10328100	-0.79442300	6.60735200
C	4.13833200	-0.96855000	4.65324400
C	4.45962300	-1.15821600	3.15630500

C	3.23733700	-1.20170300	2.23477400
O	2.20791500	-1.83140600	2.64264700
O	3.34479600	-0.63394600	1.10091500
H	4.72477700	3.95884900	8.89322900
C	3.81894800	4.54837000	8.69853700
C	3.26752900	4.27238500	7.28780100
C	2.89872300	2.79508600	7.06566000
C	2.09482000	2.50238700	5.79153400
N	2.89006300	2.63439800	4.57164600
C	2.50081400	2.16874800	3.35537400
N	1.26056200	1.66526300	3.21352100
N	3.33966500	2.17337900	2.33231700
O	5.91864400	-0.12034900	0.07917300
H	-8.58206400	6.88178100	1.04326300
H	-7.14176000	7.24265400	2.01523600
H	-7.75078700	8.43654700	0.84914100
H	-5.72567800	7.29279700	-0.11406500
H	-7.17634000	6.91888100	-1.05202600
H	-8.56260300	4.81410100	-0.08402600
H	-8.27171300	2.40941100	0.44840000
H	-3.98884400	3.03847600	0.86023400
H	-4.31088600	5.45997300	0.32421100
H	-7.09153200	0.29209400	0.37636700
H	-8.52666500	0.62531600	-1.74612400
H	-7.31688200	-2.09858600	1.25026800
H	-1.12929200	-2.85591700	4.64521800
H	-0.60099300	-5.64712800	5.38838500
H	5.34959600	-1.89466000	-6.29283300
H	-8.02551300	-3.16368900	-4.18373800
H	-10.12674900	-4.12947700	-2.34352600
H	-11.12325700	-1.89748900	-2.11847400
H	-10.02177200	-1.19840500	-3.29835500
H	-3.90202100	-2.77551200	-3.81084200
H	-5.58608600	-2.16065000	-2.10951800
H	-5.74012200	-5.17968200	-2.70885500
H	-3.46358100	-4.23868200	-2.91974800
H	-5.07586000	-3.48947000	-1.05796100
H	6.90009400	-4.76734100	2.63543900
H	3.21939700	-4.18947700	1.89333000
H	4.34497300	-4.09906600	-2.19612100
H	1.24766600	-1.47211300	-3.70774300
H	1.04238400	-5.42159500	-2.30189600
H	3.99301600	-3.34250100	-7.45066200
H	6.96908300	-1.26404600	-4.76612600

H	-0.32727200	5.77743500	-1.80662800
H	0.57567900	6.33556000	0.78674700
H	2.84890900	8.88729300	-0.07115900
H	-0.53799100	8.19256500	-2.19500000
H	8.04193300	3.36243600	0.42609300
H	1.53920700	-3.33989200	-6.87026700
H	2.41132700	-1.97276800	-6.18983600
H	0.53837200	-6.14264500	3.43196000
H	-3.37335900	-1.10726800	4.26968600
C	-2.06955100	-0.04917800	5.62291900
H	-2.32361400	0.94220200	5.22915700
H	-2.69629800	-0.25798300	6.49647500
H	-1.01697500	-0.05318300	5.93652500
H	-2.23885000	-4.85844300	6.76666900
H	-3.40755500	-5.37957200	5.53517500
H	0.39679600	-6.86231600	0.99803900
H	-1.28444800	-6.33973900	0.96165700
H	-2.17766700	4.54952500	-0.58161400
H	-1.66196300	5.27288000	0.92149200
H	6.07291800	-0.13671400	5.26978100
H	3.45335100	-1.76437900	4.97377300
H	4.99428900	-2.11126200	3.00837000
H	3.59188900	-0.02308300	4.77463100
H	5.12271600	-0.36857800	2.79299400
H	7.10746000	4.76765400	-2.16771900
H	8.33899100	3.52477000	-2.13372800
H	4.91557400	-6.33116900	2.67197400
H	5.59708400	-7.25023400	1.33888900
H	2.72109800	-3.13202600	-0.28168100
H	4.07423500	5.60758000	8.82019900
H	4.00580400	4.59835300	6.53844500
H	2.27877800	2.45934400	7.90946900
H	1.71214100	1.47297900	5.82733700
H	3.07747300	4.28736600	9.46526800
H	2.37132600	4.88961800	7.12149800
H	3.80179500	2.16622800	7.07754700
H	3.74219200	3.18026600	4.59803000
H	1.22223700	3.17743600	5.75814200
H	4.38408100	2.18715500	2.39569100
H	0.50461100	1.95805800	3.81614600
H	2.97566100	1.91298200	1.39679100
H	0.99886300	1.16381600	2.35236800
H	4.72720900	-2.24359300	-2.90187700
H	5.20365200	0.69381700	-3.61060800

H	6.04241600	-1.21098100	-2.36224600
H	4.14199900	-0.32173400	-4.60802500
H	2.11627100	-5.48278500	-4.64324200
H	1.58674800	3.40736600	-3.74473000
H	4.03002300	3.06033500	-1.07889200
H	5.98914300	2.51850500	1.04795200
H	6.13804400	1.10714600	1.51740900
H	4.98799500	-0.36003500	0.25852600
H	5.82222800	0.72053300	-0.44094300
H	-1.93200700	10.22786200	-1.72975000
H	-3.35000700	9.14157100	-1.74851700
H	-4.87987800	0.02218600	-0.76781200
H	-4.40172300	-0.29572200	-2.13150000
H	-3.79814400	-1.26021800	1.07361400
H	-2.52353700	2.50547300	-1.87404000
H	-1.85656800	4.31456000	-3.24867700
H	-0.61734100	3.38255600	-2.98053300
H	-4.32549000	0.24019100	1.29538400
H	-3.78779800	1.55585100	-1.55870600
H	2.88696100	2.27281000	-4.49872500
H	3.34449600	3.73330500	-4.68688300
H	4.02325200	4.41515500	-2.91088100
H	5.20765500	3.83514100	-3.68798700
H	-3.25879300	-3.56598000	1.99893300
H	-2.41869400	-2.77856600	0.95755300
H	-8.74902500	-3.36667600	-0.44564700
H	5.86381400	-5.74806400	-1.01087100
O	0.33627700	1.34496600	-1.39422100
H	0.27440600	0.95003900	-2.28375100

$S_3^{B,W}Y_z^{\bullet}$ (W1=OH⁻, W2=OH⁻, Ox=OH⁻)

Mn	0.18482600	-2.52304700	-0.97900900
Mn	0.64919700	-1.90539800	1.62262400
Mn	1.87336000	-0.28568800	-0.27419600
Mn	1.36067800	2.54524500	-1.77556800
Ca	-1.46688300	0.39558000	-0.04434900
O	-0.77901400	-2.09455700	0.49598400
O	0.72077700	-0.19184900	1.19808500
O	1.69833000	-2.26175600	0.08754600
O	1.99332500	1.42660100	-0.31014900
O	0.46799600	-0.81161300	-1.39440100
O	2.93740600	3.42310700	-1.76371800
O	0.74808500	3.34128100	-3.27293900
O	-3.11540800	1.89959100	-1.08982000
O	-3.77201900	-0.47242300	0.69424300

H	6.69311800	4.98630300	0.50351000
C	7.56166900	4.65661100	-0.07978700
H	8.30165400	5.46728000	-0.06951600
C	7.16436200	4.30545800	-1.51475900
C	6.20501200	3.12133900	-1.69159100
O	5.48701400	2.75080400	-0.68996200
O	6.14519600	2.60689900	-2.83600600
C	-7.93323900	7.00674900	1.07638400
C	-6.98180100	6.46137100	-0.01478000
C	-6.68197200	5.00630700	0.20186200
C	-7.74892100	4.06223900	0.15896300
C	-5.37120800	4.55567400	0.49959500
C	-7.53376200	2.73943600	0.43939800
C	-5.12992600	3.23158600	0.80039700
C	-6.21225800	2.27068900	0.79715700
O	-6.02157200	1.04338500	1.09628000
H	1.90889900	7.90177000	1.35599200
C	2.12102200	8.03493400	0.28850700
C	0.96742900	7.61428600	-0.60443900
O	0.87633200	8.00387000	-1.77374900
H	3.00327700	7.43509200	0.02910200
N	0.03631400	6.80111800	-0.03144700
C	-1.02320600	6.12331800	-0.78919900
C	-2.21742300	7.08916500	-1.03653500
O	-3.33654300	6.89761500	-0.53917900
C	-1.50011800	4.89287200	-0.01427300
C	-0.54967100	3.69323700	0.03203100
O	-0.86577900	2.70017100	0.70944500
O	0.51592000	3.84438300	-0.69083300
N	-1.90201100	8.12858900	-1.84118400
C	-2.79372500	9.24517800	-2.10349800
H	-2.90203800	9.40845400	-3.18314100
H	-5.78904900	-4.19852800	-3.96457700
C	-5.86481900	-4.40140600	-2.88604800
C	-7.31455100	-4.46437300	-2.47754700
O	-7.67817800	-4.95762300	-1.39751900
C	-4.98496400	-3.37116200	-2.12104300
C	-3.58306800	-3.33778100	-2.77278900
C	-2.51394700	-2.45406400	-2.10992300
O	-2.72997300	-1.26580700	-1.81137700
O	-1.37739100	-3.05618100	-1.96377800
N	-8.19828100	-3.93613300	-3.37187500
C	-9.61712300	-3.78753100	-3.08861600
H	-10.19671400	-3.94794000	-4.00311500

C	-9.94675500	-2.38099400	-2.50855800
C	-9.01882400	-1.90684500	-1.42187800
C	-8.44653000	-0.67881800	-1.18981300
N	-8.57636000	-2.72283300	-0.38530400
C	-7.74909700	-2.03192800	0.41163100
N	-7.67879300	-0.78248600	-0.04488500
H	3.73185000	-4.57011300	-6.62421200
C	3.85532900	-3.48970200	-6.48416500
C	4.86592000	-3.27955400	-5.36241900
O	4.92842000	-4.06314900	-4.40008900
C	2.50279000	-2.82797000	-6.10198600
C	2.02859400	-3.15184300	-4.71840700
C	1.53739900	-2.35349200	-3.71458400
N	2.00342900	-4.44373100	-4.20076100
C	1.53384500	-4.39914500	-2.93186100
N	1.23875200	-3.14464200	-2.61896600
N	5.60698200	-2.15389000	-5.45464100
C	6.40907500	-1.64630200	-4.34048400
H	7.06734700	-2.45331300	-3.99921300
C	5.52271900	-1.16082500	-3.17027400
C	4.59173800	0.00400900	-3.52753400
C	3.34758300	0.15164900	-2.67050800
O	3.26937800	-0.55738700	-1.59576000
O	2.46423100	0.96097400	-3.06118600
H	7.91556100	-5.22551700	1.32807100
C	7.23747900	-5.38845500	2.17539000
H	7.74203000	-6.04940100	2.88920500
C	5.90489700	-6.01189200	1.72110500
C	5.14290200	-5.15817300	0.75813300
C	4.05995900	-4.33555600	0.93140700
N	5.47265900	-5.03723500	-0.59130900
C	4.62912900	-4.18458000	-1.20967100
N	3.77213200	-3.75552200	-0.28774400
H	-0.26886900	-7.41569600	3.15847800
C	-0.09166100	-6.35554800	2.92298000
C	-1.23794200	-5.55312500	3.55384200
O	-2.23055100	-5.20220400	2.91399100
C	-0.02790300	-6.17359500	1.41096000
C	0.30300000	-4.74946000	0.98530300
O	0.59166900	-3.92953800	1.91571600
O	0.25447000	-4.50401600	-0.25901900
N	-1.12284100	-5.30175200	4.89422000
C	-2.27422900	-4.88873200	5.69831600
C	-2.79971600	-3.46389500	5.42997600

O	-3.98217300	-3.19775800	5.62109600
N	-1.86474700	-2.55341700	5.04960200
C	-2.24630900	-1.23066700	4.56179900
C	-1.43098000	-0.89368700	3.30815400
O	-1.74522200	0.05896100	2.58892400
O	-0.40668600	-1.68688000	3.14949600
O	-1.93466800	3.56274100	-2.96106300
O	4.32730700	4.44304500	-3.90384700
O	2.56482700	2.85604700	-5.14447100
O	5.60008400	2.56045800	2.00089400
O	-5.12968700	0.04411100	-1.65818700
O	-3.18226100	-3.02693600	1.28218400
H	5.60802300	-1.74814600	5.81181800
C	5.43706600	-0.68439400	5.59726000
H	5.21020600	-0.18086500	6.54548700
C	4.28141200	-0.49858400	4.59398400
C	4.54734900	-1.16139700	3.22415500
C	3.33611100	-1.13960600	2.29479200
O	2.31836100	-1.81989300	2.68360000
O	3.40932000	-0.49238700	1.21123100
H	4.65132800	4.43731800	8.79116900
C	3.58656900	4.70493600	8.76730300
C	2.96418500	4.40648000	7.38444100
C	3.05017100	2.92113100	6.97892900
C	2.15357100	2.47933800	5.80767700
N	2.65551100	2.86433400	4.48219900
C	2.19198600	2.31410400	3.32526100
N	1.01849300	1.65093400	3.34365000
N	2.89275700	2.40123900	2.20670500
O	5.95527400	0.28478900	0.37617800
H	-8.89049000	6.47150800	1.08246200
H	-7.48227800	6.90664500	2.07095600
H	-8.14031100	8.06870100	0.89920900
H	-6.04584700	7.03160400	-0.04085100
H	-7.46495500	6.58708400	-0.99688400
H	-8.74984400	4.41143300	-0.08860900
H	-8.35162500	2.02145900	0.43913100
H	-4.12523500	2.87166100	0.99967300
H	-4.55315600	5.27488300	0.45891700
H	-7.07724200	-0.03028900	0.39024900
H	-8.50290000	0.24459600	-1.74679200
H	-7.20575900	-2.43018000	1.25690900
H	-0.97039400	-2.89550300	4.71060800
H	-0.31726700	-5.68008800	5.37741800

H	5.45517700	-1.54471200	-6.24882700
H	-7.82629000	-3.50051800	-4.20707200
H	-9.88265000	-4.57486000	-2.37626600
H	-10.98456400	-2.39182600	-2.14330100
H	-9.91052100	-1.63591700	-3.31327500
H	-3.68054000	-2.97595900	-3.80904400
H	-5.43889000	-2.37213000	-2.14788700
H	-5.44630400	-5.40336200	-2.72333600
H	-3.17733400	-4.35414100	-2.82763600
H	-4.89761200	-3.66199100	-1.06629900
H	7.06788300	-4.41889000	2.65829800
H	3.46172300	-4.10440400	1.80264800
H	4.65763900	-3.93908500	-2.26866300
H	1.36808600	-1.28571500	-3.68478300
H	1.38357900	-5.24996600	-2.28287200
H	4.20026400	-3.07315800	-7.43840200
H	7.03536700	-0.83536400	-4.72637300
H	-0.60055300	5.80775500	-1.75086600
H	0.23560400	6.43529200	0.89117000
H	2.35693300	9.08435800	0.08920900
H	-0.91522700	8.19929400	-2.11593500
H	7.99416000	3.79223900	0.43992300
H	1.74889900	-3.12172200	-6.84679500
H	2.59420800	-1.73705300	-6.16803200
H	0.85930900	-6.07203500	3.39091300
H	-3.30294700	-1.27661300	4.27148200
C	-2.06822800	-0.13607900	5.62356200
H	-2.37070000	0.83580400	5.21565300
H	-2.69024800	-0.36624800	6.49503800
H	-1.01976300	-0.08094800	5.94598100
H	-1.98747800	-4.95847100	6.75338700
H	-3.12550400	-5.55678200	5.52621900
H	0.71695800	-6.85025000	0.97027100
H	-0.99342200	-6.42417000	0.95647400
H	-2.40202200	4.50551000	-0.49999100
H	-1.80074200	5.16249600	1.00696300
H	6.37218300	-0.26341100	5.20578400
H	3.35363500	-0.91429200	5.01048600
H	4.80608400	-2.21922000	3.38553400
H	4.10783600	0.57393400	4.43283700
H	5.38759800	-0.68064300	2.71225100
H	6.67022900	5.16315300	-1.99795200
H	8.04879200	4.09193300	-2.12839500
H	5.26119000	-6.18338900	2.59273500

H	6.08522900	-6.99991300	1.27289900
H	2.96286300	-3.07762100	-0.36684400
H	3.50122100	5.77055700	9.00993900
H	3.44473800	5.03536900	6.62019100
H	2.73930700	2.31176600	7.84019500
H	2.04716600	1.38508200	5.82400100
H	3.07816000	4.13375300	9.55540600
H	1.90417200	4.70312600	7.39977400
H	4.09299000	2.63483300	6.77089900
H	3.53740100	3.35993500	4.41742400
H	1.14871500	2.90558500	5.95858100
H	3.93886500	2.52529500	2.19777500
H	0.29187000	1.90049300	4.00004100
H	2.47111400	2.07801400	1.31975400
H	0.73479200	1.13708400	2.49965700
H	4.91924800	-2.00725500	-2.83310500
H	5.11868400	0.97065500	-3.41006800
H	6.16021800	-0.87317700	-2.32413500
H	4.24587100	-0.05175500	-4.56959300
H	2.46406100	-5.24075400	-4.62133100
H	1.41067700	3.30828500	-4.03905000
H	3.65987000	2.97483000	-1.25698000
H	5.63244500	2.98212200	1.10143200
H	5.83122100	1.63757500	1.73002800
H	5.03955100	-0.04408200	0.47791300
H	5.83340000	1.07691800	-0.21039900
H	-2.41219700	10.16830800	-1.64614200
H	-3.77108600	9.00953700	-1.67380800
H	-4.85033500	-0.17972800	-0.73613200
H	-4.39394800	-0.41373800	-2.12333500
H	-3.69403200	-1.38587100	1.08441000
H	-2.71671100	2.49024100	-1.78067300
H	-2.20462900	4.42529600	-3.31261600
H	-0.94892600	3.50009700	-3.11659500
H	-4.30070100	0.08358200	1.29972400
H	-3.92203800	1.48216700	-1.46819700
H	2.72788200	2.07002500	-4.58312200
H	3.27726400	3.49776100	-4.84586700
H	3.77411200	4.28668500	-3.09816600
H	5.09132700	3.84443200	-3.74073300
H	-3.06698600	-3.68838000	1.99453100
H	-2.26237900	-2.86429900	0.95499100
H	-8.55999700	-3.75576500	-0.45908400
H	6.24165900	-5.50719100	-1.05514300

O	-0.24701500	1.52778500	-1.79596000
H	-0.42833900	1.36103700	-2.73545600

$S_3^{A,W}Y_z^{\bullet}$ (W1=H_2O, W2=OH^-, Ox=OH^-)*

Mn	0.04586000	-2.55704100	-1.04746300
Mn	0.69192400	-1.73919000	1.52876200
Mn	2.09919800	0.13377500	-0.06964100
Mn	1.71582300	2.41788600	-1.63551700
Ca	-1.50788900	0.36602100	0.00593600
O	-0.77105000	-1.93843100	0.48868000
O	0.83727700	0.03525600	1.27870800
O	1.59798600	-1.88488300	-0.14323700
O	2.66427200	1.79538100	-0.01722900
O	-0.30416000	-1.15911500	-2.05757700
O	2.86666100	3.85284100	-1.95407500
O	0.63524800	2.71075400	-3.03361200
O	-2.94108300	1.76836600	-1.28186100
O	-3.83841700	-0.23228800	0.80888100
H	7.62915100	5.31884500	-0.52971600
C	7.79097200	4.31589800	-0.11802700
H	8.82965300	4.25334000	0.22779100
C	7.52338600	3.23188500	-1.17062500
C	6.15508500	3.29411900	-1.81596300
O	5.23015700	3.84576700	-1.02496400
O	5.91938900	2.86024200	-2.93926400
C	-7.59645100	7.28973100	1.03668100
C	-7.09204200	6.60440500	-0.25421400
C	-6.81338700	5.15155800	-0.00925600
C	-7.89377300	4.28995000	0.33775300
C	-5.49611800	4.62710200	-0.04635100
C	-7.67217500	2.98424300	0.68708400
C	-5.23956600	3.32357700	0.31927600
C	-6.32525000	2.45731900	0.72709200
O	-6.11525000	1.25594900	1.10338200
H	2.03139500	8.29078700	1.16845900
C	2.49113700	7.91118900	0.24871400
C	1.58016400	7.01220700	-0.56573900
O	1.96320300	6.46493300	-1.59941200
H	3.40204200	7.35626100	0.49993300
N	0.28699600	6.89662600	-0.11766000
C	-0.72760400	6.12720600	-0.82819400
C	-1.84309200	6.99871100	-1.42297000
O	-2.86085800	6.46681400	-1.88792500
C	-1.27530500	4.95041800	0.00324300
C	-0.45037300	3.64873300	-0.00359600

O	-0.90020200	2.66061800	0.59779200
O	0.66040400	3.70389800	-0.66044800
N	-1.57367600	8.32108100	-1.45034800
C	-2.37080400	9.31778700	-2.14403900
H	-1.81712500	9.73476800	-2.99432800
H	-5.88385400	-3.85036800	-3.95976000
C	-5.99003000	-4.19420700	-2.92058100
C	-7.44445100	-4.20513000	-2.52339000
O	-7.82874600	-4.71551400	-1.45726400
C	-5.08494500	-3.36061100	-2.01733000
C	-3.67701500	-3.43747700	-2.61225700
C	-2.64302400	-2.66501500	-1.82908900
O	-2.87131800	-1.51730500	-1.42733800
O	-1.53952000	-3.34017400	-1.66085000
N	-8.29603000	-3.59584200	-3.39128700
C	-9.71422500	-3.42961200	-3.12350200
H	-10.28235000	-3.56048600	-4.05002800
C	-10.04244900	-2.03838600	-2.51665300
C	-9.14827300	-1.62242300	-1.38194900
C	-8.60030600	-0.40448300	-1.06030900
N	-8.70974200	-2.49942800	-0.39607600
C	-7.90793800	-1.85399900	0.46023000
N	-7.84972700	-0.57460200	0.08818900
H	3.70086500	-4.75861200	-6.69052700
C	3.75914300	-3.67708700	-6.51876600
C	4.72385600	-3.45275200	-5.36332900
O	4.71303900	-4.18370500	-4.35965900
C	2.35566100	-3.11308500	-6.15799800
C	1.89022500	-3.40685500	-4.76411700
C	1.41897200	-2.56392300	-3.79017100
N	1.83126600	-4.67604500	-4.19816500
C	1.36320100	-4.57348700	-2.93198600
N	1.09497300	-3.30091800	-2.66317400
N	5.52521200	-2.36976100	-5.46955000
C	6.38511200	-1.93732500	-4.37586500
H	6.94358300	-2.80456400	-4.00728500
C	5.58005600	-1.31793600	-3.19188100
C	4.70014100	-0.10973800	-3.55333200
C	3.66308000	0.32120500	-2.50866300
O	3.46655300	-0.38241400	-1.46801600
O	3.02023600	1.38530800	-2.78108900
H	7.68480200	-5.76009100	1.23972800
C	7.06179300	-5.70693800	2.14173100
H	7.46399000	-6.42044200	2.86879200

C	5.58662100	-6.04607800	1.83797100
C	4.89792800	-5.13076500	0.86925800
C	3.87257900	-4.23474700	1.04110100
N	5.19564600	-5.07314200	-0.49217500
C	4.39147400	-4.18798700	-1.11607300
N	3.58687600	-3.66940800	-0.18945800
H	-0.60547300	-7.42871000	2.98337600
C	-0.30037100	-6.37718700	2.88953700
C	-1.39849600	-5.51790800	3.52614700
O	-2.34808500	-5.08392900	2.87072300
C	-0.10958700	-6.04973700	1.40521400
C	0.32502200	-4.63065800	1.03748500
O	0.59414200	-3.78369100	1.94104500
O	0.38099300	-4.39551400	-0.21270700
N	-1.28852800	-5.29906700	4.87004300
C	-2.42207100	-4.82215700	5.66400400
C	-2.86917500	-3.37382400	5.39295200
O	-4.02971000	-3.03964500	5.60539300
N	-1.89990100	-2.51057300	4.98301300
C	-2.24653200	-1.16881200	4.52611500
C	-1.39111000	-0.81598400	3.29520000
O	-1.69585700	0.15079500	2.58585600
O	-0.34207100	-1.57574300	3.13021800
O	-1.94891900	3.62982100	-2.86762100
O	3.84881900	4.10824400	-4.51369200
O	2.06196200	2.26393700	-5.24081000
O	4.95034500	2.38766400	1.32147500
O	-5.10099000	0.08779600	-1.62573000
O	-3.18250300	-2.73031000	1.54728200
H	5.58554600	-2.01080600	5.72282900
C	5.45252000	-0.93265500	5.56154200
H	5.23829900	-0.47317700	6.53485400
C	4.32282200	-0.66167300	4.56338900
C	4.61684000	-1.27524400	3.18035300
C	3.42758000	-1.15438500	2.25425200
O	2.38048000	-1.82212400	2.56360400
O	3.53591400	-0.40985400	1.23829100
H	4.86720400	4.22858800	8.58360100
C	3.82101500	4.52850600	8.72909000
C	2.96110200	4.14786600	7.51720500
C	2.96073500	2.63621900	7.23990100
C	2.01596700	2.20044800	6.11579100
N	2.45056400	2.73846400	4.82635700
C	1.91340400	2.41184600	3.63353900

N	0.82533600	1.64386800	3.56461000
N	2.47150500	2.89687700	2.51406800
O	6.16101200	0.11153500	0.05040700
H	-8.52462500	6.83160300	1.39773600
H	-6.84891900	7.21536100	1.83554900
H	-7.79477000	8.35109600	0.84750200
H	-6.18830300	7.10337100	-0.62408600
H	-7.86201400	6.70552800	-1.03421400
H	-8.90532700	4.69178200	0.33996700
H	-8.48769300	2.32951000	0.98802400
H	-4.24128100	2.89584200	0.24653000
H	-4.68472200	5.27038900	-0.38674600
H	-7.25277300	0.15156200	0.55415700
H	-8.66892700	0.55365500	-1.55383100
H	-7.37710700	-2.30046300	1.28907800
H	-1.02310700	-2.88401300	4.63163800
H	-0.52561200	-5.74991700	5.36063900
H	5.49049800	-1.81434600	-6.31492000
H	-7.90846300	-3.17074000	-4.22469600
H	-10.00496000	-4.23160200	-2.43758200
H	-11.09286900	-2.04534200	-2.18984600
H	-9.96555200	-1.26837700	-3.29443900
H	-3.68080500	-3.01653300	-3.63049300
H	-5.42989800	-2.32102600	-1.96843200
H	-5.65106400	-5.23940400	-2.90055900
H	-3.35267500	-4.48044600	-2.69871200
H	-5.09020900	-3.75869800	-0.99471500
H	7.15468900	-4.69637000	2.55643600
H	3.30956100	-3.95242100	1.91969000
H	4.40624100	-3.98185100	-2.18313500
H	1.24093000	-1.50009300	-3.82883400
H	1.19881000	-5.39683500	-2.25242800
H	4.10286800	-3.21548800	-7.45172000
H	7.10201800	-1.21530600	-4.78036900
H	-0.19456000	5.70637200	-1.69122800
H	0.05515600	7.27175400	0.79527500
H	2.79206200	8.75972700	-0.37720900
H	-0.66622600	8.59521200	-1.09352200
H	7.14257100	4.20139800	0.75759300
H	1.63023100	-3.48974000	-6.89282600
H	2.36254900	-2.02157000	-6.26639400
H	0.64024400	-6.26047500	3.44402300
H	-3.29314400	-1.18149800	4.20182600
C	-2.06946600	-0.10628400	5.61968300

H	-2.33009500	0.88535100	5.22990800
H	-2.72610200	-0.33873000	6.46450300
H	-1.03208400	-0.09778000	5.98410700
H	-2.14826200	-4.90484600	6.72136800
H	-3.30688700	-5.44438600	5.48891200
H	0.61511500	-6.74164500	0.95499000
H	-1.05570900	-6.20600300	0.87281200
H	-2.25268800	4.66509900	-0.39872600
H	-1.43904200	5.24311600	1.04982900
H	6.40655000	-0.52664400	5.20106200
H	3.37813300	-1.07888600	4.93920100
H	4.83806300	-2.34535900	3.30443100
H	4.17352400	0.42194800	4.44650400
H	5.47686200	-0.79648600	2.70037700
H	8.25926800	3.26930500	-1.98193800
H	7.60129300	2.22650600	-0.72783300
H	5.00669300	-6.02064600	2.76896800
H	5.51664000	-7.07856000	1.46442400
H	2.83210600	-2.93358400	-0.31571600
H	3.80247400	5.61088500	8.90089700
H	3.32383600	4.70061000	6.63518300
H	2.64991500	2.10510800	8.14991500
H	1.99697700	1.10418900	6.04047600
H	3.45522100	4.03644700	9.63967400
H	1.92448100	4.48065800	7.67833100
H	3.97946000	2.28854500	7.01031800
H	3.11818000	3.49982100	4.82978900
H	0.99366300	2.54373200	6.34514200
H	3.49679700	2.97255100	2.45477100
H	0.27938300	1.42604000	4.38603000
H	2.10169900	2.57617300	1.60955800
H	0.60044600	1.15064900	2.68788400
H	4.93821200	-2.09976600	-2.77865600
H	5.30297900	0.78594400	-3.75150400
H	6.28290300	-1.02800700	-2.39988800
H	4.12616700	-0.31463300	-4.47116000
H	2.23616200	-5.51109600	-4.60261600
H	1.10678500	2.61303600	-3.92765200
H	4.28258600	3.82344100	-1.44721500
H	5.21997200	3.10164700	0.70158600
H	4.19211300	1.99248300	0.81680000
H	5.27514400	-0.25022200	-0.12508500
H	5.95145300	0.88107600	0.62469300
H	-2.65122200	10.13119700	-1.46418000

H	-3.27480200	8.82701800	-2.51425200
H	-4.89335000	-0.02756500	-0.66663900
H	-4.41582000	-0.52219200	-1.97700300
H	-3.78231700	-1.12739000	1.23796600
H	-2.63581200	2.48440700	-1.91233700
H	-2.12351700	4.58127600	-2.74064200
H	-0.97751300	3.46817500	-2.98899000
H	-4.40120600	0.34894100	1.35665600
H	-3.77835600	1.37121300	-1.62194800
H	2.51837700	1.60302600	-4.68877300
H	2.71749600	3.02401200	-5.17934300
H	3.37485400	4.18602900	-3.65245900
H	4.65472500	3.63070800	-4.22548400
H	-3.09647600	-3.47032000	2.18255200
H	-2.29158800	-2.64742300	1.13598100
H	-8.68994500	-3.52988400	-0.52867100
H	5.91734300	-5.60946200	-0.96106500
O	0.85595900	0.79263400	-1.19220700
H	0.33564000	-0.37868100	-1.92163200
H	2.40917800	4.70501200	-1.76072800

$S_3^{TS}Y_z^{\bullet}$ (W1=H_2O, W2=OH^-, Ox=OH^-)*

Mn	0.21141500	-2.56646300	-1.05575300
Mn	0.65902600	-1.87799000	1.54699400
Mn	1.89206300	-0.04856800	-0.20535800
Mn	1.57364600	2.60007700	-1.64923300
Ca	-1.51921900	0.31079000	0.01839700
O	-0.74738000	-2.09412600	0.47568400
O	0.69537800	-0.08366000	1.14987200
O	1.71676700	-2.09598200	0.04373000
O	2.37496400	1.66249900	-0.16138100
O	0.35658700	-0.98992400	-1.64020100
O	2.88964000	3.91305100	-1.82114800
O	0.65830100	3.11930000	-3.11254300
O	-2.96327900	1.82805600	-1.14324100
O	-3.84875900	-0.42995300	0.71071800
H	7.56063200	5.45815800	-0.46712000
C	7.71533800	4.48520100	0.01347600
H	8.73379900	4.46447200	0.41945000
C	7.52240600	3.33710600	-0.98607300
C	6.17401400	3.34416100	-1.67177000
O	5.21998900	3.88834200	-0.91067800
O	5.97446300	2.88851700	-2.79308500
C	-7.73640900	7.14021400	1.08345200
C	-7.19084300	6.45898200	-0.19253400

C	-6.87748100	5.01543300	0.06562100
C	-7.94137600	4.11632800	0.36555900
C	-5.54383700	4.53492100	0.08168100
C	-7.69171600	2.81172100	0.70133900
C	-5.25984200	3.23527300	0.44028500
C	-6.33159200	2.32251000	0.77470000
O	-6.09752400	1.11282200	1.10966200
H	1.86207300	8.33165600	1.29173800
C	2.34205600	7.97036500	0.37494800
C	1.46602900	7.05015500	-0.45478700
O	1.88016500	6.50603900	-1.47793300
H	3.26639600	7.44157000	0.63312900
N	0.16761700	6.91825300	-0.02851000
C	-0.83254000	6.13269300	-0.74195500
C	-1.96120100	6.98941300	-1.33236400
O	-2.97508500	6.44610900	-1.79374200
C	-1.36119100	4.95350200	0.09417400
C	-0.46795600	3.70158600	0.12035300
O	-0.82283800	2.70949000	0.76725100
O	0.62338900	3.83063100	-0.58072800
N	-1.71147000	8.31572100	-1.35732600
C	-2.52994900	9.30215400	-2.04002900
H	-1.99197100	9.72876500	-2.89564200
H	-5.78320000	-4.01903400	-4.03553600
C	-5.87215700	-4.27142200	-2.96872300
C	-7.32385400	-4.29939400	-2.56648000
O	-7.70174100	-4.81051300	-1.49896300
C	-4.96772900	-3.31658800	-2.15185000
C	-3.56294900	-3.31848800	-2.79083600
C	-2.48103600	-2.51288600	-2.06973600
O	-2.66996700	-1.33272000	-1.71953100
O	-1.37552000	-3.17240100	-1.91693400
N	-8.18473600	-3.71671700	-3.44517600
C	-9.60939700	-3.57947500	-3.19167200
H	-10.16469700	-3.72024400	-4.12435300
C	-9.96798500	-2.19413200	-2.58652700
C	-9.08856900	-1.76471000	-1.44498300
C	-8.54384000	-0.54412100	-1.12712900
N	-8.66126500	-2.63387000	-0.44715500
C	-7.86746400	-1.98236600	0.41164700
N	-7.80427700	-0.70588400	0.03045500
H	3.81381700	-4.60740100	-6.65506900
C	3.89001600	-3.52578800	-6.49066600
C	4.85060500	-3.30827300	-5.32920200

O	4.82648500	-4.03874700	-4.32636000
C	2.49456500	-2.93460800	-6.15037100
C	2.00250400	-3.27026300	-4.77778500
C	1.60324200	-2.46196500	-3.74425900
N	1.87840600	-4.56697900	-4.28895500
C	1.43818200	-4.51661800	-3.01049500
N	1.25749100	-3.24865000	-2.66016000
N	5.66131200	-2.23101700	-5.43286600
C	6.46533300	-1.75432800	-4.31254300
H	7.03891000	-2.59465500	-3.90745200
C	5.58009100	-1.15361600	-3.17085500
C	4.62967200	-0.00721600	-3.57641500
C	3.50770100	0.34002700	-2.58141100
O	3.31248400	-0.42224900	-1.57755900
O	2.79856100	1.36202000	-2.85062400
H	7.83893200	-5.47151500	1.30946400
C	7.17100400	-5.57110600	2.17442700
H	7.63407300	-6.27137600	2.87833900
C	5.77825800	-6.08106000	1.75864900
C	5.05814400	-5.17652600	0.80844800
C	4.04240100	-4.27585800	1.00290700
N	5.34940100	-5.09807600	-0.55284100
C	4.54573000	-4.19932400	-1.15856300
N	3.75301600	-3.69448800	-0.21613300
H	-0.43866600	-7.45306200	3.01810700
C	-0.18144200	-6.39527100	2.86221400
C	-1.30136900	-5.55859500	3.49372400
O	-2.25884300	-5.14217400	2.83960200
C	-0.02909700	-6.13151400	1.37163400
C	0.38111500	-4.71854700	0.98007000
O	0.66211500	-3.87877000	1.90390200
O	0.39962400	-4.48713500	-0.26232800
N	-1.20219300	-5.34502800	4.84156300
C	-2.35346000	-4.90915100	5.63548900
C	-2.84647600	-3.47645700	5.35480500
O	-4.02797400	-3.18735300	5.50541200
N	-1.88415300	-2.57322500	5.01292100
C	-2.24316800	-1.24249700	4.53297700
C	-1.43192500	-0.91693000	3.27699800
O	-1.75466700	0.03431200	2.55272300
O	-0.38666900	-1.67554600	3.11957000
O	-2.00987100	3.67815200	-2.78450500
O	4.06154000	4.38497500	-4.30590300
O	2.20731300	2.69949000	-5.22947000

O	4.67371400	2.30397500	1.24643000
O	-5.04086300	0.09132600	-1.71297000
O	-3.19098600	-2.94053700	1.32109200
H	6.42485900	-1.14700700	5.22893800
C	5.44220800	-0.86230900	5.62656800
H	5.30412900	-1.36853400	6.58895400
C	4.30599900	-1.24229600	4.64820500
C	4.41676000	-0.58445200	3.26136200
C	3.26092400	-0.87149000	2.30156100
O	2.32866500	-1.64984600	2.66685300
O	3.32801100	-0.27531800	1.17000400
H	4.75194800	4.34815600	8.69375500
C	3.67909100	4.53489900	8.83294300
C	2.87663600	4.15123300	7.57302900
C	3.02118400	2.66685100	7.18745500
C	2.05966900	2.16873900	6.09669600
N	2.37687300	2.71249500	4.77118700
C	1.80545700	2.30859100	3.61590800
N	0.80685300	1.42284900	3.62051700
N	2.24714600	2.80909500	2.45141300
O	5.96943900	0.03114900	0.02972200
H	-8.65629100	6.65697000	1.43323800
H	-7.00047900	7.09502700	1.89518500
H	-7.96314600	8.19345200	0.88132900
H	-6.29384000	6.97930100	-0.54934700
H	-7.94868400	6.53436500	-0.98697600
H	-8.96462500	4.48650300	0.33642400
H	-8.49799700	2.12751200	0.95835600
H	-4.24612000	2.84287800	0.40304000
H	-4.74070200	5.20807700	-0.21726100
H	-7.21533200	0.02232800	0.50554700
H	-8.60681700	0.41039200	-1.62838900
H	-7.34467800	-2.42307400	1.24866200
H	-0.98769900	-2.92549400	4.69019500
H	-0.43658800	-5.79196000	5.33187400
H	5.62143900	-1.67089800	-6.27503500
H	-7.80081000	-3.29257400	-4.28073400
H	-9.89177300	-4.38745000	-2.50913800
H	-11.02042800	-2.21884400	-2.26728700
H	-9.89783900	-1.42264800	-3.36340900
H	-3.63145500	-2.90323200	-3.80908300
H	-5.38192600	-2.30053700	-2.14547500
H	-5.49350500	-5.29599700	-2.85529700
H	-3.20089000	-4.34777600	-2.89044800

H	-4.90703500	-3.65293600	-1.10884800
H	7.09836400	-4.58965200	2.65748600
H	3.49006100	-3.99876300	1.89034800
H	4.55515400	-3.97065100	-2.22210900
H	1.49608200	-1.38912400	-3.69126400
H	1.22464700	-5.36893700	-2.38178500
H	4.25245600	-3.07893200	-7.42412600
H	7.16961000	-1.01120300	-4.69975900
H	-0.29466000	5.71596900	-1.60342300
H	-0.08243100	7.30156100	0.87622100
H	2.62486000	8.83136000	-0.24257100
H	-0.80504400	8.60226100	-1.00780400
H	7.01554000	4.40917600	0.85260700
H	1.77819500	-3.26950100	-6.91374600
H	2.53156700	-1.84069000	-6.22202300
H	0.76252900	-6.21237200	3.39251200
H	-3.30322700	-1.26343400	4.25506300
C	-2.02556500	-0.15563600	5.59725800
H	-2.28532600	0.83040600	5.19291400
H	-2.65791800	-0.36002100	6.46741300
H	-0.97814300	-0.15365900	5.93336100
H	-2.07530800	-4.97824600	6.69276300
H	-3.21682600	-5.56103600	5.46136900
H	0.69991800	-6.82571300	0.93231700
H	-0.98159800	-6.31552200	0.86034300
H	-2.31036400	4.61579900	-0.33431400
H	-1.56910000	5.25436100	1.13033800
H	5.45999100	0.22090200	5.81071700
H	4.27728000	-2.33418700	4.52548000
H	5.34024900	-0.87164100	2.73690100
H	3.33571900	-0.97041400	5.08842500
H	4.47511500	0.51097100	3.34034900
H	8.28582800	3.34847300	-1.77206300
H	7.60731200	2.36003500	-0.48440000
H	5.14663700	-6.19444300	2.64843000
H	5.86352200	-7.08303100	1.31367900
H	2.97267900	-2.98240600	-0.29935200
H	3.54886200	5.59722800	9.06832100
H	3.19067400	4.79459600	6.73516500
H	2.82338900	2.05220500	8.07676800
H	2.11526300	1.07327700	6.02870600
H	3.34537500	3.95124000	9.70054000
H	1.81106700	4.36970000	7.74176800
H	4.05616700	2.44406000	6.88678000

H	2.95784400	3.54073500	4.72109200
H	1.02729200	2.43645600	6.37640600
H	3.26350700	2.91871600	2.30854100
H	0.26977100	1.22622100	4.45347700
H	1.81190100	2.45749900	1.59019700
H	0.52299800	0.97692000	2.73917400
H	4.97586100	-1.96690800	-2.76033800
H	5.17672600	0.92909200	-3.74352800
H	6.23666900	-0.80515900	-2.36348000
H	4.11603900	-0.24797800	-4.52083700
H	2.20195700	-5.40222200	-4.76055400
H	1.20124700	3.09028900	-3.96928600
H	4.27213500	3.86026500	-1.34825100
H	5.00062200	3.02067900	0.65460500
H	3.93681100	1.91290600	0.70852900
H	5.10293300	-0.29584000	-0.26882300
H	5.71150200	0.78973100	0.59742400
H	-2.81420900	10.11021300	-1.35538700
H	-3.43097300	8.79937600	-2.40122300
H	-4.85112000	-0.13886600	-0.77089900
H	-4.31095900	-0.42458200	-2.12262800
H	-3.77593900	-1.34238100	1.10714700
H	-2.63535900	2.53017800	-1.77885300
H	-2.25496600	4.61608400	-2.66735600
H	-1.03742900	3.60409200	-2.97321400
H	-4.40710700	0.13001200	1.28574400
H	-3.78765900	1.43067900	-1.51535500
H	2.56439700	1.98081100	-4.67295400
H	2.91479000	3.39939400	-5.11318200
H	3.53289500	4.38412100	-3.47571500
H	4.82784100	3.83790900	-4.03424800
H	-3.10230900	-3.63348100	2.00706900
H	-2.27265000	-2.82066300	0.97911100
H	-8.62675300	-3.66363200	-0.57920600
H	6.06447800	-5.63299200	-1.03315600
O	0.27845500	1.29467900	-1.36170200
H	0.10746100	0.90786600	-2.24202200
H	2.47209900	4.79307000	-1.65585400

$S_3^{B,W}Y_Z^{\bullet}$ (W1=H_2O, W2=OH^-, Ox=OH^-)*

Mn	0.33923000	-2.47751900	-0.99771500
Mn	0.75653200	-1.89137500	1.61621100
Mn	1.90450900	-0.16250300	-0.21853300
Mn	1.25104400	2.53549800	-1.83868400
Ca	-1.47830000	0.33153100	-0.02663600

O	-0.64585300	-2.11876600	0.49010900
O	0.73228700	-0.14461500	1.21341100
O	1.83867600	-2.14485900	0.09455100
O	1.93765200	1.57457000	-0.32285600
O	0.55948400	-0.75696000	-1.38742800
O	2.70081000	3.77782200	-1.85722200
O	0.50295300	3.26672100	-3.32453800
O	-3.10180600	1.83389200	-1.04313100
O	-3.80539900	-0.53637700	0.68702500
H	7.46839000	5.72834500	-0.47239000
C	7.55965200	4.78092600	0.07197600
H	8.54711100	4.75518700	0.54752400
C	7.40047700	3.57845500	-0.87127800
C	6.09055500	3.50812700	-1.62744800
O	5.05796700	4.03419200	-0.95673100
O	5.98022100	3.00051400	-2.73897100
C	-7.97545200	6.86605200	1.19589700
C	-7.38515500	6.22287800	-0.08023600
C	-7.02086900	4.79073400	0.17190500
C	-8.05558300	3.83566900	0.38825000
C	-5.66945300	4.37703400	0.27367000
C	-7.76324200	2.53758300	0.71574400
C	-5.34496300	3.08676000	0.63023400
C	-6.38790300	2.11205400	0.86570900
O	-6.11205900	0.90563600	1.17941700
H	1.58223400	8.37711900	1.41422100
C	2.06518300	8.06621600	0.48048300
C	1.24700200	7.07495200	-0.33220100
O	1.70654400	6.49288600	-1.31016300
H	3.04148800	7.62362400	0.70381400
N	-0.06647100	6.93954100	0.06325900
C	-1.04345800	6.12781300	-0.65155600
C	-2.20700200	6.95321000	-1.21821800
O	-3.21532700	6.38498100	-1.66445200
C	-1.51828700	4.91013800	0.16092800
C	-0.60622700	3.67922500	0.07280000
O	-0.84961400	2.67542300	0.76432700
O	0.36477300	3.82337300	-0.76992600
N	-1.99597700	8.28579400	-1.24578200
C	-2.85639400	9.24816400	-1.91185500
H	-2.34775600	9.69069400	-2.77728000
H	-5.63023000	-4.17952400	-4.05382100
C	-5.70990000	-4.42592600	-2.98495200
C	-7.15869000	-4.50708900	-2.57874200

O	-7.51379700	-5.03397300	-1.51161200
C	-4.84013000	-3.42426600	-2.17248600
C	-3.41311200	-3.39339200	-2.76735500
C	-2.37756000	-2.48733800	-2.08450700
O	-2.63953800	-1.32267300	-1.73604000
O	-1.20778700	-3.04075500	-1.97541000
N	-8.04290800	-3.95773300	-3.45586600
C	-9.47005400	-3.86656600	-3.19427400
H	-10.02561800	-4.01908600	-4.12487600
C	-9.86536000	-2.49491100	-2.57988600
C	-8.99467000	-2.04515000	-1.43901600
C	-8.47636400	-0.81284200	-1.12174300
N	-8.55231500	-2.90324900	-0.43780200
C	-7.77557000	-2.23304700	0.42260000
N	-7.73771000	-0.95662500	0.03883200
H	3.92810000	-4.37151400	-6.67766800
C	4.01207600	-3.29073800	-6.51405800
C	4.98928800	-3.07049900	-5.36607500
O	5.00831500	-3.82873600	-4.38276500
C	2.62561800	-2.68608100	-6.14515100
C	2.14952900	-3.02751800	-4.76548600
C	1.69068300	-2.24100200	-3.73621500
N	2.10881400	-4.32696600	-4.27093400
C	1.66457700	-4.30185200	-2.99449500
N	1.40105300	-3.04723900	-2.64787300
N	5.75187200	-1.95799000	-5.45082200
C	6.52632800	-1.45171800	-4.32063800
H	7.14604200	-2.26404800	-3.92652500
C	5.60922200	-0.91078200	-3.17921100
C	4.57538700	0.15497800	-3.59311800
C	3.40749200	0.38344400	-2.62077600
O	3.35664100	-0.33628000	-1.56230300
O	2.54587900	1.25255900	-2.95492300
H	8.06227800	-5.11883400	1.28372500
C	7.38181400	-5.31156200	2.12267500
H	7.88961400	-5.98684400	2.82019100
C	6.05881100	-5.93799400	1.64478600
C	5.29710300	-5.06621700	0.69785800
C	4.24202800	-4.21277800	0.89452000
N	5.60124600	-4.95375700	-0.65815400
C	4.76889500	-4.08067800	-1.26159300
N	3.94466600	-3.62624100	-0.31977200
H	-0.13947400	-7.47447600	2.98343500
C	0.06516900	-6.40738000	2.81308200

C	-1.08101900	-5.61454900	3.45508500
O	-2.05001200	-5.21721200	2.80632300
C	0.17705800	-6.15007300	1.31497400
C	0.51533500	-4.71437200	0.94056200
O	0.78571500	-3.90356400	1.88867700
O	0.48939900	-4.44173900	-0.29918300
N	-0.98770000	-5.41637600	4.80606200
C	-2.15337500	-5.03093400	5.60605900
C	-2.70643700	-3.62061100	5.32511100
O	-3.90264400	-3.38604000	5.44914900
N	-1.77580400	-2.67273000	5.01649600
C	-2.17728200	-1.35081100	4.54390700
C	-1.39460000	-0.99779800	3.27992000
O	-1.77432700	-0.10254100	2.52368000
O	-0.28946300	-1.69413600	3.15894300
O	-2.21420200	3.74117700	-2.75375200
O	3.93534800	4.25568600	-4.29668300
O	2.12197400	2.61321400	-5.36144200
O	4.06989500	2.65283100	1.16795500
O	-5.08734700	-0.00177200	-1.66903300
O	-3.08147600	-3.05708700	1.26434600
H	6.50261300	-0.76358900	5.20575100
C	5.49160500	-0.70621500	5.62933600
H	5.47514000	-1.30191300	6.54958900
C	4.43587300	-1.21148300	4.62822000
C	4.41723300	-0.41862500	3.31544900
C	3.31942500	-0.78397500	2.32438200
O	2.42436400	-1.62729800	2.68625800
O	3.36440900	-0.19385500	1.20334100
H	4.57894100	4.81879000	8.62117500
C	3.54183600	4.58851600	8.89789800
C	2.74083000	4.06543700	7.68551100
C	3.32697700	2.77843400	7.07141400
C	2.43223600	2.05496600	6.05004700
N	2.34262500	2.75111400	4.75938100
C	1.68447100	2.28344900	3.67942300
N	0.98235700	1.15094500	3.73503600
N	1.72400700	2.98241300	2.52461900
O	5.84596300	0.62576200	0.08500600
H	-8.87880800	6.33965500	1.52632300
H	-7.24939200	6.84094000	2.01732400
H	-8.24296000	7.91148300	1.00309800
H	-6.50266900	6.78114500	-0.41549600
H	-8.13286300	6.27817600	-0.88527100

H	-9.09190600	4.15706600	0.29939200
H	-8.55007400	1.81022600	0.90555600
H	-4.31336200	2.74816800	0.66197500
H	-4.88404900	5.09536400	0.04196500
H	-7.17147800	-0.21383900	0.52060700
H	-8.55542600	0.13897800	-1.62570100
H	-7.24707000	-2.66049900	1.26288800
H	-0.85790300	-2.98566000	4.71566300
H	-0.21269600	-5.85403300	5.29023100
H	5.66983900	-1.37727900	-6.27614300
H	-7.67714900	-3.51710800	-4.29104600
H	-9.72392200	-4.68623300	-2.51455300
H	-10.91544200	-2.55069500	-2.25678100
H	-9.82006600	-1.71806500	-3.35326700
H	-3.46742500	-3.05204200	-3.81399600
H	-5.28528900	-2.42151700	-2.19752700
H	-5.29076000	-5.43327600	-2.86087200
H	-2.99716600	-4.40689700	-2.79022700
H	-4.79606300	-3.73464600	-1.12066400
H	7.19888500	-4.35872600	2.63322200
H	3.67103700	-3.96617600	1.77937800
H	4.78179900	-3.83291200	-2.32179200
H	1.51517800	-1.17531800	-3.69130400
H	1.50638300	-5.16448600	-2.36330700
H	4.35874000	-2.84321000	-7.45295800
H	7.18908800	-0.66721800	-4.69918800
H	-0.49883100	5.74584700	-1.52519700
H	-0.34081800	7.34090700	0.95298100
H	2.24579100	8.95574200	-0.13593100
H	-1.09061900	8.59620200	-0.91331200
H	6.79958700	4.77174800	0.86134200
H	1.89361200	-3.01212700	-6.89751300
H	2.67057400	-1.59243900	-6.21458200
H	1.00863700	-6.17371800	3.32289000
H	-3.23908700	-1.40494400	4.27824300
C	-1.98411600	-0.26376600	5.61255300
H	-2.25197600	0.72066000	5.21164300
H	-2.61832000	-0.48519300	6.47722800
H	-0.93981600	-0.24246200	5.95580600
H	-1.86630200	-5.08730300	6.66173500
H	-2.98933300	-5.71878200	5.43641400
H	0.93367600	-6.80843000	0.86683200
H	-0.77316100	-6.38095600	0.81927500
H	-2.48279800	4.57303900	-0.23479300

H	-1.67241700	5.16000800	1.21893400
H	5.30963800	0.34310500	5.90235200
H	4.61395600	-2.27388200	4.40740700
H	5.37104800	-0.48693800	2.77342900
H	3.43650900	-1.16955600	5.08452300
H	4.28647300	0.65809400	3.50833000
H	8.19943900	3.54793500	-1.62065500
H	7.46135800	2.63205400	-0.31173700
H	5.41169500	-6.13854900	2.50754400
H	6.25153000	-6.91164300	1.17203100
H	3.14389300	-2.94959100	-0.39362000
H	3.08732200	5.50236600	9.29676500
H	2.68003500	4.85655100	6.92132400
H	3.50880100	2.05669600	7.88018000
H	2.83426900	1.04968200	5.86165900
H	3.56521400	3.84126600	9.70153600
H	1.70474000	3.86479200	7.99835500
H	4.30957900	2.97726400	6.61727200
H	2.66546900	3.70976700	4.70399900
H	1.42274200	1.93902700	6.47725400
H	2.67083600	3.21412900	2.17056900
H	0.81497200	0.66014600	4.60205000
H	1.13656500	2.60414300	1.77541200
H	0.73231600	0.68638900	2.84897300
H	5.07206500	-1.76143000	-2.75124000
H	5.04615600	1.13793000	-3.72415700
H	6.24729200	-0.50265300	-2.38481800
H	4.11066700	-0.10396800	-4.55621000
H	2.51062600	-5.13472400	-4.73011900
H	1.07281100	3.16076600	-4.15166700
H	4.14272000	3.88632800	-1.42890200
H	4.50208600	3.30744500	0.57188200
H	3.40040400	2.21406900	0.58133400
H	5.11604300	0.09832500	-0.28322700
H	5.35589900	1.26243400	0.64903600
H	-3.15067900	10.04734500	-1.22108200
H	-3.74900300	8.71907900	-2.25578600
H	-4.83945200	-0.26784800	-0.74844800
H	-4.35209400	-0.45244200	-2.13720700
H	-3.69414100	-1.45009500	1.06789300
H	-2.79198200	2.52481100	-1.69111400
H	-2.53173400	4.65206100	-2.59046000
H	-1.26488700	3.77523300	-3.03014200
H	-4.35458400	-0.00579400	1.29769200

H	-3.91461400	1.41009000	-1.40977900
H	2.37647300	1.89403900	-4.75142100
H	2.85591500	3.27342500	-5.19752600
H	3.37697900	4.20543300	-3.48471100
H	4.75099000	3.80318100	-3.99792500
H	-2.97675100	-3.74565000	1.95226500
H	-2.16249200	-2.89767300	0.94409400
H	-8.49178900	-3.93041800	-0.57062300
H	6.34299300	-5.45279700	-1.13699600
O	-0.25570900	1.47574900	-1.81297600
H	-0.65819600	1.67080400	-2.67846700
H	2.28991400	4.65684500	-1.68974900

$S_3^{A,W}Y_Z$ (W1=OH$^-$, W2=OH$^-$, Ox=OH$^-$)*

Mn	-0.30594000	-2.56212200	-1.07750900
Mn	0.36982700	-1.80360200	1.52898900
Mn	1.92381000	-0.06416200	-0.04664600
Mn	2.07213900	2.53121300	-1.54328300
Ca	-1.52603800	0.39995200	-0.23102500
O	-1.07239200	-1.91296400	0.49394900
O	0.63168700	-0.04343900	1.23360100
O	1.31777000	-1.99872000	-0.11921800
O	2.56202500	1.57268500	0.06650700
O	-0.54577600	-1.17616100	-2.00728400
O	3.52561400	3.55008900	-1.41421000
O	1.33875400	3.13888000	-3.06248800
O	-2.69641100	1.84005300	-1.69719000
O	-3.30357000	0.14640400	1.40334800
H	6.96761700	4.35376800	0.14729300
C	7.84720900	3.86880900	-0.29301100
H	8.65069800	4.61604000	-0.34438600
C	7.52578000	3.31931400	-1.67942200
C	6.45159700	2.23115900	-1.69365500
O	5.74521900	2.08636300	-0.61991500
O	6.28451200	1.57013300	-2.74271200
C	-7.35084300	7.64299600	1.02116800
C	-6.56254700	6.96757600	-0.11536900
C	-6.34328900	5.47980900	0.08278200
C	-7.42280700	4.59124600	0.18167800
C	-5.04654600	4.94687000	0.14923000
C	-7.22638700	3.21673800	0.33022600
C	-4.83007300	3.57477800	0.28720900
C	-5.92267400	2.70854100	0.37085900
O	-5.67779200	1.35132700	0.46490000
H	2.50651900	7.73924200	1.17525200

C	2.74456500	7.73596700	0.10474900
C	1.54437200	7.39805800	-0.76430700
O	1.47969400	7.76022900	-1.94516900
H	3.52849900	6.98779300	-0.07519500
N	0.54830500	6.68892000	-0.16392600
C	-0.57512800	6.11156400	-0.91636500
C	-1.66303900	7.20251100	-1.12533100
O	-2.73887600	7.18397300	-0.52315700
C	-1.14190300	4.89306600	-0.19018500
C	-0.24481100	3.65049600	-0.12715700
O	-0.69081100	2.61784800	0.39535300
O	0.93798600	3.80015100	-0.64362500
N	-1.29584600	8.15775600	-2.01879900
C	-2.06823700	9.37087500	-2.24153300
H	-2.18020900	9.56140300	-3.31601100
H	-6.27265100	-3.69346700	-3.91473100
C	-6.38812400	-3.94237700	-2.84889100
C	-7.85399700	-3.92950900	-2.46978300
O	-8.27616900	-4.49245300	-1.45128900
C	-5.49998900	-3.01798200	-2.01580000
C	-4.08835300	-3.15226200	-2.59008200
C	-3.01071800	-2.50328600	-1.75902600
O	-3.16493000	-1.35857500	-1.29684300
O	-1.96454700	-3.25981300	-1.62272000
N	-8.67499700	-3.25024900	-3.32524600
C	-10.07054000	-2.98822000	-3.01358400
H	-10.67678900	-3.08752100	-3.92182600
C	-10.29221600	-1.58628700	-2.38890500
C	-9.31317500	-1.21838400	-1.31324700
C	-8.63135000	-0.04757700	-1.06512600
N	-8.91468900	-2.08254600	-0.30205700
C	-8.00425500	-1.43293700	0.47075600
N	-7.82777800	-0.19001300	0.05076900
H	3.08099700	-5.02185200	-6.67852100
C	3.32839100	-3.96012100	-6.57005600
C	4.47035000	-3.86925900	-5.56277700
O	4.66682800	-4.76314500	-4.72548400
C	2.09031700	-3.16031500	-6.10866000
C	1.66371000	-3.41543700	-4.69128200
C	0.83438200	-2.69274000	-3.87094400
N	2.03544300	-4.53324200	-3.94819100
C	1.45698700	-4.44453100	-2.72736400
N	0.71676700	-3.34580400	-2.66075600
N	5.19786900	-2.72679900	-5.58802100

C	6.06742800	-2.33983500	-4.47546600
H	6.64656000	-3.22165300	-4.18570900
C	5.27037300	-1.80540900	-3.26239500
C	4.35814900	-0.63164100	-3.62620800
C	3.56724500	0.01599700	-2.49242200
O	3.28722600	-0.65534900	-1.45013600
O	3.17065300	1.19327400	-2.73706400
H	7.42253800	-5.40435800	1.72241300
C	6.63124300	-6.08304700	2.06797000
H	7.11382000	-7.02211800	2.36139600
C	5.58053600	-6.34137100	0.97275200
C	4.84563300	-5.10954100	0.54785700
C	3.50996900	-4.82541000	0.41122900
N	5.49961700	-3.93590600	0.17842700
C	4.60413200	-2.99200300	-0.16427200
N	3.39208300	-3.52189900	-0.03584000
H	-1.05828700	-7.41118400	3.04824900
C	-0.74570200	-6.36589300	2.91150300
C	-1.82010500	-5.48068000	3.55457200
O	-2.80157700	-5.07963000	2.92860400
C	-0.60322900	-6.07323200	1.41771600
C	-0.09555400	-4.68154900	1.03549900
O	0.14679300	-3.83800800	1.95160200
O	0.03926100	-4.47229000	-0.20741400
N	-1.65669300	-5.21034300	4.88433900
C	-2.74918100	-4.67972500	5.69720600
C	-3.15838800	-3.22174700	5.43623700
O	-4.27469700	-2.84035900	5.77770100
N	-2.18668400	-2.44430600	4.90844400
C	-2.40029900	-1.05005900	4.52325700
C	-1.12471700	-0.58295800	3.79361900
O	-0.69896900	0.56344300	3.89954300
O	-0.59489500	-1.56718500	3.10927800
O	-1.28634900	3.56125400	-3.21440400
O	4.87348800	3.79998200	-3.89588300
O	2.87892100	2.48771700	-5.15449100
O	6.36172500	1.92976400	2.08286800
O	-4.99630700	0.49623200	-2.17521400
O	-3.50509600	-2.59212400	1.51957300
H	5.58182400	-2.25722200	5.61763500
C	5.31353300	-1.20260400	5.46253200
H	5.13930000	-0.75586000	6.45033800
C	4.08524400	-1.06702500	4.57039700
C	4.32627100	-1.69938400	3.18997500

C	3.13684700	-1.45369700	2.29303600
O	2.05739900	-2.03003600	2.59290000
O	3.29908100	-0.66470600	1.30176400
H	4.69316000	3.56463600	8.91098800
C	4.00544500	4.36239000	8.59918400
C	3.47807300	4.11625800	7.17193300
C	2.70776300	2.79090800	7.02000600
C	2.03012800	2.57792100	5.65782500
N	3.01878500	2.37613600	4.59241400
C	2.70342400	2.33949300	3.26842100
N	1.42508900	2.21647400	2.87882900
N	3.66727800	2.47665100	2.35964400
O	5.87132800	-0.34917300	0.45127000
H	-8.34612300	7.19446700	1.13927700
H	-6.82344800	7.53467000	1.97775200
H	-7.48586000	8.71468600	0.82298700
H	-5.58493000	7.45472000	-0.22804700
H	-7.10102200	7.13343400	-1.06241200
H	-8.44246900	4.97396100	0.13518300
H	-8.07493700	2.54088100	0.41372000
H	-3.82251500	3.16693700	0.29956300
H	-4.19381200	5.61878500	0.07013200
H	-6.54533400	0.82211600	0.44305600
H	-8.66416600	0.88439100	-1.61557700
H	-7.49086500	-1.89954000	1.30182800
H	-1.40509400	-2.90144900	4.44830100
H	-0.84049800	-5.58206800	5.35293900
H	4.93371300	-2.00759600	-6.25072900
H	-8.24029200	-2.70619300	-4.06106500
H	-10.37688400	-3.77468700	-2.31704800
H	-11.32800500	-1.54552100	-2.01593800
H	-10.21946800	-0.81999400	-3.17227500
H	-4.04843500	-2.68383700	-3.58666800
H	-5.84319200	-1.97740400	-2.07083500
H	-6.04274200	-4.97672200	-2.72250700
H	-3.82777900	-4.20844700	-2.72238000
H	-5.51668600	-3.31873600	-0.96084300
H	6.17039600	-5.63741300	2.95785000
H	2.63852400	-5.43533200	0.59669300
H	4.84705100	-1.96891300	-0.42372700
H	0.30494400	-1.76515200	-4.03366300
H	1.57447200	-5.16073600	-1.92924800
H	3.63930100	-3.59217300	-7.55649500
H	6.75934000	-1.57495100	-4.84522600

H	-0.17756800	5.80154100	-1.89095100
H	0.72472300	6.30778800	0.75747200
H	3.12949600	8.71556700	-0.19164800
H	-0.31924000	8.12497500	-2.32674100
H	8.16639400	3.07608100	0.39386700
H	1.26545700	-3.39122800	-6.79833300
H	2.28078200	-2.08423000	-6.21906700
H	0.21271900	-6.23804400	3.43239100
H	-3.20667500	-0.99734600	3.77659100
C	-2.73879900	-0.15149100	5.70922600
H	-2.85908900	0.87927500	5.36166100
H	-3.66437300	-0.49573700	6.17760100
H	-1.92951100	-0.17527200	6.45036000
H	-2.45515900	-4.76428500	6.74935800
H	-3.65533900	-5.27800600	5.54806000
H	0.04859500	-6.81430800	0.93555300
H	-1.58472300	-6.16577900	0.93568200
H	-2.05144100	4.56299900	-0.70437100
H	-1.46234300	5.15295200	0.82727500
H	6.18207000	-0.69913600	5.01646300
H	3.21006900	-1.54390200	5.03072500
H	4.45609100	-2.78575800	3.29834600
H	3.83441600	-0.00589500	4.44306200
H	5.22831100	-1.28552000	2.72772300
H	7.16175800	4.11860400	-2.34276600
H	8.41857000	2.90851300	-2.16981800
H	4.83970400	-7.06815600	1.32787700
H	6.06746700	-6.80015000	0.09856400
H	2.47386300	-2.93775500	-0.13733300
H	4.54438300	5.31550800	8.65880000
H	4.32552300	4.14766400	6.46871200
H	1.91525300	2.75304000	7.78061400
H	1.37730000	1.69623200	5.70314300
H	3.17910100	4.39331200	9.32179400
H	2.81443700	4.94434400	6.88066300
H	3.37451000	1.93944200	7.22878300
H	3.96695500	2.67190000	4.79258600
H	1.39112300	3.44074400	5.41301500
H	4.67638000	2.35972000	2.56190300
H	0.73350100	1.71284300	3.44534100
H	3.39919000	2.20853800	1.39618300
H	1.28077600	2.04370300	1.88261500
H	4.67994000	-2.62906800	-2.84304700
H	4.94286200	0.17403800	-4.07893300

H	5.98714700	-1.47297600	-2.49985800
H	3.59677200	-0.96195100	-4.35065300
H	2.84201100	-5.11866000	-4.18368700
H	1.84593500	2.93690200	-3.91285700
H	4.23692900	2.98910100	-0.98463200
H	6.27028600	2.33312500	1.18542800
H	6.35943400	0.98178000	1.81950600
H	4.94631200	-0.33855900	0.78480700
H	5.85587800	0.45662400	-0.14006600
H	-1.58488600	10.24235200	-1.77707200
H	-3.05508300	9.22998800	-1.79238100
H	-5.31758500	0.79929400	-1.29727900
H	-4.43030100	-0.27671000	-1.93845300
H	-3.52222500	-0.81419800	1.53069600
H	-2.27146100	2.50516600	-2.29687400
H	-1.40633800	4.51153700	-3.36152300
H	-0.29909400	3.40608700	-3.18913000
H	-4.15583300	0.60216300	1.21741000
H	-3.54815000	1.52568600	-2.09680600
H	3.08380500	1.72172400	-4.58328700
H	3.62006200	3.10539300	-4.88973600
H	4.34211700	3.89682800	-3.07052300
H	5.42097100	3.00696800	-3.69934000
H	-3.41753600	-3.26368600	2.22411200
H	-2.60851300	-2.55655000	1.10881800
H	-9.00904300	-3.10013000	-0.35237200
H	6.49944200	-3.77499300	0.19656800
O	0.79135400	0.69494600	-1.30595800
H	0.47095600	-0.05572800	-1.91593200

$S_3{}^{TS}Y_z$ (W1=OH$^-$, W2=OH$^-$, Ox=OH$^-$)*

Mn	-0.12458800	-2.57057800	-1.02685400
Mn	0.37552200	-1.87996300	1.57040400
Mn	1.74271300	-0.20004200	-0.25295400
Mn	1.83959300	2.64748000	-1.46158000
Ca	-1.52456600	0.41860700	-0.22430400
O	-1.03950000	-2.05259100	0.49028700
O	0.54021700	-0.13784300	1.11074200
O	1.40048400	-2.23348300	0.05044900
O	2.40446800	1.42614900	-0.08764100
O	0.09299800	-0.97292600	-1.58205800
O	3.41902400	3.51083300	-1.36525800
O	1.22554500	3.50964200	-2.92364900
O	-2.75978200	1.89107400	-1.60951900
O	-3.31519000	0.00026700	1.36619800

H	6.90156400	4.36323700	0.42979200
C	7.79238700	4.00095700	-0.09804600
H	8.55052900	4.79460900	-0.05687500
C	7.45999800	3.64200400	-1.54305300
C	6.46467400	2.49239300	-1.71571500
O	5.79258100	2.12062000	-0.67854700
O	6.32862100	2.01029600	-2.86322700
C	-7.48757000	7.47984200	1.07346600
C	-6.67513100	6.82737000	-0.05945500
C	-6.43536300	5.34278300	0.13494000
C	-7.50276900	4.43609400	0.19166800
C	-5.13312500	4.83186000	0.24634500
C	-7.28872300	3.06460200	0.34123600
C	-4.89866800	3.46298400	0.38936700
C	-5.97895900	2.57848600	0.42816100
O	-5.71765200	1.22298100	0.52125700
H	2.36710700	7.74193000	1.35786100
C	2.61533200	7.76885100	0.28994700
C	1.42676800	7.43980300	-0.59864900
O	1.36491300	7.84066200	-1.76649200
H	3.40973300	7.03484000	0.09782100
N	0.43924300	6.69675400	-0.02399500
C	-0.66089000	6.10014100	-0.79804100
C	-1.76522100	7.17265100	-1.02283900
O	-2.85448900	7.12215100	-0.44532800
C	-1.24521400	4.89670600	-0.05751400
C	-0.33897100	3.67507800	0.10224900
O	-0.78235600	2.65758800	0.64941600
O	0.87387500	3.83379800	-0.36438100
N	-1.40304700	8.14880700	-1.89323200
C	-2.19623500	9.35129200	-2.09452900
H	-2.29065800	9.57317300	-3.16468200
H	-6.15543400	-3.81021000	-4.02655500
C	-6.26423800	-4.02733000	-2.95316500
C	-7.72933100	-4.02850900	-2.57375100
O	-8.14857400	-4.59925400	-1.55908400
C	-5.36851500	-3.05239200	-2.15084500
C	-3.94266300	-3.14029500	-2.72580000
C	-2.84056300	-2.39847900	-1.97501700
O	-3.01119400	-1.25290300	-1.51355900
O	-1.73042500	-3.06356500	-1.92707300
N	-8.55473400	-3.36837100	-3.44023500
C	-9.96103500	-3.13752600	-3.15176300
H	-10.54757600	-3.24085800	-4.07226700

C	-10.21944400	-1.74444900	-2.52015600
C	-9.27340200	-1.37193300	-1.41635300
C	-8.60798200	-0.19715900	-1.14260500
N	-8.89998500	-2.23756200	-0.39665600
C	-8.01789700	-1.58471500	0.40568700
N	-7.83768100	-0.33875000	-0.00289100
H	3.23705200	-4.87215900	-6.65851500
C	3.49764200	-3.81411300	-6.54747700
C	4.64551400	-3.73235700	-5.54141900
O	4.82993500	-4.63210600	-4.70788700
C	2.27909100	-2.99717800	-6.06174500
C	1.86395300	-3.27656000	-4.64628700
C	1.08927500	-2.54853800	-3.77871800
N	2.17969700	-4.45053000	-3.96504700
C	1.61778300	-4.39282100	-2.73470000
N	0.94221500	-3.26005300	-2.60612700
N	5.37715500	-2.58932400	-5.55376600
C	6.17971700	-2.17592700	-4.39388200
H	6.76267500	-3.04412400	-4.07257100
C	5.27962100	-1.67706900	-3.23982500
C	4.48778600	-0.41468100	-3.59347800
C	3.38886400	0.02921800	-2.63277000
O	3.10003700	-0.70637600	-1.62349400
O	2.81482600	1.11384200	-2.92044500
H	7.39530600	-5.14206000	1.97830700
C	6.72835500	-6.00537600	2.10077200
H	7.34523100	-6.86475900	2.38890500
C	5.95032700	-6.30411900	0.80733700
C	5.08980500	-5.15463500	0.40168100
C	3.73226500	-4.96412500	0.42872700
N	5.61647600	-3.93298900	-0.01243500
C	4.62373400	-3.05006600	-0.22848100
N	3.47752500	-3.66546000	0.03302300
H	-0.93687500	-7.48537400	2.99753900
C	-0.65232600	-6.43403600	2.84399600
C	-1.74743600	-5.56863900	3.48070800
O	-2.71935900	-5.16297600	2.84321600
C	-0.51407300	-6.15704500	1.35373200
C	-0.05398800	-4.74866200	0.98566900
O	0.24229700	-3.93716300	1.91998800
O	-0.01369000	-4.51414600	-0.26042100
N	-1.60962400	-5.32291300	4.81888200
C	-2.72161700	-4.82599600	5.62782000
C	-3.15802600	-3.37373300	5.37319900

O	-4.29665200	-3.02344900	5.66996900
N	-2.18318100	-2.56315000	4.90194700
C	-2.42400400	-1.17354200	4.51182100
C	-1.16230300	-0.67656100	3.79705100
O	-0.75845600	0.47649300	3.90071100
O	-0.61454800	-1.65117600	3.11322000
O	-1.40721200	3.62716300	-3.14982500
O	4.78947700	4.25870800	-3.67672900
O	2.82603800	2.96440200	-4.98147800
O	6.29571100	1.77990700	1.98724600
O	-5.04123700	0.50674000	-2.16351600
O	-3.50368000	-2.73967900	1.36514600
H	5.98196400	-2.00617700	5.29634800
C	5.27851100	-1.20071100	5.54926300
H	5.03843300	-1.28636200	6.61648200
C	4.00498900	-1.28002100	4.69105400
C	4.30679500	-1.14579000	3.19330000
C	3.08526500	-1.19999700	2.28252700
O	2.05770900	-1.81473200	2.68273100
O	3.21259100	-0.63043000	1.14449900
H	4.52387800	3.51868000	9.10331700
C	3.82924400	4.29246900	8.74971800
C	3.43623100	4.05952300	7.26994800
C	2.74057800	2.70551800	7.01225700
C	2.07158000	2.52732800	5.63483100
N	3.03345700	2.28359300	4.54800000
C	2.66521300	2.20174100	3.23609900
N	1.37519800	2.06886200	2.89924000
N	3.58535900	2.29708000	2.27649500
O	5.78684500	-0.37263100	0.19886700
H	-8.47541900	7.01171000	1.17729400
H	-6.96837200	7.37353500	2.03476500
H	-7.64021000	8.55048500	0.88236500
H	-5.70452000	7.33124400	-0.15791700
H	-7.20609000	6.98982800	-1.01117000
H	-8.52621600	4.80246300	0.10956600
H	-8.12802400	2.37393200	0.38879200
H	-3.88482800	3.07471700	0.43727100
H	-4.28957000	5.51803700	0.19413700
H	-6.57660100	0.68470900	0.45901000
H	-8.62994300	0.73696200	-1.68982200
H	-7.52760500	-2.05147100	1.25049500
H	-1.37731600	-2.99899200	4.46438000
H	-0.80376700	-5.70923300	5.29388700

H	5.11451200	-1.86947200	-6.21645600
H	-8.12297400	-2.81858800	-4.17349200
H	-10.26522500	-3.93542900	-2.46727900
H	-11.26465900	-1.72200400	-2.17285000
H	-10.13832600	-0.97056800	-3.29515700
H	-3.94176800	-2.73624400	-3.75145200
H	-5.74700500	-2.02450500	-2.22262600
H	-5.89923800	-5.05028600	-2.79463200
H	-3.63198700	-4.18861000	-2.80282300
H	-5.36701700	-3.32882800	-1.08898100
H	6.03938900	-5.78143600	2.92398200
H	2.93621600	-5.63808500	0.71000800
H	4.76012800	-2.01439100	-0.51542900
H	0.63263300	-1.57539300	-3.88440700
H	1.70005700	-5.15594500	-1.97603500
H	3.80222400	-3.44308900	-7.53472800
H	6.87042500	-1.39284100	-4.72478600
H	-0.24636000	5.77715900	-1.76131400
H	0.64022800	6.27059300	0.87234000
H	2.99139900	8.76005500	0.02227300
H	-0.41861600	8.14503100	-2.17907800
H	8.17566400	3.13672900	0.45773800
H	1.44190800	-3.19825700	-6.74605600
H	2.49074500	-1.92342500	-6.15005300
H	0.30125500	-6.27373200	3.36443000
H	-3.22368900	-1.14199100	3.75562600
C	-2.80472900	-0.28589400	5.69351600
H	-2.95358800	0.74118600	5.34579900
H	-3.72552200	-0.65918300	6.14922100
H	-2.00475200	-0.28560200	6.44489500
H	-2.43328300	-4.91382300	6.68124900
H	-3.61426400	-5.44104500	5.46558500
H	0.17532500	-6.87328400	0.88560200
H	-1.48415200	-6.29202000	0.86066100
H	-2.11682600	4.53002700	-0.61182500
H	-1.63105000	5.19177000	0.92661000
H	5.80006200	-0.24632800	5.39388900
H	3.47776200	-2.22519800	4.87284400
H	4.98186200	-1.95339400	2.86476400
H	3.31370200	-0.47735200	4.98193400
H	4.83297000	-0.20858800	2.98360600
H	7.02031900	4.50320700	-2.06847100
H	8.36279600	3.37763400	-2.10987200
H	5.30707000	-7.18104000	0.94894000

H	6.64859000	-6.55798600	-0.00366900
H	2.52355300	-3.13021600	0.02572000
H	4.31595700	5.26717300	8.87247200
H	4.33478400	4.15082500	6.63965600
H	1.94033100	2.58319200	7.75627600
H	1.38952900	1.66821100	5.68131600
H	2.94204900	4.26915900	9.39623400
H	2.75926800	4.86607400	6.95013700
H	3.44293000	1.87502800	7.18412900
H	3.98817700	2.58506400	4.70504700
H	1.45865000	3.41110700	5.39762900
H	4.60527400	2.20827700	2.43499200
H	0.68849800	1.61132400	3.50584900
H	3.27452600	2.01796700	1.33131200
H	1.17571000	1.88704900	1.91393100
H	4.60340600	-2.49471200	-2.95991300
H	5.16422400	0.45160500	-3.64314300
H	5.91537200	-1.45826100	-2.37143100
H	3.99845300	-0.51627500	-4.57397600
H	2.97375800	-5.04266300	-4.22422700
H	1.79410500	3.39145000	-3.75320900
H	4.10970500	2.92317800	-0.96476000
H	6.22751200	2.22685600	1.10418700
H	6.31991100	0.84771400	1.67420200
H	4.86340700	-0.32322800	0.53236000
H	5.82926300	0.46462400	-0.34882900
H	-1.74046500	10.21791400	-1.59412400
H	-3.18852700	9.17676300	-1.67009300
H	-5.35022300	0.72875000	-1.25785100
H	-4.41264300	-0.23460800	-2.00249300
H	-3.52635200	-0.96703300	1.45071900
H	-2.32475900	2.56055600	-2.20186700
H	-1.61333200	4.56345900	-3.29299000
H	-0.40517000	3.56837200	-3.09775900
H	-4.17247900	0.46038000	1.21645400
H	-3.61917500	1.60731300	-2.00958800
H	2.99949500	2.13773600	-4.48470800
H	3.57982900	3.54908300	-4.67893600
H	4.25417800	4.17163100	-2.84874300
H	5.41059600	3.49970800	-3.60467000
H	-3.43649800	-3.40611200	2.07718700
H	-2.58575200	-2.68884500	1.00446000
H	-8.97494900	-3.25560300	-0.46075500
H	6.59822700	-3.70753900	-0.12151300

O	0.39103700	1.37669600	-1.48568000
H	0.36489500	0.99035800	-2.38020000

$S_3^{B,W}Y_z$ (W1=OH⁻, W2=OH⁻, Ox=OH⁻)*

Mn	0.03582200	-2.52811900	-0.95799000
Mn	0.47146000	-1.88917800	1.65541900
Mn	1.75349900	-0.32497600	-0.24088900
Mn	1.48143200	2.53422600	-1.77781100
Ca	-1.49547000	0.45220900	-0.26035200
O	-0.92848600	-2.08020700	0.50019100
O	0.57130000	-0.19132200	1.19398000
O	1.53229500	-2.28589600	0.14076100
O	1.95740600	1.38817500	-0.29466000
O	0.36153200	-0.81765400	-1.38390400
O	3.13509300	3.30458500	-1.67564400
O	1.00581700	3.44864200	-3.26705500
O	-2.95799300	1.91141000	-1.50318900
O	-3.25771900	-0.08746200	1.34407900
H	6.76809500	4.61748700	0.56336600
C	7.66457400	4.30241500	0.01580600
H	8.37990700	5.13529500	0.03716000
C	7.32193900	3.91950800	-1.42504100
C	6.40633100	2.70187700	-1.59123400
O	5.68663600	2.34609300	-0.58436200
O	6.38188200	2.14526500	-2.71508800
C	-7.74088300	7.21099100	1.09459900
C	-6.89751800	6.58532100	-0.03083400
C	-6.61303200	5.11098600	0.17550200
C	-7.64976900	4.16763500	0.18164000
C	-5.30143500	4.64766600	0.35910400
C	-7.39642800	2.80499700	0.34907100
C	-5.02856500	3.28872800	0.52595800
C	-6.07703400	2.36610000	0.50936500
O	-5.77277100	1.02016300	0.61909700
H	2.12086600	7.74817300	1.45287800
C	2.34996200	7.87534800	0.38796200
C	1.19316000	7.49402400	-0.52046100
O	1.13560300	7.88830600	-1.69103100
H	3.21392400	7.24525900	0.13901900
N	0.23188900	6.71116900	0.03797100
C	-0.85478000	6.09440200	-0.73436000
C	-1.99539500	7.12839900	-0.94903900
O	-3.08995300	7.03090900	-0.38715500
C	-1.38183200	4.86337000	-0.00690800
C	-0.44316600	3.66325100	0.00634000

O	-0.76813500	2.64046800	0.63825800
O	0.64235600	3.83216300	-0.68569200
N	-1.65791100	8.13012600	-1.80214600
C	-2.49916400	9.29516700	-2.02266200
H	-2.60900900	9.49405800	-3.09627700
H	-5.95593700	-4.00271600	-4.06537600
C	-6.06670600	-4.21803400	-2.99194300
C	-7.53301400	-4.27211900	-2.62220400
O	-7.93731900	-4.86472700	-1.61427400
C	-5.21081700	-3.20110700	-2.18367400
C	-3.75480600	-3.26587800	-2.68960100
C	-2.69861200	-2.40427300	-1.98784100
O	-2.94595500	-1.27138200	-1.53159300
O	-1.52255500	-2.95162900	-1.98800700
N	-8.37595300	-3.63941800	-3.49188600
C	-9.79218300	-3.46322200	-3.21266100
H	-10.36678200	-3.57675600	-4.13939300
C	-10.10467600	-2.08674500	-2.56834400
C	-9.18849900	-1.69310800	-1.44607700
C	-8.57374500	-0.49696300	-1.14642400
N	-8.80285200	-2.55504100	-0.42771600
C	-7.96278100	-1.87766500	0.39927900
N	-7.82178800	-0.62122500	0.00773100
H	3.46656900	-4.68694800	-6.62369000
C	3.70640400	-3.62426600	-6.51221300
C	4.84630200	-3.51351000	-5.49935600
O	5.04928700	-4.41097100	-4.66756800
C	2.47344300	-2.82621100	-6.01797200
C	2.06499300	-3.11298300	-4.60011400
C	1.29136900	-2.39793500	-3.71757400
N	2.38090600	-4.29916600	-3.94010500
C	1.82044200	-4.26452200	-2.70887700
N	1.14501500	-3.13436700	-2.55781900
N	5.53759000	-2.34544700	-5.49929700
C	6.31088700	-1.90236800	-4.32895400
H	6.94949400	-2.73673000	-4.02256000
C	5.38411800	-1.48105400	-3.17270700
C	4.52698700	-0.25859300	-3.51014200
C	3.30595100	0.00044100	-2.64478200
O	3.18272100	-0.66988800	-1.55215200
O	2.50570000	0.87978800	-3.05327800
H	7.50488600	-4.80176800	2.18241400
C	6.95299700	-5.74983100	2.14646800
H	7.64037200	-6.54970800	2.44702700

C	6.38706100	-6.01457900	0.74085500
C	5.44602800	-4.93136300	0.33681800
C	4.07810000	-4.85433800	0.35264400
N	5.87647300	-3.65025300	-0.00026900
C	4.82017100	-2.83879200	-0.18517300
N	3.72525300	-3.55999800	0.02404000
H	-0.64525800	-7.51514100	3.00204400
C	-0.41212300	-6.45360800	2.83217800
C	-1.54146900	-5.62982700	3.46556500
O	-2.52264700	-5.25463300	2.82357100
C	-0.30043000	-6.18867200	1.33495300
C	0.08185900	-4.75468700	0.97876500
O	0.36061000	-3.96277300	1.92985400
O	0.07973700	-4.48703000	-0.26747500
N	-1.42137700	-5.38521300	4.80608200
C	-2.55910500	-4.94007800	5.61019800
C	-3.05676100	-3.50675400	5.35528200
O	-4.21886200	-3.21016900	5.61604900
N	-2.10282100	-2.65007000	4.92448200
C	-2.38783900	-1.27225300	4.51842200
C	-1.13202300	-0.73558600	3.83499800
O	-0.77160400	0.42825200	3.94119700
O	-0.53317800	-1.70127100	3.17433800
O	-1.63848200	3.82609600	-3.05005800
O	4.64967500	4.09000300	-3.81871600
O	2.76303300	2.71442700	-5.15023600
O	6.01831100	2.13808500	2.08020400
O	-5.12092800	0.36613500	-2.09072200
O	-3.37450600	-2.84408300	1.34803300
H	6.07717400	-1.73988400	5.30510400
C	5.30282900	-1.02333900	5.61291100
H	5.09142300	-1.19403500	6.67613600
C	4.03666300	-1.17455900	4.76168600
C	4.32819900	-0.91820300	3.28325000
C	3.14229700	-1.06481400	2.34452300
O	2.15516800	-1.76535200	2.72843100
O	3.24938700	-0.48776400	1.21427000
H	4.46947200	3.73805000	9.10444200
C	3.62973800	4.39276600	8.83547500
C	3.24245400	4.22945000	7.33792900
C	2.79779100	2.80076300	6.93929500
C	2.04328900	2.64034100	5.59413300
N	2.90799500	2.45275900	4.41399900
C	2.42182700	2.37792100	3.14015000

N	1.10161600	2.26455600	2.92903900
N	3.24637400	2.47291100	2.09743100
O	5.83090300	-0.12596300	0.35159900
H	-8.71164500	6.70694300	1.19178700
H	-7.22514100	7.12450000	2.05974100
H	-7.93043100	8.27534200	0.90098900
H	-5.94314200	7.12046600	-0.12271300
H	-7.42634200	6.72605800	-0.98701100
H	-8.67940400	4.49844100	0.04374500
H	-8.21243700	2.08540000	0.35452900
H	-4.00589000	2.93782700	0.62912500
H	-4.47972300	5.36195900	0.34124200
H	-6.60936600	0.45344200	0.53389000
H	-8.61933000	0.44115300	-1.68522700
H	-7.47317400	-2.33466100	1.24982800
H	-1.26728900	-3.05341500	4.51245200
H	-0.60904000	-5.75238200	5.28549300
H	5.25570300	-1.63391400	-6.16304900
H	-7.95982000	-3.06869700	-4.21816000
H	-10.07321500	-4.27905000	-2.53955100
H	-11.15483000	-2.10308800	-2.23579600
H	-10.03912800	-1.30206600	-3.33394500
H	-3.72612000	-2.96547200	-3.75030500
H	-5.61210800	-2.18547800	-2.29575700
H	-5.66538700	-5.22562500	-2.82307000
H	-3.39143100	-4.29983000	-2.65631400
H	-5.24628400	-3.44966100	-1.11548000
H	6.14326300	-5.69335300	2.88370200
H	3.33728100	-5.60441400	0.58878000
H	4.87582900	-1.78386000	-0.42489200
H	0.85329100	-1.41271500	-3.80037500
H	1.89798500	-5.04572200	-1.96808300
H	4.00142300	-3.24344800	-7.49838100
H	6.95095500	-1.07216200	-4.64638000
H	-0.44662900	5.79513000	-1.70507400
H	0.38352300	6.36219900	0.97596000
H	2.62322400	8.91670300	0.19322300
H	-0.67529800	8.15699800	-2.09141000
H	8.10600600	3.46084100	0.56413400
H	1.63573600	-3.04000200	-6.69774300
H	2.66671100	-1.74918100	-6.10518100
H	0.53692400	-6.23969700	3.34038100
H	-3.16836000	-1.27877500	3.74140900
C	-2.83678200	-0.39455400	5.68316900

H	-3.02358700	0.62266700	5.32479300
H	-3.75143300	-0.80650900	6.11773600
H	-2.05726300	-0.35381700	6.45448600
H	-2.27229400	-5.01841200	6.66483500
H	-3.42358000	-5.59243600	5.44117300
H	0.42913900	-6.86708300	0.87109300
H	-1.26295100	-6.38220200	0.84735200
H	-2.28288100	4.51042400	-0.52180800
H	-1.69677600	5.09888700	1.01778100
H	5.72946500	-0.01575600	5.51105900
H	3.60646000	-2.17674900	4.88447100
H	5.10769800	-1.60811700	2.91989600
H	3.26882300	-0.46436600	5.09905200
H	4.73391900	0.08927100	3.13670200
H	6.81041700	4.74984000	-1.93616500
H	8.22866300	3.72304800	-2.01165700
H	5.84822400	-6.96959900	0.72370000
H	7.20338900	-6.10275900	0.00981200
H	2.74364300	-3.11474900	0.04185900
H	3.92515600	5.42781800	9.04305100
H	4.08492400	4.55172300	6.70746100
H	2.10462100	2.43899600	7.71234600
H	1.39356000	1.75823100	5.66844000
H	2.78214300	4.13997000	9.48606500
H	2.41613700	4.92120400	7.11519400
H	3.65329400	2.10755600	6.96313200
H	3.89143000	2.68275400	4.49766500
H	1.38375900	3.50602600	5.42863700
H	4.27790300	2.42210500	2.20343300
H	0.50854900	1.73277900	3.57007400
H	2.87001300	2.17269500	1.18589200
H	0.80103200	2.12212700	1.96077900
H	4.75444500	-2.34133100	-2.91307300
H	5.13621100	0.65672900	-3.40304100
H	6.00411800	-1.24599400	-2.29750500
H	4.16444000	-0.29042900	-4.54664400
H	3.17934700	-4.88104000	-4.20791700
H	1.64194200	3.31074900	-4.03835300
H	3.78539000	2.77600300	-1.16013800
H	6.01272600	2.54341400	1.17134400
H	6.14826800	1.20008400	1.81422800
H	4.89597500	-0.12611200	0.65810800
H	5.83767300	0.69781100	-0.21762900
H	-2.07756400	10.18955200	-1.54142300

H	-3.48151600	9.08755900	-1.58962600
H	-5.42979700	0.53215600	-1.17339800
H	-4.43221100	-0.32611100	-1.96477300
H	-3.43096400	-1.06126100	1.42750800
H	-2.56322400	2.59088000	-2.10031900
H	-1.97383200	4.49124200	-3.67079700
H	-0.64982800	3.77279600	-3.19988900
H	-4.13790600	0.33615800	1.22501100
H	-3.81039800	1.59273600	-1.88968900
H	2.81507700	1.91851000	-4.58025200
H	3.52554100	3.27339100	-4.81752600
H	4.06601100	3.98271000	-3.02248600
H	5.35687500	3.42934900	-3.64883200
H	-3.29671000	-3.51324200	2.05652700
H	-2.45591700	-2.76790700	0.99525400
H	-8.83671900	-3.57414000	-0.50508400
H	6.83752200	-3.33959000	-0.08363200
O	-0.13762300	1.59018600	-1.93149700
H	-0.44408900	1.73149600	-2.84223500

$S_3{}^AY_z$ (W1=H_2O, W2=H_2O, Ox=OH^-)

Mn	0.00025300	-2.54872000	-1.06218500
Mn	0.81859300	-1.82594400	1.49815000
Mn	2.07389700	0.09732300	-0.11309700
Mn	1.67100000	2.42766900	-1.52944700
Ca	-1.50315600	0.33248500	0.19327400
O	-0.69373700	-2.00186500	0.55276500
O	0.92062000	-0.02427800	1.27740600
O	1.63247200	-1.84960400	-0.25697100
O	2.57962500	1.85668300	-0.05948800
O	0.82696200	0.80188400	-1.20762800
O	2.68689200	3.95043100	-1.86112300
O	0.48851800	2.66532500	-3.13514200
O	-2.52648500	1.70703300	-1.46399000
O	-3.63784200	0.11305500	1.23280200
H	7.44720600	5.41774000	-0.44365700
C	7.68501000	4.38710200	-0.15662900
H	8.74399000	4.34867900	0.12449300
C	7.41472600	3.40913700	-1.30623900
C	6.01819600	3.47132400	-1.88084600
O	5.11397300	3.95430200	-1.01887000
O	5.73460700	3.10574100	-3.01726000
C	-7.68744800	7.09714000	1.61252200
C	-7.31255500	6.62087100	0.19964900
C	-6.93892300	5.15533300	0.16071400

C	-7.87158900	4.17931600	0.54028300
C	-5.66178200	4.72835900	-0.22842700
C	-7.54928500	2.82410300	0.54458700
C	-5.31925000	3.37380500	-0.23723800
C	-6.26717700	2.42479200	0.15282500
O	-5.91998500	1.08877500	0.11893600
H	1.94881000	8.21158100	1.48753600
C	2.35345300	7.88876500	0.52148600
C	1.41220700	7.00572300	-0.27156000
O	1.75723200	6.49446500	-1.33881600
H	3.29440000	7.35073500	0.68049200
N	0.13710400	6.85010600	0.22094600
C	-0.87583500	6.10083700	-0.51639300
C	-1.97577600	6.96844800	-1.12703600
O	-2.87302600	6.42251300	-1.79371500
C	-1.38729500	4.86104800	0.25084600
C	-0.50483800	3.59489400	0.17260400
O	-0.76094100	2.61890300	0.89003200
O	0.45162100	3.65685000	-0.70370500
N	-1.82718200	8.29361700	-0.96795700
C	-2.60377600	9.31433800	-1.65444000
H	-1.97608000	9.85687200	-2.37173400
H	-5.97829500	-3.90496800	-3.88407400
C	-6.05726600	-4.22752900	-2.83559500
C	-7.50323700	-4.27582200	-2.39043600
O	-7.83288800	-4.82403500	-1.33090300
C	-5.19228300	-3.32914900	-1.95099200
C	-3.75419700	-3.43162400	-2.45463000
C	-2.73636800	-2.71101500	-1.60809600
O	-2.97893400	-1.64149900	-1.03651700
O	-1.58995200	-3.34802900	-1.57803900
N	-8.39201100	-3.66201800	-3.21696700
C	-9.80488200	-3.55571100	-2.90171900
H	10.39478800	-3.75404700	-3.80426800
C	10.20708700	-2.18418900	-2.31478700
C	-9.30205300	-1.71109900	-1.22133200
C	-8.74473000	-0.47941500	-0.97023400
N	-8.81908800	-2.53461900	-0.21484500
C	-7.98134000	-1.80447200	0.56325400
N	-7.93030000	-0.54791800	0.14527600
H	3.49151100	-4.49178000	-6.92467100
C	3.54762000	-3.41573700	-6.72240100
C	4.53988200	-3.21423000	-5.58765900
O	4.56016700	-3.97041600	-4.60337200

C	2.14985400	-2.87205900	-6.29777400
C	1.71860200	-3.21367900	-4.90140300
C	1.27009200	-2.41105100	-3.88094800
N	1.66849400	-4.50383000	-4.38558300
C	1.22872500	-4.45402600	-3.10674500
N	0.97082400	-3.19328400	-2.77703500
N	5.32425700	-2.11711600	-5.67987600
C	6.22050300	-1.72038900	-4.60198700
H	6.79222900	-2.59871100	-4.28237900
C	5.46294300	-1.14167000	-3.37628500
C	4.58659200	0.08259000	-3.67642200
C	3.58532100	0.45738100	-2.58639600
O	3.41757700	-0.29748100	-1.58366100
O	2.93569700	1.54097700	-2.78029700
H	7.77667800	-5.70864400	0.83008900
C	7.16436200	-5.72343900	1.74069900
H	7.59657500	-6.46245200	2.42364100
C	5.69608700	-6.08979500	1.43574400
C	4.97274800	-5.14438200	0.52317700
C	3.96545600	-4.24432400	0.76485600
N	5.21298300	-5.05023800	-0.84761600
C	4.39163700	-4.14224400	-1.41270000
N	3.63281400	-3.64271900	-0.43712200
H	-0.48199800	-7.57759200	2.73658700
C	-0.15931600	-6.52789600	2.70431800
C	-1.22916700	-5.68380500	3.40526700
O	-2.15087400	-5.15337700	2.78293000
C	0.01780900	-6.11872100	1.23558500
C	0.45339500	-4.68728300	0.91581600
O	0.78902500	-3.87950400	1.83300800
O	0.45076800	-4.39226800	-0.32538100
N	-1.11015200	-5.58575300	4.76210200
C	-2.20751400	-5.10992500	5.60431700
C	-2.53958800	-3.60731300	5.49956200
O	-3.63471600	-3.20321600	5.87082300
N	-1.55530500	-2.78756900	5.03299200
C	-1.85565000	-1.39778400	4.69687700
C	-1.08385000	-0.99394500	3.43756600
O	-1.38505600	0.02607200	2.81017600
O	-0.06392000	-1.76847300	3.15466400
O	-1.93240100	4.05795700	-2.89814000
O	3.65974900	4.51956500	-4.37536500
O	1.98741900	2.66986900	-5.19237400
O	4.86691000	2.45665300	1.31354500

O	-4.62618100	0.26448300	-2.17043200
O	-3.05233600	-2.55545300	1.97471200
H	5.86317900	-2.17362600	5.40931900
C	5.60741200	-1.10577000	5.39194400
H	5.34980500	-0.80823800	6.41614300
C	4.44643600	-0.83486300	4.43312100
C	4.79186900	-1.21881900	2.98071100
C	3.57462500	-1.14429500	2.08997900
O	2.58734300	-1.88886000	2.39852000
O	3.60158100	-0.35612400	1.09424300
H	5.04896200	3.88617100	8.66363400
C	4.01037500	4.20758800	8.81797900
C	3.13814000	3.87076400	7.60335600
C	3.10503300	2.36518200	7.29948100
C	2.12155300	1.97013100	6.19623400
N	2.53384400	2.51304400	4.89874900
C	1.87847000	2.28959400	3.74348400
N	0.72645000	1.62161000	3.74820400
N	2.37917800	2.76918400	2.59437200
O	6.11027900	0.36652500	-0.24028700
H	-8.54751300	6.54062500	2.00451900
H	-6.85227300	6.94755500	2.30913200
H	-7.95044700	8.16270300	1.61329700
H	-6.47496400	7.22009200	-0.18398400
H	-8.15984800	6.80789400	-0.47744600
H	-8.87348800	4.48254000	0.84075600
H	-8.28054400	2.07913000	0.84935900
H	-4.33503600	3.03595000	-0.55624800
H	-4.92609000	5.46497900	-0.55161700
H	-6.74875900	0.48577300	0.26555800
H	-8.87839800	0.44847700	-1.51209800
H	-7.43378200	-2.21882500	1.39982900
H	-0.74475700	-3.19530000	4.57897400
H	-0.36595100	-6.10767100	5.20894700
H	5.28832400	-1.55642900	-6.52169000
H	-8.03971700	-3.18136300	-4.03523300
H	10.01971700	-4.35725200	-2.18796700
H	11.24870800	-2.26107600	-1.96724800
H	10.20208900	-1.42274000	-3.10549300
H	-3.67642800	-2.99944100	-3.46579600
H	-5.54217100	-2.29014400	-1.99405800
H	-5.67828900	-5.25697600	-2.78504300
H	-3.44762200	-4.48061600	-2.54135800
H	-5.26200000	-3.66020900	-0.90734600

H	7.23258000	-4.73468700	2.20955000
H	3.44377000	-3.98403800	1.67503700
H	4.36341300	-3.90460800	-2.47388100
H	1.09714400	-1.34525300	-3.87429500
H	1.07916500	-5.30526300	-2.45872900
H	3.85667200	-2.92152100	-7.65050700
H	6.92476300	-0.98645200	-5.00770600
H	-0.32997700	5.72690600	-1.39230300
H	-0.06562400	7.16049000	1.16514700
H	2.58485900	8.77496000	-0.08176600
H	-1.00121500	8.58584600	-0.45923900
H	7.09923700	4.13864200	0.73539500
H	1.40517100	-3.22703700	-7.02392600
H	2.15038800	-1.77756100	-6.37103000
H	0.79115500	-6.46348000	3.25074200
H	-2.92038000	-1.32833600	4.45107700
C	-1.54213100	-0.42941500	5.84755600
H	-1.81210200	0.59535300	5.56084400
H	-2.13504400	-0.70936300	6.72412700
H	-0.47800600	-0.47691800	6.11979700
H	-1.95483400	-5.33178600	6.64629100
H	-3.13730600	-5.63597200	5.36025300
H	0.73284800	-6.79145800	0.74193400
H	-0.93493800	-6.24402000	0.70709100
H	-2.36655600	4.56199400	-0.14454400
H	-1.55294400	5.07974100	1.31367500
H	6.50773000	-0.55073800	5.09749700
H	3.55970300	-1.40337000	4.74332000
H	5.15066500	-2.25901400	2.95933300
H	4.17125600	0.23009300	4.46165900
H	5.57612600	-0.57957500	2.56284800
H	8.10667300	3.56909700	-2.14128000
H	7.55898200	2.36755400	-0.98052700
H	5.12720600	-6.12927300	2.37299800
H	5.65001200	-7.10262900	1.00924200
H	2.87837600	-2.90651700	-0.51702000
H	4.01804900	5.28649600	9.01090100
H	3.50620100	4.43139600	6.72858100
H	2.80694700	1.82239000	8.20668600
H	2.06314900	0.87602900	6.10845000
H	3.63567400	3.70712400	9.72021400
H	2.10944300	4.22096700	7.77733500
H	4.11110600	2.00186300	7.03920400
H	3.27876200	3.19883100	4.88244400

H	1.11859900	2.34481800	6.45720200
H	3.39836500	2.84073000	2.46469200
H	0.23615600	1.41291200	4.60655000
H	1.91886100	2.49834800	1.72306900
H	0.32101800	1.24885900	2.88823100
H	4.82598800	-1.93031100	-2.96825000
H	5.18717800	0.98449400	-3.85423000
H	6.19223000	-0.88042200	-2.59875300
H	3.98937800	-0.08868900	-4.58667200
H	2.04082800	-5.32781300	-4.84115400
H	1.01367500	2.78549000	-4.02545000
H	4.16568200	3.93301100	-1.41046200
H	5.12094100	3.21028300	0.73630100
H	4.14366200	2.05333200	0.77416900
H	5.25133200	-0.08616300	-0.29345100
H	5.93311500	1.05705900	0.43340600
H	-3.02576800	10.02327800	-0.93334200
H	-3.41722500	8.81898300	-2.19038000
H	-5.22194400	0.56709200	-1.44250900
H	-4.18166000	-0.52695200	-1.78933300
H	-3.75086300	-0.77825600	1.62774000
H	-2.31462000	2.50631400	-1.99488900
H	-2.32216100	4.88505400	-2.49685000
H	-2.35725700	3.97649200	-3.77028100
H	-4.51151300	0.43560000	0.90954700
H	-3.33471000	1.26914500	-1.87549100
H	2.49791800	1.96617400	-4.74894200
H	2.61414700	3.45800500	-5.06680500
H	3.21094700	4.51262000	-3.49807300
H	4.48449100	4.03356100	-4.15700200
H	-3.00970700	-3.41182000	2.44455700
H	-2.28599100	-2.58062400	1.36592300
H	-8.81148100	-3.55717800	-0.26609400
H	5.90548800	-5.58386100	-1.36183700
O	-0.49386900	-1.11517200	-1.97264300
H	0.17857100	-0.36879300	-1.94449000
H	2.22404400	4.76954100	-1.56034700
H	-0.21659900	3.35065700	-3.02846800

$S_3^{TS}Y_z$ (W1=H_2O, W2=H_2O, Ox=OH^-)

Mn	0.24897500	-2.58599700	-1.07265500
Mn	0.76284200	-1.93423000	1.55091200
Mn	1.89568300	-0.08068600	-0.19684900
Mn	1.59791500	2.58937800	-1.48643800
Ca	-1.51577600	0.24273400	0.10295100

O	-0.64876400	-2.16985400	0.51332300
O	0.76340600	-0.12428600	1.17007300
O	1.80059600	-2.03973900	-0.02212400
O	2.37837400	1.74640300	-0.12087000
O	0.27802300	-1.00022300	-1.64328300
O	2.72979200	4.03187800	-1.73847200
O	0.52448600	2.97656200	-3.16996100
O	-2.58009300	1.64732200	-1.52758900
O	-3.66679600	-0.13940300	1.05284700
H	7.36477200	5.63146200	-0.47787000
C	7.59392400	4.65234800	-0.04141300
H	8.63276800	4.67291000	0.30868400
C	7.40523500	3.52904100	-1.06890400
C	6.02569100	3.51332600	-1.68034500
O	5.08637700	3.93803100	-0.82504100
O	5.77994100	3.16471100	-2.83007300
C	-7.88837000	6.87549300	1.47674000
C	-7.46641900	6.40913200	0.07444100
C	-7.06563300	4.95135700	0.05486700
C	-7.99460200	3.96290800	0.41016800
C	-5.76689700	4.54477700	-0.27919400
C	-7.64809200	2.61476200	0.44417200
C	-5.39947900	3.19695400	-0.25785800
C	-6.34447700	2.23507100	0.10676600
O	-5.97616400	0.90548000	0.09835500
H	1.70535500	8.30424100	1.48940800
C	2.14401000	7.98600800	0.53679700
C	1.25252700	7.06370000	-0.26986400
O	1.63825500	6.55072500	-1.32247600
H	3.09871500	7.48256800	0.72483200
N	-0.02768900	6.87829800	0.19638800
C	-1.00988500	6.09199500	-0.54386300
C	-2.12881900	6.92858200	-1.16546300
O	-3.02061600	6.35831300	-1.82019500
C	-1.49979100	4.85688800	0.23808500
C	-0.54457400	3.64640600	0.22313400
O	-0.79199400	2.64715700	0.90589300
O	0.48507100	3.80959400	-0.56401400
N	-2.01006200	8.25835000	-1.02724200
C	-2.82071200	9.24495600	-1.72347200
H	-2.21595800	9.78938700	-2.45884000
H	-5.77434100	-4.14205000	-3.96127500
C	-5.83628100	-4.38660300	-2.89070400
C	-7.27882900	-4.43372800	-2.43724600

O	-7.60815900	-4.97555400	-1.37486800
C	-4.96055400	-3.39849800	-2.09029900
C	-3.53065400	-3.43915600	-2.65339100
C	-2.47826500	-2.64146200	-1.89666400
O	-2.71305500	-1.52782000	-1.39581000
O	-1.32107200	-3.23851500	-1.87168900
N	-8.16986400	-3.83953700	-3.27719300
C	-9.59457300	-3.78797200	-3.00377500
H	-10.14710200	-4.00790400	-3.92491100
C	-10.07263100	-2.43774400	-2.42778600
C	-9.23425300	-1.94311200	-1.29233800
C	-8.74038700	-0.69352100	-1.00264800
N	-8.75662300	-2.76173700	-0.27932000
C	-7.98033400	-2.00973100	0.54010800
N	-7.96711600	-0.74546000	0.14287600
H	3.77092100	-4.38299100	-6.82697300
C	3.81026700	-3.30383900	-6.63653900
C	4.78612600	-3.07841400	-5.49114500
O	4.80334600	-3.82523100	-4.50055300
C	2.40036700	-2.77565000	-6.24753900
C	1.95325200	-3.15427300	-4.86989600
C	1.57265800	-2.37902300	-3.80381900
N	1.86068100	-4.46407700	-4.41077200
C	1.45555800	-4.45322200	-3.12018500
N	1.26906300	-3.19710600	-2.73021800
N	5.56091700	-1.97407600	-5.58743200
C	6.39414000	-1.51621600	-4.48192800
H	6.99705600	-2.35687500	-4.12200700
C	5.54307300	-0.96676000	-3.29395400
C	4.57815600	0.18643300	-3.63422900
C	3.48679700	0.48363300	-2.59697100
O	3.28245000	-0.33537300	-1.64759300
O	2.79885900	1.54503400	-2.78419200
H	8.00814100	-5.33442200	1.01431800
C	7.36314600	-5.46201600	1.89287100
H	7.85850700	-6.16149400	2.57502700
C	5.97271000	-5.99867500	1.50244700
C	5.21124100	-5.09668600	0.58253600
C	4.18355400	-4.21841200	0.81398200
N	5.46943500	-4.99289900	-0.78375400
C	4.63598000	-4.10183900	-1.35883800
N	3.85606600	-3.62528200	-0.39048800
H	-0.20522100	-7.55604500	2.86502900
C	0.05426700	-6.49448900	2.74419100

C	-1.05580400	-5.67302800	3.41634800
O	-2.00640400	-5.21675400	2.78087500
C	0.19068100	-6.18098700	1.26256500
C	0.58025100	-4.75533900	0.90498100
O	0.87258400	-3.92970400	1.83883000
O	0.57830000	-4.49026900	-0.33137400
N	-0.94165700	-5.51831000	4.77196300
C	-2.08347800	-5.13038300	5.60540700
C	-2.61121500	-3.69777800	5.39050300
O	-3.79192100	-3.43923100	5.58481100
N	-1.67972700	-2.76032000	5.04996100
C	-2.09554700	-1.43765300	4.58800000
C	-1.24214200	-1.03725000	3.37890200
O	-1.49985800	-0.00977300	2.73788900
O	-0.22910000	-1.82799700	3.14266800
O	-2.02522100	4.04891100	-2.90922800
O	3.84443000	4.74545700	-4.18763500
O	2.13161000	2.98240000	-5.12986700
O	4.57904300	2.38728900	1.40123800
O	-4.67801800	0.20773700	-2.24625400
O	-3.08019600	-2.77650700	1.71154900
H	6.24684200	-1.73836300	5.26071200
C	5.60303600	-0.88121600	5.49802000
H	5.31744700	-0.95562000	6.55385900
C	4.34301000	-0.86457200	4.60252800
C	4.63405900	-0.74260400	3.09039400
C	3.39675000	-0.90678700	2.20601400
O	2.48007500	-1.68471700	2.59454400
O	3.39666700	-0.25378600	1.09689600
H	4.81572700	4.02526000	8.78645000
C	3.78461800	4.39231400	8.87474900
C	2.96224000	4.05341100	7.62147800
C	2.88341200	2.54107200	7.35425700
C	1.86950900	2.12750500	6.28298000
N	2.28164100	2.53734400	4.93507300
C	1.57107900	2.26945200	3.81947200
N	0.37924200	1.67837900	3.92023100
N	2.04686700	2.61841200	2.61792000
O	5.91949300	0.41053300	-0.25054900
H	-8.74819900	6.30215700	1.84402500
H	-7.06975500	6.73931500	2.19542600
H	-8.17097500	7.93612500	1.47334100
H	-6.62955000	7.02333600	-0.28639100
H	-8.29824000	6.57902700	-0.62585400

H	-9.01241300	4.25128500	0.66887200
H	-8.37625400	1.85997800	0.73112700
H	-4.39781400	2.87488600	-0.53543100
H	-5.03330000	5.29246200	-0.58130900
H	-6.79802900	0.29505400	0.25554900
H	-8.89007900	0.23597600	-1.53759300
H	-7.44588800	-2.41495600	1.38955900
H	-0.78970300	-3.08939400	4.68861800
H	-0.17911400	-6.00268500	5.23082300
H	5.51233600	-1.41368100	-6.42884000
H	-7.81809700	-3.37449900	-4.10449600
H	-9.80066900	-4.59984200	-2.29866300
H	-11.12490200	-2.55643400	-2.12700300
H	-10.06118800	-1.67022700	-3.21251100
H	-3.53209900	-3.04708000	-3.68355600
H	-5.36861000	-2.38241800	-2.16545800
H	-5.43410300	-5.40055800	-2.76760300
H	-3.17659900	-4.47461500	-2.71650400
H	-4.96343900	-3.67757300	-1.02892900
H	7.27952800	-4.49002600	2.39334500
H	3.64236200	-3.97090400	1.71690100
H	4.61609400	-3.85605600	-2.41905600
H	1.44888700	-1.30996900	-3.72267100
H	1.26932500	-5.32512900	-2.50995600
H	4.13211600	-2.81927300	-7.56588800
H	7.07219600	-0.74928100	-4.86978900
H	-0.44579100	5.72286600	-1.40926500
H	-0.25864700	7.20080600	1.12998600
H	2.36036300	8.87376900	-0.06978500
H	-1.18903300	8.57981200	-0.52831000
H	6.94744900	4.51221900	0.83152900
H	1.67832000	-3.12318800	-6.99960400
H	2.39426000	-1.67987600	-6.29858100
H	1.00509800	-6.33512800	3.27034100
H	-3.12955600	-1.51051500	4.23011600
C	-2.02107800	-0.37085600	5.68823400
H	-2.34339400	0.60040600	5.29324600
H	-2.68732000	-0.65274600	6.50971800
H	-0.99918100	-0.29540600	6.08763000
H	-1.78010800	-5.23347300	6.65288100
H	-2.94004700	-5.79057800	5.42790600
H	0.92018600	-6.85421800	0.79219100
H	-0.76565500	-6.35564400	0.75487100
H	-2.44604900	4.49901500	-0.18658400

H	-1.71842600	5.09872800	1.28651600
H	6.20072600	0.03071100	5.36907100
H	3.75634800	-1.77583100	4.77339000
H	5.33735800	-1.53163500	2.77651500
H	3.69630700	-0.02393700	4.89677200
H	5.10564800	0.21289700	2.83639300
H	8.12425300	3.60430500	-1.89198900
H	7.55650800	2.54094200	-0.60819700
H	5.36482500	-6.13972900	2.40463400
H	6.07031100	-6.99152100	1.04027900
H	3.07115800	-2.92436100	-0.45022300
H	3.82615900	5.47559300	9.03575800
H	3.39112200	4.57392900	6.75014200
H	2.58169200	2.03305200	8.28057200
H	1.74705200	1.03480300	6.28573300
H	3.34076800	3.93466100	9.76821700
H	1.94060100	4.44578900	7.73834400
H	3.87534300	2.13925100	7.09617900
H	3.08868200	3.14155500	4.84055400
H	0.89393200	2.57915400	6.52477100
H	3.05968600	2.70194700	2.44892400
H	-0.06487000	1.53510800	4.81612200
H	1.52792700	2.32673600	1.78833900
H	-0.11198900	1.29864200	3.10818300
H	4.95530900	-1.79748500	-2.89466800
H	5.11370100	1.13229800	-3.78481200
H	6.22177500	-0.63842700	-2.49702900
H	4.03895400	-0.03037900	-4.57094700
H	2.16210700	-5.28591200	-4.91955600
H	1.08501400	3.14062600	-4.02828800
H	4.15024000	3.94326500	-1.25151100
H	4.89721800	3.14024900	0.85380300
H	3.89536700	1.98887100	0.80698100
H	5.07063500	-0.03996000	-0.39877500
H	5.69542900	1.03934600	0.46627900
H	-3.25182500	9.95698500	-1.01089100
H	-3.62740800	8.71844400	-2.23930600
H	-5.26243600	0.42176600	-1.47979200
H	-4.13979500	-0.55631000	-1.93514600
H	-3.76633500	-1.04873500	1.41188800
H	-2.38867500	2.48454300	-2.00456300
H	-2.44777700	4.86533600	-2.51495600
H	-2.44616400	3.94223500	-3.78072100
H	-4.55026600	0.22008700	0.80679100

H	-3.41220700	1.24546300	-1.92527200
H	2.56991100	2.25580700	-4.64384800
H	2.78374700	3.73854100	-4.97441700
H	3.35626900	4.68863000	-3.33557000
H	4.64203600	4.21496400	-3.97509200
H	-3.01147600	-3.57548700	2.27042200
H	-2.23672900	-2.76669700	1.21070600
H	-8.69396300	-3.78166000	-0.35007600
H	6.18038700	-5.51143100	-1.28803300
O	0.35787200	1.24856200	-1.25414700
H	0.11261300	0.90145600	-2.13499200
H	2.28045600	4.87227400	-1.47360000
H	-0.26190900	3.57005200	-3.08473500

$S_3^{B,W}Y_z$ (W1=H_2O, W2=H_2O, Ox=OH^-)

Mn	0.44262300	-2.49858700	-0.99529900
Mn	0.87420100	-1.90634300	1.63076900
Mn	1.94625600	-0.18497700	-0.20721800
Mn	1.28694600	2.55772900	-1.67311500
Ca	-1.46152000	0.28212000	-0.00150300
O	-0.52118400	-2.16681800	0.50088200
O	0.81915000	-0.16359800	1.20601100
O	1.96663600	-2.10469000	0.07134900
O	1.92898700	1.63500700	-0.28908400
O	0.62144300	-0.75643500	-1.37151600
O	2.60061600	3.89804400	-1.70577000
O	0.52335800	3.55262900	-3.30280200
O	-2.90555700	1.64610200	-1.42843000
O	-3.58472700	-0.30386800	0.95277500
H	7.22271100	5.96020900	-0.43820600
C	7.42897200	5.00446400	0.05778900
H	8.44235200	5.04986900	0.47356400
C	7.31629000	3.83121900	-0.92586900
C	5.97251000	3.71364200	-1.60616800
O	4.96245000	4.15978900	-0.84690800
O	5.81145400	3.25533600	-2.73261700
C	-8.15302400	6.57359100	1.35694600
C	-7.84295800	6.02428900	-0.04440500
C	-7.38022500	4.58866000	0.02251700
C	-8.28952700	3.57116500	0.34393300
C	-6.03533200	4.23919000	-0.15460300
C	-7.88080800	2.24757200	0.49306500
C	-5.60420200	2.91932400	-0.01112500
C	-6.53136500	1.92542400	0.31223000
O	-6.09027000	0.62315100	0.41940800

H	1.37192900	8.39698000	1.50314800
C	1.83560200	8.10568700	0.55356400
C	1.02310700	7.09767300	-0.23999800
O	1.47530600	6.53779400	-1.23623600
H	2.83109500	7.69142000	0.74337300
N	-0.27954100	6.90746200	0.17875500
C	-1.22014300	6.07844900	-0.56665700
C	-2.35909700	6.86281000	-1.21610300
O	-3.21641500	6.25052700	-1.88086700
C	-1.68439300	4.83292400	0.21549100
C	-0.71859800	3.63769500	0.14696600
O	-0.91862700	2.62447100	0.83684300
O	0.25495300	3.81102000	-0.69039700
N	-2.29843300	8.19632900	-1.08103300
C	-3.14594200	9.15028100	-1.77780500
H	-2.56217400	9.72070500	-2.51046500
H	-5.49879500	-4.36817800	-4.03145300
C	-5.56653000	-4.59828800	-2.95809900
C	-7.01191100	-4.71186900	-2.52678000
O	-7.33298800	-5.28768200	-1.48001800
C	-4.74493700	-3.55052300	-2.15966300
C	-3.28135700	-3.55986100	-2.64368300
C	-2.29355500	-2.61555700	-1.95587200
O	-2.61680800	-1.52086800	-1.46852600
O	-1.06511800	-3.05804800	-1.98761100
N	-7.91445300	-4.14703800	-3.37400200
C	-9.34481600	-4.15919700	-3.12440800
H	-9.87233800	-4.37290000	-4.06133500
C	-9.88155900	-2.84191700	-2.52185800
C	-9.10378000	-2.35129300	-1.34036200
C	-8.69652400	-1.08870500	-0.97714900
N	-8.62723800	-3.18357400	-0.33814300
C	-7.93581300	-2.42415600	0.54797200
N	-7.97681800	-1.14533100	0.20294200
H	4.03936500	-4.19548800	-6.75021300
C	4.07685000	-3.11445300	-6.57237800
C	5.05305900	-2.86693300	-5.43059600
O	5.10144100	-3.62701600	-4.45025400
C	2.66766100	-2.57931700	-6.18004900
C	2.22303600	-2.95439500	-4.79854000
C	1.78140900	-2.19286200	-3.74327200
N	2.20302700	-4.26280800	-4.32831300
C	1.78466600	-4.26661000	-3.04307000
N	1.52108800	-3.02102000	-2.66416400

N	5.77783100	-1.72897000	-5.51198500
C	6.55269000	-1.21598100	-4.38597400
H	7.19668600	-2.01626800	-4.00634300
C	5.63596700	-0.71213700	-3.22828700
C	4.57760200	0.34120200	-3.61279500
C	3.43387900	0.53256100	-2.60671300
O	3.39496200	-0.23008000	-1.58672000
O	2.56791200	1.42920500	-2.88314000
H	8.26775300	-4.89503000	1.16656200
C	7.59871900	-5.10768900	2.00986800
H	8.13433400	-5.76014500	2.70812200
C	6.29689100	-5.78482400	1.54266700
C	5.50220100	-4.94236100	0.59691600
C	4.41266000	-4.13340700	0.79330000
N	5.81112600	-4.80657900	-0.75591600
C	4.95042500	-3.96242200	-1.35949200
N	4.10196700	-3.54911500	-0.41947600
H	0.14678800	-7.51968600	2.92009100
C	0.32883700	-6.44684500	2.76215100
C	-0.82785900	-5.68145400	3.42037700
O	-1.79643400	-5.28108400	2.77387200
C	0.42304500	-6.17080700	1.26596100
C	0.71719400	-4.72361800	0.90656000
O	0.99062700	-3.91651900	1.85410400
O	0.66149300	-4.43356400	-0.33171300
N	-0.73431400	-5.50961900	4.77491500
C	-1.90421100	-5.16999300	5.59065600
C	-2.49461900	-3.76551300	5.35244200
O	-3.69281200	-3.56401900	5.50031000
N	-1.59663400	-2.78376600	5.04590500
C	-2.05808700	-1.48261000	4.56544900
C	-1.20811300	-1.04872000	3.37172300
O	-1.48918000	-0.03487600	2.72401300
O	-0.16815300	-1.81662900	3.15894500
O	-2.27667800	3.92567400	-2.99637700
O	3.62888500	4.25631000	-4.27616200
O	1.82233200	2.61615200	-5.29277700
O	4.06799000	2.64267500	1.26056000
O	-4.87922900	-0.00989000	-2.00989900
O	-2.91782900	-2.88411300	1.69121000
H	6.03590300	-1.60567700	5.54632700
C	5.59671900	-0.59993900	5.58072300
H	5.30454600	-0.39566700	6.61780200
C	4.37561700	-0.49757800	4.64332400

C	4.70722000	-0.74614500	3.15566900
C	3.47806500	-0.85539200	2.25925300
O	2.55231200	-1.64757500	2.65388300
O	3.46626200	-0.19375500	1.17442300
H	4.58508000	4.43791400	8.76092000
C	3.51038700	4.59711300	8.92092200
C	2.69599500	4.21867300	7.67187500
C	2.87599500	2.74567000	7.26595000
C	1.85519600	2.21859700	6.25117400
N	2.06653800	2.73949100	4.89264500
C	1.27276300	2.42384500	3.84551000
N	0.08916500	1.84308800	4.07042500
N	1.62984700	2.73082300	2.59568300
O	5.88752400	0.80642500	-0.02040900
H	-8.94009000	5.98376300	1.84306100
H	-7.26280000	6.52687800	1.99780300
H	-8.49124800	7.61708900	1.31202700
H	-7.07070100	6.64153600	-0.52444900
H	-8.74257700	6.10350700	-0.67196700
H	-9.34055200	3.81983800	0.48682700
H	-8.59544600	1.46969000	0.75147200
H	-4.56501600	2.64213200	-0.17040400
H	-5.31412100	5.01119200	-0.42639200
H	-6.88514400	-0.03488800	0.49134900
H	-8.87546400	-0.14703300	-1.48084100
H	-7.41969400	-2.83441400	1.40628800
H	-0.67927900	-3.07195200	4.72093300
H	0.04576000	-5.95236400	5.24640100
H	5.69165900	-1.15592400	-6.34210100
H	-7.56930600	-3.64723100	-4.18368400
H	-9.53210700	-4.99726700	-2.44519800
H	-10.93930400	-3.00192900	-2.26164100
H	-9.86885400	-2.05148400	-3.28361100
H	-3.24724800	-3.29710500	-3.71399100
H	-5.18318300	-2.55327400	-2.29167000
H	-5.11853800	-5.58963600	-2.81136200
H	-2.86330200	-4.57112200	-2.56899200
H	-4.78951800	-3.78913700	-1.08919500
H	7.38414000	-4.15940100	2.51684400
H	3.82318900	-3.92106600	1.67497700
H	4.95788300	-3.70412700	-2.41796800
H	1.59285700	-1.13116900	-3.67539300
H	1.64136300	-5.14435900	-2.42938500
H	4.39234300	-2.63857700	-7.50824100

H	7.19225400	-0.41145800	-4.76244700
H	-0.62524700	5.71777300	-1.41643400
H	-0.54917600	7.25378700	1.09321400
H	1.96816400	9.00316400	-0.06352600
H	-1.49333100	8.54931400	-0.57669100
H	6.72412500	4.89747000	0.88956200
H	1.94226700	-2.93072000	-6.92715400
H	2.66244300	-1.48405600	-6.23851300
H	1.27211200	-6.20177900	3.26719300
H	-3.08074800	-1.60748700	4.18864700
C	-2.06385600	-0.41294500	5.66572900
H	-2.42860600	0.54053200	5.26474800
H	-2.73363800	-0.73125500	6.47074100
H	-1.05720100	-0.28464700	6.08880700
H	-1.60966600	-5.24897500	6.64273600
H	-2.72606500	-5.87135000	5.40769600
H	1.19496000	-6.80205400	0.80460400
H	-0.52377600	-6.42180600	0.77365100
H	-2.63572200	4.46884400	-0.19431600
H	-1.87886300	5.05919700	1.27167800
H	6.37901700	0.11729800	5.30017400
H	3.60890900	-1.21957700	4.95346200
H	5.23877600	-1.70737300	3.06205500
H	3.92081400	0.50081400	4.73518700
H	5.36375600	0.02562700	2.74145400
H	8.07243200	3.89129100	-1.71665500
H	7.47516500	2.87058100	-0.41214400
H	5.66143000	-6.00506500	2.40916300
H	6.52216400	-6.75239500	1.07209000
H	3.29012900	-2.89490400	-0.49467800
H	3.36234200	5.65177300	9.17963100
H	2.96832500	4.87850500	6.83418400
H	2.76730100	2.11711900	8.16098700
H	1.89355800	1.11941100	6.21644200
H	3.20595300	3.99112800	9.78408300
H	1.62843800	4.40083300	7.86855400
H	3.89734700	2.56314300	6.89568000
H	2.93211400	3.22572900	4.69188500
H	0.85142700	2.51193400	6.59331900
H	2.61859200	2.85116400	2.32421200
H	-0.23819200	1.66941400	5.00934900
H	1.01984200	2.42200400	1.83623400
H	-0.44185300	1.40022800	3.31624000
H	5.11793100	-1.58028900	-2.81178800

H	5.02730500	1.33304600	-3.74655200
H	6.27125000	-0.30413000	-2.43182300
H	4.09499900	0.07906200	-4.56699400
H	2.57826200	-5.06435700	-4.82013900
H	0.88401800	3.22234000	-4.21207400
H	4.04583400	4.02270600	-1.29802600
H	4.44150600	3.38422500	0.73205200
H	3.43439800	2.21805500	0.63176500
H	5.16748200	0.20669700	-0.27981100
H	5.42104300	1.40653100	0.59971100
H	-3.60741800	9.84271200	-1.06487800
H	-3.92936200	8.59297100	-2.29728900
H	-5.42305900	0.13616900	-1.19987200
H	-4.23225400	-0.70341800	-1.74768600
H	-3.62081800	-1.19785500	1.35961300
H	-2.80327600	2.47302500	-1.94691700
H	-2.62961200	4.76431000	-2.57486000
H	-2.77288400	3.84973700	-3.83124000
H	-4.49738000	0.05967100	0.89887900
H	-3.72718000	1.16767700	-1.75560100
H	2.11710300	1.88506100	-4.71340500
H	2.57217200	3.27509900	-5.14185000
H	3.13302800	4.26476900	-3.42472300
H	4.48894000	3.88286900	-3.98997000
H	-2.82749600	-3.68386100	2.24636500
H	-2.07179800	-2.83802300	1.19791900
H	-8.50279400	-4.19434000	-0.44760300
H	6.57401000	-5.27521300	-1.23268500
O	-0.16865500	1.47659700	-1.81291200
H	-0.75855500	1.76929700	-2.52769200
H	2.14417300	4.75771800	-1.54462100
H	-0.44692900	3.74207700	-3.30361600

$S_3^{A,W}Y_z^{\bullet}$ (W1=OH$^-$, W2=H_2O, Ox=OH$^-$)

Mn	-0.12281500	-2.59942900	-1.19699300
Mn	0.59372100	-1.92942100	1.43007000
Mn	2.07280200	-0.12796400	-0.13912100
Mn	1.95224000	2.46475900	-1.46904200
Ca	-1.44020400	0.41403500	0.08380700
O	-0.86827500	-1.95501800	0.41170900
O	0.88796000	-0.14881500	1.25118900
O	1.50211200	-2.05822200	-0.22932600
O	2.69855500	1.55250700	-0.04248700
O	-0.30990100	-1.23627500	-2.16242100
O	3.01848000	3.72908600	-1.63225100

O	0.79510400	2.80355800	-3.16011300
O	-2.73445600	1.93811600	-1.28294300
O	-3.79334000	-0.10393400	0.83557200
H	7.83220000	4.92307800	-0.44249000
C	7.99863300	3.85862100	-0.23387500
H	9.06890800	3.72309700	-0.03689500
C	7.56796900	2.98844000	-1.42314800
C	6.14686200	3.28972100	-1.83383000
O	5.29139000	3.24068500	-0.80080100
O	5.80133400	3.56435400	-2.97622900
C	-7.18767600	7.59831000	1.29674200
C	-6.76203800	6.93211300	-0.03196600
C	-6.54126400	5.46215600	0.16368000
C	-7.65145600	4.63704100	0.50229100
C	-5.24742400	4.88479500	0.09676500
C	-7.47922700	3.31464300	0.81505000
C	-5.03901400	3.56276800	0.42376900
C	-6.15495600	2.73108000	0.82417700
O	-5.98753500	1.51535100	1.16817800
H	2.52308400	7.97164800	1.29747700
C	2.90001300	7.71435700	0.30120400
C	1.85765200	7.05789000	-0.59441700
O	2.05811300	6.80142700	-1.77555600
H	3.75063300	7.02976900	0.40527400
N	0.62378400	6.82484500	-0.01326100
C	-0.42813600	6.11592500	-0.73320000
C	-1.47294300	7.04415200	-1.35982000
O	-2.45526400	6.55721600	-1.95924300
C	-1.07344600	5.00555400	0.12331600
C	-0.33028800	3.65308800	0.13251800
O	-0.81420300	2.70047900	0.76651700
O	0.74721200	3.66438200	-0.56625900
N	-1.21402700	8.35556200	-1.26353800
C	-1.93135500	9.40683400	-1.96464000
H	-1.27313000	9.90057600	-2.68959600
H	-6.16786100	-3.60630200	-3.92734700
C	-6.25827500	-3.88727300	-2.86772400
C	-7.70263400	-3.83114400	-2.44087800
O	-8.09237400	-4.33615800	-1.37407700
C	-5.30298500	-3.03007700	-2.03623900
C	-3.92596500	-3.14848000	-2.69707600
C	-2.81307700	-2.49191100	-1.91401300
O	-2.93011400	-1.32450100	-1.50444500
O	-1.79058400	-3.27236200	-1.74692100

N	-8.53963100	-3.16275200	-3.28019600
C	-9.94168800	-2.92946800	-2.97882600
H	-10.53516900	-3.01389200	-3.89475300
C	-10.18697000	-1.53568900	-2.33891900
C	-9.23952600	-1.18278800	-1.22654500
C	-8.61612500	-0.00044900	-0.90984100
N	-8.81749600	-2.09452900	-0.26555500
C	-7.95296400	-1.50544300	0.56982200
N	-7.83667800	-0.22612400	0.21008900
H	3.34362100	-4.88963700	-6.85553400
C	3.42785400	-3.81222400	-6.66680000
C	4.40337400	-3.63956100	-5.51202100
O	4.35631400	-4.36327300	-4.50599400
C	2.03629400	-3.22704200	-6.30959900
C	1.56191700	-3.53845500	-4.92635100
C	1.22436100	-2.69188100	-3.90442000
N	1.37153500	-4.81781600	-4.41225600
C	0.95150400	-4.71551500	-3.12746300
N	0.84486600	-3.43367400	-2.80096900
N	5.27825700	-2.61496100	-5.63283300
C	6.18399400	-2.24435000	-4.55479300
H	6.69966500	-3.14562900	-4.20483300
C	5.44294100	-1.58707400	-3.35136700
C	4.58780400	-0.36073000	-3.70568100
C	3.62720500	0.15465000	-2.62170700
O	3.31871900	-0.61182100	-1.64789700
O	3.17201400	1.32005100	-2.80443700
H	7.42507600	-6.14894200	0.98665800
C	6.80017600	-6.14901900	1.88884200
H	7.19772100	-6.90608900	2.57319500
C	5.32374800	-6.46465200	1.56079900
C	4.63710700	-5.49619000	0.64220200
C	3.65547700	-4.56481200	0.87486000
N	4.88648900	-5.41311500	-0.72799300
C	4.09426400	-4.48006300	-1.29738600
N	3.34703400	-3.95563900	-0.32856800
H	-0.91673600	-7.48892500	2.90620600
C	-0.56973200	-6.45303100	2.78476700
C	-1.60408700	-5.53819600	3.45399300
O	-2.53752200	-5.03387000	2.82716800
C	-0.41932300	-6.14944000	1.29288200
C	0.07697300	-4.75837100	0.89658600
O	0.41438000	-3.92805100	1.80606300
O	0.11574900	-4.52210800	-0.34231500

N	-1.45758200	-5.35301300	4.80123700
C	-2.55034300	-4.83663800	5.62766400
C	-2.93552900	-3.36520100	5.38307000
O	-4.08045700	-2.98391100	5.60184500
N	-1.92696100	-2.53875100	4.99093000
C	-2.21018000	-1.17897500	4.54147000
C	-1.36728200	-0.87714300	3.28739700
O	-1.62052100	0.12928600	2.60957000
O	-0.40029900	-1.71918600	3.06650500
O	-1.76471600	4.05097200	-2.85692500
O	3.82131400	5.49649300	-3.74626100
O	2.63447900	3.37433000	-4.79457100
O	5.56730700	2.85406900	2.13803200
O	-4.98516700	0.44651500	-1.59427900
O	-3.30115800	-2.67315700	1.41085000
H	5.70202700	-2.43087600	5.51981200
C	5.51069400	-1.35455100	5.41433500
H	5.31092900	-0.95103700	6.41490700
C	4.33013900	-1.09312300	4.47179300
C	4.59744200	-1.63276900	3.05341700
C	3.39513600	-1.48117800	2.14319000
O	2.33412800	-2.09941200	2.46197400
O	3.53055100	-0.75209300	1.10478300
H	5.24880600	3.75453700	8.54229700
C	4.22907900	4.13080500	8.69871200
C	3.32720700	3.79647400	7.50677000
C	3.21429400	2.28506300	7.25993300
C	2.25666600	1.90631500	6.12833900
N	2.74975500	2.40038700	4.84506000
C	2.23458300	2.06517600	3.64143700
N	1.08244600	1.38637800	3.57601100
N	2.87713300	2.42300100	2.53501700
O	5.77296200	0.73702200	0.40803000
H	-8.12131000	7.16903200	1.67853100
H	-6.41553900	7.46680100	2.06412100
H	-7.34567300	8.67254300	1.14641700
H	-5.85057700	7.40337900	-0.41903700
H	-7.55409200	7.09102300	-0.77921700
H	-8.64514700	5.08040800	0.52963300
H	-8.31736800	2.68664400	1.11053500
H	-4.05751800	3.09963700	0.34203300
H	-4.41450100	5.50620900	-0.23112100
H	-7.18904900	0.46349200	0.66397600
H	-8.65153000	0.96703200	-1.38848900

H	-7.41907500	-1.99148400	1.37424500
H	-1.07789200	-2.95248500	4.61613700
H	-0.71699100	-5.86301100	5.26762000
H	5.27034200	-2.05974200	-6.47877000
H	-8.14961200	-2.75298200	-4.12005600
H	-10.25826300	-3.72855300	-2.30096400
H	-11.22603500	-1.50009300	-1.97945500
H	-10.09765900	-0.75566200	-3.10531200
H	-3.95007500	-2.67162100	-3.68956600
H	-5.63010300	-1.98300500	-2.00738200
H	-5.94808400	-4.93803700	-2.79301200
H	-3.66976800	-4.20242900	-2.85101200
H	-5.26199400	-3.39297600	-1.00129200
H	6.89419300	-5.16581800	2.36498100
H	3.13453500	-4.28652800	1.78034100
H	4.07655900	-4.24320500	-2.35825200
H	1.16943900	-1.61437900	-3.88241100
H	0.70069400	-5.54160800	-2.47800900
H	3.78925700	-3.34822400	-7.59230100
H	6.93290500	-1.56227100	-4.97102600
H	0.07664300	5.64199100	-1.58407800
H	0.54364700	6.90481500	0.99385900
H	3.26712400	8.61980900	-0.19383100
H	-0.36059000	8.59079900	-0.76805300
H	7.45742300	3.59264500	0.67801100
H	1.31095500	-3.57221200	-7.05983400
H	2.06833100	-2.13369200	-6.39303300
H	0.39433500	-6.37021500	3.30415500
H	-3.26501200	-1.13478000	4.24601000
C	-1.94658200	-0.12822900	5.62837600
H	-2.15932600	0.87625300	5.24280600
H	-2.59294300	-0.32244400	6.49065700
H	-0.90132400	-0.17774100	5.96533400
H	-2.25542000	-4.94503300	6.67708100
H	-3.46721800	-5.41472000	5.46616800
H	0.25072800	-6.88227100	0.82334500
H	-1.39180300	-6.25901000	0.79770600
H	-2.07850000	4.79112300	-0.25560600
H	-1.20795600	5.33056000	1.16512100
H	6.42953900	-0.88445900	5.04032800
H	3.41715600	-1.56314400	4.86124600
H	4.83234300	-2.70578100	3.11509800
H	4.13331600	-0.01302400	4.40944100
H	5.45539100	-1.12345100	2.60118600

H	8.20706700	3.15197100	-2.29641300
H	7.63004200	1.92691200	-1.14226400
H	4.74259700	-6.48988500	2.49106600
H	5.25177100	-7.47484100	1.13134500
H	2.62275300	-3.17492300	-0.41199000
H	4.29219700	5.21393800	8.85579000
H	3.71523400	4.30369900	6.60931700
H	2.85442700	1.79588800	8.17540900
H	2.15230700	0.81300800	6.06828800
H	3.84436700	3.67904100	9.62253700
H	2.31944500	4.20603800	7.67415100
H	4.20710200	1.85788100	7.04982200
H	3.56054000	3.00698600	4.84042100
H	1.26037600	2.32875800	6.33905900
H	3.89684500	2.63427900	2.53911600
H	0.47995000	1.28601200	4.37931400
H	2.52310700	2.14556200	1.60799800
H	0.80432600	0.93503100	2.69515400
H	4.79227200	-2.33719100	-2.89611100
H	5.20535500	0.49650300	-4.00379100
H	6.18998700	-1.31420900	-2.59362200
H	3.94700000	-0.59242200	-4.57097600
H	1.62482900	-5.68110400	-4.87586800
H	1.45202700	3.14093000	-3.89899600
H	4.30879400	3.44927700	-1.14839300
H	5.47998800	3.41246300	1.33976100
H	5.77435200	1.97682100	1.71685900
H	5.00643300	0.15638100	0.59909200
H	5.38966500	1.45004600	-0.14565400
H	-2.31169700	10.15382400	-1.25778500
H	-2.77084100	8.94927900	-2.49429000
H	-4.85786300	0.31781100	-0.62414300
H	-4.35626300	-0.25321800	-1.89636600
H	-3.76433600	-1.03527900	1.19475900
H	-2.44163300	2.65352700	-1.89523900
H	-2.08028800	4.95118300	-2.55925200
H	-2.01308600	3.98446600	-3.79531300
H	-4.26760900	0.45861100	1.47430800
H	-3.60811100	1.57719500	-1.58863300
H	3.21186600	2.71803600	-4.35479900
H	3.02911900	4.25652300	-4.49267300
H	3.25670700	5.75377600	-2.98926000
H	4.49739600	4.89972400	-3.35803700
H	-3.22541500	-3.38651900	2.07721900

H	-2.40122500	-2.61365600	1.00800500
H	-8.85677700	-3.12348400	-0.41168800
H	5.55980700	-5.97227800	-1.23982800
O	0.77000700	0.65434800	-1.18456400
H	0.44559300	-0.08454400	-1.84423500
H	0.08409500	3.46113600	-2.99085700

$S_3^{TS}Y_z^{\bullet}$ (W1=OH⁻, W2=H_2O, Ox=OH⁻)

Mn	0.08244600	-2.58277100	-1.09778800
Mn	0.60951800	-1.91953900	1.50508000
Mn	1.87022000	-0.13646700	-0.27285400
Mn	1.74318700	2.68617300	-1.32889500
Ca	-1.50464900	0.32792600	-0.02093300
O	-0.82298300	-2.08954600	0.46066400
O	0.71181200	-0.12871800	1.10822100
O	1.62410400	-2.14808500	-0.04959500
O	2.46642600	1.56354500	-0.11042100
O	0.18966000	-1.01092300	-1.70088500
O	3.11614800	3.75623500	-1.37537500
O	0.85861600	3.61254200	-2.89624800
O	-2.92484900	1.85325000	-1.27080400
O	-3.82878400	-0.35253900	0.72962200
H	7.22286700	5.27157200	-0.51777900
C	7.74348300	4.36202500	-0.19108000
H	8.78601800	4.63095200	0.01951500
C	7.68320900	3.27329300	-1.27450800
C	6.26085900	2.98291800	-1.74163400
O	5.39103700	2.75493600	-0.79473700
O	5.98820600	2.95727900	-2.95634900
C	-7.64256300	7.23532500	1.21049000
C	-7.13520600	6.56875700	-0.08907400
C	-6.82788900	5.11930800	0.14067400
C	-7.89232000	4.22755900	0.45749900
C	-5.49889500	4.62436000	0.11919300
C	-7.64651900	2.91865700	0.77857900
C	-5.21723900	3.31933600	0.45908600
C	-6.28995000	2.41594700	0.81853800
O	-6.05829000	1.20621600	1.14938000
H	2.05059700	8.21424300	1.26634600
C	2.42848900	7.92270600	0.27913400
C	1.34446200	7.36445100	-0.63749900
O	1.44245400	7.33543300	-1.85360200
H	3.20173000	7.15369800	0.40863600
N	0.18116900	6.91669700	-0.00830600
C	-0.79524100	6.12765900	-0.76341400

C	-1.88766600	6.97215100	-1.42540100
O	-2.77894300	6.40410100	-2.09502100
C	-1.36567600	4.97925100	0.09064900
C	-0.49137500	3.71154400	0.22446800
O	-0.97285900	2.70620900	0.75641200
O	0.71398000	3.84789900	-0.25476300
N	-1.75943400	8.29702000	-1.27942300
C	-2.47623800	9.31694400	-2.03178300
H	-1.78407900	9.85699000	-2.68867700
H	-5.96030900	-3.98523200	-3.92727300
C	-6.02682400	-4.21044000	-2.85263800
C	-7.47025300	-4.21791000	-2.41977400
O	-7.83197900	-4.71979300	-1.34257900
C	-5.10138200	-3.24356100	-2.08127100
C	-3.72352400	-3.25936100	-2.77284700
C	-2.61430100	-2.48373200	-2.06906700
O	-2.77262600	-1.29683300	-1.72208400
O	-1.53031800	-3.17335100	-1.91666200
N	-8.34149900	-3.62280100	-3.28000200
C	-9.75830800	-3.46668400	-2.99543500
H	-10.33570700	-3.59867400	-3.91583800
C	-10.08655000	-2.07816900	-2.38005500
C	-9.17170800	-1.65783600	-1.26336000
C	-8.59986400	-0.44443300	-0.96688200
N	-8.73021700	-2.52846300	-0.27315700
C	-7.90263600	-1.88603000	0.56004200
N	-7.83000300	-0.61221200	0.17036100
H	3.56652300	-4.69039000	-6.74406000
C	3.66505500	-3.60919800	-6.58962600
C	4.65315900	-3.39910000	-5.45072900
O	4.64540600	-4.13273200	-4.44960800
C	2.29005700	-2.98983900	-6.22221100
C	1.81419400	-3.31876700	-4.84217400
C	1.44262700	-2.50411100	-3.80358000
N	1.66888900	-4.61345700	-4.35348800
C	1.24146900	-4.55568000	-3.07086900
N	1.08909800	-3.28462900	-2.71788900
N	5.46519200	-2.32500000	-5.57011200
C	6.31202300	-1.86896900	-4.47292800
H	6.88920200	-2.72148600	-4.09891900
C	5.46628900	-1.27702100	-3.29773000
C	4.60157900	-0.04628000	-3.63658000
C	3.46193400	0.29232500	-2.66188300
O	3.20485600	-0.50886200	-1.69457300

O	2.80052900	1.34672200	-2.89359600
H	7.75528600	-5.61677600	1.12377500
C	7.10665300	-5.68080400	2.00665500
H	7.56681900	-6.38226100	2.71108400
C	5.68873800	-6.15680300	1.63654700
C	4.96568800	-5.25178300	0.68840300
C	3.94065700	-4.36154200	0.88328000
N	5.25709400	-5.16820700	-0.67265100
C	4.44663600	-4.27532000	-1.27769600
N	3.64735100	-3.77985300	-0.33574000
H	-0.50432400	-7.45305100	3.03411300
C	-0.23966100	-6.40049400	2.85749700
C	-1.32943100	-5.53772800	3.50876100
O	-2.28155100	-5.08750000	2.86951800
C	-0.12851600	-6.15640200	1.35770600
C	0.26850500	-4.74687400	0.93876200
O	0.59266200	-3.90870200	1.85023200
O	0.23962000	-4.51372700	-0.30265600
N	-1.20385100	-5.33794200	4.85667400
C	-2.32943900	-4.87819700	5.67405800
C	-2.81328200	-3.44545700	5.37681500
O	-3.99562000	-3.15052600	5.51098800
N	-1.84289500	-2.54781100	5.04228800
C	-2.19211100	-1.21563100	4.55982600
C	-1.40274500	-0.90275500	3.28087500
O	-1.71891500	0.05377400	2.56256100
O	-0.38979600	-1.70053600	3.08940800
O	-1.98490200	3.91274300	-2.92240400
O	4.02165100	4.71485500	-3.84165900
O	2.24731700	3.13050400	-4.92510500
O	5.80019900	2.34091700	1.90575600
O	-5.05782500	0.20635800	-1.67127100
O	-3.26254500	-2.89897700	1.31620900
H	6.16921600	-1.80921000	5.31262600
C	5.51983000	-0.94062000	5.48394600
H	5.24730100	-0.92601600	6.54584400
C	4.24749700	-1.00721100	4.60503400
C	4.53619400	-1.01465700	3.08880000
C	3.29743800	-1.10598600	2.19414300
O	2.30653500	-1.78535800	2.59422500
O	3.36542100	-0.50390200	1.06359800
H	4.85642400	3.95280300	8.81447400
C	3.90286500	4.48781800	8.71440800
C	3.21820700	4.15845100	7.37832000

C	2.91162500	2.66003100	7.21819700
C	2.05538000	2.30409900	5.99705100
N	2.77383800	2.53132400	4.74305400
C	2.34779800	2.12604800	3.52409000
N	1.13296200	1.56246400	3.39506900
N	3.13346700	2.26212700	2.46546500
O	5.92512400	0.18570900	0.09874100
H	-8.55789700	6.75516000	1.57580300
H	-6.88702000	7.17204200	2.00258800
H	-7.86447100	8.29317600	1.02878200
H	-6.24433600	7.08953800	-0.46021000
H	-7.91246800	6.66185400	-0.86255000
H	-8.91224900	4.60761400	0.45668900
H	-8.45272000	2.24077700	1.05178000
H	-4.20614900	2.92119600	0.41120000
H	-4.69701800	5.29496200	-0.18782100
H	-7.21634000	0.11028100	0.62065500
H	-8.66456200	0.50898600	-1.46998900
H	-7.36352300	-2.33128700	1.38434600
H	-0.94506400	-2.90570300	4.73066500
H	-0.44620500	-5.81464200	5.33125700
H	5.41772100	-1.76798000	-6.41378700
H	-7.97160000	-3.21371300	-4.12918100
H	-10.03664500	-4.27190200	-2.30797000
H	-11.12985300	-2.09281000	-2.03171100
H	-10.03041900	-1.30661400	-3.15799600
H	-3.82209000	-2.82813800	-3.78168100
H	-5.51480700	-2.22690900	-2.07747200
H	-5.65234500	-5.23418700	-2.72187400
H	-3.38215100	-4.29280000	-2.89756200
H	-5.00276900	-3.56286900	-1.03588200
H	7.07644900	-4.68937300	2.47388100
H	3.38187400	-4.09549300	1.77016000
H	4.45380000	-4.04565300	-2.34127800
H	1.36739800	-1.42918100	-3.74557400
H	1.01735600	-5.40446800	-2.44099400
H	4.01465400	-3.17508500	-7.53402800
H	7.01156900	-1.12833300	-4.87320500
H	-0.24277500	5.69373500	-1.60977900
H	0.22366300	6.76159100	0.99268200
H	2.89106700	8.78462300	-0.20969100
H	-0.96258200	8.57997600	-0.71721700
H	7.29126300	4.01817500	0.74387600
H	1.55223600	-3.30548500	-6.97344600

H	2.35219900	-1.89673400	-6.28951400
H	0.72151100	-6.22160900	3.35722000
H	-3.25729600	-1.22993800	4.30109500
C	-1.94429700	-0.12322400	5.61092100
H	-2.22319400	0.85826700	5.20853800
H	-2.54798900	-0.32628600	6.50159300
H	-0.88601100	-0.10695000	5.90612800
H	-2.02178500	-4.93039000	6.72410300
H	-3.20425700	-5.52340400	5.53548800
H	0.59123300	-6.85465500	0.90920000
H	-1.09323000	-6.34959700	0.87381900
H	-2.30779300	4.63023400	-0.34562400
H	-1.61813300	5.32764200	1.10343700
H	6.10462700	-0.03730400	5.26711300
H	3.66097700	-1.89948000	4.85956500
H	5.16742900	-1.88088600	2.82987300
H	3.60713400	-0.14314400	4.83160300
H	5.09718900	-0.12585800	2.78694800
H	8.27338400	3.55342700	-2.15333100
H	8.10710700	2.33824400	-0.87584700
H	5.07789400	-6.23632700	2.54431500
H	5.73459500	-7.16885400	1.20863000
H	2.87523500	-3.06213700	-0.42017000
H	4.10980300	5.56110600	8.79545100
H	3.85542100	4.50939800	6.55084300
H	2.36200000	2.31360700	8.10455000
H	1.76861300	1.24362900	6.03830700
H	3.26643500	4.20073600	9.56163600
H	2.27699200	4.72381600	7.29892500
H	3.84541300	2.07847100	7.18685500
H	3.60189800	3.11415500	4.76172400
H	1.12890700	2.90239700	6.01973400
H	4.17414600	2.35085600	2.49205500
H	0.41309900	1.69293100	4.09101200
H	2.76580900	2.03310300	1.53023600
H	0.86844200	1.09816800	2.51752300
H	4.80385500	-2.06983500	-2.94070300
H	5.20004300	0.87514900	-3.69851900
H	6.13478400	-1.02792000	-2.46324000
H	4.11835600	-0.17108000	-4.61882500
H	1.97014100	-5.45465400	-4.82938100
H	1.32081700	3.44671100	-3.82965000
H	4.00929300	3.27987700	-1.13727600
H	5.65597400	2.78228000	1.02951600

H	6.05460800	1.44576500	1.57786600
H	5.02954200	-0.15795900	0.28415800
H	5.72535200	1.02655100	-0.37950600
H	-2.95984200	10.02869200	-1.35257500
H	-3.23758200	8.82097900	-2.63954700
H	-4.89883900	0.01398000	-0.71543100
H	-4.35997900	-0.38695800	-2.03748500
H	-3.76271900	-1.27542700	1.10765600
H	-2.64406700	2.56055900	-1.89787500
H	-2.29484400	4.82139900	-2.62637700
H	-2.24209800	3.85634700	-3.85944800
H	-4.33247800	0.20382500	1.35379100
H	-3.78250700	1.46276500	-1.58083300
H	2.61876800	2.32970800	-4.49614400
H	2.95626000	3.82481300	-4.68760000
H	3.55733400	4.74433700	-2.97658100
H	4.77004800	4.10008400	-3.64469500
H	-3.17266700	-3.56303300	2.03008500
H	-2.34282500	-2.79836500	0.96952800
H	-8.71644000	-3.55946000	-0.40052700
H	5.97695200	-5.69622500	-1.15359700
O	0.37086800	1.31437700	-1.33083200
H	0.34100300	0.86585300	-2.19749800
H	-0.12675100	3.62010800	-2.93649700

$S_3^{B,W}Y_z^\bullet$ (W1=OH⁻, W2=H_2O, Ox=OH⁻)

Mn	0.26782400	-2.51253800	-1.00443000
Mn	0.72336300	-1.91907700	1.60567100
Mn	1.90931300	-0.26644600	-0.25371100
Mn	1.37594900	2.58735400	-1.58820400
Ca	-1.47417600	0.38008900	-0.02787100
O	-0.69616300	-2.11152900	0.47253700
O	0.75885400	-0.18866600	1.18413800
O	1.78726700	-2.21529200	0.05286100
O	1.99287800	1.49206400	-0.26743100
O	0.52442600	-0.77612800	-1.40597800
O	2.85334300	3.54971100	-1.62405900
O	0.70238000	3.71256400	-3.13895900
O	-3.10390200	1.85003400	-1.13182500
O	-3.76171000	-0.45411400	0.74526800
H	6.94684200	5.63657100	-0.42628700
C	7.53529400	4.76375500	-0.11513000
H	8.56080500	5.10115800	0.07857400
C	7.53283500	3.67932100	-1.20499800
C	6.15331500	3.18819300	-1.65793200

O	5.27106600	2.95919100	-0.73107600
O	5.95741200	2.97651800	-2.87397700
C	-7.97928500	6.88565500	1.20626000
C	-7.42000700	6.23109400	-0.07821700
C	-7.04533000	4.80255400	0.17774300
C	-8.07189800	3.84862700	0.43377000
C	-5.69099800	4.38968100	0.24485900
C	-7.76831000	2.55470500	0.76706800
C	-5.35344500	3.10346200	0.60374800
C	-6.38902500	2.13167300	0.88237100
O	-6.10426600	0.92967000	1.20465800
H	1.67573800	8.29130500	1.33880100
C	2.05178800	8.06189800	0.33444700
C	0.98308200	7.48692500	-0.58941200
O	1.05478800	7.53097300	-1.80727200
H	2.86753100	7.33199800	0.42216800
N	-0.13067800	6.92949300	0.03613000
C	-1.07549400	6.11626800	-0.73146300
C	-2.21725600	6.91705800	-1.36820600
O	-3.10361200	6.31489400	-2.01112000
C	-1.57966900	4.91992300	0.09448000
C	-0.65584500	3.68794200	0.14966900
O	-1.01337700	2.68840200	0.78858600
O	0.44222100	3.82827600	-0.52745100
N	-2.13477600	8.24881600	-1.23855500
C	-2.90164400	9.22438000	-2.00066000
H	-2.24322400	9.76803100	-2.68875200
H	-5.72414400	-4.23867200	-3.95510800
C	-5.78927900	-4.45628800	-2.87900200
C	-7.23281300	-4.53145000	-2.45233500
O	-7.57291600	-5.04529600	-1.37441100
C	-4.91675200	-3.42616000	-2.10418400
C	-3.50465800	-3.38633300	-2.73369300
C	-2.45207200	-2.47981900	-2.07764800
O	-2.69462900	-1.30810700	-1.73572400
O	-1.28479200	-3.03987100	-1.98744800
N	-8.12819100	-3.98848200	-3.32209700
C	-9.55150800	-3.89336000	-3.04110600
H	-10.12020000	-4.05067600	-3.96284400
C	-9.93710400	-2.51751700	-2.42931200
C	-9.04826500	-2.05833700	-1.30617000
C	-8.52240400	-0.82391500	-1.01030500
N	-8.59271700	-2.90607200	-0.30212900
C	-7.80150800	-2.22901500	0.53932000

N	-7.76589600	-0.95675600	0.14043900
H	3.80078900	-4.45862100	-6.70983700
C	3.88290000	-3.37593200	-6.55879800
C	4.87767100	-3.14060800	-5.43096100
O	4.92126300	-3.90057400	-4.44919600
C	2.50302900	-2.77265800	-6.17215400
C	2.04549500	-3.11077900	-4.78605000
C	1.61726800	-2.31437200	-3.75243400
N	1.98287900	-4.40838900	-4.28947200
C	1.55061700	-4.37241800	-3.00885000
N	1.31792100	-3.11186700	-2.66029100
N	5.62167300	-2.01811100	-5.53016800
C	6.43075700	-1.51618500	-4.42221000
H	7.07606200	-2.32635500	-4.06538100
C	5.54439800	-1.01219200	-3.24487200
C	4.57023600	0.13436300	-3.57883500
C	3.36439800	0.27950800	-2.65142700
O	3.31929200	-0.45433100	-1.59813700
O	2.46118100	1.10875800	-2.97509400
H	8.04601600	-5.20545400	1.19051200
C	7.37260200	-5.30471500	2.05131200
H	7.86267000	-5.95137600	2.78734600
C	6.01328800	-5.90185100	1.64245000
C	5.26896300	-5.06476300	0.65166800
C	4.18720100	-4.23441400	0.79102900
N	5.62102700	-4.97333000	-0.69467500
C	4.79483000	-4.13217000	-1.34798200
N	3.92438100	-3.67975400	-0.44709000
H	-0.12467300	-7.44413900	3.06708000
C	0.06504900	-6.38126600	2.85811500
C	-1.06677200	-5.57255000	3.50761900
O	-2.03414700	-5.16258900	2.86398500
C	0.12632400	-6.16893600	1.34676300
C	0.43084700	-4.73587100	0.93642400
O	0.72967000	-3.92088200	1.86763100
O	0.35680600	-4.46628500	-0.30529300
N	-0.95829900	-5.37261200	4.85680100
C	-2.11159200	-4.97029300	5.66684500
C	-2.67440500	-3.57161700	5.34263100
O	-3.87897600	-3.35477300	5.40202800
N	-1.74415900	-2.60972500	5.07296600
C	-2.14532000	-1.30201800	4.56349000
C	-1.35290700	-0.96126700	3.30057700
O	-1.67237000	-0.00991800	2.58246600

O	-0.32026300	-1.74571500	3.12718300
O	-2.23005700	3.85122500	-2.95085300
O	3.90343900	4.47021100	-4.01433300
O	2.10951400	2.88311900	-5.08704500
O	5.43258600	2.83004500	1.99037800
O	-5.10739100	0.01937200	-1.61747200
O	-3.11660000	-2.99305700	1.34159600
H	5.80962500	-1.71281900	5.66909400
C	5.53901900	-0.65731400	5.53248100
H	5.30429100	-0.23936500	6.51904500
C	4.33439100	-0.51060500	4.58223300
C	4.61378100	-1.05816100	3.16469600
C	3.38857000	-1.06795300	2.25901500
O	2.39610000	-1.77720700	2.64882600
O	3.42982800	-0.41025800	1.17227900
H	4.69285900	4.35413100	8.77650100
C	3.64342500	4.67682200	8.76854600
C	2.97058900	4.35842800	7.41793200
C	2.97601800	2.85597700	7.07911500
C	2.05940100	2.42609100	5.92331600
N	2.56262100	2.81210700	4.59960100
C	2.09440700	2.29507400	3.43741100
N	0.94948400	1.58274200	3.44987300
N	2.76547500	2.45842000	2.30682800
O	5.94691600	0.54086100	0.42914000
H	-8.87263500	6.36077600	1.56488800
H	-7.23232500	6.87148700	2.00881400
H	-8.25413500	7.92812800	1.00857800
H	-6.54927900	6.79051500	-0.44085300
H	-8.18870100	6.27651800	-0.86390400
H	-9.11071100	4.16814900	0.37245600
H	-8.54837200	1.82916200	0.98884700
H	-4.31916500	2.77095600	0.61682900
H	-4.91291500	5.10741900	-0.01158500
H	-7.19001500	-0.20994400	0.60169200
H	-8.60944100	0.12299600	-1.52211800
H	-7.26190400	-2.64881500	1.37655600
H	-0.81083700	-2.91472100	4.81529200
H	-0.18691100	-5.82284700	5.33544700
H	5.52706000	-1.44457700	-6.35897200
H	-7.77480300	-3.56405500	-4.17079600
H	-9.79737100	-4.70841700	-2.35293200
H	-10.98148600	-2.57173000	-2.08812600
H	-9.90561200	-1.74586500	-3.20854300

H	-3.58755000	-3.04526500	-3.77849500
H	-5.37718000	-2.43028400	-2.13688200
H	-5.36261100	-5.45698000	-2.73163000
H	-3.08549200	-4.39806400	-2.76746200
H	-4.84475500	-3.71670800	-1.04820100
H	7.24448400	-4.30971000	2.49367500
H	3.57509900	-3.98736600	1.64774100
H	4.83571700	-3.90612700	-2.41335300
H	1.48415500	-1.24336300	-3.70830800
H	1.37879000	-5.23172100	-2.37662400
H	4.21150800	-2.93639100	-7.50803300
H	7.06868400	-0.71622500	-4.81088300
H	-0.51437400	5.72711500	-1.59385600
H	-0.07993400	6.76508900	1.03492500
H	2.46013300	8.96726000	-0.12299400
H	-1.33407300	8.57437000	-0.70763500
H	7.12085800	4.38758800	0.82630400
H	1.75946200	-3.09852500	-6.91325700
H	2.54927600	-1.67903900	-6.24072000
H	1.02189000	-6.12359100	3.32930800
H	-3.20321700	-1.37566900	4.28453200
C	-1.97583400	-0.18540100	5.60532200
H	-2.29882900	0.77479400	5.18529500
H	-2.58491700	-0.40998200	6.48695200
H	-0.92602500	-0.10841900	5.91993200
H	-1.80273100	-4.98922200	6.71765900
H	-2.94737800	-5.66647700	5.53470000
H	0.88280300	-6.82573600	0.89579800
H	-0.83331900	-6.42965400	0.88560900
H	-2.51207500	4.54405200	-0.34328600
H	-1.82758500	5.21472100	1.12453100
H	6.41897700	-0.13023100	5.14212300
H	3.46243900	-1.03431800	4.99673900
H	4.95006000	-2.10338100	3.24747700
H	4.06060400	0.55022300	4.49949000
H	5.40905600	-0.48944300	2.67228100
H	8.06130300	4.02246600	-2.10098600
H	8.07794100	2.79502800	-0.83613400
H	5.37525300	-6.01304600	2.52778500
H	6.15386100	-6.91349600	1.23509900
H	3.13684000	-3.00907600	-0.56440900
H	3.62118500	5.75375300	8.97075500
H	3.46472500	4.93012300	6.61735600
H	2.63166100	2.29937600	7.96259300

H	1.94526300	1.33275800	5.93150700
H	3.12756000	4.16546200	9.59175600
H	1.92727500	4.70881800	7.44395400
H	4.00121100	2.50624300	6.88209000
H	3.38622900	3.39957400	4.53781000
H	1.06052800	2.86382100	6.08355300
H	3.79852400	2.65690000	2.27798100
H	0.23954100	1.75163300	4.14863500
H	2.35172600	2.14012800	1.42070600
H	0.66481500	1.09654700	2.59433600
H	4.96264100	-1.86413600	-2.88386200
H	5.07215400	1.11717900	-3.53318500
H	6.19532100	-0.70525900	-2.41587400
H	4.16397100	0.03380600	-4.59605200
H	2.35315000	-5.22736900	-4.75565000
H	1.15026700	3.41332500	-4.03167400
H	3.71066500	3.17638400	-1.22929700
H	5.39799500	3.19589600	1.06710400
H	5.76515400	1.92482800	1.77807000
H	5.05991300	0.14037200	0.52616500
H	5.76003700	1.32787300	-0.13914800
H	-3.39292000	9.93885800	-1.32981900
H	-3.65963500	8.68632100	-2.57580100
H	-4.87067800	-0.19431800	-0.68189900
H	-4.37954000	-0.48077100	-2.04974300
H	-3.67088500	-1.36941100	1.13015800
H	-2.76336700	2.46444800	-1.82604500
H	-2.55807200	4.73824000	-2.62952600
H	-2.57795800	3.77471100	-3.85601900
H	-4.33004200	0.07635500	1.33866700
H	-3.92410600	1.41378600	-1.46888800
H	2.37276400	2.09016500	-4.57361600
H	2.85973900	3.53194600	-4.85977700
H	3.41071100	4.47369200	-3.16233700
H	4.70616700	3.95150000	-3.76960000
H	-3.00456300	-3.66190700	2.04787100
H	-2.20451800	-2.86504200	0.98889800
H	-8.53849800	-3.93482400	-0.42374200
H	6.39330700	-5.46286000	-1.13348600
O	-0.23584200	1.60238600	-1.82977700
H	0.00175200	0.86857200	-2.42643500
H	-0.27673500	3.59229400	-3.16591900

$S_3^{A,W}Y_z$ (W1=H_2O, W2=OH^-, Ox=oxo)

Mn	-0.12387500	-2.47117200	-1.11173000

Mn	0.54567100	-1.71868900	1.49447500
Mn	1.94052500	0.13443800	-0.14540500
Mn	1.67146200	2.43475400	-1.58784300
Ca	-1.47178300	0.36552300	-0.31222000
O	-0.90482800	-1.91383000	0.50438800
O	0.67173000	0.07284600	1.17888000
O	1.47793400	-1.82988000	-0.14617100
O	2.58539200	1.79393600	-0.05927300
O	-0.43369400	-1.07890400	-1.95721400
O	3.00676500	3.95906300	-1.83289900
O	0.71636500	3.02332800	-3.01841700
O	-2.76166100	1.79038900	-1.69732200
O	-3.21966200	0.05531400	1.38330800
H	7.72440800	5.06443300	-0.47492800
C	7.82780400	4.06613100	-0.02977000
H	8.83557800	3.99083700	0.39987700
C	7.59327100	2.96996000	-1.07706700
C	6.20835300	3.06215500	-1.72311400
O	5.24835000	3.32413200	-0.89784000
O	6.09296800	2.88018300	-2.95497100
C	-7.45237700	7.38536700	1.53462600
C	-7.01948900	6.75989000	0.19850700
C	-6.65284000	5.29126300	0.28622900
C	-7.58129200	4.34236300	0.73721500
C	-5.38723500	4.83044500	-0.10800000
C	-7.26727400	2.98421200	0.80064300
C	-5.05393600	3.47548700	-0.05710400
C	-5.99827000	2.55094200	0.39863800
O	-5.65219400	1.21341500	0.42312600
H	2.21769400	8.11419000	1.55128800
C	2.63363000	7.77825900	0.59454000
C	1.66697800	6.97140500	-0.24707400
O	2.00387600	6.51546100	-1.34369400
H	3.52579200	7.17090400	0.78547300
N	0.39526300	6.82684000	0.24247100
C	-0.64956600	6.13014500	-0.50397900
C	-1.70741500	7.06212800	-1.09858700
O	-2.65996700	6.57918000	-1.72601700
C	-1.24260700	4.93310400	0.25121600
C	-0.46922500	3.61182400	0.09209900
O	-0.96482300	2.58322800	0.57042500
O	0.64653900	3.70447900	-0.55486400
N	-1.47541000	8.38675300	-0.95467200
C	-2.22806400	9.41953300	-1.64433700

H	-1.63451800	9.87349100	-2.44803900
H	-6.10590500	-3.58133200	-3.99881500
C	-6.22247200	-3.94353100	-2.96692100
C	-7.68548100	-3.98508000	-2.58835500
O	-8.08478600	-4.60998400	-1.59307500
C	-5.34082500	-3.11276700	-2.03798400
C	-3.91380000	-3.26482600	-2.56082400
C	-2.85322400	-2.55608900	-1.75789400
O	-3.06165100	-1.41144300	-1.30718700
O	-1.76522800	-3.24837600	-1.64584000
N	-8.53172800	-3.30002200	-3.41114700
C	-9.92764700	-3.07332200	-3.06805100
H	-10.54250600	-3.12995200	-3.97275800
C	-10.16296400	-1.71170300	-2.36832100
C	-9.19555600	-1.38825100	-1.26847200
C	-8.55118700	-0.21765400	-0.93935600
N	-8.77231100	-2.30747000	-0.31629100
C	-7.87350900	-1.68707300	0.49112600
N	-7.74103800	-0.41057000	0.16612200
H	3.24216500	-4.57569000	-6.86994400
C	3.47539300	-3.51741100	-6.71250400
C	4.60707600	-3.44502100	-5.68953800
O	4.75783200	-4.33354000	-4.83503700
C	2.23668600	-2.75393600	-6.18514600
C	1.84210300	-3.08261200	-4.77494300
C	1.12872800	-2.35731900	-3.85493500
N	2.07532200	-4.31926100	-4.17255400
C	1.53673200	-4.29372000	-2.92777200
N	0.93589000	-3.12999400	-2.72520800
N	5.37872500	-2.32455800	-5.71097000
C	6.18281800	-1.94682500	-4.54053500
H	6.71844100	-2.84068000	-4.21191200
C	5.29257400	-1.42307900	-3.38090900
C	4.64111300	-0.04578300	-3.58854200
C	3.58108600	0.35383000	-2.55270500
O	3.35339700	-0.38967200	-1.54909400
O	2.98076300	1.45267200	-2.78394100
H	7.31041900	-6.19751900	0.79615900
C	6.86679900	-6.02708700	1.78639000
H	7.28463500	-6.78239700	2.46114600
C	5.33470600	-6.13264700	1.75479000
C	4.67207100	-5.10369800	0.89664800
C	3.62461100	-4.25175300	1.13549200
N	5.04339600	-4.86127500	-0.42413500

C	4.25716400	-3.90152800	-0.95003000
N	3.39027200	-3.52914900	-0.01894000
H	-0.84902400	-7.57908500	2.62561400
C	-0.49721500	-6.53814600	2.63519300
C	-1.56722800	-5.69839400	3.33785700
O	-2.52863200	-5.21780800	2.73327900
C	-0.27418300	-6.07497100	1.19504600
C	0.21139900	-4.64437400	0.92306400
O	0.46230900	-3.83421000	1.87444500
O	0.31928900	-4.37434600	-0.30742900
N	-1.42720800	-5.54768700	4.68972000
C	-2.52936800	-5.05675000	5.51502200
C	-2.96563700	-3.60847100	5.27448800
O	-4.11525200	-3.27642200	5.56282000
N	-1.99256700	-2.76314600	4.85651700
C	-2.27464600	-1.36092400	4.54068600
C	-0.99755700	-0.77084600	3.91409200
O	-0.62571200	0.36789700	4.20297900
O	-0.40191300	-1.62206200	3.12791100
O	-1.83996300	3.94259000	-2.94900400
O	3.99404000	3.98676900	-4.40819100
O	2.13086800	2.24426500	-5.27688000
O	5.47124100	1.55248800	1.16525700
O	-4.98487900	0.34075100	-2.19889800
O	-3.30645800	-2.70908100	1.48721600
H	6.30022600	-1.94300300	5.05904400
C	5.44438700	-1.37368300	5.44719000
H	5.25344500	-1.71720400	6.47196600
C	4.21483200	-1.54325500	4.56045800
C	4.50182500	-1.08412200	3.13381200
C	3.29300600	-1.06338500	2.22956200
O	2.28881000	-1.77380700	2.53079900
O	3.38464200	-0.33324700	1.19115500
H	4.97020600	3.44338000	8.96494400
C	4.01381300	3.97749500	8.88773100
C	3.39220500	3.81081300	7.48797200
C	3.10853900	2.34498400	7.11017100
C	2.29902900	2.14274200	5.82195500
N	3.05776000	2.51954600	4.62459000
C	2.55164200	2.44153300	3.36750000
N	1.35649400	1.92219800	3.13512700
N	3.27173400	2.96387200	2.34589200
O	5.92760100	-1.00262900	-0.14031500
H	-8.33449500	6.87950300	1.94758400

H	-6.65033300	7.31090500	2.28043000
H	-7.70497400	8.44632600	1.40814300
H	-6.16163400	7.31683800	-0.20260000
H	-7.83453500	6.89263600	-0.53038900
H	-8.57679300	4.66512900	1.04073100
H	-8.00002800	2.26140600	1.15237300
H	-4.08859500	3.11084000	-0.40384300
H	-4.65368600	5.53944700	-0.49329400
H	-6.48447000	0.62718300	0.48713700
H	-8.62031900	0.74971300	-1.42023700
H	-7.34141700	-2.19412500	1.28522300
H	-1.19930500	-3.15891600	4.35870200
H	-0.65083100	-6.01009100	5.14438300
H	5.10567800	-1.58523500	-6.34787500
H	-8.11363100	-2.70310900	-4.11454200
H	-10.21889100	-3.90057200	-2.41448000
H	-11.20021600	-1.70390400	-1.99860700
H	-10.09792700	-0.90388900	-3.10891200
H	-3.85182500	-2.85536000	-3.58243500
H	-5.65304600	-2.06182800	-2.04563500
H	-5.87347200	-4.98497500	-2.95523400
H	-3.64238200	-4.32419100	-2.63090100
H	-5.41392600	-3.47585900	-1.00514300
H	7.18819000	-5.03800400	2.13361600
H	3.01514600	-4.09827300	2.01448500
H	4.32935700	-3.52577800	-1.95828500
H	0.71060600	-1.36097000	-3.89291700
H	1.56572100	-5.10704500	-2.21808400
H	3.77201400	-3.09457300	-7.67995600
H	6.91874800	-1.20041000	-4.85663600
H	-0.12188200	5.72826700	-1.37950300
H	0.20685400	7.11084800	1.19695400
H	2.95025500	8.65343800	0.01550500
H	-0.60501300	8.63563400	-0.50167100
H	7.09896600	3.98517600	0.78298000
H	1.39922300	-2.95945400	-6.86735300
H	2.41348600	-1.67195100	-6.24224000
H	0.43882100	-6.51716500	3.20937000
H	-3.04533300	-1.31633300	3.75648000
C	-2.72784300	-0.56419400	5.75777500
H	-2.90335800	0.47622900	5.46739300
H	-3.64845600	-0.99448100	6.15971400
H	-1.95441000	-0.57539500	6.53575200
H	-2.22561800	-5.14662600	6.56347300

H	-3.42472200	-5.67095500	5.36644000
H	0.42889700	-6.74945600	0.68903100
H	-1.21987000	-6.15106900	0.64499200
H	-2.25138400	4.73287300	-0.12481900
H	-1.34994000	5.14149400	1.32432800
H	5.74921700	-0.31907900	5.50030500
H	3.88146500	-2.58923100	4.55379400
H	5.24584200	-1.73105900	2.64253000
H	3.37453400	-0.96189500	4.96617200
H	4.94147300	-0.07860900	3.12033800
H	8.34573000	3.00429000	-1.87299000
H	7.67023900	1.98168900	-0.59811100
H	4.93005300	-6.02481400	2.76835000
H	5.04341900	-7.13746100	1.41341700
H	2.59271100	-2.78495500	-0.12703800
H	4.19969900	5.03503400	9.10970500
H	4.06151300	4.27335300	6.74496700
H	2.52953200	1.87887500	7.91964400
H	2.01521600	1.08676400	5.72697000
H	3.34531400	3.58095400	9.66321100
H	2.44958100	4.37654400	7.44066000
H	4.05000200	1.77943900	7.04096800
H	3.79274000	3.20554900	4.74744700
H	1.36458000	2.72327900	5.87604100
H	4.28023300	2.78456200	2.36205400
H	0.76254700	1.45397300	3.82947900
H	2.90631500	2.70747900	1.40956400
H	1.12173700	1.59075100	2.19245700
H	4.48731800	-2.15483600	-3.24350800
H	5.38290400	0.76749900	-3.56751300
H	5.88168100	-1.40089500	-2.45516200
H	4.14645800	0.02257600	-4.56964800
H	2.83503100	-4.93203100	-4.46981400
H	1.12191000	2.78229600	-3.89699400
H	3.94494100	3.71299400	-1.42342900
H	5.39364600	2.22382200	0.43166400
H	4.63566900	1.04178100	1.09035900
H	5.00130500	-0.93212000	-0.43807400
H	6.05005300	-0.14342800	0.32190700
H	-2.53912300	10.20367400	-0.94491200
H	-3.11518500	8.95279600	-2.07929200
H	-5.31189100	0.64301600	-1.32235700
H	-4.37185100	-0.39493400	-1.95243700
H	-3.40091400	-0.91212300	1.50289900

H	-2.47324200	2.60982900	-2.19226900
H	-2.01415500	4.81839300	-2.55073500
H	-0.86142900	3.75822500	-2.96013500
H	-4.07821800	0.47590700	1.15696700
H	-3.59103500	1.43290800	-2.10405900
H	2.38201900	1.56317100	-4.62679500
H	2.85468300	2.91828500	-5.10210900
H	3.46172900	4.15628300	-3.59827900
H	4.75726900	3.50296400	-4.00145500
H	-3.18910400	-3.41597000	2.15173600
H	-2.40744500	-2.61419600	1.08753000
H	-8.81567700	-3.32204800	-0.45639000
H	5.81722300	-5.29978900	-0.90767600
O	0.79544800	0.89108800	-1.32399100
H	2.66287900	4.82543500	-1.51457300

$S_3^{TS}Y_z$ (W1=H_2O, W2=OH^-, Ox=oxo)

Mn	0.09994600	-2.47637100	-1.03965400
Mn	0.58354600	-1.81055600	1.56173900
Mn	1.78294800	-0.01815900	-0.27504100
Mn	1.41064800	2.60274500	-1.62852600
Ca	-1.49136300	0.39874600	-0.22865200
O	-0.83452400	-2.04931300	0.48428500
O	0.59793500	-0.05298500	1.13562500
O	1.61719500	-2.06677200	0.03293500
O	2.27053800	1.65495400	-0.17302200
O	0.29228400	-0.84362500	-1.53068000
O	2.98313600	3.97788600	-1.65881800
O	0.74569900	3.56337000	-3.04963700
O	-2.88855500	1.81050900	-1.53283900
O	-3.24735200	-0.11003400	1.39943100
H	7.46638700	5.36144400	-0.44837800
C	7.67348800	4.40238000	0.04493300
H	8.66042700	4.47453000	0.52087400
C	7.62549700	3.24450500	-0.96204000
C	6.25472000	3.13444800	-1.63832900
O	5.25404900	3.17421100	-0.82420400
O	6.18926200	3.02196200	-2.88222300
C	-7.73819600	7.13264600	1.45001000
C	-7.34945300	6.48027300	0.11367900
C	-6.91946800	5.03374900	0.24408400
C	-7.83110400	4.04827200	0.64830800
C	-5.60682400	4.63197800	-0.04425300
C	-7.45699000	2.70872700	0.76298900
C	-5.21176600	3.29748600	0.06235500

C	-6.14185100	2.33416500	0.46283800
O	-5.73615200	1.01291000	0.52734300
H	1.88964100	8.23639700	1.55191500
C	2.33354400	7.91272400	0.60312800
C	1.42833800	7.03316500	-0.23530900
O	1.82310800	6.54132900	-1.29346800
H	3.26232900	7.36938400	0.80984400
N	0.14285000	6.88087400	0.21894600
C	-0.87259800	6.13375300	-0.51862000
C	-1.95936100	7.02204300	-1.12745000
O	-2.90615100	6.50513900	-1.73576100
C	-1.43961800	4.94534200	0.26635000
C	-0.58098200	3.67305900	0.19104700
O	-1.00511400	2.64127700	0.72943300
O	0.52826500	3.82449500	-0.46311400
N	-1.76592600	8.35670700	-1.00442200
C	-2.56644000	9.35450900	-1.68899500
H	-2.00855100	9.81743500	-2.51321800
H	-5.93081400	-3.88086200	-4.04708500
C	-6.02685800	-4.15903700	-2.98730100
C	-7.48603500	-4.24241800	-2.60370600
O	-7.86538600	-4.86811300	-1.60198600
C	-5.16581700	-3.19577900	-2.13730300
C	-3.72724100	-3.24877900	-2.68280900
C	-2.65908100	-2.43990800	-1.95398700
O	-2.90136600	-1.31279000	-1.47467900
O	-1.50003500	-3.01542100	-1.95215900
N	-8.35198400	-3.60638200	-3.44559500
C	-9.76184700	-3.43373800	-3.12733900
H	10.35711500	-3.52030400	-4.04257900
C	10.06028300	-2.07722900	-2.44150000
C	-9.13176700	-1.71835600	-1.31912500
C	-8.52622500	-0.52968900	-0.98056300
N	-8.71469500	-2.62020800	-0.34766200
C	-7.85501500	-1.97209200	0.48017700
N	-7.74426400	-0.69524100	0.14974300
H	3.49014500	-4.43841900	-6.80811000
C	3.68266100	-3.37276700	-6.64333400
C	4.78028000	-3.25701100	-5.58678400
O	4.90333400	-4.11483000	-4.69701100
C	2.40441400	-2.65221400	-6.14545600
C	2.00060500	-3.00086700	-4.74502000
C	1.38020500	-2.26145100	-3.77043900
N	2.15038900	-4.27746300	-4.20354800

C	1.65643900	-4.26784200	-2.94152700
N	1.16645300	-3.06843000	-2.66731600
N	5.54407700	-2.13279400	-5.63033700
C	6.30620500	-1.68904300	-4.45325700
H	6.88581400	-2.54313400	-4.09349200
C	5.36125400	-1.19440500	-3.32196300
C	4.58729400	0.11309800	-3.57955000
C	3.43105400	0.41417500	-2.60867000
O	3.22999900	-0.38329100	-1.62616500
O	2.73554000	1.44068200	-2.85583100
H	7.66589100	-5.69053500	0.96597700
C	7.08717700	-5.71271000	1.89863600
H	7.58035800	-6.42019900	2.57506800
C	5.63079200	-6.12847400	1.65405300
C	4.90598700	-5.17146100	0.77023900
C	3.85159300	-4.33026800	1.00599500
N	5.26114900	-4.94602700	-0.55807100
C	4.45760000	-4.00779400	-1.09662200
N	3.59705700	-3.63619000	-0.15846200
H	-0.53136600	-7.56716900	2.76851900
C	-0.25928400	-6.50559900	2.68041700
C	-1.36722200	-5.69320200	3.35972900
O	-2.33910200	-5.25433200	2.74085200
C	-0.10452300	-6.14373700	1.21084300
C	0.28840500	-4.70171200	0.90238900
O	0.56298800	-3.90590000	1.85809900
O	0.30009800	-4.42357100	-0.33606100
N	-1.24533200	-5.52771200	4.71274100
C	-2.37486600	-5.09195000	5.53409900
C	-2.86937600	-3.65844900	5.30429200
O	-4.03538900	-3.37177900	5.57278400
N	-1.92254800	-2.77462500	4.90750200
C	-2.25430200	-1.39320400	4.54511900
C	-1.01915300	-0.79691100	3.86560000
O	-0.68569400	0.37010600	4.05347600
O	-0.40920700	-1.69208300	3.13303200
O	-1.92585800	3.85165000	-2.92613700
O	4.02937600	4.18097400	-4.21008000
O	2.14109100	2.57163600	-5.28302900
O	5.47564000	1.46193300	1.19428500
O	-5.06388400	0.27973600	-2.14390100
O	-3.25139600	-2.86036700	1.36875800
H	6.25269000	-1.77423000	5.18993500
C	5.45037400	-1.10248100	5.52431400

H	5.22268600	-1.34512000	6.56977000
C	4.19992600	-1.24246300	4.64165900
C	4.47540500	-0.92103600	3.16744100
C	3.25136500	-0.96826600	2.26185700
O	2.28143700	-1.71166400	2.61736500
O	3.29494300	-0.27915600	1.19294700
H	4.76102200	3.72370300	9.04376700
C	3.78047300	4.20373500	8.92644100
C	3.24870200	4.05032300	7.48055400
C	3.06849000	2.58545700	7.02779600
C	2.26894800	2.35977300	5.72977100
N	3.00968300	2.69653100	4.50623300
C	2.52331800	2.47742600	3.25465600
N	1.36609100	1.87169200	3.05193700
N	3.22667900	2.93830600	2.19030100
O	5.79085900	-1.12166100	-0.08838700
H	-8.56956200	6.59737900	1.92688600
H	-6.89351200	7.12079500	2.15109500
H	-8.04878900	8.17600400	1.30576500
H	-6.53618900	7.05556400	-0.34948300
H	-8.20580600	6.55085900	-0.57469000
H	-8.86061900	4.32826400	0.87128800
H	-8.17833900	1.95594600	1.07293300
H	-4.20879400	2.97785100	-0.21377400
H	-4.88353900	5.36959000	-0.39396900
H	-6.54429100	0.39216200	0.54350300
H	-8.60362100	0.43227900	-1.47079200
H	-7.33532200	-2.46077300	1.29377800
H	-1.10720200	-3.14974200	4.43089700
H	-0.45862900	-5.96604400	5.17409200
H	5.27995600	-1.42308400	-6.30358000
H	-7.95174200	-3.00899400	-4.15890900
H	10.03259500	-4.26762800	-2.47318000
H	11.10610100	-2.10179500	-2.09759000
H	10.00293400	-1.27165200	-3.18508700
H	-3.72269300	-2.88347300	-3.72288300
H	-5.56521500	-2.17524400	-2.19277300
H	-5.62256600	-5.17483900	-2.88732700
H	-3.37758300	-4.28686200	-2.72139900
H	-5.18420400	-3.49907600	-1.08275400
H	7.13773900	-4.71310700	2.34601300
H	3.25208800	-4.16147300	1.89005400
H	4.51422800	-3.66051300	-2.11857200
H	1.05547400	-1.22986700	-3.75444500

H	1.63331200	-5.11633700	-2.27373600
H	3.98746800	-2.93997100	-7.60366800
H	7.00345000	-0.90908200	-4.77510300
H	-0.32841100	5.72129900	-1.37965400
H	-0.07766400	7.20181400	1.15460200
H	2.59688900	8.79974600	0.01482800
H	-0.89416100	8.63685600	-0.57280800
H	6.91989700	4.25832900	0.82635400
H	1.58770000	-2.88387800	-6.84412800
H	2.54641700	-1.56514100	-6.19186400
H	0.68671200	-6.36974600	3.22073400
H	-3.04520600	-1.40207700	3.77925200
C	-2.71089300	-0.56664900	5.74044800
H	-2.93927900	0.45314200	5.41530200
H	-3.60326800	-1.01994100	6.18032000
H	-1.92065800	-0.51601200	6.49972600
H	-2.07603400	-5.18222700	6.58386200
H	-3.24294900	-5.74063500	5.37063000
H	0.63647900	-6.79824800	0.73283400
H	-1.05180200	-6.31044800	0.68528000
H	-2.41258500	4.67060300	-0.15538700
H	-1.61716800	5.19662100	1.32080300
H	5.84359500	-0.07703000	5.49363700
H	3.78937100	-2.25701600	4.72319800
H	5.19464300	-1.63367400	2.73253400
H	3.41197300	-0.56955200	5.01068700
H	4.94157200	0.06469300	3.05623500
H	8.39168800	3.35360200	-1.73772900
H	7.81939400	2.29740900	-0.43521600
H	5.08904400	-6.17892000	2.60604700
H	5.59482600	-7.13933000	1.22239700
H	2.79006100	-2.91872400	-0.20283700
H	3.88863400	5.26283700	9.18794600
H	3.92631300	4.57906900	6.79189900
H	2.51851000	2.04877200	7.81405800
H	1.99318600	1.29956600	5.66337300
H	3.09189600	3.74322700	9.64689200
H	2.27766300	4.56237300	7.40386900
H	4.04457300	2.08456200	6.94476800
H	3.70529100	3.42971300	4.57222500
H	1.32914000	2.93309200	5.77316900
H	4.23721300	2.76429500	2.20057100
H	0.74201100	1.49324500	3.76728300
H	2.83371800	2.63465800	1.28223000

H	1.13732100	1.49859800	2.12295000
H	4.62444000	-1.98784300	-3.15307000
H	5.24577800	0.99440100	-3.52701200
H	5.93617700	-1.09972700	-2.39259000
H	4.14094000	0.12783900	-4.58601500
H	2.85067600	-4.93296100	-4.54067700
H	1.10827900	3.23319600	-3.91396600
H	3.88545900	3.68656200	-1.27140900
H	5.45480900	2.12813700	0.44532700
H	4.62548800	0.97912800	1.08814300
H	4.89326200	-0.97630200	-0.44318500
H	5.96508100	-0.26662700	0.36496400
H	-2.88731800	10.13833300	-0.99325000
H	-3.44747300	8.85278700	-2.09681800
H	-5.37920600	0.50336400	-1.24052500
H	-4.37330400	-0.40176900	-1.96656200
H	-3.40028000	-1.08863600	1.47255200
H	-2.52899600	2.57234200	-2.07890300
H	-2.16521900	4.73020200	-2.57223000
H	-0.92419000	3.77880700	-2.97541600
H	-4.11897900	0.30080100	1.20529100
H	-3.70559500	1.46411700	-1.96504800
H	2.30654800	1.87427700	-4.61917000
H	2.88447200	3.20028200	-5.05015600
H	3.47313500	4.25747900	-3.40089000
H	4.79931600	3.67693600	-3.84619000
H	-3.12298700	-3.54136400	2.05866800
H	-2.34392400	-2.74415200	0.99359200
H	-8.71731000	-3.63528700	-0.48843700
H	6.03102200	-5.38878500	-1.04458700
O	0.22074200	1.42978300	-1.56009600
H	2.68276000	4.86504400	-1.36336700

$S_3^{B,W}Y_z$ (W1=H_2O, W2=OH^-, Ox=oxo)

Mn	0.22204900	-2.39661500	-0.99848900
Mn	0.66383300	-1.82660200	1.62623700
Mn	1.83621400	-0.10043500	-0.20576700
Mn	1.12131500	2.56094700	-1.82059300
Ca	-1.47302600	0.42152300	-0.22201400
O	-0.74338800	-2.04722300	0.49298600
O	0.63938000	-0.09685600	1.22498200
O	1.72918800	-2.08875800	0.08861800
O	1.88619600	1.61411400	-0.27229800
O	0.47763400	-0.64096300	-1.37183900
O	2.81906700	3.90407800	-1.70546400

O	0.67949700	3.62501400	-3.26789200
O	-3.00286200	1.82267800	-1.43265600
O	-3.20443700	-0.16827800	1.41396700
H	7.32125700	5.60040900	-0.43225700
C	7.55972700	4.64955000	0.06265100
H	8.55025400	4.74841300	0.52558600
C	7.53015000	3.48394200	-0.93875900
C	6.15652700	3.29130000	-1.59956900
O	5.15813700	3.25529100	-0.78315100
O	6.09226500	3.17470100	-2.84367700
C	-7.92797100	6.92684800	1.43882500
C	-7.63502900	6.23817100	0.09675900
C	-7.14268900	4.81518400	0.24966800
C	-8.01825400	3.79212500	0.63895200
C	-5.80255800	4.47578500	0.01346900
C	-7.58374000	2.47369300	0.78464300
C	-5.34673200	3.16456600	0.15343400
C	-6.24265600	2.16091100	0.53254300
O	-5.77594800	0.86073500	0.61988500
H	1.66516800	8.30550200	1.55727300
C	2.11771900	8.00197300	0.60584600
C	1.25665100	7.06070100	-0.21571800
O	1.68475500	6.53702200	-1.24344000
H	3.08092900	7.51966200	0.80348800
N	-0.03538000	6.89560000	0.22164900
C	-1.03276700	6.12709100	-0.51731800
C	-2.14285200	6.98714100	-1.12426400
O	-3.08382000	6.44532000	-1.71881700
C	-1.57813000	4.92519700	0.26118900
C	-0.71780600	3.66606200	0.12137300
O	-1.04407200	2.63663300	0.73225900
O	0.28114500	3.81659700	-0.68419300
N	-1.97999700	8.32753500	-1.01399500
C	-2.81899200	9.29428100	-1.69657600
H	-2.29339500	9.75412700	-2.54362100
H	-5.78404000	-4.05022200	-4.03657100
C	-5.87738800	-4.31783400	-2.97394500
C	-7.33439800	-4.44511500	-2.59476600
O	-7.69700800	-5.08668000	-1.59708600
C	-5.05064200	-3.31069900	-2.12879600
C	-3.58879600	-3.34380000	-2.61859800
C	-2.56428100	-2.43388600	-1.93616200
O	-2.87509600	-1.33443900	-1.43507900
O	-1.35283500	-2.89092400	-2.00047800

N	-8.21685400	-3.83354000	-3.43754400
C	-9.63192600	-3.70326000	-3.12110600
H	-10.22252200	-3.80054200	-4.03824500
C	-9.96846300	-2.35939900	-2.42767000
C	-9.05480500	-1.98307100	-1.29858600
C	-8.48111800	-0.78143300	-0.95007100
N	-8.62348200	-2.87885000	-0.32760600
C	-7.78640900	-2.21335300	0.50980700
N	-7.70410100	-0.93286700	0.18576900
H	3.63434800	-4.32297000	-6.76756300
C	3.81048900	-3.25251600	-6.61661800
C	4.88593500	-3.10063900	-5.54305500
O	5.01142800	-3.94348900	-4.63923900
C	2.51523400	-2.53709300	-6.14334400
C	2.10173500	-2.87220800	-4.74163600
C	1.52496000	-2.11760400	-3.74917400
N	2.20580700	-4.15789600	-4.21354600
C	1.73240900	-4.14457400	-2.94530000
N	1.29943300	-2.92797100	-2.64896300
N	5.62049400	-1.95890200	-5.59265300
C	6.37979000	-1.48803200	-4.42580800
H	7.01530800	-2.31209600	-4.08886700
C	5.44284000	-1.05399700	-3.27131100
C	4.59624400	0.20449600	-3.52294300
C	3.39890800	0.40508900	-2.58504900
O	3.32992500	-0.31809100	-1.52071600
O	2.54878400	1.26627400	-2.92764900
H	7.87731000	-5.44532400	1.02177700
C	7.26896900	-5.47488800	1.93505600
H	7.76710400	-6.14759800	2.64260700
C	5.84069200	-5.95769700	1.65010000
C	5.10386900	-5.05385900	0.72100900
C	4.03972600	-4.21642300	0.92042400
N	5.45007200	-4.88923700	-0.61881900
C	4.63182200	-3.99264700	-1.20370900
N	3.77105200	-3.58789700	-0.27728300
H	-0.28025900	-7.55204900	2.82103000
C	-0.05216300	-6.48259200	2.70713900
C	-1.18307600	-5.69838200	3.38296300
O	-2.16767000	-5.29139400	2.76239600
C	0.06730700	-6.14524100	1.22458100
C	0.40103700	-4.69085200	0.90109000
O	0.66702800	-3.90234900	1.86554700
O	0.37662100	-4.39679000	-0.33523000

N	-1.06652000	-5.52149200	4.73543700
C	-2.21302500	-5.12652200	5.55482600
C	-2.75614200	-3.70894200	5.32798700
O	-3.93512100	-3.46250200	5.57759400
N	-1.83215800	-2.79290500	4.95148100
C	-2.20002200	-1.42771900	4.55887800
C	-0.97940700	-0.81877900	3.87053400
O	-0.68206000	0.36118600	4.01237500
O	-0.33292900	-1.72944100	3.17942300
O	-2.04981300	3.80057100	-2.98841900
O	3.81361500	3.96155100	-4.29235700
O	1.99655700	2.27180200	-5.41309000
O	5.46991000	1.60868800	1.25545700
O	-5.10460300	0.18361000	-2.07119300
O	-3.13966700	-2.92508800	1.38462100
H	6.31663700	-1.55030200	5.21300800
C	5.49116600	-0.90795900	5.54913900
H	5.28834000	-1.14663700	6.60079800
C	4.24359300	-1.10675100	4.68323900
C	4.51724300	-0.78693100	3.21174300
C	3.30246600	-0.86970800	2.30754900
O	2.37661500	-1.68109200	2.64097000
O	3.30609200	-0.14168200	1.26416800
H	4.67577900	3.94888400	9.04230800
C	3.66017400	4.35303200	8.93854800
C	3.14944900	4.22871700	7.47802400
C	3.08538500	2.77852800	6.94497900
C	2.26350800	2.53660200	5.65849700
N	2.95566900	2.87072900	4.40281400
C	2.43684400	2.59676400	3.17367100
N	1.28367900	1.96631900	3.03197100
N	3.09391200	3.02225300	2.06622900
O	5.82569200	-1.01674100	-0.01301900
H	-8.69202300	6.37838500	2.00521900
H	-7.02429200	6.96947200	2.06075800
H	-8.28937300	7.95311900	1.28932300
H	-6.88647200	6.82226600	-0.45592700
H	-8.55007100	6.25038700	-0.51452600
H	-9.06686000	4.02580000	0.82385600
H	-8.27824900	1.69020000	1.07935200
H	-4.32234200	2.89215000	-0.09192000
H	-5.10461000	5.24335200	-0.32357500
H	-6.55357600	0.20346600	0.61871900
H	-8.57755700	0.18051700	-1.43687800

H	-7.26211500	-2.69282500	1.32591900
H	-0.99740900	-3.14741200	4.49324700
H	-0.27020900	-5.93991700	5.19909700
H	5.35199000	-1.26689400	-6.28208200
H	-7.83289800	-3.22015200	-4.14630500
H	-9.88041600	-4.54848400	-2.47273300
H	-11.01474800	-2.41331000	-2.08852100
H	-9.92896700	-1.54803500	-3.16611200
H	-3.55723700	-3.07177900	-3.68654200
H	-5.47117900	-2.30145400	-2.22391900
H	-5.43978800	-5.31784300	-2.85696000
H	-3.19508300	-4.36557800	-2.55853900
H	-5.09677200	-3.58328000	-1.06664800
H	7.26109300	-4.46556600	2.36290000
H	3.44331900	-4.00701000	1.79806900
H	4.67896600	-3.70347700	-2.24624300
H	1.23872800	-1.07398800	-3.72573400
H	1.67632100	-5.00090000	-2.28916800
H	4.12212400	-2.82932600	-7.57881600
H	7.02719300	-0.66909400	-4.75514800
H	-0.48082600	5.72632200	-1.37812500
H	-0.28217500	7.25352600	1.13703700
H	2.31664100	8.90019100	0.00857000
H	-1.10881700	8.63476300	-0.60010000
H	6.81962800	4.48908600	0.85449300
H	1.70773500	-2.78713700	-6.84619100
H	2.64558700	-1.44925500	-6.20202400
H	0.89464800	-6.29584200	3.22976100
H	-2.98717800	-1.47540000	3.79056000
C	-2.68500100	-0.58816300	5.73315200
H	-2.94595900	0.41600000	5.38439800
H	-3.56416100	-1.06031100	6.18035800
H	-1.89988000	-0.49564700	6.49375500
H	-1.91426000	-5.21105700	6.60511900
H	-3.05832400	-5.80333100	5.38604300
H	0.82901400	-6.77822300	0.74985400
H	-0.87908000	-6.35926200	0.71499400
H	-2.55833300	4.65214500	-0.14776700
H	-1.72975000	5.15032800	1.32465000
H	5.84315100	0.13190000	5.50245100
H	3.87175200	-2.13515700	4.77456300
H	5.25551300	-1.48494400	2.78496000
H	3.43219100	-0.45879600	5.04656400
H	4.95913300	0.20950800	3.09970800

H	8.27729300	3.61966100	-1.72876300
H	7.77666800	2.55023900	-0.40944200
H	5.26949300	-6.01106400	2.58458000
H	5.86159200	-6.97809300	1.24084800
H	2.96559100	-2.89775100	-0.33253700
H	3.67982200	5.40392400	9.24965300
H	3.78316500	4.84495000	6.82181800
H	2.60829000	2.15711200	7.71642900
H	2.00827900	1.47048400	5.60618000
H	3.00636600	3.80409300	9.62873600
H	2.14048300	4.66437600	7.42191400
H	4.09739400	2.36764100	6.81339500
H	3.64389800	3.61326600	4.42726100
H	1.31318300	3.09002000	5.72405400
H	4.11019400	2.87789300	2.05286200
H	0.69836000	1.60649700	3.78382100
H	2.66952700	2.69486800	1.18252800
H	1.04374000	1.53661300	2.13050700
H	4.75914800	-1.88769900	-3.08055500
H	5.19781200	1.12324400	-3.42704300
H	6.03629300	-0.92739500	-2.35800200
H	4.18113800	0.22259600	-4.54115100
H	2.84112000	-4.85814200	-4.58146900
H	0.90788800	3.14503200	-4.10426200
H	3.70967400	3.65216600	-1.29247600
H	5.43218900	2.24947600	0.48245900
H	4.65015500	1.07595800	1.15158700
H	4.96332500	-0.85403800	-0.44367400
H	5.99561400	-0.15686200	0.42964100
H	-3.13972100	10.08393900	-1.00739400
H	-3.69812500	8.76518400	-2.07247900
H	-5.42283300	0.37674500	-1.16232100
H	-4.38626700	-0.47105200	-1.90910500
H	-3.33066200	-1.15027500	1.48247900
H	-2.61418500	2.51647700	-2.04258700
H	-2.28335900	4.67498700	-2.62230100
H	-1.05347800	3.75631700	-3.08967000
H	-4.09076600	0.21754700	1.23628400
H	-3.80201900	1.43964600	-1.86659700
H	2.09557500	1.62811100	-4.68494700
H	2.72445000	2.90836100	-5.16181400
H	3.22629800	4.06961300	-3.50925400
H	4.62246300	3.58934800	-3.86396600
H	-2.98900100	-3.60298900	2.07332600

H	-2.24032600	-2.78751500	1.00025800
H	-8.59773700	-3.89247900	-0.47466800
H	6.22003500	-5.35060400	-1.08786300
O	-0.17025400	1.53619200	-1.98155300
H	2.51434600	4.79382600	-1.42813600

$S_3^{A,W}Y_z$ (W1=H_2O, W2=H_2O, Ox=oxo)

Mn	-0.10751200	-2.50539800	-1.10149700
Mn	0.57082400	-1.77504100	1.52450300
Mn	1.97789500	0.05356100	-0.08955600
Mn	1.77788100	2.37938200	-1.49623300
Ca	-1.43409700	0.33901600	-0.30396900
O	-0.87036700	-1.93467700	0.51753500
O	0.73089300	0.02551600	1.22144000
O	1.52422300	-1.85914700	-0.13473200
O	2.62583900	1.79174000	-0.02389200
O	-0.38997400	-1.11161800	-1.94987600
O	2.92742000	3.87679500	-1.83365800
O	0.62530000	2.73786200	-3.13567000
O	-2.60581300	1.84217700	-1.73724700
O	-3.19012400	0.07231900	1.36119500
H	7.73412200	5.07094000	-0.55132300
C	7.81683600	4.07503800	-0.09888700
H	8.83381100	3.96448600	0.29608100
C	7.52538800	2.97380600	-1.13064000
C	6.16163100	3.11227300	-1.77900300
O	5.20857200	3.41675300	-0.89680600
O	5.96648500	2.94984000	-2.98012500
C	-7.46203900	7.37395300	1.52015000
C	-7.07706200	6.75045200	0.16724000
C	-6.67793500	5.29056900	0.25249600
C	-7.57760000	4.32648200	0.73059400
C	-5.41167300	4.85266800	-0.16270600
C	-7.23605100	2.97563800	0.79694200
C	-5.05194400	3.50373500	-0.11164600
C	-5.96881700	2.56333100	0.36732700
O	-5.60380100	1.23183900	0.38252500
H	2.20779400	8.12970800	1.47259200
C	2.61736600	7.78106000	0.51739800
C	1.63743900	6.97201300	-0.31024400
O	1.95071100	6.51016300	-1.40696600
H	3.50680600	7.16936900	0.70740900
N	0.36428000	6.83180600	0.19627200
C	-0.66806900	6.11387200	-0.54495600
C	-1.72843600	7.02996100	-1.15986100

O	-2.65529800	6.53578700	-1.82707000
C	-1.24014000	4.90280400	0.21339000
C	-0.44158100	3.58888400	0.07909000
O	-0.87098000	2.57102900	0.63068200
O	0.63236200	3.67486500	-0.64591600
N	-1.52492000	8.35030300	-1.00588400
C	-2.26117700	9.38765300	-1.70989800
H	-1.61314200	9.89842700	-2.43310400
H	-6.13246400	-3.68321000	-3.92179900
C	-6.23073600	-3.99768300	-2.87191700
C	-7.68783000	-4.01769600	-2.46303000
O	-8.07239300	-4.60316300	-1.44270500
C	-5.32588500	-3.12917700	-1.99683100
C	-3.90340900	-3.30465900	-2.52976400
C	-2.83150600	-2.57810600	-1.75487200
O	-3.02280200	-1.42191300	-1.32898500
O	-1.74517000	-3.27355800	-1.62972800
N	-8.53993100	-3.34900300	-3.29335200
C	-9.93851700	-3.13732800	-2.96040600
H	-10.55108500	-3.22728000	-3.86483000
C	-10.19301800	-1.75955800	-2.29233500
C	-9.20352300	-1.38936800	-1.22630800
C	-8.53083900	-0.21379500	-0.97583800
N	-8.77333600	-2.26161300	-0.23588300
C	-7.85373500	-1.61569100	0.52642500
N	-7.69940800	-0.36414900	0.12001000
H	3.29392800	-4.65710400	-6.86389000
C	3.44417300	-3.58711100	-6.67803800
C	4.47885100	-3.45722300	-5.56755500
O	4.52459900	-4.25984800	-4.62361900
C	2.11081200	-2.92018000	-6.24021100
C	1.67320400	-3.24320100	-4.84366000
C	1.18032600	-2.43935900	-3.84577000
N	1.65455100	-4.53100800	-4.31373300
C	1.19161500	-4.47239100	-3.04087300
N	0.88545100	-3.21828500	-2.74158500
N	5.27986100	-2.36651000	-5.63889800
C	6.16033000	-1.98790900	-4.53814400
H	6.70473300	-2.88163200	-4.21575400
C	5.37997000	-1.40983600	-3.33009500
C	4.58844900	-0.12688100	-3.61742600
C	3.60637800	0.30923500	-2.52621400
O	3.40159000	-0.42070900	-1.51235400
O	3.03082100	1.43019900	-2.73433300

H	7.43064500	-6.09499400	0.87481000
C	6.89087500	-6.00125500	1.82627900
H	7.29587100	-6.75086700	2.51490500
C	5.37382300	-6.21812900	1.64569300
C	4.68574100	-5.23821500	0.74300600
C	3.69538100	-4.31974300	0.98709800
N	4.94145600	-5.13226700	-0.62368400
C	4.14447900	-4.19375900	-1.17611000
N	3.38738400	-3.69183000	-0.20555600
H	-0.81209500	-7.56420000	2.74285300
C	-0.46684600	-6.52105100	2.72313200
C	-1.53636000	-5.66639900	3.41359800
O	-2.49135800	-5.19072000	2.79948000
C	-0.26466600	-6.09393800	1.26637100
C	0.22463400	-4.67246300	0.95575300
O	0.48574700	-3.84773700	1.89305000
O	0.32701000	-4.41626200	-0.27762300
N	-1.38735000	-5.49632900	4.76227200
C	-2.48585900	-5.01487700	5.59937600
C	-2.93541500	-3.56730900	5.35536300
O	-4.07895200	-3.23210400	5.64768700
N	-1.96917000	-2.72854300	4.90672900
C	-2.24561400	-1.32865000	4.58565200
C	-0.98498400	-0.76011800	3.90125200
O	-0.62425300	0.39507700	4.11769200
O	-0.38394900	-1.64273100	3.14208600
O	-1.76926200	4.20760800	-2.99644200
O	3.90824100	4.40483100	-4.31625100
O	2.20099900	2.58544600	-5.16249900
O	5.43136900	1.61827800	1.27213100
O	-4.81371500	0.45634000	-2.20878200
O	-3.29787000	-2.67064700	1.52826600
H	5.63567000	-2.40085400	5.46790900
C	5.47828500	-1.31373100	5.44722900
H	5.29202100	-0.98464800	6.47773100
C	4.31586000	-0.94723500	4.52798400
C	4.59050500	-1.38465000	3.08021700
C	3.36819700	-1.20492600	2.21169200
O	2.34156600	-1.88144200	2.51113900
O	3.44623000	-0.38442000	1.23722000
H	5.01240100	3.53178200	8.85570100
C	4.05480200	4.06902600	8.84098000
C	3.35044800	3.91450800	7.47914100
C	3.03847900	2.45186600	7.10980300

C	2.19966300	2.26443700	5.83662200
N	2.94578100	2.66717200	4.63926100
C	2.47438100	2.56915600	3.37483200
N	1.29671400	2.02970100	3.10736900
N	3.22427100	3.09113700	2.36864500
O	6.07589800	-0.78147500	-0.19302800
H	-8.31204300	6.84849900	1.97328200
H	-6.62498800	7.31888900	2.22828200
H	-7.74454200	8.42793600	1.40073600
H	-6.24947100	7.32155900	-0.27657600
H	-7.92651800	6.85856800	-0.52519500
H	-8.57159000	4.63306300	1.05458900
H	-7.94574100	2.24032200	1.16925600
H	-4.08537400	3.15680700	-0.47163600
H	-4.69994600	5.57571800	-0.56206100
H	-6.43889700	0.63874200	0.43424200
H	-8.59344400	0.72806700	-1.50626100
H	-7.31888600	-2.08856200	1.34012600
H	-1.15782600	-3.13299400	4.44927200
H	-0.61142200	-5.96155600	5.21634500
H	5.17443200	-1.73352400	-6.42142000
H	-8.13739800	-2.80084100	-4.04382000
H	-10.21654500	-3.95140600	-2.28408100
H	-11.22074800	-1.76206700	-1.89692100
H	-10.16077000	-0.97031200	-3.05510500
H	-3.84775900	-2.92745800	-3.56437800
H	-5.63263400	-2.07693300	-2.04220700
H	-5.88348800	-5.03831400	-2.81794200
H	-3.63804300	-4.36730600	-2.56797500
H	-5.38387900	-3.44813500	-0.94879800
H	7.09841800	-5.00447500	2.23269100
H	3.16384000	-4.06265800	1.89275900
H	4.12944000	-3.93982900	-2.22912700
H	0.96430000	-1.37985600	-3.82169400
H	1.06248000	-5.31313700	-2.37476100
H	3.78187100	-3.13538900	-7.61875600
H	6.88791100	-1.26505700	-4.92358800
H	-0.13222700	5.71957100	-1.41855800
H	0.19502500	7.07152600	1.16706000
H	2.93656900	8.64641000	-0.07477700
H	-0.68946500	8.60840500	-0.49369500
H	7.11516400	4.02032500	0.73954700
H	1.33178000	-3.20206800	-6.96346800
H	2.20781100	-1.82925600	-6.30425900

H	0.47600800	-6.47770800	3.28540600
H	-3.04346300	-1.28312500	3.82774700
C	-2.64682400	-0.51017800	5.80908400
H	-2.82488800	0.52827400	5.51340700
H	-3.55641000	-0.93083900	6.24480900
H	-1.84640100	-0.52570100	6.55942400
H	-2.16960800	-5.09924300	6.64494700
H	-3.37854900	-5.63577600	5.46016700
H	0.42362600	-6.78528600	0.76176300
H	-1.22258900	-6.17440400	0.73759900
H	-2.24852400	4.67867400	-0.15309500
H	-1.35605700	5.11191600	1.28556700
H	6.41426600	-0.84806100	5.11047600
H	3.39000900	-1.42659200	4.87231200
H	4.84298300	-2.45501300	3.06816700
H	4.14244400	0.13903900	4.54898900
H	5.43277900	-0.83881500	2.64482100
H	8.26815900	2.96392800	-1.93507800
H	7.55798200	1.98651200	-0.64500000
H	4.87602200	-6.15765500	2.62148000
H	5.19143900	-7.23795100	1.27415100
H	2.63690500	-2.91877100	-0.27974400
H	4.25556100	5.12427900	9.05978300
H	3.97921800	4.37892700	6.70157500
H	2.47391000	1.99178700	7.93257400
H	1.91934600	1.20908000	5.72463200
H	3.43198400	3.66695900	9.65067900
H	2.40925100	4.48485900	7.48802900
H	3.97193200	1.87605800	7.01532000
H	3.70061300	3.33013300	4.76997700
H	1.26672400	2.84523300	5.91096900
H	4.23174400	2.92205200	2.42845900
H	0.69128600	1.54387000	3.78097700
H	2.89621700	2.81596000	1.42929400
H	1.09287600	1.70768800	2.15777400
H	4.67423200	-2.17434300	-2.99501300
H	5.24840600	0.73447900	-3.78980800
H	6.07585800	-1.23307000	-2.50047100
H	3.98227600	-0.24479800	-4.52987400
H	2.12896000	-5.32652100	-4.72188900
H	1.16016700	2.80802300	-4.01123300
H	4.26278300	3.59228500	-1.33166700
H	5.29632900	2.23459000	0.51522400
H	4.63759500	1.03931200	1.24113500

H	5.13127100	-0.80700400	-0.43251400
H	6.13218200	0.03227200	0.35247100
H	-2.66251800	10.12257700	-1.00248600
H	-3.08846000	8.91078800	-2.24148700
H	-5.19709800	0.71660300	-1.33924300
H	-4.28322000	-0.34628300	-1.98294500
H	-3.38973200	-0.88718300	1.51827300
H	-2.34957500	2.64130600	-2.24780900
H	-2.16534100	5.01569600	-2.56666500
H	-2.08295700	4.22656100	-3.91713700
H	-4.05010700	0.51287300	1.17971600
H	-3.46157500	1.47042600	-2.10274100
H	2.63109000	1.88966000	-4.62742500
H	2.85178300	3.34861200	-5.02575500
H	3.45107600	4.37599000	-3.43811900
H	4.72133200	3.89932900	-4.10121500
H	-3.22367200	-3.37651600	2.20001700
H	-2.39490900	-2.61933000	1.13591400
H	-8.85561900	-3.27961600	-0.30056400
H	5.63526700	-5.66136500	-1.13891500
O	0.86541800	0.83396200	-1.26032000
H	2.52699800	4.71663200	-1.51952900
H	-0.03183700	3.46564700	-3.04774000
